# A Novel Strategy to Identify Prognosis-Relevant Gene Sets in Cancers

**DOI:** 10.3390/genes13050862

**Published:** 2022-05-12

**Authors:** Junyi Pu, Hui Yu, Yan Guo

**Affiliations:** 1 School of Life Sciences, Northwest University, Xi’an 710069, China; junyi_pu@163.com; 2 Comprehensive Cancer Center, New Mexico University, Albuquerque, NM 87131, USA; huiyu1@salud.unm.edu

**Keywords:** cancer prognosis, pathway, gene sets

## Abstract

Molecular prognosis markers hold promise for improved prediction of patient survival, and a pathway or gene set may add mechanistic interpretation to their prognostic prediction power. In this study, we demonstrated a novel strategy to identify prognosis-relevant gene sets in cancers. Our study consists of a first round of gene-level analyses and a second round of gene-set-level analyses, in which the Composite Gene Expression Score critically summarizes a surrogate expression value at gene set level and a permutation procedure is exerted to assess prognostic significance of gene sets. An optional differential coexpression module is appended to the two phases of survival analyses to corroborate and refine prognostic gene sets. Our strategy was demonstrated in 33 cancer types across 32,234 gene sets. We found oncogenic gene sets accounted for an increased proportion among the final gene sets, and genes involved in DNA replication and DNA repair have ubiquitous prognositic value for multiple cancer types. In summary, we carried out the largest gene set based prognosis study to date. Compared to previous similar studies, our approach offered multiple improvements in design and methodology implementation. Functionally relevant gene sets of ubiquitous prognostic significance in multiple cancer types were identified.

## 1. Introduction

The quote of mathematician Henri Poincare, “It is far better to foresee even without certainty than not to foresee at all,” has obvious and acute relevance to cancer prognosis. Cancer prognosis entails the estimation of development course, speed, and outcome of cancer progression, which provides the most critical information for the patients and family to make life decisions [1]. A very important prognosis statistic is disease survival, which can be further classified into overall survival (OS) and disease specific survival (DSS). OS and DSS record the timespan from the diagnosis of disease to the (disease-caused) death of the patient. It takes a rather long follow-up time to record OS/DSS for a patient cohort, and many patients may contribute censored survival time due to loss of contact or living longer than the observation period. However, the obtained survival data are very valuable in cancer research, and researchers have always been correlating measurable traits of patients with their survival in the hope of finding effective prognosis predictors. Oncologists and physicians rely on relevant clinical factors to make their best prognosis predictions. Patients’ age, gender, and ethnicity often affect cancer prognosis [2,3]; lab measurements such as lymph node status, tumor size, and histologic grade are also important factors.

In addition to conventional clinical factors, genomic characteristics such as gene expression and somatic mutation hold promise for molecular markers for survival prediction [4,5,6]. In the simplest scenario, a single gene or gene product may confer significant prognostic value, of which representative examples include Prostate-Specific-Antigen for prostate cancer and CA 125 for ovarian cancer. More often, a set of genes, usually with coherent functions, are deemed as a unit (“signature”) that collectively provides the prognosis signal. Well known gene signatures for cancer prognosis prediction include MammaPrint (70 genes) [7] and Oncotype DX Breast Recurrence Score (21 genes) [8] for breast cancer and VeriStrat test [9] for non-small cell lung cancer. Gene set biomarkers received increasing attention from researchers and pharmaceutical developers, because the process of tumorigenesis, progression, and metastasis are consequences of disrupted cellular functions, each of which hinges on the coordination of a set of genes [10]. Compared to single gene markers, a prognostic gene set of coherent functions provides unparalleled interpretability and mechanistic clues. Ideally, a gene set should be defined to correspond to a cellular pathway whose members are activated in concert to perform a coherent biological function. In practice, however, gene sets are defined from many different angles [11]. The Molecular Signatures Database (MSigDB) [12] collects various types of annotated gene sets for transcriptome analyses at gene set level, most often in combined use with Gene Set Enrichment Analysis (GSEA) [13].

Development of gene set prognostic markers is still at an early phase, with few clinical applications limited to select cancer types. It is imperative to carry out a unified bioinformatics screening of prognostic gene sets using pan-cancer datasets so that the disparity in prognostic research of diverse cancer types can be diminished. Previously, our group devised a promising gene set survival analysis method based on Composite Gene Expression Score (CGES) [14]. The CGES approach involved a first round of gene-level analyses and a second round of gene-set-level analyses. In the present study, we applied the CGES approach to transcriptome and survival data of 33 cancer types and systematically screened for prognostic gene sets from all MSigDB gene sets. The CGES survival analyses were appended with two differential coexpression analyses from different angles. Overall, our work demonstrated a novel strategy to identify the most prognosis-relevant cancer gene set. Computational code for the proposed analysis strategy is available in a GitHub repository (https://github.com/hui-sheen/proggset) (accessed on 6 April 2022).

## 2. Materials and Methods

### 2.1. Data Acquisition

Gene expression data (FPKM) of over 10,000 patients from 33 cancer types were obtained from Genomic Data Commons (GDC). OS data for TCGA was available from GDC. However, given that OS does not account for cancer-specific death, we chose DSS as our primary survival outcome variable, which was obtained from a publication by Liu et al. [15]. A full list of 32,234 gene sets was obtained from MSigDB (v7.4) (UC San Diego, San Diego, U.S.). These gene sets are organized into eight categories: C1. Positional gene sets (N = 278); C2. Curated gene sets (N = 6290); C3. Regulatory target gene sets (N = 3731); C4. Computational gene sets (N = 858); C5. Ontology gene sets (N = 14,998); C6. Oncogenic signature gene sets (N = 189); C7. Immunologic signature gene sets (N = 5219); C8. Cell type signature gene sets (N = 671).

### 2.2. Using GSEA to Detect Enrichment of Single Gene Prognostic Signals

GSEA is commonly used to test whether a set of genes collectively show statistically significant differences between two biological phenotypes [13]. GSEA requires two inputs: a ranked gene list (commonly ranked by expression fold change) and a gene set. The set of genes is projected to a ranked gene list to reveal whether there is significant enrichment at either end of the gene ranking. We utilized GSEA with an alternative approach by using a gene list ranked by survival analysis *p*-values rather than fold changes. Single gene survival analysis was conducted with Cox proportional hazard model in R. The survival *p*-values of single genes were sorted and supplied to GSEA, along with one gene set each time. Because the primary usage of the *p*-values in this context was ranking rather than statistical assertion, no multiple test correction was needed. By default, the GSEA method surveys for enrichment at both ends of the gene ranking. However, because only survival significant genes were of interest, we scanned only one end of the *p*-value list to select gene sets enriched for survival significance. GSEA analysis was conducted using R package fgsea [16].

### 2.3. Gene Set Survival Analysis

Gene set survival analysis was conducted using the Composite Gene Expression Score (CGES) method [14]. Briefly, CGES is a surrogate for the expression of gene sets. It is computed using the inverse-log transformation of a linear combination of individual gene expression values, which can be expressed using the following formula: $CGES=\frac{1}{\left[1+\exp\left(-\left(\sum_{i=1}^{k}\beta_{i}x_{i}-m\right)\right)\right]}$, where $x_{i}$ is the standardized log-transformed expression value of one gene (the ith out of a k-gene set), and the coefficient $\beta_{i}$ is the score test statistic for the same gene from the prior univariate Cox proportional hazard regression. The normalization factor *m* captures the median of the linear combination term ($\sum_{i=1}^{k}\beta_{i}x_{i}$) across all patients of a cancer type. To correct for overfitting, we conducted leave-one-out cross validation (LOOCV) for 1000 iterations. The permutation *p*-value is computed as $p_{perm}=\frac{\#\left(\chi_{perm}^{2}\geq \chi_{obs}^{2}\right)\;}{\#Perm}$, where $\#\left(\chi_{perm}^{2}\geq \chi_{obs}^{2}\right)$ is the number of times that the log-rank test statistic values ($\chi_{perm}^{2}$) calculated from permutated data are greater than or equal to that ($\chi_{obs}^{2}$) calculated from the real data, and #Perm is the total number of permutations.

### 2.4. Meta Differential Coexpression Analysis

Gene expression correlation indicates functional dependence, and coexpression changes may reflect systematic cellular function changes [17,18]. The credibility of a prognostic gene set would be strengthened if it showed differential coexpression signals between the normal and tumor samples. We previously developed MetaGSCA [19] as a tool to assess gene sets’ differential coexpression significance across multiple studies. In the inner layer, MetaGSCA enrolls a differential coexpression algorithm GSNCA [20] to evaluate the degree of gene–gene re-wiring between two phenotypes; in the outer layer, MetaGSCA wraps a meta-analysis framework around the inner algorithm to estimate an overall effect over individual studies and constructs nonparametric confidence intervals via bootstrapping. Here, to affirm the ubiquitous relevance of a gene set across multiple cancer types, we used MetaGSCA (v0.99.6) (Guo Bioinformatics Lab, Albuquerque, U.S.) to perform meta differential coexpression analysis on 14 TCGA transcriptome datasets that contained both tumor and matched normal samples. To promote brevity and relevance of the results, we only included gene sets of prognostic value in five or more individual cancer types. As for meta-analysis parameter setting, we used the GLMM model and assumed a random effect; permutation and bootstrap were each performed 100 times.

In our previously developed R package DCGL [21], we developed a robust statistical approach (DCe) [22] to discern differentially coexpressed links of genes. DCGL models the relationship between maximum coexpression and log coexpression ratio of links and deemed as differentially coexpressed links the outliers that significantly betray the fitted boundary curve. Using DCGL (v2.1.2) (Shanghai Center for Bioinformation Technology, Shanghai, China), we sought for differentially coexpressed links among all genes involved in the prognostic-significant gene sets, in datasets of each cancer type separately. Consequently, differentially coexpressed links (gene pairs) were generated for each cancer type. The differentially coexpressed links of different cancer types were merged into one background gene network to allow for the subsequent gene set crosstalk analysis.

### 2.5. Gene Set Crosstalk

A live cell is dictated by a hierarchical organization of modular functions [23]. The coordinate or antagonistic relationships among modular functions are subject to systemic regulation, and changes in the organization of functional modules may be associated with phenotypic changes. A variety of strategies have been proposed to interrogate pathway crosstalk in normal and pathophysiological states. Here, we followed a workflow that has been applied to study attenuated pathway crosstalk in chronic kidney disease [24] and schizophrenia [25]. First, the differentially coexpressed links were divided into the correlation-strengthened class and the correlation-weakened class. Gene links of the same class were merged across cancer types, and they formed a gene-level network that represented strengthened (or weakened) gene–gene connections. Next, we applied the Characteristic Sub Pathway Network (CSPN) algorithm [26] to identify statistically significant pathway–pathway connections. We performed pathway crosstalk analyses for the strengthened and weakened gene links separately so that the resultant pathway-level network represented either enhanced or attenuated function coordinations, but not both. In parallel to the CSPN approach, we also exerted the inherent gene set crosstalk analysis from MetaGSCA to derive another pathway crosstalk network. Unlike the CSPN-derived pathway network, the MetaGSCA-derived pathway network captures the similarity between gene sets in their differential coexpression significances across the cancer panel.

## 3. Results

### 3.1. Overall Strategy

**Figure 1:**
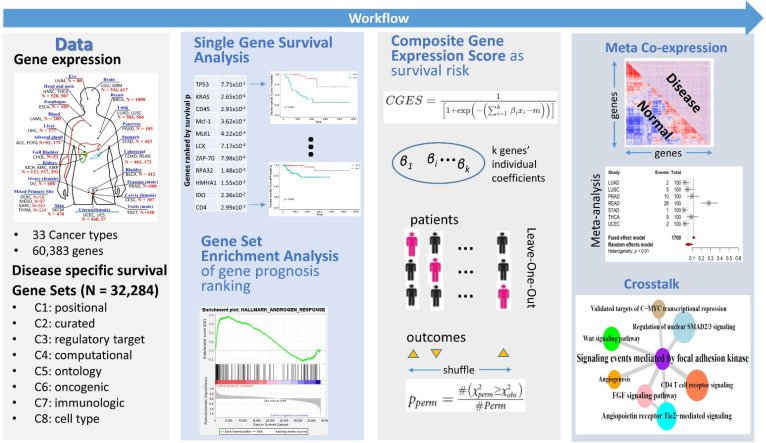
The strategy to identify prognosis-relevant gene sets in cancers. Panel 1: Transcriptome data of tumor samples and patient survival data (disease specific survival) were obtained from The Cancer Genome Atlas, and compilation of 32,284 gene sets was obtained from MSigDB. Panel 2: transcriptome data and survival data underwent the single-gene survival analysis step, which generated the Cox model *p*-values of each gene; the ranked *p*-value list was analyzed with the GSEA approach on the individual gene sets so that each gene set received an enrichment *p*-value. Panel 3: for each gene set with a nominally significant enrichment *p*-value, we employed CGES to assess if it has prognostic prediction power. CGES constructs a surrogate expression value for a gene set as a logistic transform of weighted sum. A Leave-One-Out procedure was imposed to calculate the CGES score for each patient. Patient survival outcomes were permutated many times (e.g., 1000) to derive the final gene set *p*-values. Panel 4: The difference in coexpression was interrogated in each cancer type and meta-analysis was conducted across cancer types. Gene set crosstalk analysis was performed with a post hoc module of MetaGSCA or through the combined use of DCGL and CSPN.

The overall strategy to identify prognosis-relevant gene sets is depicted in Figure 1. Our strategy involves three types of data: gene expression, survival data, and gene sets. To demonstrate the strategy, we used gene expression data from 33 cancer types, DSS data, and 32,234 gene sets. The process consisted of the following steps: (1) single gene survival analysis; (2) GSEA analysis based on single gene survival analysis results; (3) gene set survival analysis based on CGES; and (4) optionally, a meta coexpression and crosstalk analysis to further evaluate the gene sets selected across cancer types.

### 3.2. Single Gene Survival

**Figure 2:**
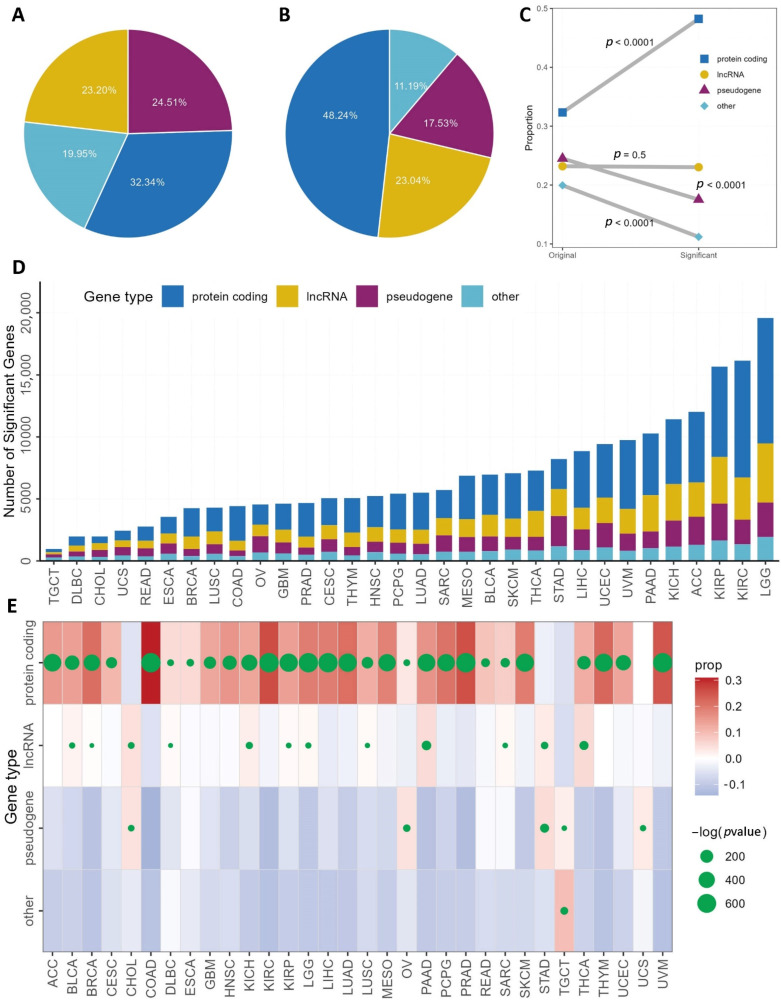
Single gene survival analysis results. (**A**) A pie chart that depicts the original composition of gene types for the 60,483 genes. (**B**) A pie chart depicts the survival significant genes’ composition by gene type. (**C**) A line and dot plot that shows how gene composition changed and chi-square tests results. (**D**) A barplot that shows the detailed survival significant genes by gene type and cancer type. (**E**) A dot plot that shows the results of chi-square tests by cancer type and gene type. The background color indicates whether the proportion of the gene type increased (redder) or decreased (bluer). The size of the dot indicates the significance of the chi-square tests. A larger dot denotes a more significant *p*-value.

The gene expression data contained 60,483 genes (protein coding: 32.34%; lincRNA: 23.20%; pseudogene: 24.51%; other: 19.95%) (Figure 2A, Supplementary Table S1). We conducted single gene survival analysis by fitting DSS with Cox proportional hazard regression models. Similar analyses were repeated for each gene in each cancer type. The analyses yielded a total of 222,015 (protein coding: 48.24%; lincRNA: 23.04%; pseudogene: 17.53%; other 11.19%) significant associations (Figure 2B, Supplementary Table S1). Chi-square tests were used to test whether there is significant proportional difference between gene type proportion and significant gene type proportion (Figure 2C). The proportion of lincRNA stayed the same. The other types of RNA had significant shift in proportion. Protein coding genes occupied 32.34% of the original gene expression data, and its proportion increased to 48.24% in the survival significant associations (chi-square *p* < 0.0001). Pseudogene and other RNA type’s proportions decreased significantly. Low grade glioma had the greatest number of genes (19,579) associated with survival. Thyroid carcinoma had the lowest amount of genes (960) associated with survival (Figure 2D). The same trends of survival significance with gene type are also observed in individual cancer types (Figure 2E). Most cancer types showed enrichment of survival for protein coding genes. These results are within expectation and further solidify the importance of protein coding genes.

### 3.3. Gene Set Survival Analysis

**Figure 3:**
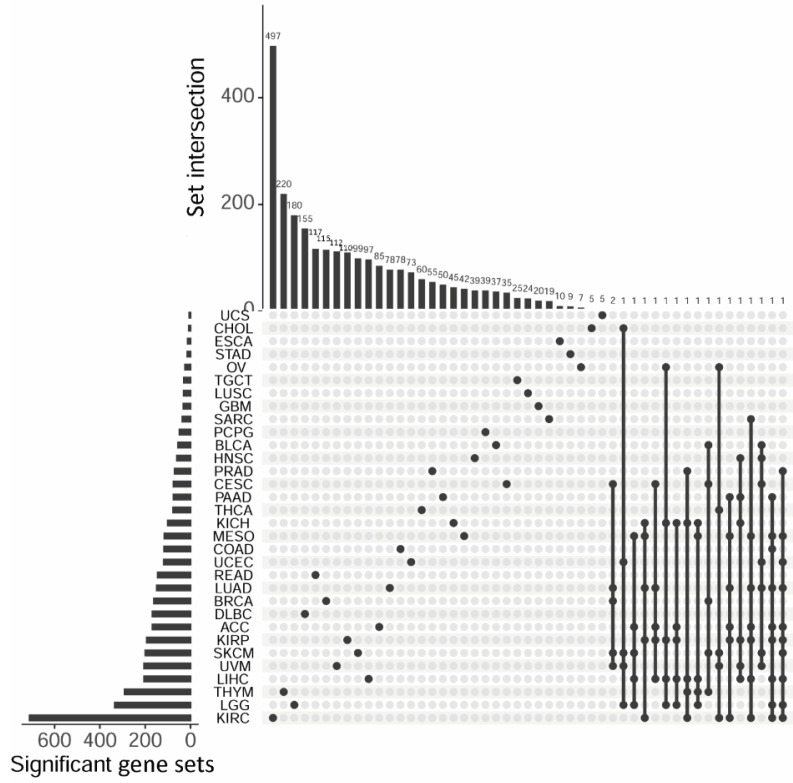
Prognostic gene sets were identified for 32 cancer types and 18 gene sets were shared by five or more cancer types. Due to space limitation, the vast intersections involving four or fewer cancer types were not indicated.

**Figure 4:**
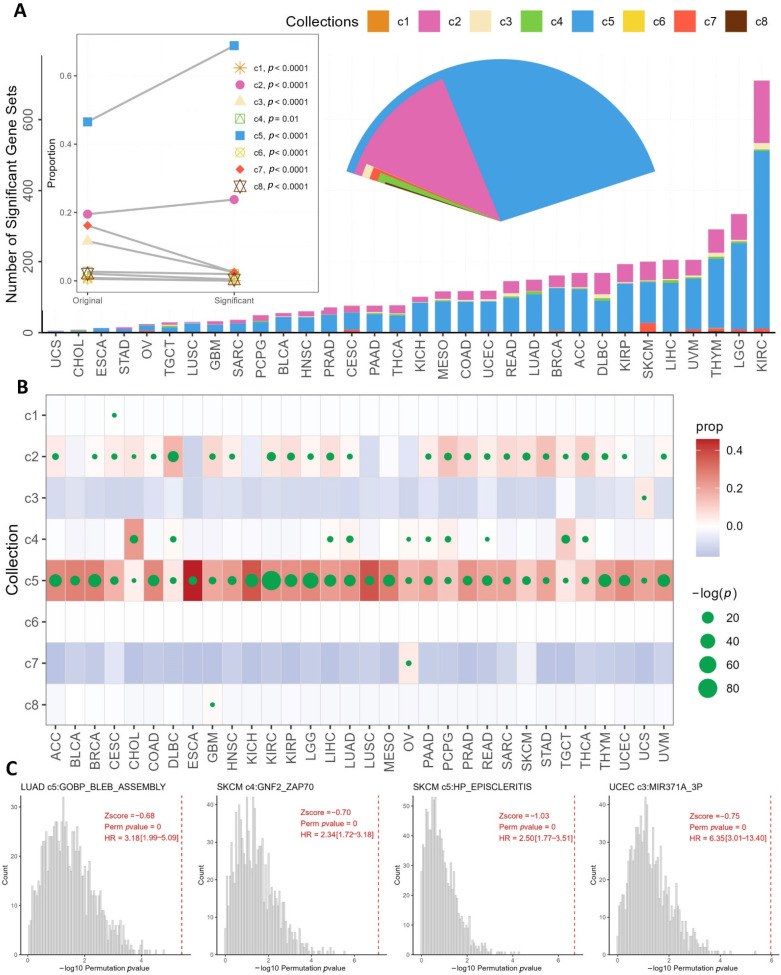
Gene-set-based survival analysis results. (**A**) Amounts of significant prognostic gene sets and their composition. Three subplots are enclosed: fan plot, dot and line plot, and bar plot (main content). The fan plot denotes the overall composition of survival significant gene set categories (c1 to c8). The dot and line plot depicts the gene set category proportion change from original to survival significant and the corresponding chi-square test results. (**B**) A dot plot that shows the results of chi-square tests by cancer type and gene set categories. The background color indicates whether the proportion of the gene type increased (redder) or decreased (bluer). The size of the dot indicates the significance of the chi-square tests. A larger dot denotes a more significant *p*-value. (**C**) Four examples of permutation tests using a histogram. The gray bars indicate the survival *p*-value from 1000 iterations of permutation. The red dotted line indicates the survival *p*-value based on the real gene set. The cancer and gene sets for these are examples are (from left to right): (1) lung adenocarcinoma, c5 GOBP BLEB ASSEMBLY; (2) skin cutaneous melanoma, c4 GNF2 ZAP70; (3) skin cutaneous melanoma, c5 HP EPISCLEPRITIS; and (4) uterine corpus endometrial carcinoma, c3 MIR371A 3P.

GSEA analysis was performed on genes ranked by survival significance on all available 32,234 gene sets from MSigDB. The gene sets were organized into eight categories: c1 to c8 (see Section 2 for detail). Of all 32,234 gene sets, 25,113 were significantly enriched for survival signal (*p* < 0.05). At this stage, these results were inflated by false positives due to the high number of tests conducted. Instead of conducting multiple test corrections, we utilized the CGES method with 1000 iterations of permutation to evaluate each gene set’s prognostic value. The permutation *p*-value out of CGES was cut off at 0.05 to affirm prognostic gene sets. As a result, the number of prognostic gene sets per cancer type ranged from 6 to 710, and a total of 3173 unique gene sets were associated with any cancer type (Figure 3, Supplementary Table S2). Kidney renal clear cell carcinoma was associated with the most prognostic gene sets (710), greatly surpassing the runner-up, Brain Lower Grade Glioma (334). The overall proportion of the gene set categories can be seen in Figure 4A (fan plot inset). The majority of the survival-enriched gene sets were c5 (gene ontology) followed by c2 (curated gene sets). Chi-square tests show that all gene sets had a significant shift in proportion after GSEA analysis (Figure 4A, dot and line plot inset). For example, c5 gene sets accounted for 46.5% of all GSEA gene sets, while the proportion of c5 in survival significant gene sets increased to 68.8% (chi-square *p* < 0.001). C7 gene sets (immunologic signature) accounted for 16.2% of the entire GSEA gene sets, while the survival significant c7 dropped to 2.5% (chi-square *p* < 0.0001). The chi-square tests were also performed for cancer type by gene set categories (Figure 4B). Notably, all cancer types showed an increased proportion of c5 gene set after GSEA analysis. Furthermore, of the 4038 significant survival associations, multiple gene sets had the equal permutation *p*-value of zero. To properly rank them, we computed z-score. Four examples of permutation results are shown in Figure 4C. In these four examples, all four gene sets had a permutation *p*-value at zero. However, their significance can be ordered by either original hazard ratio or z-score.

### 3.4. Pan-Cancer Differential Coexpression of Gene Sets

**Figure 5:**
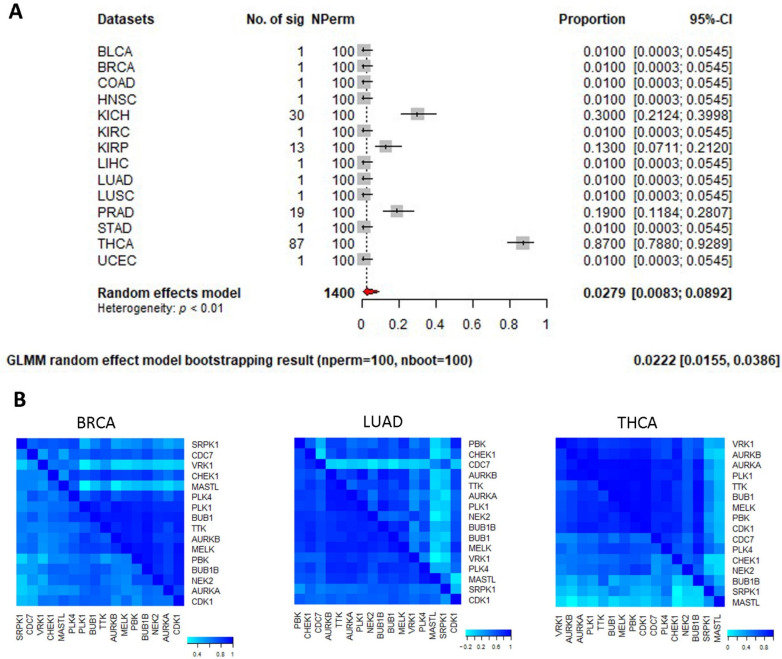
Meta differential coexpression analysis of gene set FINETTI_BREAST_CANCER_KINOME_RED (a C2 curated gene set from MSigDB). (**A**) Forest plot showing differential coexpression *p*-values in 14 cancer types. (**B**) Intra-set gene–gene correlation heatmap in three exemplar cancer types (BRCA and LUAD have *p*-values < 0.05 while THCA has a *p*-value > 0.05). In each heatmap, the lower and upper triangles represent the normal state and the tumor state, respectively.

Compared to conventional differential expression approaches where genes are evaluated individually assuming gene independence, differential coexpression analysis interrogates gene–gene co-transcription relations and exerts a complementary dissection into diseased transcriptomes. Multiple cancer types may share the same prognosis-relevant gene sets. For individual gene sets, MetaGSCA examines gene coexpression changes between tumor and normal. The MetaGSCA analysis was performed on the 18 MSigDB gene sets that demonstrated significant prognostic value in five or more cancer types (Figure 3 and Table 1). As a result, seven gene sets stood out as collectively differentially coexpressed across the 14 cancer datasets (bootstrap *p* < 0.05, Table 1). Intra-set gene correlation changes for these seven gene sets were shown as asymmetric heatmaps for representative positive cases and negative controls (Supplementary Figure S1). Of these seven gene sets, four were curated as oncogenic gene signatures in diverse studies, with regard to thyroid cancer [27], prostate cancer [28], breast cancer [29], and skin cancer [30]. For example, the gene set FINETTI_BREAST_CANCER_KINOME_RED consisted of 16 kinase genes that were originally curated to predict luminal breast cancers with poor prognosis [29]. With our CGES approach, we found these 16 genes conferred significant prognostic value in a total of seven cancer types (ACC, KIRC, KIRP, LIHC, LUAD, MESO, and SARC), and mutual expression correlations among the 16 genes were significantly changed from normal to tumor in 11 cancer types, including breast cancer (Figure 5A). We demonstrated the intra-gene-set gene–gene correlation values (Pearson correlation coefficient) for two prognosis-relevant cancer types (BRCA and HNSC) and one prognosis-irrelevant cancer type (THCA). Overall intra-set correlations were drastically changed in BRCA and HNSC, yet in THCA it was hard to discern such correlation change pattern (Figure 5B). The other three gene sets of significant pan-cancer differential coexpression were biological processes or cellular compartments pertinent to DNA replication and its necessary machinery.

**Figure 6:**
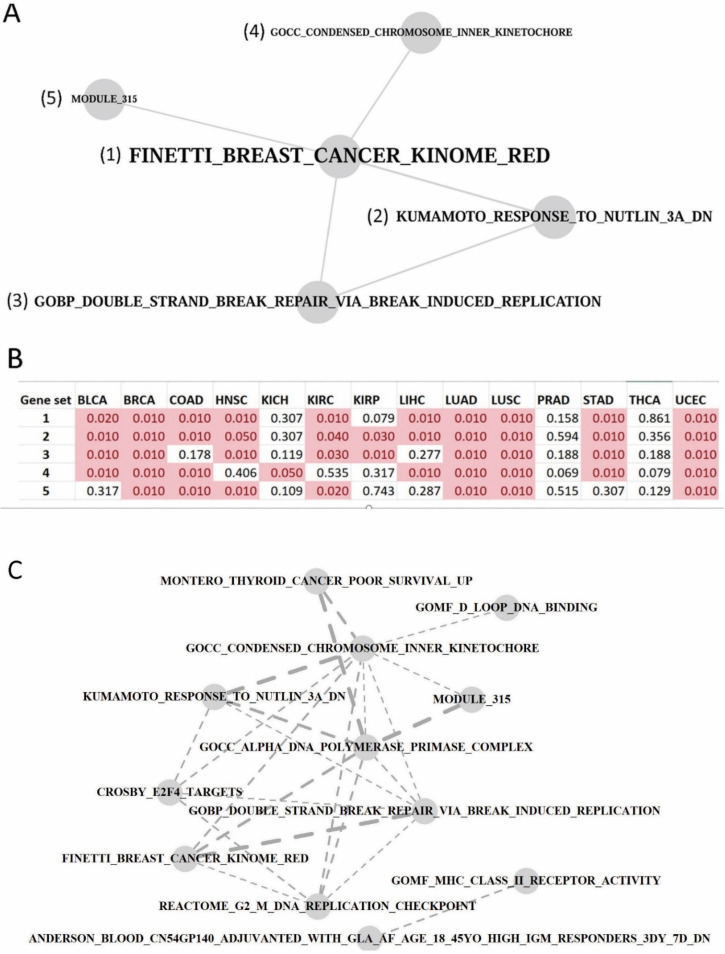
Gene set crosstalk network based on pan-cancer transcriptomes. (**A**) Gene set crosstalk network based on pan-cancer differential coexpression similarity. MetaGSCA was exerted to generate the network, and all *p*-value thresholds were set at 0.05. (**B**) For the five gene sets in the crosstalk network above (refer to number index), MetaGSCA-calculated differential coexpression *p*-values in individual cancer types. *p*-values less than 0.05 were highlighted in pink shading. (**C**) Gene set crosstalk network based on pan-cancer weakened gene–gene connections. DCGL and CSPN were collectively utilized to generate the network. Dashed edges were employed deliberately to indicate that coordination among gene sets was attenuated in cancer compared to normal. Edge width is proportional to the statistical significance of the between-set connection (all *p*-values were less than 0.05).

The concerted coordination among discrete cellular functions may be disrupted in pathophysiological states. Therefore, we adopted two distinct strategies to delineate gene set crosstalk networks in the pan-cancer context. On one hand, we employed MetaGSCA to perform a *post hoc* gene set crosstalk analysis of differential coexpression profiles. MetaGSCA inferred the similarity between two gene sets based on their differential coexpression profiles across individual cancer datasets, so the resultant network (Figure 6A) informed that the five gene sets included had overall similar coexpression changes across the 14 cancer types. These five gene sets showed differential coexpression in a majority of the 14 cancer types, including BLCA, BRCA, COAD, HNSC, KIRC, LUAD, LUSC, STAD, and UCEC (Figure 6B). This might prompt that the five gene sets are tightly coordinated or mutually dependent in the general course of tumorigenesis.

On the other hand, we employed the CSPN algorithm to detect significantly denser cross-set gene links, thereby constructing a gene set interconnection network. Differentially coexpressed gene links were identified in individual cancer datasets, and links of the same correlation change direction (strengthened or weakened) were merged across cancer types to form a background network for CSPN to process. Gene networks of strengthened correlations and weakened correlations were analyzed in two separate runs. For the background network of strengthened gene links, we did not retrieve a single pair of gene sets showing increased coordination at *p* < 0.05; for the background network of weakened gene links, we identified 23 significant gene set connections (Figure 6C), which should be interpreted as attenuated crosstalk connections in tumors. It has been repeatedly reported that cancer transcriptomes lost massive gene–gene transcriptional correlations [31,32], but the changing trend of gene set inter-connections has not been studied for cancer datasets yet. We previously carried out the same workflow in chronic kidney disease [24] and schizophrenia [25] datasets, and coincidently found the disease transcriptomes showed exclusively diminishing inter-pathway connections. The current finding of diminished crosstalk among functional gene sets in cancers may represent another instance of regulatory decoherence in abnormal physiology [18].

## 4. Discussion

Generally speaking, neoplastic diseases are regarded as life-threatening malignancies, and a question of critical importance is how long the patient will survive and how good (or bad) the life quality will remain. Greater certainty on what to expect can enhance decision making for many personal and healthcare issues. Single-molecule biomarkers, such as CA 125 for ovarian cancer and PSA for prostate cancer, can be incorporated into routine blood tests through radioimmunoassay or similar traditional lab procedures, but these single-molecule biomarkers suffer from unsatisfactory sensitivity and/or specificity [33]. Multi-gene signatures have been preferentially researched for predicting cancer patients’ prognosis, and a few gene signatures have been put into clinical practice. Many prognosis gene signatures are in the transition from research settings to clinical application; for example, the influential biomedical research portal MSigDB has structurally curated 36 survival-related gene sets from scattered studies in its C2 division, which may be prioritized for imperative clinical investigations. However, immense time elapsed from the conduction of individual prognosis signature research through the structural curation. The present work reported in the manuscript appears to be the first large-scale, pan-cancer prognostic gene set screening, which resulted in comprehensive lists of functionally interpretable gene signatures as candidate prognosis markers for dozens of cancer types. Our results can help accelerate the passage of cancer prognosis signatures from basic research to clinic translation.

Because of the inherent functional interpretability associated with pathways or gene sets, researchers have made efforts to seek prognostic gene sets for cancer patients in the past. An early study in 2011 [34] evaluated 2331 gene sets of two sources for predicting different subtypes of breast cancer. In this study, the prognostic outcome was dichotomized to a binary variable (recurrence vs. no recurrence), differential expression of single genes was examined between the two groups, and the ranked gene-wise *p*-values were assessed for enrichment in a gene set with Gene Set Analysis (GSA) [35], a modified variant of GSEA. Our strategy improved over this approach by reserving the continuous nature of the survival outcome and building a Cox proportional hazard model directly at the gene set level. In another direction, a few groups [36,37] first performed unsupervised summarization of gene set activity (sometimes termed “enrichment score”) and subsequently fitted Cox proportional hazard models on gene sets’ activity scores. Our CGES method has some similarities with this workflow because CGES also seeks to derive a surrogate expression value at the gene set level. Nevertheless, CGES adopts a supervised strategy (single gene survival analysis) to assign variable weights to individual genes within a set; thus theoretically the surrogate score generated by CGES should be more correlated with the survival outcome than the unsupervised activity score. Finally, people also explored combining multiple gene sets in one Cox proportional hazard model to predict patient survival [38,39]. This strategy differs from the above conventional approaches with primary focus shifted from mechanistic interpretation to prediction accuracy. Like the conventional approaches, our CGES-featured workflow chooses to evaluate individual gene sets separately; thus allowing for comparing their prognostic prediction power directly. In addition to the aforesaid technical considerations and improvements, our strategy also advocates the more disease-relevant outcome of DSS, while previous works were all oriented towards overall survival or even its degenerate binary form. Our study covered 33 cancer types and 32,234 gene sets, which represents an unparalleled analysis scope.

By conducting survival analyses at gene set level, we prioritized 18 gene sets that each can predict patient survival in five or more cancer types. Seven of these gene sets showed significant differential coexpression in a pan-cancer meta-analysis, which corroborated their prognostic value. The origins of these seven pan-cancer prognostic gene sets are as follows. MONTERO THYROID CANCER POOR SURVIVAL UP [27] included 12 upregulated genes that could predict poor survival of thyroid carcinoma [27]. FINETTI BREAST CANCER KINOME RED [29] included 16 kinase genes identified from clustering analysis of microarray data, which were found to correlate with poor prognosis of luminal breast tumors. KUMAMOTO RESPONSE TO NUTLIN 3A DN [30] included nine genes that were remarkably decreased in a p53-dependent manner in skin fibroblast cultures post MDM2 inhibition, and these genes were mostly involved in cell cycle and DNA replication [30]. CROSBY E2F4 TARGETS [28] included six putative E2F4 target genes downregulated in the LNCaP C4-2 cells (prostate cancer) at both 6 and 24 h following irradiation; these six genes were therefore presumed to help cells bypass irritation-induced G2 arrest and allow DNA damage to accumulate. In all, these four curated gene sets can be regarded as oncogenic gene signatures that predict poor survival in their respective originating cancers. Our analyses extended their prognostic value from individual cancer types to a pan-cancer scale, greatly expanding their clinical application. Beyond these four curated oncogenic gene signatures, we also presented three functionally defined gene sets: GOBP DOUBLE STRAND BREAK REPAIR VIA BREAK INDUCED REPLICATION, GOCC CONDENSED CHROMOSOME INNER KINETOCHORE, and MODULE 315 (spindle and kinetochore). The centromere, kinetochore, and spindle are essential apparatuses in cell mitosis, whose normal functions and intricate coordination are indispensable for cell proliferation. DNA repair deficiency is a major cause of carcinogenesis. The functions or cellular locations of the latter three gene sets reasonably explained their relevance with prognosis in a wide spectrum of cancer types.

The present study entailed merely computational analyses in multiple cancer datasets from a single consortium. Most of the highlighted prognostic gene signatures concur with the Hallmarks of Cancer, which cannot be regarded as overwhelming discoveries. Furthermore, the enormous results of prognostic gene sets for dozens of cancer types need to be proven in other patient cohorts solidly and robustly. The challenge is that large-scale cancer gene expression data set with survival data is very rare. Thus, conducting exact validation on a gene set in the same cancer type may be challenging. As we observed in the results, some gene sets tended to have prognostic relevance in multiple cancer types. Therefore, an approximate validation may be pursued by examining the same gene set in related, more easily accessible tumor samples. Still, great efforts need to be made to push forward our results to clinical translation, as real clinical advance needs to be instantiated with calibrated, efficacious gene signatures, not just mechanically defined gene functional groups.

## 5. Conclusions

In this study, we conducted the largest scale gene-set-based cancer prognosis study to date. The study followed a novel strategy based on previously developed methodologies and accumulated experience. By implementing several improved approaches compared to previous studies, we demonstrated that gene sets have substantial prognostic value, and should be considered for clinical application. Furthermore, a gene-set-based prognostic study may better illuminate the fundamental biological mechanism compared to the single gene approach.

## Figures and Tables

**Table 1:** Meta differential coexpression analysis results for 18 ubiquitously prognosis-relevant gene sets across 14 TCGA cancer datasets.

Category	Gene Set Name	# Genes	*p*
C2	MONTERO THYROID CANCER POOR SURVIVAL UP	12	0.021
C2	FINETTI BREAST CANCER KINOME RED	16	0.022
C2	KUMAMOTO RESPONSE TO NUTLIN 3A DN	9	0.023
C5	GOBP DOUBLE STRAND BREAK REPAIR VIA BREAK INDUCED REPLICATION	12	0.025
C5	GOCC CONDENSED CHROMOSOME INNER KINETOCHORE	5	0.029
C4	MODULE 315	16	0.031
C2	CROSBY E2F4 TARGETS	6	0.031
C5	GOCC MHC CLASS II PROTEIN COMPLEX	14	0.060
C5	GOCC α DNA POLYMERASE PRIMASE COMPLEX	5	0.061
C2	REACTOME G2 M DNA REPLICATION CHECKPOINT	5	0.063
C2	BIOCARTA TCRA PATHWAY	12	0.070
C7	MATSUMIYA PBMC MODIFIED VACCINIA ANKARA VACCINE AGE 4 6MO BCG PRIMED 28DY UP	9	0.091
C5	GOCC NUCLEAR INCLUSION BODY	11	0.117
C5	GOCC EUKARYOTIC TRANSLATION INITIATION FACTOR 2 COMPLEX	4	0.132
C5	GOMF MHC CLASS II RECEPTOR ACTIVITY	9	0.158
C5	GOCC ALPHA BETA T CELL RECEPTOR COMPLEX	7	0.161
C5	GOMF D LOOP DNA BINDING	5	0.182
C7	ANDERSON BLOOD CN54GP140 ADJUVANTED WITH GLA AF AGE 18 45YO HIGH IGM RESPONDERS 3DY 7D DN	6	0.254

## Data Availability

All data used in this study were obtained from public repositories.

## Supplementary Materials

The following supporting information can be downloaded at: https://www.mdpi.com/article/10.3390/genes13050862/s1, Table S1: Gene composition before and after single-gene survival analysis, respectively; Table S2: MSigDB gene sets showing prognostic significance in 33 cancer types; Figure S1: Gene–gene correlation heatmap in three exemplar cancer types.
