# Supplementary material for: A Novel Strategy to Identify Prognosis-Relevant Gene Sets in Cancers
Source: Genes (Basel). 2022 May 12;13(5):862. doi: 10.3390/genes13050862 (PMC9141699; doi:10.3390/genes13050862)
Supplement: Supplementary file 1 [file genes-13-00862-s001.zip › genes-1694031-supplementary.pdf]

A Novel Strategy to Identify Prognosis-Relevant Gene Sets in Cancers

Junyi Pu [1], Hui Yu [2] and Yan Guo [2*], yaguo@salud.unmn.edu

Figure S1. Gene–gene correlation heatmap in three exemplar cancer types. In each heatmap, the lower and upper triangles represent the normal state and the tumor state, respectively. For each gene set, two cancer types showing discernable differential coexpression (positive cases) and one cancer type showing illegible differential coexpression (negative control) are displayed, which are divided with a red vertical line.

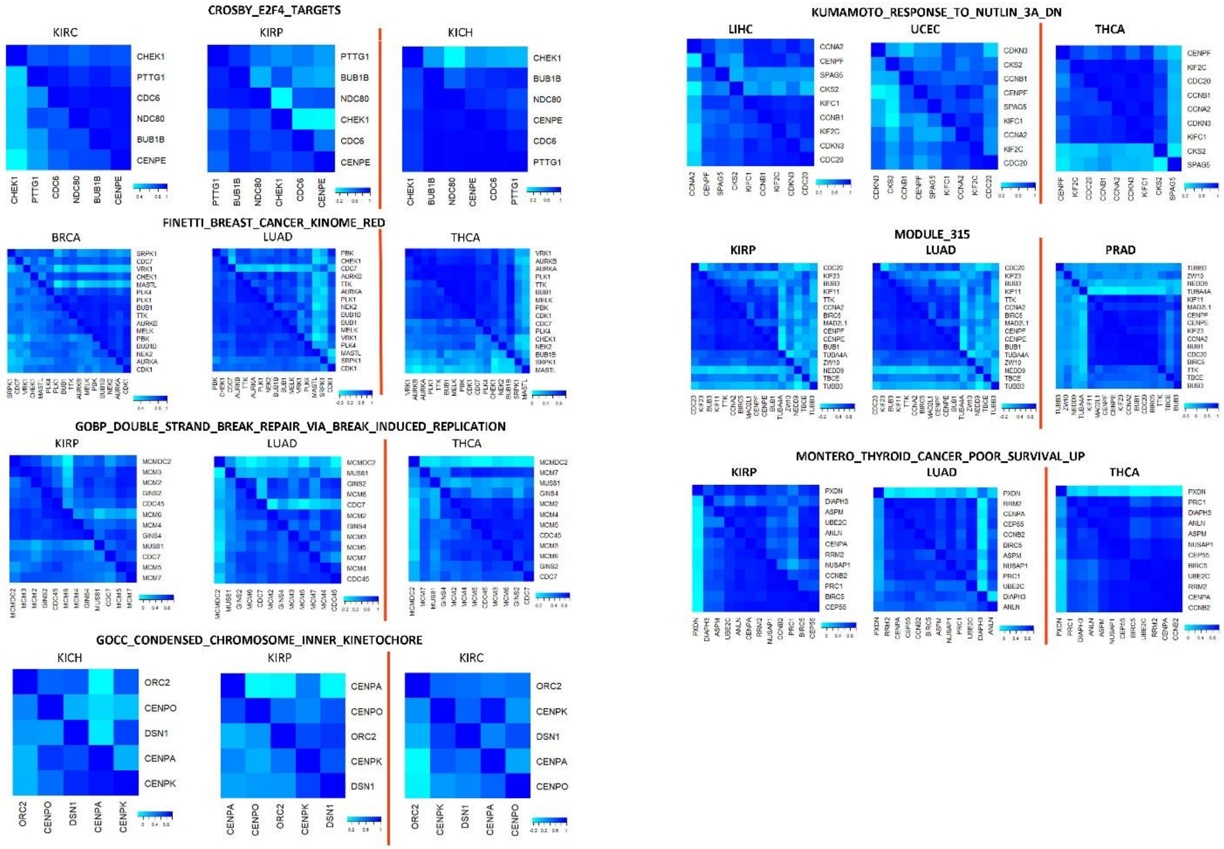

Supplementary Table S1. Gene composition before and after single-gene survival analysis, respectively.

Genes/Associations	All genes involved in transcriptome (original composition)		Prognosis-significant genes (post single-gene survival analysis)	
	Count	Proportion	Count	Proportion
lncRNA	14032	0.232	51145	0.230
other	12069	0.200	24852	0.112
protein coding	19559	0.323	107089	0.482
pseudogene	14823	0.245	38929	0.175

Supplementary Table S2. MSigDB gene sets showing prognostic significance in 33 cancer types.

**MSigDB division**	**Gene set name**	**Cancer types**	**# Cancer types**
c2: curated gene sets	KUMAMOTO RESPONSE TO NUTLIN 3A DN	ACC, KIRC, KIRP, LGG, LIHC, LUAD, MESO, PRAD, SKCM, UCEC	10
c2: curated gene sets	CROSBY E2F4 TARGETS	ACC, COAD, KIRC, KIRP, LGG, LIHC, LUAD, MESO, PAAD	9
c5: Ontology gene sets	GOCC ALPHA BETA T CELL RECEPTOR COMPLEX	BLCA, CESC, HNSC, LUAD, SKCM, UCEC, UVM	7
c2: curated gene sets	FINETTI BREAST CANCER KINOME RED	ACC, KIRC, KIRP, LIHC, LUAD, MESO, SARC	7
c2: curated gene sets	MONTERO THYROID CANCER POOR SURVIVAL UP	ACC, KIRC, KIRP, LUAD, MESO, PAAD	6
c5: Ontology gene sets	GOCC EUKARYOTIC TRANSLATION INITIATION FACTOR 2 COMPLEX	HNSC, KICH, KIRP, LIHC, PAAD, UVM	6
c2: curated gene sets	REACTOME G2 M DNA REPLICATION CHECKPOINT	ACC, LGG, LIHC, MESO, SKCM	5
c4: computational gene sets	MODULE 315	KICH, KIRC, KIRP, LUAD, MESO	5

c7: immunologic signature gene sets	MATSUMIYA PBMC MODIFIED VACCINIA ANKARA VACCINE AGE 4 6MO BCG PRIMED 28DY UP	KIRC, OV, SKCM, THCA, UVM	5
c5: Ontology gene sets	GOMF MHC CLASS II RECEPTOR ACTIVITY	BRCA, CESC, LUAD, SKCM, UVM	5
c5: Ontology gene sets	GOMF D LOOP DNA BINDING	KICH, LGG, LIHC, MESO, THYM	5
c5: Ontology gene sets	GOCC NUCLEAR INCLUSION BODY	KICH, KIRC, LIHC, PRAD, THYM	5
c5: Ontology gene sets	GOCC MHC CLASS II PROTEIN COMPLEX	BRCA, CESC, LUAD, SKCM, UVM	5
c5: Ontology gene sets	GOCC CONDENSED CHROMOSOME INNER KINETOCHORE	ACC, KICH, KIRP, LGG, LIHC	5
c5: Ontology gene sets	GOCC ALPHA DNA POLYMERASE PRIMASE COMPLEX	KICH, KIRP, LGG, LIHC, OV	5
c5: Ontology gene sets	GOBP DOUBLE STRAND BREAK REPAIR VIA BREAK INDUCED REPLICATION	ACC, CESC, KIRP, LIHC, LUAD	5
c2: curated gene sets	BIOCARTA TCRA PATHWAY	CHOL, LGG, SKCM, UCEC, UVM	5
c7: immunologic signature gene sets	ANDERSON BLOOD CN54GP140 ADJUVANTED WITH GLA AF AGE 18 45YO HIGH IGM RESPONDERS 3DY 7D DN	BLCA, BRCA, CESC, SKCM, THYM	5
c2: curated gene sets	REACTOME UNWINDING OF DNA	KIRP, LIHC, LUAD, MESO	4
c2: curated gene sets	REACTOME PHOSPHORYLATION OF EMI1	ACC, MESO, UCEC, UVM	4
c2: curated gene sets	REACTOME G2 PHASE	KIRC, KIRP, LIHC, PAAD	4
c7: immunologic signature gene sets	QI NAIVE T CELL ZOSTAVAX AGE 52 75YO CD4 T CELL VS NAIVE CD4 T CELL 7 TO 9DY UP	CESC, SKCM, THYM, UVM	4
c4: computational gene sets	MODULE 90	KIRC, LGG, LIHC, SKCM	4
c4: computational gene sets	MODULE 293	LGG, THCA, THYM, UVM	4

c4: computational gene sets	MODULE 143	OV, THCA, THYM, UVM	4
c2: curated gene sets	KALMA E2F1 TARGETS	ACC, KIRC, LIHC, MESO	4
c5: Ontology gene sets	HP MOLLUSCOID PSEUDOTUMORS	ACC, KIRC, KIRP, MESO	4
c5: Ontology gene sets	HP DEEP SET NAILS	ACC, KICH, KIRC, MESO	4
c5: Ontology gene sets	HP DECREASED PROPORTION OF CD8 POSITIVE T CELLS	BRCA, LGG, SKCM, UVM	4
c5: Ontology gene sets	HP ARTERIAL RUPTURE	ACC, KIRC, LGG, MESO	4
c5: Ontology gene sets	GOMF RNA DNA HYBRID RIBONUCLEASE ACTIVITY	ACC, KIRP, LIHC, MESO	4
c5: Ontology gene sets	GOMF MUTLALPHA COMPLEX BINDING	KICH, LGG, LIHC, SARC	4
c5: Ontology gene sets	GOMF KINETOCHORE BINDING	ACC, KIRC, KIRP, MESO	4
c5: Ontology gene sets	GOCC NUCLEAR PORE OUTER RING	ACC, KICH, LIHC, THYM	4
c5: Ontology gene sets	GOCC MHC CLASS I PROTEIN COMPLEX	LGG, OV, THCA, UVM	4
c5: Ontology gene sets	GOCC HISTONE PRE MRNA 3 END PROCESSING COMPLEX	KIRP, LGG, LIHC, MESO	4
c5: Ontology gene sets	GOCC ENDOLYSOSOME LUMEN	GBM, LGG, THCA, UVM	4
c5: Ontology gene sets	GOCC DNA REPLICATION PREINITIATION COMPLEX	KIRP, LIHC, LUAD, MESO	4
c5: Ontology gene sets	GOCC CONDENSIN COMPLEX	ACC, LGG, LIHC, LUAD	4
c5: Ontology gene sets	GOCC CMG COMPLEX	KIRP, LIHC, LUAD, MESO	4
c5: Ontology gene sets	GOBP SMOOTHENED SIGNALING PATHWAY INVOLVED IN REGULATION OF CEREBELLAR GRANULE CELL PRECURSOR CELL PROLIFERATION	ACC, KIRC, LGG, STAD	4
c5: Ontology gene sets	GOBP PRE-REPLICATIVE COMPLEX ASSEMBLY INVOLVED IN CELL CYCLE DNA REPLICATION	KICH, KIRP, LIHC, LUAD	4

c5: Ontology gene sets	GOBP POSITIVE REGULATION OF PROTEIN LOCALIZATION TO CILIUM	ACC, KICH, PAAD, UCEC	4
c5: Ontology gene sets	GOBP HINDGUT DEVELOPMENT	ACC, BLCA, KIRP, LGG	4
c5: Ontology gene sets	GOBP DNA REPLICATION DEPENDENT NUCLEOSOME ORGANIZATION	ACC, CESC, LGG, LIHC	4
c5: Ontology gene sets	GOBP CELL CYCLE DNA REPLICATION INITIATION	KICH, KIRP, LIHC, UCEC	4
c2: curated gene sets	EGUCHI CELL CYCLE RB1 TARGETS	ACC, KIRC, LIHC, LUAD	4
c2: curated gene sets	BIOCARTA TCAPOPTOSIS PATHWAY	CESC, LGG, SKCM, UVM	4
c2: curated gene sets	BIOCARTA RANMS PATHWAY	KICH, KIRC, KIRP, LIHC	4
c2: curated gene sets	BIOCARTA CTL PATHWAY	CESC, SKCM, UCEC, UVM	4
c2: curated gene sets	BIOCARTA BBCELL PATHWAY	CESC, LGG, SKCM, UVM	4
c3: regulatory target gene sets	ZNF774 TARGET GENES	GBM, KIRC, UVM	3
c2: curated gene sets	ZERBINI RESPONSE TO SULINDAC DN	KIRC, PAAD, UVM	3
c2: curated gene sets	WP MACROPHAGE MARKERS	KIRC, LGG, SKCM	3
c2: curated gene sets	WANG THOC1 TARGETS UP	BRCA, LGG, LUAD	3
c2: curated gene sets	STONER ESOPHAGEAL CARCINOGENESIS DN	HNSC, LIHC, THYM	3
c2: curated gene sets	SCIAN CELL CYCLE TARGETS OF TP53 AND TP73 DN	ACC, KIRC, LUAD	3
c7: immunologic signature gene sets	RICHERT PBMC HIV LIPO 5 AGE 37 48YO STIMULATED VS UNSTIMULATED 14W INTERFERON SUBSET UP	LGG, SKCM, THCA	3
c2: curated gene sets	REICHERT G1S REGULATORS AS PI3K TARGETS	KIRC, KIRP, SKCM	3

c2: curated gene sets	REACTOME TFAP2A ACTS AS A TRANSCRIPTIONAL REPRESSOR DURING RETINOIC ACID INDUCED CELL DIFFERENTIATION	KIRP, LUAD, MESO	3
c2: curated gene sets	REACTOME NEF AND SIGNAL TRANSDUCTION	LGG, SKCM, THYM	3
c2: curated gene sets	REACTOME FLT3 SIGNALING THROUGH SRC FAMILY KINASES	HNSC, KIRC, UVM	3
c2: curated gene sets	REACTOME E2F ENABLED INHIBITION OF PRE-REPLICATION COMPLEX FORMATION	KICH, KIRC, LIHC	3
c2: curated gene sets	REACTOME DNA REPLICATION INITIATION	CESC, KICH, LIHC	3
c2: curated gene sets	REACTOME DNA METHYLATION	LGG, MESO, SARC	3
c2: curated gene sets	REACTOME CDC6 ASSOCIATION WITH THE ORC ORIGIN COMPLEX	KICH, KIRC, LIHC	3
c7: immunologic signature gene sets	QI NAIVE T CELL ZOSTAVAX AGE 52 75YO CD4 T CELL VS NAIVE CD4 T CELL 7 TO 9DY DN	CESC, SKCM, THYM	3
c7: immunologic signature gene sets	QI CD4 POSITIVE ALPHA BETA MEMORY T CELL ZOSTAVAX AGE 52 75YO CD4 T CELL VS NAIVE CD4 T CELL 7 TO 9DY DN	CESC, SKCM, THYM	3
c2: curated gene sets	OHASHI AURKB TARGETS	KIRP, LIHC, SKCM	3
c2: curated gene sets	OHASHI AURKA TARGETS	ACC, LGG, UCEC	3
c2: curated gene sets	MYLLYKANGAS AMPLIFICATION HOT SPOT 21	KIRC, LGG, MESO	3
c4: computational gene sets	MODULE 534	KIRC, LGG, PAAD	3

c7: immunologic signature gene sets	MATSUMIYA PBMC MODIFIED VACCINIA ANKARA VACCINE AGE 18 55YO HIGH RESPONDERS VS LOW RESPONDERS 0DY UP	CESC, LUAD, UVM	3
c2: curated gene sets	LY AGING MIDDLE DN	ACC, KIRC, UVM	3
c2: curated gene sets	LOPEZ MESOTELIOMA SURVIVAL TIME UP	LGG, LIHC, MESO	3
c2: curated gene sets	IIZUKA LIVER CANCER PROGRESSION L1 G1 DN	KIRC, LIHC, MESO	3
c5: Ontology gene sets	HP RECURRENT PHARYNGITIS	KIRC, SKCM, UVM	3
c5: Ontology gene sets	HP PREMATURE RUPTURE OF MEMBRANES	ACC, BRCA, LGG	3
c5: Ontology gene sets	HP PERSISTENT EBV VIREMIA	LUAD, OV, THYM	3
c5: Ontology gene sets	HP NARROW ILIAC WINGS	ACC, BRCA, COAD	3
c5: Ontology gene sets	HP MICROTIA THIRD DEGREE	ACC, KICH, LIHC	3
c5: Ontology gene sets	HP INCREASED LAXITY OF FINGERS	KIRC, KIRP, MESO	3
c5: Ontology gene sets	HP INCREASED LAXITY OF ANKLES	BRCA, KIRC, KIRP	3
c5: Ontology gene sets	HP IMPAIRED OXIDATIVE BURST	LGG, SKCM, UVM	3
c5: Ontology gene sets	HP HERPES SIMPLEX ENCEPHALITIS	BRCA, LGG, LUAD	3
c5: Ontology gene sets	HP GEOGRAPHIC ATROPHY	LGG, STAD, UCS	3
c5: Ontology gene sets	HP DIAPHRAGMATIC PARALYSIS	COAD, LUAD, UVM	3
c5: Ontology gene sets	HP ABNORMALITY OF THE TONGUE MUSCLE	KIRC, MESO, PAAD	3
c5: Ontology gene sets	GOMF Y FORM DNA BINDING	LGG, LIHC, MESO	3
c5: Ontology gene sets	GOMF UBIQUITIN PROTEIN TRANSFERASE INHIBITOR ACTIVITY	KICH, KIRP, LIHC	3
c5: Ontology gene sets	GOMF UBIQUITIN LIGASE INHIBITOR ACTIVITY	KICH, KIRP, LIHC	3
c5: Ontology gene sets	GOMF TAT PROTEIN BINDING	HNSC, LIHC, MESO	3

c5: Ontology gene sets	GOMF IMMUNOGLOBULIN RECEPTOR ACTIVITY	CESC, LUAD, SKCM	3
c5: Ontology gene sets	GOMF HISTONE METHYLTRANSFERASE ACTIVITY H3 K27 SPECIFIC	ACC, LIHC, SKCM	3
c5: Ontology gene sets	GOMF GLUCURONOSYL N ACETYLGALACTOSAMINYL PROTEOGLYCAN 4 BETA N ACETYLGALACTOSAMINYLTRANSFERASE ACTIVITY	BLCA, BRCA, READ	3
c5: Ontology gene sets	GOMF ER RETENTION SEQUENCE BINDING	KIRC, LGG, MESO	3
c5: Ontology gene sets	GOMF DOLICHYL DIPHOSPHOOLIGOSACCHARIDE PROTEIN GLYCOTRANSFERASE ACTIVITY	KICH, LGG, LIHC	3
c5: Ontology gene sets	GOMF DNA INSERTION OR DELETION BINDING	KICH, READ, UCEC	3
c5: Ontology gene sets	GOMF CCR1 CHEMOKINE RECEPTOR BINDING	KIRC, SKCM, THYM	3
c5: Ontology gene sets	GOMF CARBON NITROGEN LIGASE ACTIVITY WITH GLUTAMINE AS AMIDO N DONOR	LIHC, MESO, UCEC	3
c5: Ontology gene sets	GOMF 7S RNA BINDING	COAD, KICH, KIRP	3
c5: Ontology gene sets	GOCC SEC61 TRANSLOCON COMPLEX	KIRC, KIRP, UCEC	3
c5: Ontology gene sets	GOCC PWP2P CONTAINING SUBCOMPLEX OF 90S PRERIBOSOME	KIRP, LGG, OV	3
c5: Ontology gene sets	GOCC PROTEASOME REGULATORY PARTICLE LID SUBCOMPLEX	BRCA, KIRP, LIHC	3
c5: Ontology gene sets	GOCC PRONUCLEUS	ACC, LIHC, MESO	3
c5: Ontology gene sets	GOCC PRESPLICEOSOME	KIRC, LIHC, READ	3
c5: Ontology gene sets	GOCC MICROTUBULE MINUS END	KIRC, LGG, THYM	3
c5: Ontology gene sets	GOCC MCM COMPLEX	KICH, KIRP, LGG	3

c5: Ontology gene sets	GOCC INTEGRAL COMPONENT OF CYTOPLASMIC SIDE OF ENDOPLASMIC RETICULUM MEMBRANE	KIRC, KIRP, LGG	3
c5: Ontology gene sets	GOCC GLYCOGEN GRANULE	BRCA, LGG, THCA	3
c5: Ontology gene sets	GOCC DNA RECOMBINASE MEDIATOR COMPLEX	ACC, KICH, PAAD	3
c5: Ontology gene sets	GOCC CTF18 RFC LIKE COMPLEX	CESC, LIHC, MESO	3
c5: Ontology gene sets	GOCC CRD MEDIATED MRNA STABILITY COMPLEX	KICH, KIRC, LIHC	3
c5: Ontology gene sets	GOCC CONDENSED CHROMOSOME OUTER KINETOCHORE	KICH, KIRC, LUAD	3
c5: Ontology gene sets	GOCC COMMITMENT COMPLEX	ACC, OV, SARC	3
c5: Ontology gene sets	GOCC CHROMOSOME PASSENGER COMPLEX	KIRP, LGG, SKCM	3
c5: Ontology gene sets	GOBP THYMOCYTE MIGRATION	CESC, KIRC, SKCM	3
c5: Ontology gene sets	GOBP SUCCINATE METABOLIC PROCESS	BRCA, KIRC, PRAD	3
c5: Ontology gene sets	GOBP SCARNA LOCALIZATION TO CAJAL BODY	KICH, KIRP, LIHC	3
c5: Ontology gene sets	GOBP RESPONSE TO INDOLE 3 METHANOL	KICH, KIRC, LGG	3
c5: Ontology gene sets	GOBP REGULATION OF HYPERSENSITIVITY	KIRC, LUAD, SKCM	3
c5: Ontology gene sets	GOBP REGULATION OF DNA DIRECTED DNA POLYMERASE ACTIVITY	KIRP, LIHC, MESO	3
c5: Ontology gene sets	GOBP REGULATION OF CD4 POSITIVE CD25 POSITIVE ALPHA BETA REGULATORY T CELL DIFFERENTIATION	CESC, SKCM, UVM	3
c5: Ontology gene sets	GOBP PULMONARY ARTERY MORPHOGENESIS	KIRC, PRAD, THCA	3

c5: Ontology gene sets	GOBP POST EMBRYONIC EYE MORPHOGENESIS	ACC, LGG, UCEC	3
c5: Ontology gene sets	GOBP POSITIVE REGULATION OF NUCLEOBASE CONTAINING COMPOUND TRANSPORT	KICH, KIRP, LIHC	3
c5: Ontology gene sets	GOBP POSITIVE REGULATION OF NATURAL KILLER CELL CHEMOTAXIS	DLBC, KIRC, LGG	3
c5: Ontology gene sets	GOBP POSITIVE REGULATION OF ISOTYPE SWITCHING TO IGA ISOTYPES	KIRP, LGG, MESO	3
c5: Ontology gene sets	GOBP POSITIVE REGULATION OF ESTABLISHMENT OF PROTEIN LOCALIZATION TO TELOMERE	ACC, KIRP, LIHC	3
c5: Ontology gene sets	GOBP POLYPRENOL METABOLIC PROCESS	LIHC, SKCM, THYM	3
c5: Ontology gene sets	GOBP NEGATIVE REGULATION OF INTERFERON ALPHA PRODUCTION	CESC, LUAD, SKCM	3
c5: Ontology gene sets	GOBP NEGATIVE REGULATION OF CERAMIDE BIOSYNTHETIC PROCESS	DLBC, KIRC, UVM	3
c5: Ontology gene sets	GOBP NECROPTOTIC SIGNALING PATHWAY	LGG, SARC, SKCM	3
c5: Ontology gene sets	GOBP MEIOTIC SPINDLE ORGANIZATION	ACC, KIRC, LUAD	3
c5: Ontology gene sets	GOBP HYPOTONIC RESPONSE	COAD, KIRC, KIRP	3
c5: Ontology gene sets	GOBP G QUADRUPLEX DNA UNWINDING	KIRC, LIHC, SARC	3
c5: Ontology gene sets	GOBP FEMALE MEIOSIS CHROMOSOME SEGREGATION	ACC, LIHC, MESO	3

c5: Ontology gene sets	GOBP ESTABLISHMENT OF VIRAL LATENCY	KIRC, LIHC, PCPG	3
c5: Ontology gene sets	GOBP DNA METHYLATION ON CYTOSINE	LGG, MESO, SARC	3
c5: Ontology gene sets	GOBP DNA ENDOREDUPLICATION	LUAD, MESO, UVM	3
c5: Ontology gene sets	GOBP CRD MEDIATED MRNA STABILIZATION	ACC, KICH, SARC	3
c5: Ontology gene sets	GOBP CEREBELLAR PURKINJE CELL GRANULE CELL PRECURSOR CELL SIGNALING INVOLVED IN REGULATION OF GRANULE CELL PRECURSOR CELL PROLIFERATION	ACC, KIRC, LGG	3
c5: Ontology gene sets	GOBP ASSEMBLY OF ACTOMYOSIN APPARATUS INVOLVED IN CYTOKINESIS	KIRC, KIRP, LIHC	3
c4: computational gene sets	GNF2 ZAP70	SKCM, THYM, UVM	3
c4: computational gene sets	GNF2 MKI67	LIHC, LUAD, MESO	3
c7: immunologic signature gene sets	CAO BLOOD FLUZONE AGE 05 14YO CORRELATED WITH H3N1 HI TITER 1DY POSITIVE	OV, SKCM, UCEC	3
c7: immunologic signature gene sets	CAO BLOOD FLUMIST AGE 05 14YO CORRELATED WITH H3N2 VN TITER 7DY POSITIVE	OV, SKCM, THCA	3
c2: curated gene sets	BIOCARTA THELPER PATHWAY	LGG, UCEC, UVM	3
c2: curated gene sets	BIOCARTA TCYTOTOXIC PATHWAY	BLCA, LGG, UVM	3
c2: curated gene sets	BIOCARTA NO2IL12 PATHWAY	CESC, THYM, UVM	3
c3: regulatory target gene sets	ZSCAN26 TARGET GENES	KIRC, UVM	2
c3: regulatory target gene sets	ZNF20 TARGET GENES	KICH, UCEC	2
c3: regulatory target gene sets	ZNF136 TARGET GENES	KIRC, THYM	2

c2: curated gene sets	ZHU SKIL TARGETS DN	KIRC, THYM	2
c2: curated gene sets	WP PILOCYTIC ASTROCYTOMA	KICH, THYM	2
c2: curated gene sets	WP PENTOSE PHOSPHATE METABOLISM	ACC, KIRC	2
c2: curated gene sets	WP KETOGENESIS AND KETOLYSIS	KIRP, PAAD	2
c2: curated gene sets	WP FBXL10 ENHANCEMENT OF MAPERK SIGNALING IN DIFFUSE LARGE BCELL LYMPHOMA	KIRC, LIHC	2
c2: curated gene sets	WP EV RELEASE FROM CARDIAC CELLS AND THEIR FUNCTIONAL EFFECTS	COAD, THYM	2
c2: curated gene sets	WP DUAL HIJACK MODEL OF VIF IN HIV INFECTION	KIRC, UVM	2
c2: curated gene sets	WP DDX1 AS A REGULATORY COMPONENT OF THE DROSHA MICROPROCESSOR	READ, SKCM	2
c2: curated gene sets	WP BIOGENIC AMINE SYNTHESIS	DLBC, READ	2
c2: curated gene sets	WILENSKY RESPONSE TO DARAPLADIB	DLBC, SKCM	2
c7: immunologic signature gene sets	WEINBERGER BLOOD TWINRIX AGE 20 40 AND 60 84YO CORRELATED WITH HIGH ANTI HBS CONC AT WEEK 4 POST BOOSTER VACC 1DY POSITIVE	CESC, SKCM	2
c7: immunologic signature gene sets	WEINBERGER BLOOD TWINRIX AGE 20 40 AND 60 84YO CORRELATED WITH HIGH ANTI HBS CONC AT WEEK 30 PRIMARY VACC 1DY POSITIVE	LGG, SKCM	2
c2: curated gene sets	WATANABE ULCERATIVE COLITIS WITH CANCER DN	KIRC, UVM	2
c2: curated gene sets	TURJANSKI MAPK7 TARGETS	KIRP, THYM	2

c2: curated gene sets	TURJANSKI MAPK11 TARGETS	KIRC, THYM	2
c2: curated gene sets	TSAI DNAJB4 TARGETS DN	LGG, UVM	2
c3: regulatory target gene sets	TCGATGG MIR213	MESO, THYM	2
c2: curated gene sets	SMID BREAST CANCER LUMINAL A DN	KIRC, LUAD	2
c2: curated gene sets	SHARMA PILOCYTIC ASTROCYTOMA LOCATION DN	LGG, MESO	2
c2: curated gene sets	SALVADOR MARTIN PEDIATRIC TBD ANTI TNF THERAPY NONRESPONDER POST TREATMENT DN	HNSC, SKCM	2
c2: curated gene sets	ROETH TERT TARGETS UP	PCPG, UCEC	2
c2: curated gene sets	REACTOME ZINC TRANSPORTERS	COAD, READ	2
c2: curated gene sets	REACTOME TYSND1 CLEAVES PEROXISOMAL PROTEINS	KIRC, READ	2
c2: curated gene sets	REACTOME TLR3 MEDIATED TICAM1 DEPENDENT PROGRAMMED CELL DEATH	LGG, SARC	2
c2: curated gene sets	REACTOME SYNTHESIS OF PIPS AT THE ER MEMBRANE	SKCM, THYM	2
c2: curated gene sets	REACTOME SUMO IS CONJUGATED TO E1 UBA2 SAE1	ACC, LIHC	2
c2: curated gene sets	REACTOME RUNX3 REGULATES IMMUNE RESPONSE AND CELL MIGRATION	LGG, THYM	2
c2: curated gene sets	REACTOME RUNX1 REGULATES TRANSCRIPTION OF GENES INVOLVED IN DIFFERENTIATION OF KERATINOCYTES	GBM, UVM	2
c2: curated gene sets	REACTOME RRNA MODIFICATION IN THE MITOCHONDRION	DLBC, LGG	2

c2: curated gene sets	REACTOME RELEASE OF APOPTOTIC FACTORS FROM THE MITOCHONDRIA	LGG, UCEC	2
c2: curated gene sets	REACTOME PURINE RIBONUCLEOSIDE MONOPHOSPHATE BIOSYNTHESIS	ACC, KIRP	2
c2: curated gene sets	REACTOME PRC2 METHYLATES HISTONES AND DNA	LGG, LIHC	2
c2: curated gene sets	REACTOME POLB DEPENDENT LONG PATCH BASE EXCISION REPAIR	ACC, LGG	2
c2: curated gene sets	REACTOME PD 1 SIGNALING	CESC, UVM	2
c2: curated gene sets	REACTOME NEF MEDIATED CD4 DOWNREGULATION	DLBC, KIRC	2
c2: curated gene sets	REACTOME N GLYCAN TRIMMING AND ELONGATION IN THE CIS GOLGI	COAD, LGG	2
c2: curated gene sets	REACTOME MASTL FACILITATES MITOTIC PROGRESSION	LIHC, LUAD	2
c2: curated gene sets	REACTOME INTERLEUKIN 9 SIGNALING	BRCA, LUAD	2
c2: curated gene sets	REACTOME INTERLEUKIN 23 SIGNALING	BLCA, CESC	2
c2: curated gene sets	REACTOME INTERLEUKIN 2 SIGNALING	BRCA, KIRC	2
c2: curated gene sets	REACTOME INTERLEUKIN 18 SIGNALING	BRCA, SKCM	2
c2: curated gene sets	REACTOME INTERLEUKIN 15 SIGNALING	KIRC, SKCM	2
c2: curated gene sets	REACTOME FLT3 SIGNALING BY CBL MUTANTS	SARC, UVM	2

c2: curated gene sets	REACTOME FICOLINS BIND TO REPETITIVE CARBOHYDRATE STRUCTURES ON THE TARGET CELL SURFACE	HNSC, STAD	2
c2: curated gene sets	REACTOME FASL CD95L SIGNALING	LGG, SKCM	2
c2: curated gene sets	REACTOME ERCC6 CSB AND EHMT2 G9A POSITIVELY REGULATE RRNA EXPRESSION	ACC, THYM	2
c2: curated gene sets	REACTOME DISEASES OF MISMATCH REPAIR MMR	KICH, READ	2
c2: curated gene sets	REACTOME DEFECTIVE RIPK1 MEDIATED REGULATED NECROSIS	LGG, MESO	2
c2: curated gene sets	REACTOME CROSS PRESENTATION OF PARTICULATE EXOGENOUS ANTIGENS PHAGOSOMES	COAD, LGG	2
c2: curated gene sets	REACTOME CREB PHOSPHORYLATION	COAD, KIRP	2
c2: curated gene sets	REACTOME CONSTITUTIVE SIGNALING BY EGFRVIII	KIRC, LIHC	2
c2: curated gene sets	REACTOME CONDENSATION OF PROMETAPHASE CHROMOSOMES	LIHC, LUAD	2
c2: curated gene sets	REACTOME BETA OXIDATION OF LAUROYL COA TO DECANOYL COA COA	KIRP, SKCM	2
c2: curated gene sets	REACTOME BETA OXIDATION OF HEXANOYL COA TO BUTANOYL COA	KIRP, SKCM	2
c2: curated gene sets	REACTOME ATF6 ATF6 ALPHA ACTIVATES CHAPERONES	KIRP, LUSC	2

c2: curated gene sets	REACTOME ASSEMBLY OF THE ORC COMPLEX AT THE ORIGIN OF REPLICATION	KICH, LIHC	2
c2: curated gene sets	REACTOME ARMS MEDIATED ACTIVATION	KIRC, LUAD	2
c2: curated gene sets	REACTOME ALPHA DEFENSINS	GBM, HNSC	2
c2: curated gene sets	REACTOME ACTIVATION OF NIMA KINASES NEK9 NEK6 NEK7	KIRC, LIHC	2
c2: curated gene sets	REACTOME ACTIVATION OF C3 AND C5	LGG, SKCM	2
c2: curated gene sets	PID TCR JNK PATHWAY	LGG, THYM	2
c2: curated gene sets	PID NFKAPPAB CANONICAL PATHWAY	READ, THYM	2
c2: curated gene sets	PETRETTO LEFT VENTRICLE MASS QTL CIS DN	KIRC, THYM	2
c2: curated gene sets	PEDERSEN METASTASIS BY ERBB2 ISOFORM 5	KIRC, LUAD	2
c7: immunologic signature gene sets	PANAPASA BLOOD FLUENZ AGE 03 17YO 6DY 7DY UP	CESC, LGG	2
c7: immunologic signature gene sets	OVSYANNIKOVA PBMC FLUARIX AGE 55 64YO RESPONDERS VS NONRESPONDERS 0DY DN	COAD, KIRC	2
c2: curated gene sets	OHM EMBRYONIC CARCINOMA DN	BLCA, KIRC	2
c2: curated gene sets	NGO MALIGNANT GLIOMA 1P LOH	KIRC, LIHC	2
c7: immunologic signature gene sets	NAKAYA PBMC FLUAD IMUVAC MALE AGE 14 27YO CORRELATED WITH HAI RESPONSE MF59 ADJUVANTED AND NON 1DY GENES IN BTM M75 POSITIVE	PCPG, SKCM	2
c2: curated gene sets	NADELLA PRKAR1A TARGETS UP	COAD, KIRC	2

c2: curated gene sets	MYLLYKANGAS AMPLIFICATION HOT SPOT 2	KIRC, PRAD	2
c2: curated gene sets	MOSERLE IFNA RESPONSE	SKCM, THCA	2
c4: computational gene sets	MODULE 362	PCPG, READ	2
c3: regulatory target gene sets	MIR6082	DLBC, THYM	2
c3: regulatory target gene sets	MIR4787 5P	COAD, KIRC	2
c3: regulatory target gene sets	MIR4537	LGG, PAAD	2
c2: curated gene sets	MIKHAYLOVA OXIDATIVE STRESS RESPONSE VIA VHL UP	ACC, KIRC	2
c7: immunologic signature gene sets	MATSUMIYA PBMC MODIFIED VACCINIA ANKARA VACCINE AGE 4 6MO VACCINATED VS CANDIN PLACEBO BCG PRIMED 28DY UP	OV, SKCM	2
c7: immunologic signature gene sets	MATSUMIYA BLOOD MODIFIED VACCINIA ANKARA VACCINE AGE 4 6MO VACCINATED VS CANDIN PLACEBO BCG PRIMED 1DY DN	KIRC, LUSC	2
c2: curated gene sets	LUI THYROID CANCER CLUSTER 4	LUAD, SKCM	2
c2: curated gene sets	LOPEZ MESOTHELIOMA SURVIVAL WORST VS BEST DN	BRCA, LGG	2
c2: curated gene sets	LIANG SILENCED BY METHYLATION DN	KIRP, LUAD	2
c7: immunologic signature gene sets	LI PBMC MENACTRA AGE 18 45YO 7DY UP	HNSC, UCEC	2
c2: curated gene sets	KUROZUMI RESPONSE TO ONCOCYTIC VIRUS AND CYCLIC RGD	SKCM, UVM	2
c2: curated gene sets	KUMAMOTO RESPONSE TO NUTLIN 3A UP	KIRC, LUAD	2

c2: curated gene sets	KEGG PRIMARY IMMUNODEFICIENCY	CESC, DLBC	2
c2: curated gene sets	KEGG LIMONENE AND PINENE DEGRADATION	KIRC, LGG	2
c2: curated gene sets	KAUFFMANN MELANOMA RELAPSE DN	LGG, UVM	2
c2: curated gene sets	KASLER HDAC7 TARGETS 2 UP	CESC, THYM	2
c2: curated gene sets	KAPOSI LIVER CANCER MET UP	COAD, MESO	2
c2: curated gene sets	KAPOSI LIVER CANCER MET DN	LGG, MESO	2
c2: curated gene sets	JONES TCOF1 TARGETS	KIRC, LUAD	2
c2: curated gene sets	INGA TP53 TARGETS	BRCA, KIRC	2
c5: Ontology gene sets	HP UTERINE PROLAPSE	ACC, KIRP	2
c5: Ontology gene sets	HP URETHROVAGINAL FISTULA	CESC, KIRC	2
c5: Ontology gene sets	HP UPPER LIMB ASYMMETRY	KIRC, LGG	2
c5: Ontology gene sets	HP UNILATERAL CLEFT LIP	KIRP, LIHC	2
c5: Ontology gene sets	HP TYPE II LISSENCEPHALY	KIRC, MESO	2
c5: Ontology gene sets	HP TIGER TAIL BANDING	LUAD, THCA	2
c5: Ontology gene sets	HP THIN CLAVICLES	ACC, UVM	2
c5: Ontology gene sets	HP THICKENED CALVARIA	TGCT, THYM	2
c5: Ontology gene sets	HP TERATOMA	READ, THYM	2
c5: Ontology gene sets	HP STOMACH CANCER	KIRC, UVM	2
c5: Ontology gene sets	HP SPINAL CORD TUMOR	PCPG, READ	2
c5: Ontology gene sets	HP SMOOTH MUSCLE ANTIBODY POSITIVITY	LGG, THYM	2
c5: Ontology gene sets	HP SLOWED SLURRED SPEECH	COAD, KIRC	2
c5: Ontology gene sets	HP SINGLE FIBER EMG ABNORMALITY	KIRC, UVM	2
c5: Ontology gene sets	HP SHORTENING OF ALL PHALANGES OF THE TOES	PAAD, UVM	2
c5: Ontology gene sets	HP SHORT FOURTH METATARSAL	HNSC, KIRC	2
c5: Ontology gene sets	HP SEVERE B LYMPHOCYTOPENIA	COAD, THYM	2
c5: Ontology gene sets	HP SECRETORY DIARRHEA	PCPG, UCEC	2

c5: Ontology gene sets	HP SACROCOCCYGEAL TERATOMA	READ, THYM	2
c5: Ontology gene sets	HP RIGHT VENTRICULAR CARDIOMYOPATHY	KIRC, LGG	2
c5: Ontology gene sets	HP RESPIRATORY PARALYSIS	KIRC, LIHC	2
c5: Ontology gene sets	HP REDUCED NATURAL KILLER CELL COUNT	BRCA, LUAD	2
c5: Ontology gene sets	HP REDUCED DELAYED HYPERSENSITIVITY	LGG, THCA	2
c5: Ontology gene sets	HP RECURRENT ASPERGILLUS INFECTIONS	LGG, SKCM	2
c5: Ontology gene sets	HP RECTAL ABSCESS	LGG, SKCM	2
c5: Ontology gene sets	HP PYODERMA	CESC, LUAD	2
c5: Ontology gene sets	HP PROPORTIONATE TALL STATURE	COAD, KIRC	2
c5: Ontology gene sets	HP POSTERIOR RIB CUPPING	ACC, LGG	2
c5: Ontology gene sets	HP PHALANGEAL DISLOCATION	KIRC, MESO	2
c5: Ontology gene sets	HP PERSISTENT PUPILLARY MEMBRANE	KIRC, LUSC	2
c5: Ontology gene sets	HP PERINEAL HYPOSPADIAS	BRCA, UVM	2
c5: Ontology gene sets	HP PERINEAL FISTULA	HNSC, KIRC	2
c5: Ontology gene sets	HP OPTIC NERVE COMPRESSION	KIRC, OV	2
c5: Ontology gene sets	HP NUCHAL RIGIDITY	BRCA, LGG	2
c5: Ontology gene sets	HP NEUTROPHILIA	LGG, UVM	2
c5: Ontology gene sets	HP NARROW FORAMEN OBTURATORIUM	LGG, LUAD	2
c5: Ontology gene sets	HP MONOCYTOSIS	BRCA, CHOL	2
c5: Ontology gene sets	HP MOLLUSCUM CONTAGIOSUM	BRCA, CESC	2
c5: Ontology gene sets	HP MODERATELY REDUCED VISUAL ACUITY	KIRC, READ	2
c5: Ontology gene sets	HP MENINGOENCEPHALOCELE	ACC, MESO	2
c5: Ontology gene sets	HP LYMPHOPROLIFERATIVE DISORDER	LUAD, UVM	2

c5: Ontology gene sets	HP LOP EAR	CESC, UVM	2
c5: Ontology gene sets	HP LIMITED NECK RANGE OF MOTION	STAD, UCEC	2
c5: Ontology gene sets	HP LACK OF INSIGHT	LIHC, UVM	2
c5: Ontology gene sets	HP JUVENILE MYELOMONOCYTIC LEUKEMIA	KIRC, THYM	2
c5: Ontology gene sets	HP INTRAMUSCULAR HEMATOMA	MESO, STAD	2
c5: Ontology gene sets	HP INSULINOMA	KIRC, UCEC	2
c5: Ontology gene sets	HP INCREASED VERTEBRAL HEIGHT	THYM, UVM	2
c5: Ontology gene sets	HP INCREASED CIRCULATING IGG LEVEL	THYM, UVM	2
c5: Ontology gene sets	HP INCREASED C PEPTIDE LEVEL	KIRC, UVM	2
c5: Ontology gene sets	HP INCREASED ARM SPAN	LGG, PRAD	2
c5: Ontology gene sets	HP IMPAIRED COLLAGEN INDUCED PLATELET AGGREGATION	KIRC, UVM	2
c5: Ontology gene sets	HP HYPOPLASTIC DERMOEPIDERMAL HEMIDESMOSOMES	COAD, PAAD	2
c5: Ontology gene sets	HP HYPOPLASIA OF THE VENTRAL PONS	KIRC, LIHC	2
c5: Ontology gene sets	HP HYPOGLYCORRHACHIA	BRCA, LUAD	2
c5: Ontology gene sets	HP HYPERMAGNESIURIA	PAAD, UCEC	2
c5: Ontology gene sets	HP HYPERGASTRINEMIA	KIRC, UCEC	2
c5: Ontology gene sets	HP HORIZONTAL RIBS	KIRC, THYM	2
c5: Ontology gene sets	HP GLOSSITIS	ESCA, UVM	2
c5: Ontology gene sets	HP GENERALIZED HYPERTRICHOSIS	LUAD, PAAD	2
c5: Ontology gene sets	HP GASTROINTESTINAL INFARCTIONS	ACC, BLCA	2
c5: Ontology gene sets	HP FRACTURES OF THE LONG BONES	LIHC, LUAD	2
c5: Ontology gene sets	HP EPISCLERITIS	LUAD, SKCM	2

c5: Ontology gene sets	HP DYSGENESIS OF THE CEREBELLAR VERMIS	ACC, MESO	2
c5: Ontology gene sets	HP DISTAL JOINT LAXITY	KIRC, PCPG	2
c5: Ontology gene sets	HP DECREASED PALMAR CREASES	KIRC, LGG	2
c5: Ontology gene sets	HP DECREASED LYMPHOCYTE PROLIFERATION IN RESPONSE TO ANTI CD3	KIRC, LGG	2
c5: Ontology gene sets	HP DECREASED LYMPHOCYTE APOPTOSIS	SARC, THYM	2
c5: Ontology gene sets	HP DECREASED ACTIVITY OF MITOCHONDRIAL COMPLEX II	BRCA, KIRP	2
c5: Ontology gene sets	HP CUTANEOUS ANERGY	HNSC, SKCM	2
c5: Ontology gene sets	HP CONSPICUOUSLY HAPPY DISPOSITION	COAD, THYM	2
c5: Ontology gene sets	HP CONGENITAL BILATERAL HIP DISLOCATION	KIRC, LGG	2
c5: Ontology gene sets	HP COLPOCEPHALY	TGCT, THYM	2
c5: Ontology gene sets	HP CIGARETTE PAPER SCARS	KIRP, UVM	2
c5: Ontology gene sets	HP CHRONIC ORAL CANDIDIASIS	KIRC, THYM	2
c5: Ontology gene sets	HP CHRONIC HEPATITIS DUE TO CRYPTOSPORIDIUM INFECTION	HNSC, SKCM	2
c5: Ontology gene sets	HP CHROMOSOMAL BREAKAGE INDUCED BY CROSSLINKING AGENTS	KIRP, LIHC	2
c5: Ontology gene sets	HP CERVICAL LYMPHADENOPATHY	CESC, READ	2
c5: Ontology gene sets	HP CARPAL BONE HYPOPLASIA	KIRC, UCEC	2
c5: Ontology gene sets	HP BURKITT LYMPHOMA	SARC, THYM	2
c5: Ontology gene sets	HP BLEPHAROCHALASIS	KIRC, LGG	2
c5: Ontology gene sets	HP BIRTH LENGTH LESS THAN 3RD PERCENTILE	ACC, KIRC	2
c5: Ontology gene sets	HP BEADED RIBS	BLCA, KIRC	2
c5: Ontology gene sets	HP BASAL GANGLIA CYSTS	KIRC, KIRP	2

c5: Ontology gene sets	HP ASYMMETRY OF THE EARS	KIRC, KIRP	2
c5: Ontology gene sets	HP ANTINEUTROPHIL ANTIBODY POSITIVITY	SKCM, THYM	2
c5: Ontology gene sets	HP ALOPECIA TOTALIS	COAD, UVM	2
c5: Ontology gene sets	HP AGENESIS OF LATERAL INCISOR	KIRC, THYM	2
c5: Ontology gene sets	HP AGAMMAGLOBULINEMIA	BRCA, HNSC	2
c5: Ontology gene sets	HP ACUTE OTITIS MEDIA	HNSC, SKCM	2
c5: Ontology gene sets	HP ABSENT INNER DYNEIN ARMS	BRCA, LGG	2
c5: Ontology gene sets	HP ABSENT INNER AND OUTER DYNEIN ARMS	KIRP, LGG	2
c5: Ontology gene sets	HP ABNORMALITY OF ULNAR METAPHYSIS	KIRC, READ	2
c5: Ontology gene sets	HP ABNORMALITY OF THE TONSILS	CESC, READ	2
c5: Ontology gene sets	HP ABNORMALITY OF THE NASAL BONE	KICH, KIRC	2
c5: Ontology gene sets	HP ABNORMAL TARSAL OSSIFICATION	KIRC, LUAD	2
c5: Ontology gene sets	HP ABNORMAL PROPORTION OF CD8 POSITIVE T CELLS	SKCM, UVM	2
c5: Ontology gene sets	HP ABNORMAL LENGTH OF CORPUS CALLOSUM	BRCA, LGG	2
c5: Ontology gene sets	HP ABNORMAL COCHLEA MORPHOLOGY	READ, TGCT	2
c3: regulatory target gene sets	HOXA3 01	KIRC, TGCT	2
c7: immunologic signature gene sets	HOWARD DENDRITIC CELL INACT MONOV INFLUENZA A INDONESIA 05 2005 H5N1 AGE 18 49YO 3DY UP	KIRC, LGG	2

c8: cell type signature gene sets	HAY BONE MARROW CD34 POS LMPP	SKCM, THYM	2
c2: curated gene sets	GRANDVAUX IFN RESPONSE NOT VIA IRF3	KIRC, SKCM	2
c5: Ontology gene sets	GOMF ZINC ION TRANSMEMBRANE TRANSPORTER ACTIVITY	COAD, READ	2
c5: Ontology gene sets	GOMF VOLTAGE GATED CALCIUM CHANNEL ACTIVITY INVOLVED IN CARDIAC MUSCLE CELL ACTION POTENTIAL	BRCA, KIRP	2
c5: Ontology gene sets	GOMF UDP N ACETYLGLUCOSAMINE TRANSMEMBRANE TRANSPORTER ACTIVITY	KIRC, LGG	2
c5: Ontology gene sets	GOMF UBIQUITIN LIKE MODIFIER ACTIVATING ENZYME ACTIVITY	KIRP, LIHC	2
c5: Ontology gene sets	GOMF TUMOR NECROSIS FACTOR BINDING	KIRC, LGG	2
c5: Ontology gene sets	GOMF TRNA ADENINE METHYLTRANSFERASE ACTIVITY	KIRC, LIHC	2
c5: Ontology gene sets	GOMF THIOESTERASE BINDING	BRCA, LGG	2
c5: Ontology gene sets	GOMF TFIID CLASS TRANSCRIPTION FACTOR COMPLEX BINDING	ACC, KIRC	2
c5: Ontology gene sets	GOMF T CELL RECEPTOR BINDING	LGG, SKCM	2
c5: Ontology gene sets	GOMF SINGLE STRANDED DNA HELICASE ACTIVITY	KIRP, MESO	2
c5: Ontology gene sets	GOMF SIALIC ACID TRANSMEMBRANE TRANSPORTER ACTIVITY	BLCA, KIRC	2
c5: Ontology gene sets	GOMF SERINE TYPE CARBOXYPEPTIDASE ACTIVITY	LGG, UVM	2
c5: Ontology gene sets	GOMF RRNA CYTOSINE METHYLTRANSFERASE ACTIVITY	KICH, KIRC	2

c5: Ontology gene sets	GOMF RIBOFLAVIN TRANSMEMBRANE TRANSPORTER ACTIVITY	MESO, PRAD	2
c5: Ontology gene sets	GOMF PROTEIN LYSINE 6 OXIDASE ACTIVITY	GBM, LGG	2
c5: Ontology gene sets	GOMF PROCOLLAGEN PROLINE DIOXYGENASE ACTIVITY	GBM, KIRP	2
c5: Ontology gene sets	GOMF PROCOLLAGEN PROLINE 4 DIOXYGENASE ACTIVITY	GBM, KIRP	2
c5: Ontology gene sets	GOMF PORIN ACTIVITY	LUAD, UVM	2
c5: Ontology gene sets	GOMF PHOSPHATIDYLINOSITOL TRANSFER ACTIVITY	ACC, LUSC	2
c5: Ontology gene sets	GOMF PHOSPHATIDYLINOSITOL MONOPHOSPHATE PHOSPHATASE ACTIVITY	KIRC, THYM	2
c5: Ontology gene sets	GOMF PHOSPHATIDYLINOSITOL 3 KINASE ACTIVITY	READ, THYM	2
c5: Ontology gene sets	GOMF PHOSPHATIDYLCHOLINE FLOPPASE ACTIVITY	KIRC, SKCM	2
c5: Ontology gene sets	GOMF OXIDOREDUCTASE ACTIVITY ACTING ON NAD P H OXYGEN AS ACCEPTOR	BRCA, COAD	2
c5: Ontology gene sets	GOMF OXIDOREDUCTASE ACTIVITY ACTING ON NAD P H HEME PROTEIN AS ACCEPTOR	DLBC, KIRC	2
c5: Ontology gene sets	GOMF MYOSIN LIGHT CHAIN BINDING	KIRC, LUAD	2
c5: Ontology gene sets	GOMF LYSOPHOSPHATIDIC ACID RECEPTOR ACTIVITY	ACC, PCPG	2
c5: Ontology gene sets	GOMF LIPID PHOSPHATASE ACTIVITY	KIRC, MESO	2
c5: Ontology gene sets	GOMF LIPID ANTIGEN BINDING	CESC, SARC	2

c5: Ontology gene sets	GOMF LIGASE ACTIVITY FORMING PHOSPHORIC ESTER BONDS	HNSC, LIHC	2
c5: Ontology gene sets	GOMF L GLUTAMINE TRANSMEMBRANE TRANSPORTER ACTIVITY	BRCA, MESO	2
c5: Ontology gene sets	GOMF INTRAMOLECULAR OXIDOREDUCTASE ACTIVITY TRANSPOSING C C BONDS	KIRP, PRAD	2
c5: Ontology gene sets	GOMF INOSITOL TRISPHOSPHATE KINASE ACTIVITY	COAD, LUAD	2
c5: Ontology gene sets	GOMF ICOSATETRAENOIC ACID BINDING	LUAD, SKCM	2
c5: Ontology gene sets	GOMF HYDROLASE ACTIVITY ACTING ON CARBON NITROGEN BUT NOT PEPTIDE BONDS IN CYCLIC AMIDES	KIRC, UVM	2
c5: Ontology gene sets	GOMF GPI ANCHOR BINDING	COAD, UVM	2
c5: Ontology gene sets	GOMF DYNACTIN BINDING	KIRC, LIHC	2
c5: Ontology gene sets	GOMF DOUBLE STRANDED TELOMERIC DNA BINDING	KIRC, LGG	2
c5: Ontology gene sets	GOMF DNA TRANSLOCASE ACTIVITY	KICH, KIRC	2
c5: Ontology gene sets	GOMF DNA REPLICATION ORIGIN BINDING	KICH, LIHC	2
c5: Ontology gene sets	GOMF DEOXYCYTIDINE DEAMINASE ACTIVITY	CESC, LGG	2
c5: Ontology gene sets	GOMF CYCLIN DEPENDENT PROTEIN SERINE THREONINE KINASE INHIBITOR ACTIVITY	KIRC, KIRP	2
c5: Ontology gene sets	GOMF COMPLEMENT COMPONENT C3B BINDING	LGG, SKCM	2
c5: Ontology gene sets	GOMF COLLAGEN BINDING INVOLVED IN CELL MATRIX ADHESION	LGG, MESO	2

c5: Ontology gene sets	GOMF CCR2 CHEMOKINE RECEPTOR BINDING	GBM, SKCM	2
c5: Ontology gene sets	GOMF BOX H ACA SNORNA BINDING	KIRP, READ	2
c5: Ontology gene sets	GOMF ACYL COA DEHYDROGENASE ACTIVITY	KIRP, UCEC	2
c5: Ontology gene sets	GOMF ACETYL COA C ACYLTRANSFERASE ACTIVITY	KIRC, KIRP	2
c5: Ontology gene sets	GOMF ACETYL COA C ACETYLTRANSFERASE ACTIVITY	KIRC, KIRP	2
c5: Ontology gene sets	GOMF 5 DEOXYRIBOSE 5 PHOSPHATE LYASE ACTIVITY	ACC, LUAD	2
c5: Ontology gene sets	GOMF 3 HYDROXYACYL COA DEHYDROGENASE ACTIVITY	KIRC, UVM	2
c5: Ontology gene sets	GOMF 3 5 DNA HELICASE ACTIVITY	KICH, LIHC	2
c5: Ontology gene sets	GOMF 1 PHOSPHATIDYLINOSITOL 3 KINASE ACTIVITY	READ, THYM	2
c5: Ontology gene sets	GOCC VCB COMPLEX	LGG, LIHC	2
c5: Ontology gene sets	GOCC TRNA METHYLTRANSFERASE COMPLEX	KIRC, LIHC	2
c5: Ontology gene sets	GOCC POLAR MICROTUBULE	ACC, LIHC	2
c5: Ontology gene sets	GOCC PHAGOPHORE ASSEMBLY SITE	DLBC, THYM	2
c5: Ontology gene sets	GOCC ORIGIN RECOGNITION COMPLEX	KIRC, LIHC	2
c5: Ontology gene sets	GOCC NEUROFIBRILLARY TANGLE	BRCA, LGG	2
c5: Ontology gene sets	GOCC N TERMINAL PROTEIN ACETYLTRANSFERASE COMPLEX	KIRC, MESO	2
c5: Ontology gene sets	GOCC MISMATCH REPAIR COMPLEX	KICH, PRAD	2
c5: Ontology gene sets	GOCC MALE PRONUCLEUS	LIHC, SKCM	2

c5: Ontology gene sets	GOCC KINETOCHORE MICROTUBULE	KIRC, THYM	2
c5: Ontology gene sets	GOCC GARP COMPLEX	LGG, MESO	2
c5: Ontology gene sets	GOCC FIBRILLAR COLLAGEN TRIMER	ACC, KIRP	2
c5: Ontology gene sets	GOCC FEMALE PRONUCLEUS	ACC, MESO	2
c5: Ontology gene sets	GOCC F ACTIN CAPPING PROTEIN COMPLEX	KICH, KIRC	2
c5: Ontology gene sets	GOCC EXTERNAL SIDE OF APICAL PLASMA MEMBRANE	KIRC, MESO	2
c5: Ontology gene sets	GOCC EUKARYOTIC TRANSLATION INITIATION FACTOR 3 COMPLEX EIF3M	LUAD, PRAD	2
c5: Ontology gene sets	GOCC ESC E Z COMPLEX	LGG, LIHC	2
c5: Ontology gene sets	GOCC EMC COMPLEX	ESCA, KICH	2
c5: Ontology gene sets	GOCC EKC KEOPS COMPLEX	LIHC, UCEC	2
c5: Ontology gene sets	GOCC EARP COMPLEX	LGG, MESO	2
c5: Ontology gene sets	GOCC DNA REPLICATION FACTOR C COMPLEX	KICH, LGG	2
c5: Ontology gene sets	GOCC CYCLIN A2 CDK2 COMPLEX	ACC, KIRC	2
c5: Ontology gene sets	GOCC CILIARY TRANSITION FIBER	MESO, THYM	2
c5: Ontology gene sets	GOCC CIA COMPLEX	ACC, COAD	2
c5: Ontology gene sets	GOCC CD95 DEATH INDUCING SIGNALING COMPLEX	LGG, SARC	2
c5: Ontology gene sets	GOCC BOX H ACA SNORNP COMPLEX	KIRP, READ	2
c5: Ontology gene sets	GOCC ARP2 3 PROTEIN COMPLEX	KIRC, LIHC	2
c5: Ontology gene sets	GOBP VESTIBULOCOCHLEAR NERVE MORPHOGENESIS	BLCA, DLBC	2
c5: Ontology gene sets	GOBP VACUOLE FUSION	LGG, PAAD	2
c5: Ontology gene sets	GOBP UTP METABOLIC PROCESS	ACC, LGG	2
c5: Ontology gene sets	GOBP UMP CATABOLIC PROCESS	SKCM, UVM	2

c5: Ontology gene sets	GOBP UDP N ACETYLGLUCOSAMINE TRANSMEMBRANE TRANSPORT	KIRC, LGG	2
c5: Ontology gene sets	GOBP TRNA AMINOACYLATION FOR MITOCHONDRIAL PROTEIN TRANSLATION	KIRC, PRAD	2
c5: Ontology gene sets	GOBP TRANSDIFFERENTIATION	KIRP, THYM	2
c5: Ontology gene sets	GOBP TOLL LIKE RECEPTOR 7 SIGNALING PATHWAY	LGG, SKCM	2
c5: Ontology gene sets	GOBP THREONINE CATABOLIC PROCESS	COAD, UVM	2
c5: Ontology gene sets	GOBP TETRAHYDROFOLATE INTERCONVERSION	KIRP, MESO	2
c5: Ontology gene sets	GOBP TETRAHYDROFOLATE BIOSYNTHETIC PROCESS	ACC, LUAD	2
c5: Ontology gene sets	GOBP TELOMERIC D LOOP DISASSEMBLY	KIRC, THYM	2
c5: Ontology gene sets	GOBP TELOMERE ASSEMBLY	KIRC, LIHC	2
c5: Ontology gene sets	GOBP T CELL ANTIGEN PROCESSING AND PRESENTATION	CESC, UVM	2
c5: Ontology gene sets	GOBP SUBSTRATE DEPENDENT CELL MIGRATION CELL EXTENSION	LUSC, SKCM	2
c5: Ontology gene sets	GOBP STRAND INVASION	KICH, MESO	2
c5: Ontology gene sets	GOBP SPINDLE ELONGATION	KIRP, LGG	2
c5: Ontology gene sets	GOBP SMOOTHENED SIGNALING PATHWAY INVOLVED IN VENTRAL SPINAL CORD PATTERNING	LGG, UCEC	2
c5: Ontology gene sets	GOBP SMOOTH MUSCLE CELL MATRIX ADHESION	MESO, PRAD	2
c5: Ontology gene sets	GOBP SINOATRIAL NODE CELL DEVELOPMENT	ACC, MESO	2
c5: Ontology gene sets	GOBP SIALIC ACID TRANSPORT	BLCA, KIRC	2
c5: Ontology gene sets	GOBP SELF PROTEOLYSIS	COAD, READ	2

c5: Ontology gene sets	GOBP RIBOSOMAL LARGE SUBUNIT EXPORT FROM NUCLEUS	KICH, UVM	2
c5: Ontology gene sets	GOBP RIBOFLAVIN TRANSPORT	MESO, PRAD	2
c5: Ontology gene sets	GOBP RHOMBOMERE DEVELOPMENT	ACC, GBM	2
c5: Ontology gene sets	GOBP RESPONSE TO SORBITOL	BRCA, KIRC	2
c5: Ontology gene sets	GOBP RESPONSE TO SODIUM ARSENITE	BRCA, UVM	2
c5: Ontology gene sets	GOBP RESPONSE TO L ASCORBIC ACID	BRCA, UVM	2
c5: Ontology gene sets	GOBP RESPONSE TO IRON III ION	ACC, LGG	2
c5: Ontology gene sets	GOBP RESPONSE TO INTERLEUKIN 2	BRCA, KIRC	2
c5: Ontology gene sets	GOBP RESPONSE TO CAMPTOTHECIN	KICH, KIRC	2
c5: Ontology gene sets	GOBP REPLICATIVE SENESCENCE	BRCA, READ	2
c5: Ontology gene sets	GOBP REPLICATION FORK PROTECTION	ACC, LGG	2
c5: Ontology gene sets	GOBP REGULATION OF VITAMIN D BIOSYNTHETIC PROCESS	LGG, UVM	2
c5: Ontology gene sets	GOBP REGULATION OF VESICLE TRANSPORT ALONG MICROTUBULE	COAD, UCEC	2
c5: Ontology gene sets	GOBP REGULATION OF TOLL LIKE RECEPTOR 7 SIGNALING PATHWAY	LGG, SKCM	2
c5: Ontology gene sets	GOBP REGULATION OF T CELL EXTRAVASATION	LUAD, SKCM	2
c5: Ontology gene sets	GOBP REGULATION OF T CELL COSTIMULATION	LGG, SKCM	2
c5: Ontology gene sets	GOBP REGULATION OF SYNAPTIC VESICLE PRIMING	KIRC, THCA	2

c5: Ontology gene sets	GOBP REGULATION OF RNA EXPORT FROM NUCLEUS	LIHC, THYM	2
c5: Ontology gene sets	GOBP REGULATION OF PROTEIN LOCALIZATION TO CILIUM	KICH, PAAD	2
c5: Ontology gene sets	GOBP REGULATION OF PROTEIN LOCALIZATION TO CELL CORTEX	LIHC, LUAD	2
c5: Ontology gene sets	GOBP REGULATION OF PROTEIN KINASE A SIGNALING	KIRC, UVM	2
c5: Ontology gene sets	GOBP REGULATION OF PROTEIN AUTOUBIQUITINATION	KIRC, PCPG	2
c5: Ontology gene sets	GOBP REGULATION OF NATURAL KILLER CELL CHEMOTAXIS	LGG, SKCM	2
c5: Ontology gene sets	GOBP REGULATION OF MITOTIC SPINDLE ASSEMBLY	KIRC, THYM	2
c5: Ontology gene sets	GOBP REGULATION OF MITOCHONDRIAL MRNA STABILITY	ACC, KIRC	2
c5: Ontology gene sets	GOBP REGULATION OF MICROVILLUS ASSEMBLY	GBM, LGG	2
c5: Ontology gene sets	GOBP REGULATION OF METAPHASE PLATE CONGRESSION	KIRC, MESO	2
c5: Ontology gene sets	GOBP REGULATION OF ISOMERASE ACTIVITY	BLCA, UCEC	2
c5: Ontology gene sets	GOBP REGULATION OF FEMALE GONAD DEVELOPMENT	BRCA, KIRC	2
c5: Ontology gene sets	GOBP REGULATION OF CILIUM BEAT FREQUENCY INVOLVED IN CILIARY MOTILITY	PAAD, UCEC	2
c5: Ontology gene sets	GOBP REGULATION OF CHRONIC INFLAMMATORY RESPONSE	CESC, SKCM	2
c5: Ontology gene sets	GOBP REGULATION OF CHROMOSOME CONDENSATION	KIRC, SKCM	2

c5: Ontology gene sets	GOBP REGULATION OF CELLULAR RESPONSE TO MACROPHAGE COLONY STIMULATING FACTOR STIMULUS	OV, SKCM	2
c5: Ontology gene sets	GOBP REGULATION OF BONE DEVELOPMENT	KIRC, LUAD	2
c5: Ontology gene sets	GOBP REGULATION OF ATTACHMENT OF SPINDLE MICROTUBULES TO KINETOCHORE	KIRC, UCEC	2
c5: Ontology gene sets	GOBP RECOGNITION OF APOPTOTIC CELL	COAD, LUAD	2
c5: Ontology gene sets	GOBP PYRIMIDINE NUCLEOSIDE MONOPHOSPHATE CATABOLIC PROCESS	SKCM, UVM	2
c5: Ontology gene sets	GOBP PROTEIN RETENTION IN GOLGI APPARATUS	COAD, KIRC	2
c5: Ontology gene sets	GOBP PROTEIN O LINKED FUCOSYLATION	BRCA, LGG	2
c5: Ontology gene sets	GOBP PROTEIN LOCALIZATION TO CYTOPLASMIC STRESS GRANULE	LIHC, SKCM	2
c5: Ontology gene sets	GOBP PROTEIN LINEAR POLYUBIQUITINATION	KIRC, SKCM	2
c5: Ontology gene sets	GOBP PROTEIN INSERTION INTO ER MEMBRANE BY STOP TRANSFER MEMBRANE ANCHOR SEQUENCE	ESCA, KICH	2
c5: Ontology gene sets	GOBP PROTEIN FOLDING IN ENDOPLASMIC RETICULUM	LGG, UVM	2
c5: Ontology gene sets	GOBP POSTTRANSLATIONAL PROTEIN TARGETING TO MEMBRANE TRANSLOCATION	KIRC, LGG	2

c5: Ontology gene sets	GOBP POSITIVE REGULATION OF TELOMERASE RNA LOCALIZATION TO CAJAL BODY	KICH, LGG	2
c5: Ontology gene sets	GOBP POSITIVE REGULATION OF SPINDLE CHECKPOINT	ACC, LIHC	2
c5: Ontology gene sets	GOBP POSITIVE REGULATION OF RRNA PROCESSING	KICH, KIRP	2
c5: Ontology gene sets	GOBP POSITIVE REGULATION OF RETINOIC ACID RECEPTOR SIGNALING PATHWAY	ACC, KIRC	2
c5: Ontology gene sets	GOBP POSITIVE REGULATION OF OSTEOBLAST PROLIFERATION	LGG, LIHC	2
c5: Ontology gene sets	GOBP POSITIVE REGULATION OF NMDA GLUTAMATE RECEPTOR ACTIVITY	LGG, SKCM	2
c5: Ontology gene sets	GOBP POSITIVE REGULATION OF NATURAL KILLER CELL MEDIATED IMMUNE RESPONSE TO TUMOR CELL	CESC, THCA	2
c5: Ontology gene sets	GOBP POSITIVE REGULATION OF MYELOID LEUKOCYTE CYTOKINE PRODUCTION INVOLVED IN IMMUNE RESPONSE	KIRC, THYM	2
c5: Ontology gene sets	GOBP POSITIVE REGULATION OF MRNA 3 END PROCESSING	LIHC, THYM	2
c5: Ontology gene sets	GOBP POSITIVE REGULATION OF MITOCHONDRIAL CALCIUM ION CONCENTRATION	HNSC, KIRC	2
c5: Ontology gene sets	GOBP POSITIVE REGULATION OF MEMBRANE PROTEIN ECTODOMAIN PROTEOLYSIS	OV, UVM	2

c5: Ontology gene sets	GOBP POSITIVE REGULATION OF MEIOTIC CELL CYCLE PHASE TRANSITION	ACC, KIRP	2
c5: Ontology gene sets	GOBP POSITIVE REGULATION OF HYPERSENSITIVITY	LGG, SKCM	2
c5: Ontology gene sets	GOBP POSITIVE REGULATION OF HUMORAL IMMUNE RESPONSE MEDIATED BY CIRCULATING IMMUNOGLOBULIN	HNSC, UVM	2
c5: Ontology gene sets	GOBP POSITIVE REGULATION OF HELICASE ACTIVITY	KICH, THYM	2
c5: Ontology gene sets	GOBP POSITIVE REGULATION OF ENDOPLASMIC RETICULUM STRESS INDUCED INTRINSIC APOPTOTIC SIGNALING PATHWAY	KIRC, KIRP	2
c5: Ontology gene sets	GOBP POSITIVE REGULATION OF DNA DEPENDENT DNA REPLICATION	ACC, KIRC	2
c5: Ontology gene sets	GOBP POSITIVE REGULATION OF CHROMOSOME SEGREGATION	KIRC, LIHC	2
c5: Ontology gene sets	GOBP POSITIVE REGULATION OF CELLULAR RESPIRATION	SKCM, UVM	2
c5: Ontology gene sets	GOBP POSITIVE REGULATION OF CELL CYCLE CHECKPOINT	ACC, LIHC	2
c5: Ontology gene sets	GOBP POSITIVE REGULATION OF ARP2 3 COMPLEX MEDIATED ACTIN NUCLEATION	COAD, UVM	2
c5: Ontology gene sets	GOBP POSITIVE REGULATION BY HOST OF VIRAL GENOME REPLICATION	KIRC, OV	2
c5: Ontology gene sets	GOBP PEPTIDYL LYSINE OXIDATION	GBM, LGG	2

c5: Ontology gene sets	GOBP PEPTIDYL LYSINE HYDROXYLATION	COAD, KICH	2
c5: Ontology gene sets	GOBP OUTER MITOCHONDRIAL MEMBRANE ORGANIZATION	LIHC, UCEC	2
c5: Ontology gene sets	GOBP ODONTOBLAST DIFFERENTIATION	UCEC, UVM	2
c5: Ontology gene sets	GOBP NEGATIVE REGULATION OF VITAMIN METABOLIC PROCESS	BRCA, LGG	2
c5: Ontology gene sets	GOBP NEGATIVE REGULATION OF VITAMIN D BIOSYNTHETIC PROCESS	LGG, UVM	2
c5: Ontology gene sets	GOBP NEGATIVE REGULATION OF TYPE B PANCREATIC CELL APOPTOTIC PROCESS	PAAD, UCEC	2
c5: Ontology gene sets	GOBP NEGATIVE REGULATION OF TUBULIN DEACETYLATION	KIRC, THYM	2
c5: Ontology gene sets	GOBP NEGATIVE REGULATION OF TELOMERE CAPPING	KIRC, PRAD	2
c5: Ontology gene sets	GOBP NEGATIVE REGULATION OF PHOTORECEPTOR CELL DIFFERENTIATION	LUAD, MESO	2
c5: Ontology gene sets	GOBP NEGATIVE REGULATION OF HISTONE H3 K4 METHYLATION	LIHC, LUAD	2
c5: Ontology gene sets	GOBP NEGATIVE REGULATION OF GLYCOGEN METABOLIC PROCESS	MESO, UCEC	2
c5: Ontology gene sets	GOBP NEGATIVE REGULATION OF FAT CELL PROLIFERATION	KIRC, MESO	2
c5: Ontology gene sets	GOBP NEGATIVE REGULATION OF EXTRACELLULAR MATRIX DISASSEMBLY	KIRP, LUSC	2
c5: Ontology gene sets	GOBP NEGATIVE REGULATION OF CYTOKINESIS	KICH, KIRP	2

c5: Ontology gene sets	GOBP NEGATIVE REGULATION OF CHROMATIN SILENCING	ACC, SARC	2
c5: Ontology gene sets	GOBP NEGATIVE REGULATION OF CENTRIOLE REPLICATION	MESO, PAAD	2
c5: Ontology gene sets	GOBP NEGATIVE REGULATION OF CELL CYCLE CHECKPOINT	ACC, KIRC	2
c5: Ontology gene sets	GOBP NEGATIVE REGULATION OF ACUTE INFLAMMATORY RESPONSE TO ANTIGENIC STIMULUS	COAD, LUAD	2
c5: Ontology gene sets	GOBP MYELOID DENDRITIC CELL CYTOKINE PRODUCTION	KIRC, PAAD	2
c5: Ontology gene sets	GOBP MULTIVESICULAR BODY ORGANIZATION	PRAD, THYM	2
c5: Ontology gene sets	GOBP MRNA CLEAVAGE INVOLVED IN MRNA PROCESSING	MESO, THYM	2
c5: Ontology gene sets	GOBP MRNA 3 END PROCESSING BY STEM LOOP BINDING AND CLEAVAGE	LIHC, MESO	2
c5: Ontology gene sets	GOBP MONOUBIQUITINATED PROTEIN DEUBIQUITINATION	SKCM, THYM	2
c5: Ontology gene sets	GOBP MITOTIC CHROMOSOME CONDENSATION	LIHC, LUAD	2
c5: Ontology gene sets	GOBP MITOCHONDRIAL RIBOSOME ASSEMBLY	ACC, SKCM	2
c5: Ontology gene sets	GOBP MITOCHONDRIAL PROTEIN CATABOLIC PROCESS	CHOL, KIRC	2
c5: Ontology gene sets	GOBP MICROVILLUS ASSEMBLY	COAD, KIRC	2
c5: Ontology gene sets	GOBP METANEPHRIC MESENCHYMAL CELL DIFFERENTIATION	KIRC, SKCM	2
c5: Ontology gene sets	GOBP MEMBRANE RAFT LOCALIZATION	BRCA, THYM	2

c5: Ontology gene sets	GOBP MEIOTIC MISMATCH REPAIR	LGG, UCEC	2
c5: Ontology gene sets	GOBP MEIOTIC CHROMOSOME CONDENSATION	ACC, LGG	2
c5: Ontology gene sets	GOBP MDA 5 SIGNALING PATHWAY	KIRC, THYM	2
c5: Ontology gene sets	GOBP MAINTENANCE OF DNA REPEAT ELEMENTS	KIRC, UCEC	2
c5: Ontology gene sets	GOBP LEUKOTRIENE TRANSPORT	KIRC, LIHC	2
c5: Ontology gene sets	GOBP LEUKOCYTE AGGREGATION	KIRC, SARC	2
c5: Ontology gene sets	GOBP KINETOCHORE ORGANIZATION	COAD, READ	2
c5: Ontology gene sets	GOBP INTRINSIC APOPTOTIC SIGNALING PATHWAY IN RESPONSE TO HYPOXIA	KIRC, LGG	2
c5: Ontology gene sets	GOBP INTERLEUKIN 23 MEDIATED SIGNALING PATHWAY	BLCA, CESC	2
c5: Ontology gene sets	GOBP INSULIN METABOLIC PROCESS	HNSC, UCEC	2
c5: Ontology gene sets	GOBP IMMUNE COMPLEX CLEARANCE	LGG, SKCM	2
c5: Ontology gene sets	GOBP HISTONE H4 K20 TRIMETHYLATION	MESO, SKCM	2
c5: Ontology gene sets	GOBP HISTONE H3 K27 TRIMETHYLATION	LGG, MESO	2
c5: Ontology gene sets	GOBP HISTONE H3 K27 METHYLATION	LGG, UCEC	2
c5: Ontology gene sets	GOBP HEPATIC STELLATE CELL ACTIVATION	LGG, LUAD	2
c5: Ontology gene sets	GOBP GROWTH PLATE CARTILAGE CHONDROCYTE DEVELOPMENT	LGG, PAAD	2
c5: Ontology gene sets	GOBP GLUTAMINE TRANSPORT	BRCA, MESO	2

c5: Ontology gene sets	GOBP GLOMERULAR EPITHELIAL CELL DIFFERENTIATION	KIRC, THCA	2
c5: Ontology gene sets	GOBP GERMINAL CENTER B CELL DIFFERENTIATION	COAD, LUAD	2
c5: Ontology gene sets	GOBP GAMMA DELTA T CELL DIFFERENTIATION	BRCA, UVM	2
c5: Ontology gene sets	GOBP FREE UBIQUITIN CHAIN POLYMERIZATION	KIRC, UVM	2
c5: Ontology gene sets	GOBP FOREGUT MORPHOGENESIS	LUAD, THYM	2
c5: Ontology gene sets	GOBP FOLIC ACID CONTAINING COMPOUND BIOSYNTHETIC PROCESS	KIRC, KIRP	2
c5: Ontology gene sets	GOBP FEMALE MEIOSIS I	KICH, LIHC	2
c5: Ontology gene sets	GOBP EXTRACELLULAR EXOSOME ASSEMBLY	LGG, UVM	2
c5: Ontology gene sets	GOBP ERBB2 SIGNALING PATHWAY	DLBC, READ	2
c5: Ontology gene sets	GOBP EOSINOPHIL ACTIVATION	CESC, KIRC	2
c5: Ontology gene sets	GOBP ENDOTHELIAL CELL FATE COMMITMENT	KIRC, LGG	2
c5: Ontology gene sets	GOBP ENDOPLASMIC RETICULUM MEMBRANE ORGANIZATION	COAD, UCEC	2
c5: Ontology gene sets	GOBP ENDOCANNABINOID SIGNALING PATHWAY	DLBC, KIRP	2
c5: Ontology gene sets	GOBP DOLICHOL METABOLIC PROCESS	KIRC, SKCM	2
c5: Ontology gene sets	GOBP DNA UNWINDING INVOLVED IN DNA REPLICATION	KIRC, UVM	2
c5: Ontology gene sets	GOBP DNA REPLICATION SYNTHESIS OF RNA PRIMER	KICH, LGG	2
c5: Ontology gene sets	GOBP DIAPHRAGM DEVELOPMENT	LGG, PCPG	2
c5: Ontology gene sets	GOBP DENTINOGENESIS	STAD, UCEC	2

c5: Ontology gene sets	GOBP DE NOVO IMP BIOSYNTHETIC PROCESS	ACC, UVM	2
c5: Ontology gene sets	GOBP CORTICOSPINAL TRACT MORPHOGENESIS	BLCA, OV	2
c5: Ontology gene sets	GOBP COP9 SIGNALOSOME ASSEMBLY	BLCA, UVM	2
c5: Ontology gene sets	GOBP CHROMOSOME MOVEMENT TOWARDS SPINDLE POLE	KIRP, LUAD	2
c5: Ontology gene sets	GOBP CHONDROCYTE DEVELOPMENT INVOLVED IN ENDOCHONDRAL BONE MORPHOGENESIS	COAD, PAAD	2
c5: Ontology gene sets	GOBP CELLULAR RESPONSE TO SODIUM ARSENITE	BRCA, UVM	2
c5: Ontology gene sets	GOBP CELLULAR RESPONSE TO ETHER	SKCM, UVM	2
c5: Ontology gene sets	GOBP CELL PROLIFERATION INVOLVED IN METANEPHROS DEVELOPMENT	LUAD, UCEC	2
c5: Ontology gene sets	GOBP CD4 POSITIVE CD25 POSITIVE ALPHA BETA REGULATORY T CELL DIFFERENTIATION	SKCM, UVM	2
c5: Ontology gene sets	GOBP CANONICAL WNT SIGNALING PATHWAY INVOLVED IN CARDIAC MUSCLE CELL FATE COMMITMENT	GBM, LUAD	2
c5: Ontology gene sets	GOBP BASIC AMINO ACID TRANSMEMBRANE TRANSPORT	BRCA, UVM	2
c5: Ontology gene sets	GOBP BASE EXCISION REPAIR GAP FILLING	ACC, UVM	2
c5: Ontology gene sets	GOBP B 1 B CELL DIFFERENTIATION	LGG, SKCM	2

c5: Ontology gene sets	GOBP ASTROCYTE CELL MIGRATION	HNSC, LGG	2
c5: Ontology gene sets	GOBP APOPTOTIC PROCESS INVOLVED IN BLOOD VESSEL MORPHOGENESIS	KIRC, UCEC	2
c5: Ontology gene sets	GOBP ANTIGEN PROCESSING AND PRESENTATION OF EXOGENOUS PEPTIDE ANTIGEN VIA MHC CLASS I TAP INDEPENDENT	LGG, THCA	2
c5: Ontology gene sets	GOBP ANTIGEN PROCESSING AND PRESENTATION ENDOGENOUS LIPID ANTIGEN VIA MHC CLASS IB	CESC, SARC	2
c5: Ontology gene sets	GOBP ACTIVATION INDUCED CELL DEATH OF T CELLS	LIHC, UCEC	2
c5: Ontology gene sets	GOBP 10 FORMYLTETRAHYDROFOLATE METABOLIC PROCESS	BLCA, PAAD	2
c4: computational gene sets	GNF2 H2AFX	LIHC, MESO	2
c4: computational gene sets	GNF2 BUB1	LIHC, LUAD	2
c7: immunologic signature gene sets	GIAROLA SILVA BLOOD PANDEMRIX AGE 21 51YO 3DY UP	LGG, SKCM	2
c3: regulatory target gene sets	GCGCCTT MIR525 MIR524	BLCA, READ	2
c2: curated gene sets	FUNG IL2 SIGNALING 1	KIRC, UVM	2
c2: curated gene sets	FUJIWARA PARK2 IN LIVER CANCER DN	HNSC, KIRC	2
c2: curated gene sets	FRIDMAN SENESCENCE DN	KIRC, KIRP	2
c2: curated gene sets	FARDIN HYPOXIA 9	KICH, LGG	2
c8: cell type signature gene sets	DURANTE ADULT OLFACTORY NEUROEPITHELIUM CD4 T CELLS	SKCM, THYM	2
c2: curated gene sets	CROONQUIST STROMAL STIMULATION DN	KIRC, LGG	2

c2: curated gene sets	COLLIS PRKDC SUBSTRATES	LIHC, PRAD	2
c7: immunologic signature gene sets	COLE BLOOD FLUMIST QUADRIVALENT AGE 03 17YO 7DY UP	OV, SKCM	2
c2: curated gene sets	CALVET IRINOTECAN SENSITIVE VS REVERTED UP	KIRC, LGG	2
c7: immunologic signature gene sets	BUCASAS PBMC FLUARIX FLUVIRIN CAUCASIAN MALE AGE 18 40YO LOW RESPONDERS 1DY POSITIVE PREDICTIVE OF TITER	KIRC, LGG	2
c7: immunologic signature gene sets	BUCASAS PBMC FLUARIX FLUVIRIN CAUCASIAN MALE AGE 18 40YO HIGH RESPONDERS 1DY 3DY POSITIVE PREDICTIVE OF TITER	SKCM, UVM	2
c2: curated gene sets	BOYERINAS ONCOFETAL TARGETS OF LET7A1	KIRC, LGG	2
c2: curated gene sets	BOWIE RESPONSE TO EXTRACELLULAR MATRIX	OV, SKCM	2
c2: curated gene sets	BIOCARTA SUMO PATHWAY	ACC, LIHC	2
c2: curated gene sets	BIOCARTA SKP2E2F PATHWAY	KIRC, LGG	2
c2: curated gene sets	BIOCARTA SAM68 PATHWAY	ACC, KIRC	2
c2: curated gene sets	BIOCARTA RNAPOL3 PATHWAY	ACC, LIHC	2
c2: curated gene sets	BIOCARTA RAN PATHWAY	ACC, KIRC	2
c2: curated gene sets	BIOCARTA PTEN PATHWAY	COAD, KIRC	2
c2: curated gene sets	BIOCARTA PRC2 PATHWAY	LIHC, THCA	2
c2: curated gene sets	BIOCARTA PITX2 PATHWAY	KIRC, THYM	2
c2: curated gene sets	BIOCARTA P53 PATHWAY	KIRC, UCEC	2
c2: curated gene sets	BIOCARTA DNA FRAGMENT PATHWAY	ACC, LIHC	2
c2: curated gene sets	BIOCARTA BLYMPHOCYTE PATHWAY	CESC, SKCM	2
c2: curated gene sets	BIOCARTA ASBCELL PATHWAY	SKCM, UVM	2
c2: curated gene sets	BENPORATH ES CORE NINE	ACC, PAAD	2

c7: immunologic signature gene sets	ANDERSON BLOOD CN54GP140 ADJUVANTED WITH GLA AF AGE 18 45YO LOW IGM RESPONDERS 6HY 1DY UP	PAAD, UVM	2
c3: regulatory target gene sets	ZNF626 TARGET GENES	KIRC	1
c3: regulatory target gene sets	ZNF530 TARGET GENES	ACC	1
c3: regulatory target gene sets	ZNF454 TARGET GENES	THYM	1
c3: regulatory target gene sets	ZNF26 TARGET GENES	UCEC	1
c2: curated gene sets	ZHOU CELL CYCLE GENES IN IR RESPONSE 2HR	MESO	1
c8: cell type signature gene sets	ZHONG PFC MAJOR TYPES EXCITATORY NEURON	CESC	1
c2: curated gene sets	ZHANG INTERFERON RESPONSE	SKCM	1
c2: curated gene sets	ZHAN VARIABLE EARLY DIFFERENTIATION GENES UP	KIRC	1
c2: curated gene sets	ZHAN MULTIPLE MYELOMA MS DN	READ	1
c2: curated gene sets	ZHAN EARLY DIFFERENTIATION GENES UP	SKCM	1
c2: curated gene sets	ZEILSTRA CD44 TARGETS UP	BRCA	1
c2: curated gene sets	ZEILSTRA CD44 TARGETS DN	BRCA	1
c2: curated gene sets	YAO HOXA10 TARGETS VIA PROGESTERONE DN	KIRC	1
c2: curated gene sets	YANG BCL3 TARGETS DN	KIRC	1
c2: curated gene sets	YANAGISAWA LUNG CANCER RECURRENCE	KIRC	1
c2: curated gene sets	XU RESPONSE TO TRETINOIN DN	PRAD	1
c2: curated gene sets	XU RESPONSE TO TRETINOIN AND NSC682994 UP	THCA	1

c2: curated gene sets	XU HGF TARGETS INDUCED BY AKT1 6HR	LGG	1
c2: curated gene sets	XU HGF TARGETS INDUCED BY AKT1 48HR UP	SKCM	1
c2: curated gene sets	XU CREBBP TARGETS UP	THCA	1
c2: curated gene sets	WU HBX TARGETS 3 DN	LUSC	1
c2: curated gene sets	WU ALZHEIMER DISEASE UP	HNSC	1
c2: curated gene sets	WP UREA CYCLE AND ASSOCIATED PATHWAYS	THCA	1
c2: curated gene sets	WP TRANSLATION INHIBITORS IN CHRONICALLY ACTIVATED PDGFRA CELLS	DLBC	1
c2: curated gene sets	WP TRANSCRIPTION FACTOR REGULATION IN ADIPOGENESIS	COAD	1
c2: curated gene sets	WP TP53 NETWORK	SKCM	1
c2: curated gene sets	WP THE HUMAN IMMUNE RESPONSE TO TUBERCULOSIS	THYM	1
c2: curated gene sets	WP TGIF DISRUPTION OF SHH SIGNALING	ACC	1
c2: curated gene sets	WP T CELL RECEPTOR AND COSTIMULATORY SIGNALING	SKCM	1
c2: curated gene sets	WP TCA CYCLE AND DEFICIENCY OF PYRUVATE DEHYDROGENASE COMPLEX PDHC	PRAD	1
c2: curated gene sets	WP SYNTHESIS AND DEGRADATION OF KETONE BODIES	LIHC	1
c2: curated gene sets	WP STRIATED MUSCLE CONTRACTION PATHWAY	PCPG	1
c2: curated gene sets	WP SELECTIVE EXPRESSION OF CHEMOKINE RECEPTORS DURING T CELL POLARIZATION	SKCM	1

c2: curated gene sets	WP SARSCOV2 AND COVID-19 PATHWAY	GBM	1
c2: curated gene sets	WP REGULATION OF SISTER CHROMATID SEPARATION AT THE METAPHASEANAPHASE TRANSITION	ACC	1
c2: curated gene sets	WP PYRIMIDINE METABOLISM AND RELATED DISEASES	MESO	1
c2: curated gene sets	WP PURINE METABOLISM AND RELATED DISORDERS	READ	1
c2: curated gene sets	WP PLATELET MEDIATED INTERACTIONS WITH VASCULAR AND CIRCULATING CELLS	SKCM	1
c2: curated gene sets	WP PEROXIREDOXIN 2 INDUCED OVARIAN FAILURE	LGG	1
c2: curated gene sets	WP PATHWAYS OF NUCLEIC ACID METABOLISM AND INNATE IMMUNE SENSING	KIRC	1
c2: curated gene sets	WP NEUROINFLAMMATION	ACC	1
c2: curated gene sets	WP NANOPARTICLE TRIGGERED REGULATED NECROSIS	LGG	1
c2: curated gene sets	WP MOLYBDENUM COFACTOR MOCO BIOSYNTHESIS	BRCA	1
c2: curated gene sets	WP MIRNA BIOGENESIS	PAAD	1
c2: curated gene sets	WP MIR5093P ALTERATION OF YAP1ECM AXIS	UVM	1
c2: curated gene sets	WP MEVALONATE PATHWAY	CESC	1
c2: curated gene sets	WP MAMMARY GLAND DEVELOPMENT PATHWAY PUBERTY STAGE 2 OF 4	KIRC	1
c2: curated gene sets	WP MAMMARY GLAND DEVELOPMENT PATHWAY INVOLUTION STAGE 4 OF 4	LGG	1

c2: curated gene sets	WP LDLRAD4 AND WHAT WE KNOW ABOUT IT	LUAD	1
c2: curated gene sets	WP IRON METABOLISM IN PLACENTA	KIRC	1
c2: curated gene sets	WP INTERACTOME OF POLYCOMB REPRESSIVE COMPLEX 2 PRC2	KIRC	1
c2: curated gene sets	WP IL5 SIGNALING PATHWAY	DLBC	1
c2: curated gene sets	WP IL10 ANTIINFLAMMATORY SIGNALING PATHWAY	KIRC	1
c2: curated gene sets	WP HYPOTHESIZED PATHWAYS IN PATHOGENESIS OF CARDIOVASCULAR DISEASE	DLBC	1
c2: curated gene sets	WP HOST–PATHOGEN INTERACTION OF HUMAN CORONA VIRUSES MAPK SIGNALING	PCPG	1
c2: curated gene sets	WP HOST–PATHOGEN INTERACTION OF HUMAN CORONA VIRUSES AUTOPHAGY	DLBC	1
c2: curated gene sets	WP HOMOLOGOUS RECOMBINATION	LUAD	1
c2: curated gene sets	WP HIJACK OF UBIQUITINATION BY SARSCOV2	LUSC	1
c2: curated gene sets	WP HIF1A AND PPARG REGULATION OF GLYCOLYSIS	LUAD	1
c2: curated gene sets	WP HEDGEHOG SIGNALING PATHWAY NETPATH	KIRC	1
c2: curated gene sets	WP GLYCOLYSIS IN SENESCENCE	LIHC	1
c2: curated gene sets	WP GLYCEROPHOSPHOLIPID BIOSYNTHETIC PATHWAY	THYM	1
c2: curated gene sets	WP GLUTATHIONE METABOLISM	COAD	1
c2: curated gene sets	WP GASTRIC CANCER NETWORK 1	ACC	1

c2: curated gene sets	WP GAMMAGLUTAMYL CYCLE FOR THE BIOSYNTHESIS AND DEGRADATION OF GLUTATHIONE INCLUDING DISEASES	KIRP	1
c2: curated gene sets	WP FOLATEALCOHOL AND CANCER PATHWAY HYPOTHESES	KIRC	1
c2: curated gene sets	WP FLUOROACETIC ACID TOXICITY	KIRC	1
c2: curated gene sets	WP FATTY ACID BIOSYNTHESIS	THCA	1
c2: curated gene sets	WP EDA SIGNALING IN HAIR FOLLICLE DEVELOPMENT	UCEC	1
c2: curated gene sets	WP DISORDERS OF THE KREBS CYCLE	KIRC	1
c2: curated gene sets	WP DIFFERENTIATION OF WHITE AND BROWN ADIPOCYTE	READ	1
c2: curated gene sets	WP DEREGULATION OF RAB AND RAB EFFECTOR GENES IN BLADDER CANCER	TGCT	1
c2: curated gene sets	WP COVID-19 THROMBOSIS AND ANTICOAGULATION	STAD	1
c2: curated gene sets	WP COVID-19 ADVERSE OUTCOME PATHWAY	SKCM	1
c2: curated gene sets	WP COMMON PATHWAYS UNDERLYING DRUG ADDICTION	READ	1
c2: curated gene sets	WP CELLULAR PROTEOSTASIS	BRCA	1
c2: curated gene sets	WP CANCER IMMUNOTHERAPY BY PD1 BLOCKADE	THYM	1
c2: curated gene sets	WP CANCER IMMUNOTHERAPY BY CTLA4 BLOCKADE	KIRC	1
c2: curated gene sets	WP BIOTIN METABOLISM INCLUDING IEMS	UCEC	1
c2: curated gene sets	WP ATR SIGNALING	KICH	1

c2: curated gene sets	WP AMPLIFICATION AND EXPANSION OF ONCOGENIC PATHWAYS AS METASTATIC TRAITS	THCA	1
c2: curated gene sets	WP ALANINE AND ASPARTATE METABOLISM	THYM	1
c2: curated gene sets	WONG ENDOMETRIAL CANCER LATE	CESC	1
c2: curated gene sets	WILLIAMS ESR1 TARGETS DN	LGG	1
c2: curated gene sets	WENDT COHESIN TARGETS UP	THYM	1
c2: curated gene sets	WEBER METHYLATED LCP IN SPERM DN	KIRP	1
c2: curated gene sets	WEBER METHYLATED HCP IN FIBROBLAST UP	KIRP	1
c2: curated gene sets	WANG TNF TARGETS	PCPG	1
c2: curated gene sets	WANG IMMORTALIZED BY HOXA9 AND MEIS1 UP	THYM	1
c2: curated gene sets	WANG ESOPHAGUS CANCER PROGRESSION UP	KIRC	1
c2: curated gene sets	WANG BARRETTS ESOPHAGUS UP	DLBC	1
c2: curated gene sets	WANG BARRETTS ESOPHAGUS DN	DLBC	1
c2: curated gene sets	WALLACE PROSTATE CANCER DN	LGG	1
c2: curated gene sets	WAESCH ANAPHASE PROMOTING COMPLEX	THYM	1
c2: curated gene sets	VISALA AGING LYMPHOCYTE UP	KIRC	1
c2: curated gene sets	VICENT METASTASIS UP	PCPG	1
c2: curated gene sets	VETTER TARGETS OF PRKCA AND ETS1 DN	UVM	1
c2: curated gene sets	VERRECCHIA RESPONSE TO TGFB1 C6	THCA	1
c2: curated gene sets	VERRECCHIA RESPONSE TO TGFB1 C4	DLBC	1
c2: curated gene sets	VALK AML CLUSTER 10	READ	1

c2: curated gene sets	TURJANSKI MAPK8 AND MAPK9 TARGETS	THYM	1
c2: curated gene sets	TURJANSKI MAPK14 TARGETS	KIRC	1
c2: curated gene sets	TSUDA ALVEOLAR SOFT PART SARCOMA	LGG	1
c8: cell type signature gene sets	TRAVAGLINI LUNG NATURAL KILLER T CELL	THYM	1
c2: curated gene sets	TONKS TARGETS OF RUNX1 RUNX1T1 FUSION SUSTAINED IN MONOCYTE DN	SKCM	1
c2: curated gene sets	TOMLINS METASTASIS UP	KIRC	1
c7: immunologic signature gene sets	THAKAR PBMC INACTIVATED INFLUENZA AGE 70PLS VS 21 30YO 0DY UP	KIRC	1
c3: regulatory target gene sets	TFDP2 TARGET GENES	KIRP	1
c2: curated gene sets	TESAR ALK TARGETS EPISC 3D UP	KIRC	1
c3: regulatory target gene sets	TDRD3 TARGET GENES	LIHC	1
c3: regulatory target gene sets	TATCTGG MIR488	DLBC	1
c2: curated gene sets	TAKADA GASTRIC CANCER COPY NUMBER DN	DLBC	1
c2: curated gene sets	TAGHAVI NEOPLASTIC TRANSFORMATION	KIRC	1
c2: curated gene sets	SUH COEXPRESSED WITH ID1 AND ID2 UP	UCEC	1
c2: curated gene sets	SU THYMUS	THCA	1
c3: regulatory target gene sets	STN1 TARGET GENES	PAAD	1
c2: curated gene sets	STEARMAN TUMOR FIELD EFFECT UP	DLBC	1
c3: regulatory target gene sets	STAT3 01	THYM	1

c2: curated gene sets	SPIRA SMOKERS LUNG CANCER DN	THCA	1
c2: curated gene sets	SOUCEK MYC TARGETS	GBM	1
c7: immunologic signature gene sets	SOBOLEV PBMC PANDEMRIX AGE 18 64YO 7DY DN	THYM	1
c7: immunologic signature gene sets	SOBOLEV PBMC PANDEMRIX AGE 18 64YO 1DY UP	SKCM	1
c2: curated gene sets	SMID BREAST CANCER RELAPSE IN PLEURA DN	THCA	1
c2: curated gene sets	SIMBULAN PARP1 TARGETS DN	LIHC	1
c2: curated gene sets	SILIGAN TARGETS OF EWS FLI1 FUSION DN	KIRC	1
c2: curated gene sets	SHIRAISHI PLZF TARGETS UP	KIRP	1
c2: curated gene sets	SHIN B CELL LYMPHOMA CLUSTER 1	KIRC	1
c2: curated gene sets	SEMBA FHIT TARGETS DN	ACC	1
c2: curated gene sets	SEKI INFLAMMATORY RESPONSE LPS DN	KIRC	1
c2: curated gene sets	SEIDEN MET SIGNALING	COAD	1
c3: regulatory target gene sets	SEF1 C	KIRC	1
c7: immunologic signature gene sets	SCHERER PBMC YF VAX OR APSV WETVAX AGE 18 40YO JOINT TO VACCINIA AND YELLOW FEVER DN	SARC	1
c7: immunologic signature gene sets	SCHERER PBMC APSV WETVAX AGE 18 40YO 5 TO 7DY DN	READ	1
c2: curated gene sets	SCHEIDEREIT IKK TARGETS	READ	1
c2: curated gene sets	SCHAVOLT TARGETS OF TP53 AND TP63	KIRC	1
c2: curated gene sets	SCHAEFFER PROSTATE DEVELOPMENT AND CANCER BOX2 DN	LGG	1

c2: curated gene sets	SCHAEFFER PROSTATE DEVELOPMENT AND CANCER BOX1 UP	BRCA	1
c2: curated gene sets	SASSON FSH RESPONSE	HNSC	1
c2: curated gene sets	SARTIPY NORMAL AT INSULIN RESISTANCE UP	PRAD	1
c2: curated gene sets	SANCHEZ MDM2 TARGETS	THCA	1
c2: curated gene sets	SA TRKA RECEPTOR	PRAD	1
c2: curated gene sets	SA PTEN PATHWAY	KIRC	1
c2: curated gene sets	SA FAS SIGNALING	KIRC	1
c2: curated gene sets	SA CASPASE CASCADE	THYM	1
c2: curated gene sets	RUNNE GENDER EFFECT UP	THCA	1
c2: curated gene sets	RUAN RESPONSE TO TROGLITAZONE UP	THCA	1
c2: curated gene sets	ROZANOV MMP14 TARGETS DN	DLBC	1
c2: curated gene sets	ROZANOV MMP14 CORRELATED	THYM	1
c2: curated gene sets	ROETH TERT TARGETS DN	KIRC	1
c2: curated gene sets	RICKMAN TUMOR DIFFERENTIATED MODERATELY VS POORLY DN	COAD	1
c7: immunologic signature gene sets	RICHERT PBMC HIV LIPO 5 AGE 37 48YO STIMULATED VS UNSTIMULATED 0W 14W METALLOTHIONEIN SUBSET UP	LGG	1
c2: curated gene sets	REN MIF TARGETS DN	KIRP	1
c7: immunologic signature gene sets	RECHTIEN PBMC RVSV ZEBOV AGE 18 55YO 1DY UP	SKCM	1
c2: curated gene sets	REACTOME ZINC INFLUX INTO CELLS BY THE SLC39 GENE FAMILY	READ	1
c2: curated gene sets	REACTOME ZINC EFFLUX AND COMPARTMENTALIZATION BY THE SLC30 FAMILY	COAD	1
c2: curated gene sets	REACTOME VLDLR INTERNALIZATION AND DEGRADATION	DLBC	1

c2: curated gene sets	REACTOME TRNA MODIFICATION IN THE MITOCHONDRION	KIRC	1
c2: curated gene sets	REACTOME TRIF MEDIATED PROGRAMMED CELL DEATH	LUAD	1
c2: curated gene sets	REACTOME TRANSPORT OF NUCLEOTIDE SUGARS	KIRC	1
c2: curated gene sets	REACTOME TRANSCRIPTIONAL REGULATION OF TESTIS DIFFERENTIATION	COAD	1
c2: curated gene sets	REACTOME TRANSCRIPTIONAL REGULATION OF GRANULOPOIESIS	THYM	1
c2: curated gene sets	REACTOME TRANSCRIPTIONAL REGULATION BY E2F6	THYM	1
c2: curated gene sets	REACTOME TRAIL SIGNALING	LGG	1
c2: curated gene sets	REACTOME TRAFFICKING OF GLUR2 CONTAINING AMPA RECEPTORS	TGCT	1
c2: curated gene sets	REACTOME TP53 REGULATES TRANSCRIPTION OF GENES INVOLVED IN G2 CELL CYCLE ARREST	READ	1
c2: curated gene sets	REACTOME TP53 REGULATES TRANSCRIPTION OF GENES INVOLVED IN G1 CELL CYCLE ARREST	KIRC	1
c2: curated gene sets	REACTOME TP53 REGULATES TRANSCRIPTION OF GENES INVOLVED IN CYTOCHROME C RELEASE	KIRC	1
c2: curated gene sets	REACTOME TIE2 SIGNALING	KIRC	1
c2: curated gene sets	REACTOME TFAP2 AP 2 FAMILY REGULATES TRANSCRIPTION OF CELL CYCLE FACTORS	PAAD	1
c2: curated gene sets	REACTOME TERMINATION OF TRANSLESION DNA SYNTHESIS	THYM	1

c2: curated gene sets	REACTOME TBC RABGAPS	DLBC	1
c2: curated gene sets	REACTOME SYNTHESIS OF PIPS AT THE LATE ENDOSOME MEMBRANE	KIRC	1
c2: curated gene sets	REACTOME SYNTHESIS OF PIPS AT THE GOLGI MEMBRANE	READ	1
c2: curated gene sets	REACTOME SYNTHESIS OF PIPS AT THE EARLY ENDOSOME MEMBRANE	READ	1
c2: curated gene sets	REACTOME SYNTHESIS OF LIPOXINS LX	DLBC	1
c2: curated gene sets	REACTOME SYNTHESIS OF GDP MANNOSE	KIRC	1
c2: curated gene sets	REACTOME SYNTHESIS OF BILE ACIDS AND BILE SALTS VIA 24 HYDROXYCHOLESTEROL	DLBC	1
c2: curated gene sets	REACTOME SYNDECAN INTERACTIONS	DLBC	1
c2: curated gene sets	REACTOME SUMO IS TRANSFERRED FROM E1 TO E2 UBE2I UBC9	THYM	1
c2: curated gene sets	REACTOME SUMO IS PROTEOLYTICALLY PROCESSED	UCEC	1
c2: curated gene sets	REACTOME STRIATED MUSCLE CONTRACTION	DLBC	1
c2: curated gene sets	REACTOME SODIUM PROTON EXCHANGERS	KIRC	1
c2: curated gene sets	REACTOME SMAC XIAP REGULATED APOPTOTIC RESPONSE	COAD	1
c2: curated gene sets	REACTOME SLBP DEPENDENT PROCESSING OF REPLICATION DEPENDENT HISTONE PRE MRNAS	KIRP	1

c2: curated gene sets	REACTOME SIGNALING BY PDGFRA TRANSMEMBRANE JUXTAMEMBRANE AND KINASE DOMAIN MUTANTS	KIRC	1
c2: curated gene sets	REACTOME SIGNALING BY PDGFR IN DISEASE	KIRC	1
c2: curated gene sets	REACTOME SIGNALING BY NTRK2 TRKB	DLBC	1
c2: curated gene sets	REACTOME SIGNALING BY NOTCH1 T 7 9 NOTCH1 M1580 K2555 TRANSLOCATION MUTANT	UVM	1
c2: curated gene sets	REACTOME SIGNALING BY NOTCH1 HD DOMAIN MUTANTS IN CANCER	UVM	1
c2: curated gene sets	REACTOME SIGNALING BY MRAS COMPLEX MUTANTS	THYM	1
c2: curated gene sets	REACTOME SIGNALING BY MEMBRANE TETHERED FUSIONS OF PDGFRA OR PDGFRB	LGG	1
c2: curated gene sets	REACTOME SIGNALING BY MAPK MUTANTS	LGG	1
c2: curated gene sets	REACTOME SIGNALING BY LEPTIN	UVM	1
c2: curated gene sets	REACTOME SIGNALING BY KIT IN DISEASE	KIRC	1
c2: curated gene sets	REACTOME SIGNALING BY FGFR4 IN DISEASE	KIRC	1
c2: curated gene sets	REACTOME SIGNALING BY FGFR3 FUSIONS IN CANCER	KIRC	1
c2: curated gene sets	REACTOME SIGNALING BY ERBB4	DLBC	1
c2: curated gene sets	REACTOME SIGNALING BY ERBB2 ECD MUTANTS	KIRC	1
c2: curated gene sets	REACTOME SIGNALING BY EGFR	DLBC	1

c2: curated gene sets	REACTOME SIGNALING BY CYTOSOLIC FGFR1 FUSION MUTANTS	THYM	1
c2: curated gene sets	REACTOME SIGNALING BY CTNNB1 PHOSPHO SITE MUTANTS	KIRC	1
c2: curated gene sets	REACTOME SHC1 EVENTS IN EGFR SIGNALING	PAAD	1
c2: curated gene sets	REACTOME RUNX2 REGULATES GENES INVOLVED IN CELL MIGRATION	LGG	1
c2: curated gene sets	REACTOME RUNX1 REGULATES TRANSCRIPTION OF GENES INVOLVED IN DIFFERENTIATION OF MYELOID CELLS	THYM	1
c2: curated gene sets	REACTOME RUNX1 REGULATES TRANSCRIPTION OF GENES INVOLVED IN BCR SIGNALING	KIRC	1
c2: curated gene sets	REACTOME RUNX1 REGULATES ESTROGEN RECEPTOR MEDIATED TRANSCRIPTION	LIHC	1
c2: curated gene sets	REACTOME RUNX1 AND FOXP3 CONTROL THE DEVELOPMENT OF REGULATORY T LYMPHOCYTES TREGS	GBM	1
c2: curated gene sets	REACTOME RRNA PROCESSING IN THE MITOCHONDRION	KICH	1
c2: curated gene sets	REACTOME ROS AND RNS PRODUCTION IN PHAGOCYTES	DLBC	1
c2: curated gene sets	REACTOME RNA POLYMERASE III TRANSCRIPTION INITIATION FROM TYPE 1 PROMOTER	LIHC	1
c2: curated gene sets	REACTOME RNA POLYMERASE I PROMOTER ESCAPE	THCA	1

c2: curated gene sets	REACTOME RHOBTB3 ATPASE CYCLE	SARC	1
c2: curated gene sets	REACTOME RHOBTB1 GTPASE CYCLE	COAD	1
c2: curated gene sets	REACTOME RHO GTPASES ACTIVATE RHOTEKIN AND RHOPHILINS	KIRC	1
c2: curated gene sets	REACTOME RESPONSE OF EIF2AK1 HRI TO HEME DEFICIENCY	THYM	1
c2: curated gene sets	REACTOME REGULATION OF SIGNALING BY CBL	PRAD	1
c2: curated gene sets	REACTOME REGULATION OF LOCALIZATION OF FOXO TRANSCRIPTION FACTORS	KIRC	1
c2: curated gene sets	REACTOME REGULATION OF KIT SIGNALING	KIRC	1
c2: curated gene sets	REACTOME REGULATION OF INNATE IMMUNE RESPONSES TO CYTOSOLIC DNA	SKCM	1
c2: curated gene sets	REACTOME REGULATION OF FOXO TRANSCRIPTIONAL ACTIVITY BY ACETYLATION	KIRC	1
c2: curated gene sets	REACTOME REGULATION OF CYTOSKELETAL REMODELING AND CELL SPREADING BY IPP COMPLEX COMPONENTS	ACC	1
c2: curated gene sets	REACTOME REGULATION BY C FLIP	LGG	1
c2: curated gene sets	REACTOME REGULATED PROTEOLYSIS OF P75NTR	KIRP	1
c2: curated gene sets	REACTOME REELIN SIGNALING PATHWAY	PAAD	1

c2: curated gene sets	REACTOME RECYCLING OF EIF2 GDP	LIHC	1
c2: curated gene sets	REACTOME RECEPTOR MEDIATED MITOPHAGY	KICH	1
c2: curated gene sets	REACTOME REACTIONS SPECIFIC TO THE COMPLEX N GLYCAN SYNTHESIS PATHWAY	PAAD	1
c2: curated gene sets	REACTOME RAS SIGNALING DOWNSTREAM OF NF1 LOSS OF FUNCTION VARIANTS	KIRC	1
c2: curated gene sets	REACTOME PYROPTOSIS	THYM	1
c2: curated gene sets	REACTOME PTK6 REGULATES RHO GTPASES RAS GTPASE AND MAP KINASES	KIRC	1
c2: curated gene sets	REACTOME PTK6 REGULATES PROTEINS INVOLVED IN RNA PROCESSING	ACC	1
c2: curated gene sets	REACTOME PTK6 REGULATES CELL CYCLE	KIRC	1
c2: curated gene sets	REACTOME PROTEIN REPAIR	KIRP	1
c2: curated gene sets	REACTOME PROTEIN METHYLATION	UVM	1
c2: curated gene sets	REACTOME PROPIONYL COA CATABOLISM	KIRP	1
c2: curated gene sets	REACTOME PROCESSIVE SYNTHESIS ON THE LAGGING STRAND	LGG	1
c2: curated gene sets	REACTOME PROCESSIVE SYNTHESIS ON THE C STRAND OF THE TELOMERE	LGG	1
c2: curated gene sets	REACTOME PROCESSING AND ACTIVATION OF SUMO	THYM	1

c2: curated gene sets	REACTOME PREVENTION OF PHAGOSOMAL LYSOSOMAL FUSION	UVM	1
c2: curated gene sets	REACTOME POLYMERASE SWITCHING	LGG	1
c2: curated gene sets	REACTOME POLO LIKE KINASE MEDIATED EVENTS	LIHC	1
c2: curated gene sets	REACTOME PLASMA LIPOPROTEIN CLEARANCE	DLBC	1
c2: curated gene sets	REACTOME PI5P REGULATES TP53 ACETYLATION	READ	1
c2: curated gene sets	REACTOME PI3K EVENTS IN ERBB2 SIGNALING	THYM	1
c2: curated gene sets	REACTOME PHASE 3 RAPID REPOLARIZATION	BRCA	1
c2: curated gene sets	REACTOME PEXOPHAGY	PRAD	1
c2: curated gene sets	REACTOME PEROXISOMAL LIPID METABOLISM	DLBC	1
c2: curated gene sets	REACTOME PEPTIDE HORMONE BIOSYNTHESIS	PAAD	1
c2: curated gene sets	REACTOME ORGANIC CATION TRANSPORT	UCEC	1
c2: curated gene sets	REACTOME ORGANIC CATION ANION ZWITTERION TRANSPORT	UCEC	1
c2: curated gene sets	REACTOME OAS ANTIVIRAL RESPONSE	KIRC	1
c2: curated gene sets	REACTOME NUCLEAR SIGNALING BY ERBB4	DLBC	1
c2: curated gene sets	REACTOME NTRK2 ACTIVATES RAC1	KIRC	1
c2: curated gene sets	REACTOME NRIF SIGNALS CELL DEATH FROM THE NUCLEUS	KIRP	1

c2: curated gene sets	REACTOME NRCAM INTERACTIONS	KIRP	1
c2: curated gene sets	REACTOME NR1H2 NR1H3 REGULATE GENE EXPRESSION LINKED TO TRIGLYCERIDE LIPOLYSIS IN ADIPOSE	COAD	1
c2: curated gene sets	REACTOME NOTCH4 ACTIVATION AND TRANSMISSION OF SIGNAL TO THE NUCLEUS	KIRC	1
c2: curated gene sets	REACTOME NOTCH2 INTRACELLULAR DOMAIN REGULATES TRANSCRIPTION	UCEC	1
c2: curated gene sets	REACTOME NOTCH2 ACTIVATION AND TRANSMISSION OF SIGNAL TO THE NUCLEUS	PRAD	1
c2: curated gene sets	REACTOME NONCANONICAL ACTIVATION OF NOTCH3	SKCM	1
c2: curated gene sets	REACTOME NITRIC OXIDE STIMULATES GUANYLATE CYCLASE	PCPG	1
c2: curated gene sets	REACTOME NEUROTRANSMITTER CLEARANCE	PCPG	1
c2: curated gene sets	REACTOME NEUROTOXICITY OF CLOSTRIDIUM TOXINS	KIRP	1
c2: curated gene sets	REACTOME NEUROFASCIN INTERACTIONS	LUAD	1
c2: curated gene sets	REACTOME NEUREXINS AND NEUROLIGINS	DLBC	1
c2: curated gene sets	REACTOME NETRIN MEDIATED REPULSION SIGNALS	MESO	1
c2: curated gene sets	REACTOME NEGATIVE REGULATION OF FLT3	UVM	1

c2: curated gene sets	REACTOME NEGATIVE FEEDBACK REGULATION OF MAPK PATHWAY	LIHC	1
c2: curated gene sets	REACTOME NEF MEDIATED DOWNREGULATION OF MHC CLASS I COMPLEX CELL SURFACE EXPRESSION	THYM	1
c2: curated gene sets	REACTOME NEF MEDIATED CD8 DOWN REGULATION	KIRC	1
c2: curated gene sets	REACTOME MYOGENESIS	TGCT	1
c2: curated gene sets	REACTOME MULTIFUNCTIONAL ANION EXCHANGERS	HNSC	1
c2: curated gene sets	REACTOME MUCOPOLYSACCHARIDOSES	PRAD	1
c2: curated gene sets	REACTOME MRNA EDITING C TO U CONVERSION	CESC	1
c2: curated gene sets	REACTOME MOLYBDENUM COFACTOR BIOSYNTHESIS	PAAD	1
c2: curated gene sets	REACTOME MITOTIC TELOPHASE CYTOKINESIS	THYM	1
c2: curated gene sets	REACTOME MITOCHONDRIAL UNCOUPLING	CESC	1
c2: curated gene sets	REACTOME MITOCHONDRIAL FATTY ACID BETA OXIDATION OF UNSATURATED FATTY ACIDS	KIRP	1
c2: curated gene sets	REACTOME MITOCHONDRIAL FATTY ACID BETA OXIDATION OF SATURATED FATTY ACIDS	KIRP	1
c2: curated gene sets	REACTOME MISMATCH REPAIR	LGG	1
c2: curated gene sets	REACTOME METAL ION SLC TRANSPORTERS	COAD	1

c2: curated gene sets	REACTOME METABOLISM OF INGESTED SEMET SEC MESEC INTO H2SE	SARC	1
c2: curated gene sets	REACTOME METABOLISM OF FOLATE AND PTERINES	KIRP	1
c2: curated gene sets	REACTOME MET ACTIVATES RAS SIGNALING	READ	1
c2: curated gene sets	REACTOME MET ACTIVATES PTPN11	READ	1
c2: curated gene sets	REACTOME MET ACTIVATES PI3K AKT SIGNALING	GBM	1
c2: curated gene sets	REACTOME MATURATION OF SARS COV 1 SPIKE PROTEIN	LGG	1
c2: curated gene sets	REACTOME MATURATION OF NUCLEOPROTEIN	SKCM	1
c2: curated gene sets	REACTOME MAPK1 ERK2 ACTIVATION	KIRC	1
c2: curated gene sets	REACTOME LYSINE CATABOLISM	KIRC	1
c2: curated gene sets	REACTOME LTC4 CYSLTR MEDIATED IL4 PRODUCTION	KIRP	1
c2: curated gene sets	REACTOME LIGAND RECEPTOR INTERACTIONS	UCEC	1
c2: curated gene sets	REACTOME LEUKOTRIENE RECEPTORS	UVM	1
c2: curated gene sets	REACTOME KERATAN SULFATE DEGRADATION	DLBC	1
c2: curated gene sets	REACTOME JNK C JUN KINASES PHOSPHORYLATION AND ACTIVATION MEDIATED BY ACTIVATED HUMAN TAK1	THYM	1
c2: curated gene sets	REACTOME IRON UPTAKE AND TRANSPORT	DLBC	1

c2: curated gene sets	REACTOME IRF3 MEDIATED INDUCTION OF TYPE I IFN	PCPG	1
c2: curated gene sets	REACTOME IRF3 MEDIATED ACTIVATION OF TYPE 1 IFN	SKCM	1
c2: curated gene sets	REACTOME INTESTINAL ABSORPTION	ESCA	1
c2: curated gene sets	REACTOME INTERLEUKIN 6 SIGNALING	KIRC	1
c2: curated gene sets	REACTOME INTERLEUKIN 36 PATHWAY	KIRP	1
c2: curated gene sets	REACTOME INTERLEUKIN 35 SIGNALING	SKCM	1
c2: curated gene sets	REACTOME INTERLEUKIN 21 SIGNALING	BRCA	1
c2: curated gene sets	REACTOME INTEGRATION OF PROVIRUS	KIRC	1
c2: curated gene sets	REACTOME INSERTION OF TAIL ANCHORED PROTEINS INTO THE ENDOPLASMIC RETICULUM MEMBRANE	UCEC	1
c2: curated gene sets	REACTOME INITIATION OF NUCLEAR ENVELOPE NE REFORMATION	LIHC	1
c2: curated gene sets	REACTOME INHIBITION OF REPLICATION INITIATION OF DAMAGED DNA BY RB1 E2F1	LIHC	1
c2: curated gene sets	REACTOME INACTIVATION OF CSF3 G CSF SIGNALING	BRCA	1
c2: curated gene sets	REACTOME INACTIVATION OF CDC42 AND RAC1	KIRP	1
c2: curated gene sets	REACTOME HS GAG DEGRADATION	DLBC	1

c2: curated gene sets	REACTOME HIV ELONGATION ARREST AND RECOVERY	THYM	1
c2: curated gene sets	REACTOME HDR THROUGH MMEJ ALT NHEJ	LIHC	1
c2: curated gene sets	REACTOME HDMS DEMETHYLATE HISTONES	PRAD	1
c2: curated gene sets	REACTOME GRB7 EVENTS IN ERBB2 SIGNALING	PCPG	1
c2: curated gene sets	REACTOME GLUTAMATE AND GLUTAMINE METABOLISM	BRCA	1
c2: curated gene sets	REACTOME GALACTOSE CATABOLISM	COAD	1
c2: curated gene sets	REACTOME FRUCTOSE METABOLISM	GBM	1
c2: curated gene sets	REACTOME FOXO MEDIATED TRANSCRIPTION OF OXIDATIVE STRESS METABOLIC AND NEURONAL GENES	TGCT	1
c2: curated gene sets	REACTOME FOXO MEDIATED TRANSCRIPTION OF CELL DEATH GENES	KIRC	1
c2: curated gene sets	REACTOME FORMATION OF SENESCENCE ASSOCIATED HETEROCHROMATIN FOCI SAHF	PAAD	1
c2: curated gene sets	REACTOME FOLDING OF ACTIN BY CCT TRIC	LIHC	1
c2: curated gene sets	REACTOME FIBRONECTIN MATRIX FORMATION	CESC	1
c2: curated gene sets	REACTOME FGFR1B LIGAND BINDING AND ACTIVATION	STAD	1

c2: curated gene sets	REACTOME FATTY ACIDS BOUND TO GPR40 FFAR1 REGULATE INSULIN SECRETION	KIRC	1
c2: curated gene sets	REACTOME ESTROGEN STIMULATED SIGNALING THROUGH PRKCZ	KIRC	1
c2: curated gene sets	REACTOME ESTROGEN BIOSYNTHESIS	BLCA	1
c2: curated gene sets	REACTOME ESTABLISHMENT OF SISTER CHROMATID COHESION	KICH	1
c2: curated gene sets	REACTOME ERYTHROPOIETIN ACTIVATES PHOSPHOINOSITIDE 3 KINASE PI3K	LUAD	1
c2: curated gene sets	REACTOME ERBB2 REGULATES CELL MOTILITY	THYM	1
c2: curated gene sets	REACTOME ENOS ACTIVATION	BLCA	1
c2: curated gene sets	REACTOME ENDOSOMAL VACUOLAR PATHWAY	THCA	1
c2: curated gene sets	REACTOME EGR2 AND SOX10 MEDIATED INITIATION OF SCHWANN CELL MYELINATION	DLBC	1
c2: curated gene sets	REACTOME EGFR INTERACTS WITH PHOSPHOLIPASE C GAMMA	PAAD	1
c2: curated gene sets	REACTOME EARLY PHASE OF HIV LIFE CYCLE	KIRC	1
c2: curated gene sets	REACTOME DOWNREGULATION OF ERBB4 SIGNALING	UVM	1
c2: curated gene sets	REACTOME DISINHIBITION OF SNARE FORMATION	CESC	1
c2: curated gene sets	REACTOME DISEASES OF DNA REPAIR	KICH	1

c2: curated gene sets	REACTOME DISEASES ASSOCIATED WITH N GLYCOSYLATION OF PROTEINS	UVM	1
c2: curated gene sets	REACTOME DEFECTS IN BIOTIN BTN METABOLISM	UCEC	1
c2: curated gene sets	REACTOME DEFECTIVE CSF2RB CAUSES SMDP5	BRCA	1
c2: curated gene sets	REACTOME DEFECTIVE B4GALT1 CAUSES B4GALT1 CDG CDG 2D	LUAD	1
c2: curated gene sets	REACTOME CYTOSOLIC IRON SULFUR CLUSTER ASSEMBLY	COAD	1
c2: curated gene sets	REACTOME CYCLIN A B1 B2 ASSOCIATED EVENTS DURING G2 M TRANSITION	LIHC	1
c2: curated gene sets	REACTOME CTLA4 INHIBITORY SIGNALING	KIRC	1
c2: curated gene sets	REACTOME CREB3 FACTORS ACTIVATE GENES	UCEC	1
c2: curated gene sets	REACTOME CREB1 PHOSPHORYLATION THROUGH THE ACTIVATION OF CAMKII CAMKK CAMKIV CASCADE	LIHC	1
c2: curated gene sets	REACTOME CONDENSATION OF PROPHASE CHROMOSOMES	LIHC	1
c2: curated gene sets	REACTOME COHESIN LOADING ONTO CHROMATIN	THYM	1
c2: curated gene sets	REACTOME CLASS I PEROXISOMAL MEMBRANE PROTEIN IMPORT	KIRC	1
c2: curated gene sets	REACTOME CHOLINE CATABOLISM	KIRC	1
c2: curated gene sets	REACTOME CELL EXTRACELLULAR MATRIX INTERACTIONS	THYM	1

c2: curated gene sets	REACTOME CD28 CO STIMULATION	THYM	1
c2: curated gene sets	REACTOME CD209 DC SIGN SIGNALING	READ	1
c2: curated gene sets	REACTOME CALCINEURIN ACTIVATES NFAT	LGG	1
c2: curated gene sets	REACTOME BUDDING AND MATURATION OF HIV VIRION	PRAD	1
c2: curated gene sets	REACTOME BETA OXIDATION OF PRISTANOYL COA	KIRC	1
c2: curated gene sets	REACTOME BETA OXIDATION OF OCTANOYL COA TO HEXANOYL COA	KIRP	1
c2: curated gene sets	REACTOME BETA OXIDATION OF DECANOYL COA TO OCTANOYL COA COA	KIRP	1
c2: curated gene sets	REACTOME BETA OXIDATION OF BUTANOYL COA TO ACETYL COA	BLCA	1
c2: curated gene sets	REACTOME ATF6 ATF6 ALPHA ACTIVATES CHAPERONE GENES	KIRP	1
c2: curated gene sets	REACTOME ARACHIDONATE PRODUCTION FROM DAG	UVM	1
c2: curated gene sets	REACTOME APOPTOTIC CLEAVAGE OF CELLULAR PROTEINS	DLBC	1
c2: curated gene sets	REACTOME APOBEC3G MEDIATED RESISTANCE TO HIV 1 INFECTION	KIRC	1
c2: curated gene sets	REACTOME APC CDC20 MEDIATED DEGRADATION OF NEK2A	READ	1
c2: curated gene sets	REACTOME AMINO ACID CONJUGATION	READ	1

c2: curated gene sets	REACTOME ALPHA OXIDATION OF PHYTANATE	KIRC	1
c2: curated gene sets	REACTOME ACTIVATION OF THE TFAP2 AP 2 FAMILY OF TRANSCRIPTION FACTORS	BRCA	1
c2: curated gene sets	REACTOME ACTIVATION OF THE PHOTOTRANSDUCTION CASCADE	BRCA	1
c2: curated gene sets	REACTOME ACTIVATION OF THE AP 1 FAMILY OF TRANSCRIPTION FACTORS	PCPG	1
c2: curated gene sets	REACTOME ACTIVATION OF SMO	KIRC	1
c2: curated gene sets	REACTOME ACTIVATION OF RAC1 DOWNSTREAM OF NMDARS	PAAD	1
c2: curated gene sets	REACTOME ACTIVATION OF PUMA AND TRANSLOCATION TO MITOCHONDRIA	KIRC	1
c2: curated gene sets	REACTOME ACTIVATION OF CASPASES THROUGH APOPTOSOME MEDIATED CLEAVAGE	COAD	1
c2: curated gene sets	REACTOME ACTIVATION OF BAD AND TRANSLOCATION TO MITOCHONDRIA	BRCA	1
c2: curated gene sets	REACTOME ACTIVATED NTRK3 SIGNALS THROUGH RAS	ACC	1
c2: curated gene sets	REACTOME ACTIVATED NTRK3 SIGNALS THROUGH PI3K	ACC	1
c2: curated gene sets	REACTOME ACTIVATED NTRK2 SIGNALS THROUGH RAS	KIRC	1
c2: curated gene sets	REACTOME ACTIVATED NTRK2 SIGNALS THROUGH FYN	KIRC	1
c2: curated gene sets	REACTOME ACTIVATED NTRK2 SIGNALS THROUGH FRS2 AND FRS3	KIRC	1

c2: curated gene sets	REACTOME ACETYLCHOLINE REGULATES INSULIN SECRETION	KIRC	1
c2: curated gene sets	REACTOME ABERRANT REGULATION OF MITOTIC G1 S TRANSITION IN CANCER DUE TO RB1 DEFECTS	KIRC	1
c2: curated gene sets	REACTOME ABC TRANSPORTERS IN LIPID HOMEOSTASIS	BRCA	1
c2: curated gene sets	REACTOME 2 LTR CIRCLE FORMATION	KIRC	1
c2: curated gene sets	POS RESPONSE TO HISTAMINE DN	KIRC	1
c2: curated gene sets	PLASARI TGFB1 TARGETS 1HR DN	THCA	1
c2: curated gene sets	PLASARI NFIC TARGETS BASAL DN	COAD	1
c2: curated gene sets	PID WNT SIGNALING PATHWAY	TGCT	1
c2: curated gene sets	PID THROMBIN PAR4 PATHWAY	KIRC	1
c2: curated gene sets	PID SYNDECAN 4 PATHWAY	DLBC	1
c2: curated gene sets	PID SYNDECAN 2 PATHWAY	DLBC	1
c2: curated gene sets	PID SYNDECAN 1 PATHWAY	DLBC	1
c2: curated gene sets	PID SMAD2 3PATHWAY	THYM	1
c2: curated gene sets	PID P38 MKK3 6PATHWAY	THYM	1
c2: curated gene sets	PID LIS1 PATHWAY	DLBC	1
c2: curated gene sets	PID INTEGRIN5 PATHWAY	LGG	1
c2: curated gene sets	PID INTEGRIN2 PATHWAY	BRCA	1
c2: curated gene sets	PID INSULIN PATHWAY	DLBC	1
c2: curated gene sets	PID IL27 PATHWAY	SKCM	1
c2: curated gene sets	PID ERBB1 RECEPTOR PROXIMAL PATHWAY	READ	1
c2: curated gene sets	PID EPHB FWD PATHWAY	DLBC	1
c2: curated gene sets	PID ECADHERIN KERATINOCYTE PATHWAY	KIRC	1
c2: curated gene sets	PID AVB3 OPN PATHWAY	DLBC	1

c2: curated gene sets	PID AR NONGENOMIC PATHWAY	DLBC	1
c2: curated gene sets	PHONG TNF TARGETS DN	ACC	1
c2: curated gene sets	PETRETTO BLOOD PRESSURE DN	KIRC	1
c7: immunologic signature gene sets	PATEL SKIN OF BODY ZOSTAVAX AGE 70 93YO VZV CHALLENGED POST VACCINATION VS UNCHALLENGED 72HR TOP 30 DEG UP	SKCM	1
c2: curated gene sets	PARK OSTEOBLAST DIFFERENTIATION BY PHENYLAMIL DN	PAAD	1
c7: immunologic signature gene sets	PANAPASA BLOOD FLUENZ AGE 03 17YO 3DY 4DY DN	THYM	1
c2: curated gene sets	PALOMERO GSI SENSITIVITY UP	PRAD	1
c2: curated gene sets	PAL PRMT5 TARGETS DN	THYM	1
c2: curated gene sets	ONO FOXP3 TARGETS UP	KIRC	1
c3: regulatory target gene sets	NUP214 TARGET GENES	READ	1
c2: curated gene sets	NUNODA RESPONSE TO DASATINIB IMATINIB DN	THCA	1
c3: regulatory target gene sets	NUFIP1 TARGET GENES	READ	1
c3: regulatory target gene sets	NR2E3 TARGET GENES	BLCA	1
c2: curated gene sets	NIKOLSKY BREAST CANCER 7P15 AMPLICON	LGG	1
c2: curated gene sets	NIKOLSKY BREAST CANCER 1Q32 AMPLICON	KIRP	1
c2: curated gene sets	NIKOLSKY BREAST CANCER 19P13 AMPLICON	KIRC	1
c2: curated gene sets	NIKOLSKY BREAST CANCER 17P11 AMPLICON	KIRP	1
c2: curated gene sets	NICK RESPONSE TO PROC TREATMENT UP	BRCA	1

c7: immunologic signature gene sets	NAKAYA PBMC FLUMIST AGE 18 50YO 7DY IFN SUBSET DN	KIRC	1
c2: curated gene sets	NAKAMURA LUNG CANCER	UCEC	1
c2: curated gene sets	NAKAMURA BRONCHIAL AND BRONCHIOLAR EPITHELIA	KIRP	1
c2: curated gene sets	NAKAMURA ALVEOLAR EPITHELIUM	UVM	1
c2: curated gene sets	NADELLA PRKAR1A TARGETS DN	KIRP	1
c2: curated gene sets	MYLLYKANGAS AMPLIFICATION HOT SPOT 9	THYM	1
c2: curated gene sets	MYLLYKANGAS AMPLIFICATION HOT SPOT 30	MESO	1
c2: curated gene sets	MYLLYKANGAS AMPLIFICATION HOT SPOT 27	UCEC	1
c2: curated gene sets	MYLLYKANGAS AMPLIFICATION HOT SPOT 24	KIRP	1
c2: curated gene sets	MYLLYKANGAS AMPLIFICATION HOT SPOT 16	UVM	1
c2: curated gene sets	MURAKAMI UV RESPONSE 24HR	TGCT	1
c2: curated gene sets	MORI PLASMA CELL DN	COAD	1
c2: curated gene sets	MORI EMU MYC LYMPHOMA BY ONSET TIME DN	LGG	1
c4: computational gene sets	MORF RAB11A	DLBC	1
c2: curated gene sets	MOOTHA TCA	PRAD	1
c2: curated gene sets	MOOTHA GLYCOLYSIS	THCA	1
c4: computational gene sets	MODULE 78	THCA	1
c4: computational gene sets	MODULE 562	DLBC	1
c4: computational gene sets	MODULE 552	ACC	1
c4: computational gene sets	MODULE 540	LGG	1

c4: computational gene sets	MODULE 528	CHOL	1
c4: computational gene sets	MODULE 516	READ	1
c4: computational gene sets	MODULE 500	READ	1
c4: computational gene sets	MODULE 495	PAAD	1
c4: computational gene sets	MODULE 471	CHOL	1
c4: computational gene sets	MODULE 458	PRAD	1
c4: computational gene sets	MODULE 387	DLBC	1
c4: computational gene sets	MODULE 381	LGG	1
c4: computational gene sets	MODULE 352	THYM	1
c4: computational gene sets	MODULE 333	DLBC	1
c4: computational gene sets	MODULE 329	DLBC	1
c4: computational gene sets	MODULE 326	BRCA	1
c4: computational gene sets	MODULE 284	READ	1
c4: computational gene sets	MODULE 263	PCPG	1
c4: computational gene sets	MODULE 262	SKCM	1
c4: computational gene sets	MODULE 222	LIHC	1
c4: computational gene sets	MODULE 211	DLBC	1
c4: computational gene sets	MODULE 21	KIRC	1
c4: computational gene sets	MODULE 203	ACC	1

c4: computational gene sets	MODULE 183	TGCT	1
c4: computational gene sets	MODULE 168	COAD	1
c4: computational gene sets	MODULE 110	ACC	1
c3: regulatory target gene sets	MIR92B 5P	ACC	1
c3: regulatory target gene sets	MIR8078	READ	1
c3: regulatory target gene sets	MIR8074	KIRC	1
c3: regulatory target gene sets	MIR8053	KIRP	1
c3: regulatory target gene sets	MIR6871 5P	DLBC	1
c3: regulatory target gene sets	MIR6853 5P	COAD	1
c3: regulatory target gene sets	MIR6808 3P	DLBC	1
c3: regulatory target gene sets	MIR6800 5P	READ	1
c3: regulatory target gene sets	MIR6788 3P	COAD	1
c3: regulatory target gene sets	MIR6775 5P	LGG	1
c3: regulatory target gene sets	MIR6746 5P	KIRC	1
c3: regulatory target gene sets	MIR6738 5P	DLBC	1
c3: regulatory target gene sets	MIR662	SKCM	1
c3: regulatory target gene sets	MIR6506 3P	LUAD	1
c3: regulatory target gene sets	MIR6089	MESO	1
c3: regulatory target gene sets	MIR604	ACC	1

c3: regulatory target gene sets	MIR602	KIRC	1
c3: regulatory target gene sets	MIR598 5P	KIRP	1
c3: regulatory target gene sets	MIR5681B	DLBC	1
c3: regulatory target gene sets	MIR554	BRCA	1
c3: regulatory target gene sets	MIR548AL	KIRC	1
c3: regulatory target gene sets	MIR520G 5P	DLBC	1
c3: regulatory target gene sets	MIR4804 5P	KIRC	1
c3: regulatory target gene sets	MIR4781 5P	BRCA	1
c3: regulatory target gene sets	MIR4749 5P	LUSC	1
c3: regulatory target gene sets	MIR4740 3P	HNSC	1
c3: regulatory target gene sets	MIR4681	UCEC	1
c3: regulatory target gene sets	MIR4675	READ	1
c3: regulatory target gene sets	MIR451A	SKCM	1
c3: regulatory target gene sets	MIR4506	DLBC	1
c3: regulatory target gene sets	MIR4497	THYM	1
c3: regulatory target gene sets	MIR4480	DLBC	1
c3: regulatory target gene sets	MIR4445 3P	KIRC	1
c3: regulatory target gene sets	MIR4433B 3P	TGCT	1
c3: regulatory target gene sets	MIR4315	LIHC	1

c3: regulatory target gene sets	MIR3960 MIR8072	UVM	1
c3: regulatory target gene sets	MIR371A 3P	UCEC	1
c3: regulatory target gene sets	MIR3683	UVM	1
c3: regulatory target gene sets	MIR3651	THCA	1
c3: regulatory target gene sets	MIR339 3P	KIRC	1
c3: regulatory target gene sets	MIR3177 3P	READ	1
c3: regulatory target gene sets	MIR25 5P	LGG	1
c3: regulatory target gene sets	MIR1973	UCS	1
c3: regulatory target gene sets	MIR1539	MESO	1
c3: regulatory target gene sets	MIR147B 3P	MESO	1
c3: regulatory target gene sets	MIR1470	UVM	1
c3: regulatory target gene sets	MIR1468 5P	THYM	1
c3: regulatory target gene sets	MIR139 3P	KIRC	1
c3: regulatory target gene sets	MIR1277 3P	THYM	1
c3: regulatory target gene sets	MIR1250 5P	LGG	1
c3: regulatory target gene sets	MIR1238 3P	DLBC	1
c3: regulatory target gene sets	MIR122 3P	KIRC	1
c3: regulatory target gene sets	MIR12126	DLBC	1
c3: regulatory target gene sets	MIR1203	UCEC	1

c3: regulatory target gene sets	MIR105 3P	HNSC	1
c3: regulatory target gene sets	MIR10392 3P	UCEC	1
c2: curated gene sets	MINGUEZ LIVER CANCER VASCULAR INVASION DN	LIHC	1
c2: curated gene sets	MILICIC FAMILIAL ADENOMATOUS POLYPOSIS DN	UCEC	1
c2: curated gene sets	MIKKELSEN DEDIFFERENTIATED STATE UP	THYM	1
c2: curated gene sets	MEINHOLD OVARIAN CANCER LOW GRADE UP	KIRC	1
c2: curated gene sets	MEINHOLD OVARIAN CANCER LOW GRADE DN	ACC	1
c2: curated gene sets	MEBARKI HCC PROGENITOR WNT UP CTNNB1 INDEPENDENT	KIRC	1
c3: regulatory target gene sets	MDM2 TARGET GENES	THYM	1
c2: curated gene sets	MCCLUNG CREB1 TARGETS DN	DLBC	1
c2: curated gene sets	MCBRYAN TERMINAL END BUD DN	DLBC	1
c2: curated gene sets	MATZUK POSTIMPLANTATION AND POSTPARTUM	READ	1
c2: curated gene sets	MATZUK LUTEAL GENES	COAD	1
c7: immunologic signature gene sets	MATSUMIYA PBMC MODIFIED VACCINIA ANKARA VACCINE AGE 18 55YO LOW VS HIGH RESPONDERS 2DY GO T CELL ACTIVE AND CO STIM UP	KIRC	1
c2: curated gene sets	MATHEW FANCONI ANEMIA GENES	LIHC	1
c2: curated gene sets	MARSON FOXP3 TARGETS STIMULATED DN	KIRC	1

c2: curated gene sets	MARIADASON RESPONSE TO BUTYRATE CURCUMIN SULINDAC TSA 2	HNSC	1
c2: curated gene sets	MANN RESPONSE TO AMIFOSTINE DN	LIHC	1
c2: curated gene sets	MAHADEVAN IMATINIB RESISTANCE UP	KIRC	1
c2: curated gene sets	MACLACHLAN BRCA1 TARGETS UP	UVM	1
c2: curated gene sets	LUND SILENCED BY METHYLATION	THYM	1
c2: curated gene sets	LUI THYROID CANCER CLUSTER 5	CESC	1
c2: curated gene sets	LU TUMOR VASCULATURE DN	DLBC	1
c2: curated gene sets	LOPEZ MESOTHELIOMA SURVIVAL OVERALL UP	BRCA	1
c2: curated gene sets	LOPEZ MESOTELIOMA SURVIVAL TIME DN	BRCA	1
c2: curated gene sets	LIU IL13 MEMORY MODEL UP	SARC	1
c2: curated gene sets	LIEN BREAST CARCINOMA METAPLASTIC	PCPG	1
c2: curated gene sets	LI WILMS TUMOR ANAPLASTIC DN	HNSC	1
c7: immunologic signature gene sets	LI PBMC ZOSTAVAX AGE 25 40 AND 60 79YO 1DY UP	SARC	1
c2: curated gene sets	LI ADIPOGENESIS BY ACTIVATED PPARG	DLBC	1
c2: curated gene sets	LEE INTRATHYMIC T PROGENITOR	CESC	1
c2: curated gene sets	LEE DOUBLE POLAR THYMOCYTE	THYM	1
c2: curated gene sets	LE NEURONAL DIFFERENTIATION DN	KIRP	1
c2: curated gene sets	LANDIS ERBB2 BREAST TUMORS 65 UP	THYM	1
c2: curated gene sets	KYNG WERNER SYNDROME DN	KIRC	1

c2: curated gene sets	KYNG NORMAL AGING DN	KIRC	1
c2: curated gene sets	KUROKAWA LIVER CANCER EARLY RECURRENCE UP	KIRC	1
c2: curated gene sets	KRISHNAN FURIN TARGETS DN	TGCT	1
c2: curated gene sets	KORKOLA CHORIOCARCINOMA	LGG	1
c2: curated gene sets	KONG E2F1 TARGETS	KIRP	1
c2: curated gene sets	KONDO HYPOXIA	HNSC	1
c2: curated gene sets	KONDO COLON CANCER HCP WITH H3K27ME1	PRAD	1
c2: curated gene sets	KOINUMA COLON CANCER MSI DN	BRCA	1
c2: curated gene sets	KIM LIVER CANCER POOR SURVIVAL UP	LUAD	1
c2: curated gene sets	KIM GLIS2 TARGETS DN	THYM	1
c7: immunologic signature gene sets	KENNEDY PBMC DRYVAX AGE 18 40YO STIMULATED VS UNSTIMULATED 1 TO 48MO TOP DEG DN	DLBC	1
c2: curated gene sets	KEGG PHENYLALANINE METABOLISM	DLBC	1
c2: curated gene sets	KEGG PANTOTHENATE AND COA BIOSYNTHESIS	UVM	1
c2: curated gene sets	KEGG GLYCOSAMINOGLYCAN BIOSYNTHESIS KERATAN SULFATE	KIRC	1
c2: curated gene sets	KEGG FRUCTOSE AND MANNOSE METABOLISM	READ	1
c2: curated gene sets	KEGG ALLOGRAFT REJECTION	THYM	1
c7: immunologic signature gene sets	KAZMIN PBMC P FALCIPARUM RTSS AS01 UNKN AGE IMM WITH ARR VS IMM BY RRR PRIMARY IMMUNIZE WITH RECOMB ADENOVIRUS 35 1DY UP	SKCM	1

c7: immunologic signature gene sets	KAZMIN PBMC P FALCIPARUM RTSS AS01 AGE UNKNOWN CORRELATED WITH PROTECTION 56DY POSITIVE	UVM	1
c2: curated gene sets	KANNAN TP53 TARGETS DN	ACC	1
c2: curated gene sets	KANG CISPLATIN RESISTANCE DN	KIRC	1
c2: curated gene sets	JOSEPH RESPONSE TO SODIUM BUTYRATE UP	BRCA	1
c2: curated gene sets	JOHANSSON BRAIN CANCER EARLY VS LATE UP	KIRP	1
c2: curated gene sets	JIANG TIP30 TARGETS DN	READ	1
c2: curated gene sets	JIANG AGING HYPOTHALAMUS DN	DLBC	1
c2: curated gene sets	JI CARCINOGENESIS BY KRAS AND STK11 UP	UCS	1
c2: curated gene sets	IWANAGA E2F1 TARGETS INDUCED BY SERUM	SARC	1
c2: curated gene sets	IVANOV MUTATED IN COLON CANCER	KIRC	1
c2: curated gene sets	ITO PTTG1 TARGETS DN	LGG	1
c2: curated gene sets	INAMURA LUNG CANCER SCC SUBTYPES UP	ACC	1
c2: curated gene sets	IM SREBF1A TARGETS	PAAD	1
c2: curated gene sets	IIZUKA LIVER CANCER PROGRESSION G2 G3 DN	KIRC	1
c2: curated gene sets	IIZUKA LIVER CANCER EARLY RECURRENCE	LUAD	1
c2: curated gene sets	IGARASHI ATF4 TARGETS UP	UVM	1
c2: curated gene sets	ICHIBA GRAFT VERSUS HOST DISEASE 35D DN	DLBC	1
c2: curated gene sets	HWANG PROSTATE CANCER MARKERS	THYM	1

c2: curated gene sets	HUNSBERGER EXERCISE REGULATED GENES	DLBC	1
c2: curated gene sets	HUMMERICH BENIGN SKIN TUMOR DN	DLBC	1
c2: curated gene sets	HU GENOTOXIC DAMAGE 4HR	THYM	1
c8: cell type signature gene sets	HU FETAL RETINA HORIZONTAL	DLBC	1
c5: Ontology gene sets	HP ZOLLINGER ELLISON SYNDROME	KIRC	1
c5: Ontology gene sets	HP YELLOW WHITE LESIONS OF THE RETINA	DLBC	1
c5: Ontology gene sets	HP WIDENED CEREBELLAR SUBARACHNOID SPACE	KIRC	1
c5: Ontology gene sets	HP WIDE NASAL RIDGE	KIRP	1
c5: Ontology gene sets	HP WIDE CRANIAL SUTURES	DLBC	1
c5: Ontology gene sets	HP WHITE EYEBROW	SKCM	1
c5: Ontology gene sets	HP VOLVULUS	PRAD	1
c5: Ontology gene sets	HP VISUAL HALLUCINATIONS	KIRP	1
c5: Ontology gene sets	HP VISUAL FIXATION INSTABILITY	KIRC	1
c5: Ontology gene sets	HP VIRAL INFECTION INDUCED RHABDOMYOLYSIS	KIRC	1
c5: Ontology gene sets	HP VERY LONG CHAIN FATTY ACID ACCUMULATION	KIRC	1
c5: Ontology gene sets	HP VERTICAL NYSTAGMUS	DLBC	1
c5: Ontology gene sets	HP VERRUCAE	SKCM	1
c5: Ontology gene sets	HP VENOUS MALFORMATION	THCA	1
c5: Ontology gene sets	HP VAGINAL NEOPLASM	THYM	1
c5: Ontology gene sets	HP VAGINAL FISTULA	KIRC	1
c5: Ontology gene sets	HP UTERUS DIDELPHYS	LIHC	1
c5: Ontology gene sets	HP URETHRAL VALVE	READ	1
c5: Ontology gene sets	HP URETERAL STENOSIS	THYM	1
c5: Ontology gene sets	HP UPPER LIMB HYPERTONIA	KIRC	1

c5: Ontology gene sets	HP UPPER EYELID EDEMA	LUAD	1
c5: Ontology gene sets	HP UPPER EYELID COLOBOMA	KIRC	1
c5: Ontology gene sets	HP UNUSUAL SKIN INFECTION	BRCA	1
c5: Ontology gene sets	HP UNILATERAL CRYPTORCHIDISM	COAD	1
c5: Ontology gene sets	HP UNCONTROLLED EYE MOVEMENTS	PAAD	1
c5: Ontology gene sets	HP UNCONJUGATED HYPERBILIRUBINEMIA	HNSC	1
c5: Ontology gene sets	HP UNCOMBABLE HAIR	KIRC	1
c5: Ontology gene sets	HP ULNAR BOWING	COAD	1
c5: Ontology gene sets	HP TYPE II TRANSFERRIN ISOFORM PROFILE	PAAD	1
c5: Ontology gene sets	HP TURRICEPHALY	READ	1
c5: Ontology gene sets	HP TUBULOINTERSTITIAL NEPHRITIS	THYM	1
c5: Ontology gene sets	HP TRICUSPID ATRESIA	KIRC	1
c5: Ontology gene sets	HP TRIANGULAR MOUTH	DLBC	1
c5: Ontology gene sets	HP TRANSIENT NEUTROPENIA	KICH	1
c5: Ontology gene sets	HP TOTAL ANOMALOUS PULMONARY VENOUS RETURN	SARC	1
c5: Ontology gene sets	HP TONGUE THRUSTING	KIRC	1
c5: Ontology gene sets	HP TIP TOE GAIT	PRAD	1
c5: Ontology gene sets	HP THYROID ADENOMA	THYM	1
c5: Ontology gene sets	HP THIN NAIL	KIRC	1
c5: Ontology gene sets	HP THIN BONY CORTEX	KIRC	1
c5: Ontology gene sets	HP THICKENED RIBS	DLBC	1
c5: Ontology gene sets	HP THICKENED NUCHAL SKIN FOLD	DLBC	1
c5: Ontology gene sets	HP THICKENED CORTEX OF LONG BONES	KIRC	1
c5: Ontology gene sets	HP THICK HAIR	THCA	1
c5: Ontology gene sets	HP TALIPES VALGUS	KIRP	1

c5: Ontology gene sets	HP T CELL LYMPHOMA	THYM	1
c5: Ontology gene sets	HP SWAN NECK LIKE DEFORMITIES OF THE FINGERS	BLCA	1
c5: Ontology gene sets	HP SUPRANUCLEAR OPHTHALMOPLEGIA	SKCM	1
c5: Ontology gene sets	HP SUPRANUCLEAR GAZE PALSY	DLBC	1
c5: Ontology gene sets	HP SUPERNUMERARY MAXILLARY INCISOR	ACC	1
c5: Ontology gene sets	HP SUBDURAL HEMORRHAGE	UCEC	1
c5: Ontology gene sets	HP SUBACUTE PROGRESSIVE VIRAL HEPATITIS	KIRC	1
c5: Ontology gene sets	HP STEROID RESISTANT NEPHROTIC SYNDROME	ACC	1
c5: Ontology gene sets	HP STENOSIS OF THE EXTERNAL AUDITORY CANAL	READ	1
c5: Ontology gene sets	HP SPONTANEOUS RECURRENT EPISTAXIS	LGG	1
c5: Ontology gene sets	HP SPONTANEOUS PAIN SENSATION	LGG	1
c5: Ontology gene sets	HP SPOKEN WORD RECOGNITION DEFICIT	UVM	1
c5: Ontology gene sets	HP SPLIT NAIL	LUAD	1
c5: Ontology gene sets	HP SPASTIC ATAXIA	KIRC	1
c5: Ontology gene sets	HP SPARSE BONE TRABECULAE	ACC	1
c5: Ontology gene sets	HP SNORING	HNSC	1
c5: Ontology gene sets	HP SMALL SCROTUM	KICH	1
c5: Ontology gene sets	HP SMALL PLACENTA	DLBC	1
c5: Ontology gene sets	HP SMALL CEREBRAL CORTEX	LIHC	1
c5: Ontology gene sets	HP SLOW DECREASE IN VISUAL ACUITY	DLBC	1
c5: Ontology gene sets	HP SLENDER TOE	KIRC	1
c5: Ontology gene sets	HP SLENDER BUILD	THYM	1

c5: Ontology gene sets	HP SKULL ASYMMETRY	LUAD	1
c5: Ontology gene sets	HP SINGLE VENTRICLE	LUSC	1
c5: Ontology gene sets	HP SILVER GRAY HAIR	PCPG	1
c5: Ontology gene sets	HP SHOULDER PAIN	LGG	1
c5: Ontology gene sets	HP SHORT UMBILICAL CORD	DLBC	1
c5: Ontology gene sets	HP SHORT MIDDLE PHALANX OF FINGER	READ	1
c5: Ontology gene sets	HP SHORT LOWER LIMBS	LGG	1
c5: Ontology gene sets	HP SHORT 1ST METACARPAL	KIRC	1
c5: Ontology gene sets	HP SEVERE HEARING IMPAIRMENT	READ	1
c5: Ontology gene sets	HP SENSORY ATAXIA	UVM	1
c5: Ontology gene sets	HP SEMILOBAR HOLOPROSENCEPHALY	KIRC	1
c5: Ontology gene sets	HP SELECTIVE TOOTH AGENESIS	THYM	1
c5: Ontology gene sets	HP SEESAW NYSTAGMUS	LGG	1
c5: Ontology gene sets	HP SECUNDUM ATRIAL SEPTAL DEFECT	BRCA	1
c5: Ontology gene sets	HP SECOND DEGREE ATRIOVENTRICULAR BLOCK	KIRP	1
c5: Ontology gene sets	HP SEA BLUE HISTIOCYTOSIS	COAD	1
c5: Ontology gene sets	HP SCLEROSING CHOLANGITIS	HNSC	1
c5: Ontology gene sets	HP SCLERAL STAPHYLOMA	KIRC	1
c5: Ontology gene sets	HP SACCADIC OSCILLATION	KIRC	1
c5: Ontology gene sets	HP ROTARY NYSTAGMUS	THYM	1
c5: Ontology gene sets	HP RIGHT BUNDLE BRANCH BLOCK	DLBC	1
c5: Ontology gene sets	HP RICKETS	DLBC	1
c5: Ontology gene sets	HP RHIZOMELIC ARM SHORTENING	THCA	1
c5: Ontology gene sets	HP RETINAL COLOBOMA	THYM	1

c5: Ontology gene sets	HP RETINAL ARTERIAL TORTUOSITY	KICH	1
c5: Ontology gene sets	HP RETICULAR HYPERPIGMENTATION	MESO	1
c5: Ontology gene sets	HP RESTLESS LEGS	KIRC	1
c5: Ontology gene sets	HP RESPONSE TO DRUGS ACTING ON NEUROMUSCULAR TRANSMISSION	KIRC	1
c5: Ontology gene sets	HP RESPIRATORY INSUFFICIENCY DUE TO DEFECTIVE CILIARY CLEARANCE	BRCA	1
c5: Ontology gene sets	HP RESPIRATORY ARREST	LUAD	1
c5: Ontology gene sets	HP RENAL HAMARTOMA	THYM	1
c5: Ontology gene sets	HP REDUCTION OF NEUTROPHIL MOTILITY	KIRC	1
c5: Ontology gene sets	HP REDUCED SYSTOLIC FUNCTION	LUAD	1
c5: Ontology gene sets	HP REDUCED FACTOR XI ACTIVITY	LGG	1
c5: Ontology gene sets	HP REDUCED BRAIN N ACETYL ASPARTATE LEVEL BY MRS	KIRC	1
c5: Ontology gene sets	HP RED GREEN DYSCHROMATOPSIA	LUAD	1
c5: Ontology gene sets	HP RECURRENT STAPHYLOCOCCAL INFECTIONS	DLBC	1
c5: Ontology gene sets	HP RECURRENT HEMOPHILUS INFLUENZAE INFECTIONS	OV	1
c5: Ontology gene sets	HP RECURRENT GASTROENTERITIS	CESC	1
c5: Ontology gene sets	HP RECURRENT CUTANEOUS ABSCESS FORMATION	SKCM	1
c5: Ontology gene sets	HP RECURRENT CANDIDA INFECTIONS	SKCM	1

c5: Ontology gene sets	HP RECTAL POLYPOSIS	LIHC	1
c5: Ontology gene sets	HP RECTAL FISTULA	KIRC	1
c5: Ontology gene sets	HP PYRIDOXINE RESPONSIVE SIDEROBLASTIC ANEMIA	LIHC	1
c5: Ontology gene sets	HP PYODERMA GANGRENOSUM	SKCM	1
c5: Ontology gene sets	HP PURE RED CELL APLASIA	ACC	1
c5: Ontology gene sets	HP PULMONARY ARTERY ANEURYSM	LGG	1
c5: Ontology gene sets	HP PSYCHOTIC EPISODES	UVM	1
c5: Ontology gene sets	HP PSEUDOHYPOPARATHYROIDISM	PAAD	1
c5: Ontology gene sets	HP PSEUDOHYPOALDOSTERONISM	HNSC	1
c5: Ontology gene sets	HP PSEUDOEPIPHYSES	THYM	1
c5: Ontology gene sets	HP PROXIMAL MUSCLE WEAKNESS IN LOWER LIMBS	READ	1
c5: Ontology gene sets	HP PROMINENT VEINS ON TRUNK	LGG	1
c5: Ontology gene sets	HP PROMINENT SUPERFICIAL BLOOD VESSELS	DLBC	1
c5: Ontology gene sets	HP PROMINENT EAR HELIX	LGG	1
c5: Ontology gene sets	HP PROMINENT CALCANEUS	BLCA	1
c5: Ontology gene sets	HP PROGRESSIVE SPASTIC PARAPARESIS	KIRP	1
c5: Ontology gene sets	HP PRIMITIVE REFLEX	KIRC	1
c5: Ontology gene sets	HP PRIMARY HYPERCORTISOLISM	UCEC	1
c5: Ontology gene sets	HP PREMATURE PUBARCHE	KIRP	1
c5: Ontology gene sets	HP PRECOCIOUS PUBERTY IN FEMALES	KIRC	1
c5: Ontology gene sets	HP POSTEXERTIONAL MALAISE	LUSC	1
c5: Ontology gene sets	HP POSTERIOR POLAR CATARACT	KIRC	1
c5: Ontology gene sets	HP POSTERIOR EMBRYOTOXON	TGCT	1
c5: Ontology gene sets	HP POST PARTUM HEMORRHAGE	MESO	1
c5: Ontology gene sets	HP PORTAL FIBROSIS	PAAD	1

c5: Ontology gene sets	HP PONTOCEREBELLAR ATROPHY	KIRP	1
c5: Ontology gene sets	HP POLYGENIC INHERITANCE	PCPG	1
c5: Ontology gene sets	HP POLYCYSTIC LIVER DISEASE	LGG	1
c5: Ontology gene sets	HP POLAR CATARACT	KIRC	1
c5: Ontology gene sets	HP PITUITARY ADENOMA	DLBC	1
c5: Ontology gene sets	HP PILOMATRIXOMA	READ	1
c5: Ontology gene sets	HP PHYSICAL URTICARIA	KIRC	1
c5: Ontology gene sets	HP PHONOPHOBIA	THYM	1
c5: Ontology gene sets	HP PHOCOMELIA	KICH	1
c5: Ontology gene sets	HP PHARYNGITIS	SKCM	1
c5: Ontology gene sets	HP PES VALGUS	DLBC	1
c5: Ontology gene sets	HP PERIVASCULAR SPACES	UCEC	1
c5: Ontology gene sets	HP PERIPHERAL ARTERIAL STENOSIS	THCA	1
c5: Ontology gene sets	HP PELVIC KIDNEY	THYM	1
c5: Ontology gene sets	HP PECTORAL MUSCLE HYPOPLASIA APLASIA	KIRC	1
c5: Ontology gene sets	HP PATELLAR HYPOPLASIA	ACC	1
c5: Ontology gene sets	HP PATCHY CHANGES OF BONE MINERAL DENSITY	LGG	1
c5: Ontology gene sets	HP PAROXYSMAL DYSTONIA	KIRC	1
c5: Ontology gene sets	HP PAROXYSMAL BURSTS OF LAUGHTER	KIRC	1
c5: Ontology gene sets	HP PARATHYROID ADENOMA	LGG	1
c5: Ontology gene sets	HP PANCREATIC CALCIFICATION	OV	1
c5: Ontology gene sets	HP OXYCEPHALY	LUAD	1
c5: Ontology gene sets	HP OVERFRIENDLINESS	GBM	1
c5: Ontology gene sets	HP OVERBITE	THYM	1
c5: Ontology gene sets	HP OSTEOPETROSIS	KIRC	1
c5: Ontology gene sets	HP OSTEOLYSIS INVOLVING BONES OF THE FEET	ACC	1
c5: Ontology gene sets	HP OSTEOCHONDROSIS	LUAD	1

c5: Ontology gene sets	HP ORAL CAVITY BLEEDING	THYM	1
c5: Ontology gene sets	HP OPTIC NERVE MISROUTING	SKCM	1
c5: Ontology gene sets	HP OPTIC NERVE DYSPLASIA	KIRC	1
c5: Ontology gene sets	HP OPSOCLONUS	KIRC	1
c5: Ontology gene sets	HP OPEN OPERCULUM	LUSC	1
c5: Ontology gene sets	HP OPEN ANGLE GLAUCOMA	KIRP	1
c5: Ontology gene sets	HP OLIVOPONTOCEREBELLAR ATROPHY	KIRC	1
c5: Ontology gene sets	HP OCULOMOTOR NERVE PALSY	READ	1
c5: Ontology gene sets	HP OCCIPITAL MENINGOCELE	LUSC	1
c5: Ontology gene sets	HP NYSTAGMUS INDUCED HEAD NODDING	KICH	1
c5: Ontology gene sets	HP NUMEROUS NEVI	KICH	1
c5: Ontology gene sets	HP NONPROGRESSIVE ENCEPHALOPATHY	LGG	1
c5: Ontology gene sets	HP NON-HODGKIN LYMPHOMA	THYM	1
c5: Ontology gene sets	HP NON-CONVULSIVE STATUS EPILEPTICUS WITHOUT COMA	KIRP	1
c5: Ontology gene sets	HP NEVUS FLAMMEUS OF THE FOREHEAD	KIRC	1
c5: Ontology gene sets	HP NEUTROPENIA IN PRESENCE OF ANTI NEUTROPHIL ANTIBODIES	SKCM	1
c5: Ontology gene sets	HP NEURONAL LOSS IN CENTRAL NERVOUS SYSTEM	DLBC	1
c5: Ontology gene sets	HP NEURONAL LOSS IN BASAL GANGLIA	KIRC	1
c5: Ontology gene sets	HP NEPHRONOPHTHISIS	PRAD	1
c5: Ontology gene sets	HP NEOPLASM OF THE THYMUS	UCEC	1
c5: Ontology gene sets	HP NEOPLASM OF THE PITUITARY GLAND	DLBC	1
c5: Ontology gene sets	HP NEONATAL BREATHING DYSREGULATION	COAD	1

c5: Ontology gene sets	HP NARROW PELVIS BONE	COAD	1
c5: Ontology gene sets	HP NARROW JAW	LUAD	1
c5: Ontology gene sets	HP NAIL PITS	BRCA	1
c5: Ontology gene sets	HP MYOCARDIAL FIBROSIS	KICH	1
c5: Ontology gene sets	HP MYELOPROLIFERATIVE DISORDER	UVM	1
c5: Ontology gene sets	HP MUSCLE FLACCIDITY	COAD	1
c5: Ontology gene sets	HP MUSCLE FIBRILLATION	PCPG	1
c5: Ontology gene sets	HP MULTIPLE SUTURE CRANIOSYNOSTOSIS	READ	1
c5: Ontology gene sets	HP MULTIPLE ROWS OF EYELASHES	KIRC	1
c5: Ontology gene sets	HP MULTIPLE LENTIGINES	PCPG	1
c5: Ontology gene sets	HP MOTTLED PIGMENTATION	UVM	1
c5: Ontology gene sets	HP MORPHOLOGICAL ABNORMALITY OF THE VESTIBULE OF THE INNER EAR	READ	1
c5: Ontology gene sets	HP MIXED HYPO AND HYPERPIGMENTATION OF THE SKIN	UVM	1
c5: Ontology gene sets	HP MITTEN DEFORMITY	PAAD	1
c5: Ontology gene sets	HP MICROVESICULAR HEPATIC STEATOSIS	READ	1
c5: Ontology gene sets	HP METRORRHAGIA	THYM	1
c5: Ontology gene sets	HP METOPIC SYNOSTOSIS	KIRC	1
c5: Ontology gene sets	HP METATARSUS VALGUS	MESO	1
c5: Ontology gene sets	HP METAPHYSEAL SCLEROSIS	THYM	1
c5: Ontology gene sets	HP MENOMETRORRHAGIA	HNSC	1
c5: Ontology gene sets	HP MEMBRANOPROLIFERATIVE GLOMERULONEPHRITIS	LIHC	1
c5: Ontology gene sets	HP MEDIASTINAL LYMPHADENOPATHY	THYM	1
c5: Ontology gene sets	HP MEDIAN CLEFT PALATE	DLBC	1

c5: Ontology gene sets	HP MANIA	READ	1
c5: Ontology gene sets	HP MALROTATION OF SMALL BOWEL	UVM	1
c5: Ontology gene sets	HP MALIGNANT GENITOURINARY TRACT TUMOR	ACC	1
c5: Ontology gene sets	HP MACROSCOPIC HEMATURIA	UVM	1
c5: Ontology gene sets	HP MACROORCHIDISM	THYM	1
c5: Ontology gene sets	HP LYMPHADENITIS	THYM	1
c5: Ontology gene sets	HP LUPUS ANTICOAGULANT	CESC	1
c5: Ontology gene sets	HP LOWER LIMB UNDERGROWTH	COAD	1
c5: Ontology gene sets	HP LOW SET NIPPLES	KICH	1
c5: Ontology gene sets	HP LOW SELF ESTEEM	PAAD	1
c5: Ontology gene sets	HP LOSS OF PURKINJE CELLS IN THE CEREBELLAR VERMIS	KIRC	1
c5: Ontology gene sets	HP LONG UPPER LIP	HNSC	1
c5: Ontology gene sets	HP LONG NECK	READ	1
c5: Ontology gene sets	HP LOCALIZED HIRSUTISM	KIRC	1
c5: Ontology gene sets	HP LOBULATED TONGUE	PCPG	1
c5: Ontology gene sets	HP LINGUAL DYSTONIA	KIRP	1
c5: Ontology gene sets	HP LEYDIG CELL NEOPLASIA	KIRP	1
c5: Ontology gene sets	HP LENS LUXATION	PAAD	1
c5: Ontology gene sets	HP LEBER OPTIC ATROPHY	LGG	1
c5: Ontology gene sets	HP LATERAL CLAVICLE HOOK	KIRC	1
c5: Ontology gene sets	HP LARYNGOTRACHEOMALACIA	READ	1
c5: Ontology gene sets	HP LARYNGEAL HYPOPLASIA	ACC	1
c5: Ontology gene sets	HP LARYNGEAL DYSTONIA	THYM	1
c5: Ontology gene sets	HP LARGE CAFE AU LAIT MACULES WITH IRREGULAR MARGINS	KIRC	1
c5: Ontology gene sets	HP LACK OF T CELL FUNCTION	LUAD	1
c5: Ontology gene sets	HP LABIAL HYPERTROPHY	UVM	1
c5: Ontology gene sets	HP KETOTIC HYPOGLYCEMIA	KIRP	1

c5: Ontology gene sets	HP JOINT CONTRACTURE OF THE 5TH FINGER	KIRC	1
c5: Ontology gene sets	HP JERKY HEAD MOVEMENTS	KIRC	1
c5: Ontology gene sets	HP IRREGULAR VERTEBRAL ENDPLATES	DLBC	1
c5: Ontology gene sets	HP IRIDODONESIS	GBM	1
c5: Ontology gene sets	HP IRIDOCYCLITIS	UVM	1
c5: Ontology gene sets	HP INTUSSUSCEPTION	KIRC	1
c5: Ontology gene sets	HP INTRAVENTRICULAR HEMORRHAGE	ACC	1
c5: Ontology gene sets	HP INTESTINAL FISTULA	KIRC	1
c5: Ontology gene sets	HP INTESTINAL CARCINOID	KIRP	1
c5: Ontology gene sets	HP INTERSTITIAL PNEUMONITIS	BRCA	1
c5: Ontology gene sets	HP INTELLECTUAL DISABILITY BORDERLINE	GBM	1
c5: Ontology gene sets	HP INSPIRATORY STRIDOR	PAAD	1
c5: Ontology gene sets	HP INCREASED SKULL OSSIFICATION	BRCA	1
c5: Ontology gene sets	HP INCREASED SERUM TESTOSTERONE LEVEL	UVM	1
c5: Ontology gene sets	HP INCREASED SERUM IRON	KIRC	1
c5: Ontology gene sets	HP INCREASED MEGAKARYOCYTE COUNT	KIRC	1
c5: Ontology gene sets	HP INCREASED LEVEL OF L FUCOSE IN URINE	KIRC	1
c5: Ontology gene sets	HP INCREASED HEMOGLOBIN	BLCA	1
c5: Ontology gene sets	HP INCREASED FIBULAR DIAMETER	KIRC	1
c5: Ontology gene sets	HP INCREASED DENSITY OF LONG BONES	KIRC	1
c5: Ontology gene sets	HP INCREASED CUP TO DISC RATIO	KIRP	1

c5: Ontology gene sets	HP INCREASED CIRCULATING IGA LEVEL	UVM	1
c5: Ontology gene sets	HP INCREASED CIRCULATING CORTISOL LEVEL	UCEC	1
c5: Ontology gene sets	HP INCREASED BLOOD UREA NITROGEN	DLBC	1
c5: Ontology gene sets	HP INCREASED ASPARTATE FAMILY AMINO ACID LEVEL IN URINE	BRCA	1
c5: Ontology gene sets	HP INCOMPREHENSIBLE SPEECH	TGCT	1
c5: Ontology gene sets	HP INCISOR MACRODONTIA	THYM	1
c5: Ontology gene sets	HP INABILITY TO WALK BY CHILDHOOD ADOLESCENCE	KIRP	1
c5: Ontology gene sets	HP IMPAIRED THERMAL SENSITIVITY	LGG	1
c5: Ontology gene sets	HP IMPAIRED TEMPERATURE SENSATION	KIRC	1
c5: Ontology gene sets	HP IMPAIRED RISTOCETIN INDUCED PLATELET AGGREGATION	UVM	1
c5: Ontology gene sets	HP IMPAIRED OROPHARYNGEAL SWALLOW RESPONSE	KIRC	1
c5: Ontology gene sets	HP IMPAIRED NEUTROPHIL BACTERICIDAL ACTIVITY	LGG	1
c5: Ontology gene sets	HP IMPAIRED LYMPHOCYTE TRANSFORMATION WITH PHYTOHEMAGGLUTININ	BLCA	1
c5: Ontology gene sets	HP IMPAIRED ANTIGEN SPECIFIC RESPONSE	UVM	1
c5: Ontology gene sets	HP IMPACTED TOOTH	LGG	1
c5: Ontology gene sets	HP IMMUNE DYSREGULATION	CESC	1
c5: Ontology gene sets	HP IMMOTILE CILIA	KIRP	1
c5: Ontology gene sets	HP HYPOPLASTIC IRIS STROMA	KIRC	1

c5: Ontology gene sets	HP HYPOPLASIA OF THE EPIGLOTTIS	KIRC	1
c5: Ontology gene sets	HP HYPOPHOSPHATEMIA	DLBC	1
c5: Ontology gene sets	HP HYPOKALEMIC METABOLIC ALKALOSIS	PAAD	1
c5: Ontology gene sets	HP HYPOKALEMIC ALKALOSIS	PAAD	1
c5: Ontology gene sets	HP HYPOCHROMIA	UVM	1
c5: Ontology gene sets	HP HYPOCALCEMIC TETANY	SKCM	1
c5: Ontology gene sets	HP HYPOCALCEMIC SEIZURES	SKCM	1
c5: Ontology gene sets	HP HYPERURICOSURIA	PRAD	1
c5: Ontology gene sets	HP HYPERSPLENISM	THYM	1
c5: Ontology gene sets	HP HYPERPLASIA OF THE MAXILLA	THYM	1
c5: Ontology gene sets	HP HYPERORALITY	UVM	1
c5: Ontology gene sets	HP HYPERKETONEMIA	CESC	1
c5: Ontology gene sets	HP HYPEREXTENSIBILITY OF THE KNEE	DLBC	1
c5: Ontology gene sets	HP HYPEREXTENSIBILITY AT WRISTS	KIRC	1
c5: Ontology gene sets	HP HYPERACTIVE PATELLAR REFLEX	BRCA	1
c5: Ontology gene sets	HP HODGKIN LYMPHOMA	READ	1
c5: Ontology gene sets	HP HIP FLEXOR WEAKNESS	KIRC	1
c5: Ontology gene sets	HP HEPATIC NECROSIS	KIRP	1
c5: Ontology gene sets	HP HEPATIC ENCEPHALOPATHY	KIRC	1
c5: Ontology gene sets	HP HEMOLYTIC UREMIC SYNDROME	LGG	1
c5: Ontology gene sets	HP HEAD TREMOR	DLBC	1
c5: Ontology gene sets	HP HASHIMOTO THYROIDITIS	UVM	1
c5: Ontology gene sets	HP HAMSTRING CONTRACTURES	READ	1
c5: Ontology gene sets	HP HAMMERTOE	DLBC	1
c5: Ontology gene sets	HP HAMARTOMA OF TONGUE	PCPG	1

c5: Ontology gene sets	HP GLUTARIC ACIDURIA	OV	1
c5: Ontology gene sets	HP GLUCAGONOMA	UCEC	1
c5: Ontology gene sets	HP GINGIVITIS	READ	1
c5: Ontology gene sets	HP GENERALIZED LYMPHADENOPATHY	UVM	1
c5: Ontology gene sets	HP FUSION OF THE CEREBELLAR HEMISPHERES	MESO	1
c5: Ontology gene sets	HP FULMINANT HEPATIC FAILURE	KIRC	1
c5: Ontology gene sets	HP FRONTAL BALDING	KIRC	1
c5: Ontology gene sets	HP FOOT ACROOSTEOLYSIS	ACC	1
c5: Ontology gene sets	HP FOLLICULITIS	SKCM	1
c5: Ontology gene sets	HP FOCAL SENSORY SEIZURE	PAAD	1
c5: Ontology gene sets	HP FOCAL CORTICAL DYSPLASIA	PAAD	1
c5: Ontology gene sets	HP FLAT NASAL ALAE	BRCA	1
c5: Ontology gene sets	HP FLAT CORNEA	KICH	1
c5: Ontology gene sets	HP FLARED NOSTRILS	COAD	1
c5: Ontology gene sets	HP FLANK PAIN	KIRC	1
c5: Ontology gene sets	HP FINGER SWELLING	BRCA	1
c5: Ontology gene sets	HP FINGER AGNOSIA	UCEC	1
c5: Ontology gene sets	HP FIBROSARCOMA	KIRC	1
c5: Ontology gene sets	HP FIBROADENOMA OF THE BREAST	THYM	1
c5: Ontology gene sets	HP FEMALE SEXUAL DYSFUNCTION	DLBC	1
c5: Ontology gene sets	HP FEMALE PSEUDOHERMAPHRODITISM	BRCA	1
c5: Ontology gene sets	HP FATTY REPLACEMENT OF SKELETAL MUSCLE	KIRC	1
c5: Ontology gene sets	HP FACIAL TICS	UCEC	1
c5: Ontology gene sets	HP FACIAL GRIMACING	UCEC	1
c5: Ontology gene sets	HP FACIAL CAPILLARY HEMANGIOMA	BLCA	1

c5: Ontology gene sets	HP EXSTROPHY	MESO	1
c5: Ontology gene sets	HP EXERCISE INDUCED MUSCLE CRAMPS	LUAD	1
c5: Ontology gene sets	HP EXAGGERATED CUPID S BOW	KIRC	1
c5: Ontology gene sets	HP EVERSION OF LATERAL THIRD OF LOWER EYELIDS	HNSC	1
c5: Ontology gene sets	HP EURYBLEPHARON	KIRC	1
c5: Ontology gene sets	HP ETHYLMALONIC ACIDURIA	KIRP	1
c5: Ontology gene sets	HP ESOPHAGEAL ULCERATION	PAAD	1
c5: Ontology gene sets	HP ERYTHROID HYPERPLASIA	THYM	1
c5: Ontology gene sets	HP ERYTHRODERMA	READ	1
c5: Ontology gene sets	HP EQUINUS CALCANEUS	KIRC	1
c5: Ontology gene sets	HP EPISODIC FLACCID WEAKNESS	LIHC	1
c5: Ontology gene sets	HP EPIGASTRIC PAIN	LGG	1
c5: Ontology gene sets	HP ENLARGEMENT OF THE COSTOCHONDRAL JUNCTION	KIRC	1
c5: Ontology gene sets	HP ENLARGEMENT OF THE ANKLES	ACC	1
c5: Ontology gene sets	HP ENLARGED TONSILS	SKCM	1
c5: Ontology gene sets	HP ENLARGED THORAX	DLBC	1
c5: Ontology gene sets	HP ENLARGED EPIPHYSES	KIRC	1
c5: Ontology gene sets	HP ENDOCARDIAL FIBROELASTOSIS	LIHC	1
c5: Ontology gene sets	HP ENCEPHALOMALACIA	KIRC	1
c5: Ontology gene sets	HP ENCEPHALITIS	SARC	1
c5: Ontology gene sets	HP EMG CHRONIC DENERVATION SIGNS	THYM	1
c5: Ontology gene sets	HP ELEVATED TISSUE NON-SPECIFIC ALKALINE PHOSPHATASE	KIRC	1
c5: Ontology gene sets	HP ELEVATED SERUM ALANINE AMINOTRANSFERASE	BLCA	1

c5: Ontology gene sets	HP ELEVATED RED CELL ADENOSINE DEAMINASE LEVEL	ACC	1
c5: Ontology gene sets	HP ELEVATED CIRCULATING LONG CHAIN FATTY ACID CONCENTRATION	KIRC	1
c5: Ontology gene sets	HP ELEVATED CALCITONIN	PRAD	1
c5: Ontology gene sets	HP ELEVATED ALKALINE PHOSPHATASE OF BONE ORIGIN	KIRC	1
c5: Ontology gene sets	HP EEG WITH CONTINUOUS SLOW ACTIVITY	UVM	1
c5: Ontology gene sets	HP ECTOPIC ANTERIOR PITUITARY GLAND	KIRP	1
c5: Ontology gene sets	HP DYSPLASTIC CORPUS CALLOSUM	THYM	1
c5: Ontology gene sets	HP DYSPAREUNIA	DLBC	1
c5: Ontology gene sets	HP DYSMENORRHEA	BLCA	1
c5: Ontology gene sets	HP DYSGAMMAGLOBULINEMIA	SKCM	1
c5: Ontology gene sets	HP DISPROPORTIONATE SHORT TRUNK SHORT STATURE	COAD	1
c5: Ontology gene sets	HP DILATATION OF THE CEREBRAL ARTERY	READ	1
c5: Ontology gene sets	HP DIAPHYSEAL SCLEROSIS	KIRC	1
c5: Ontology gene sets	HP DERMATOCHALASIS	LGG	1
c5: Ontology gene sets	HP DELAYED ERUPTION OF PERMANENT TEETH	LGG	1
c5: Ontology gene sets	HP DEGENERATION OF THE LATERAL CORTICOSPINAL TRACTS	THYM	1
c5: Ontology gene sets	HP DEFECTIVE T CELL PROLIFERATION	SKCM	1
c5: Ontology gene sets	HP DECREASED SPECIFIC ANTIBODY RESPONSE TO VACCINATION	UVM	1

c5: Ontology gene sets	HP DECREASED SPECIFIC ANTI POLYSACCHARIDE ANTIBODY LEVEL	THYM	1
c5: Ontology gene sets	HP DECREASED SIZE OF NERVE TERMINALS	KIRC	1
c5: Ontology gene sets	HP DECREASED SERUM TERMINAL COMPLEMENT COMPONENT	LIHC	1
c5: Ontology gene sets	HP DECREASED SERUM COMPLEMENT FACTOR I	LGG	1
c5: Ontology gene sets	HP DECREASED SERUM COMPLEMENT FACTOR B	LGG	1
c5: Ontology gene sets	HP DECREASED PULMONARY FUNCTION	READ	1
c5: Ontology gene sets	HP DECREASED MINIATURE ENDPLATE POTENTIALS	KIRC	1
c5: Ontology gene sets	HP DECREASED METHYLMALONYL COA MUTASE ACTIVITY	LGG	1
c5: Ontology gene sets	HP DECREASED CIRCULATING LEVEL OF SPECIFIC ANTIBODY	UVM	1
c5: Ontology gene sets	HP DECREASED CIRCULATING ACTH LEVEL	TGCT	1
c5: Ontology gene sets	HP DECREASED CD4 CD8 RATIO	LGG	1
c5: Ontology gene sets	HP DECREASED ACTIVITY OF NADPH OXIDASE	LGG	1
c5: Ontology gene sets	HP CYSTINURIA	UCEC	1
c5: Ontology gene sets	HP CUTANEOUS ABSCESS	SKCM	1
c5: Ontology gene sets	HP CROSSED FUSED RENAL ECTOPIA	KIRC	1
c5: Ontology gene sets	HP CRANIOFACIAL DYSOSTOSIS	THCA	1
c5: Ontology gene sets	HP CORTICAL THICKENING OF LONG BONE DIAPHYSES	LGG	1
c5: Ontology gene sets	HP CORTICAL MYOCLONUS	HNSC	1
c5: Ontology gene sets	HP CORNEAL STROMAL EDEMA	SKCM	1

c5: Ontology gene sets	HP COOMBS POSITIVE HEMOLYTIC ANEMIA	THYM	1
c5: Ontology gene sets	HP CONTIGUOUS GENE SYNDROME	BRCA	1
c5: Ontology gene sets	HP CONNECTIVE TISSUE NEVI	THYM	1
c5: Ontology gene sets	HP CONGENITAL THROMBOCYTOPENIA	LGG	1
c5: Ontology gene sets	HP CONGENITAL POSTERIOR URETHRAL VALVE	PRAD	1
c5: Ontology gene sets	HP CONGENITAL MUSCULAR TORTICOLLIS	UCEC	1
c5: Ontology gene sets	HP CONGENITAL FINGER FLEXION CONTRACTURES	ACC	1
c5: Ontology gene sets	HP CONGENITAL BLINDNESS	COAD	1
c5: Ontology gene sets	HP CONFETTI LIKE HYPOPIGMENTED MACULES	KIRP	1
c5: Ontology gene sets	HP CONE CONE ROD DYSTROPHY	PRAD	1
c5: Ontology gene sets	HP COMPLEX FEBRILE SEIZURE	UCEC	1
c5: Ontology gene sets	HP CLINODACTYLY OF THE 4TH FINGER	LGG	1
c5: Ontology gene sets	HP CHYLOTHORAX	MESO	1
c5: Ontology gene sets	HP CHRONIC PAIN	PAAD	1
c5: Ontology gene sets	HP CHRONIC MYELOGENOUS LEUKEMIA	HNSC	1
c5: Ontology gene sets	HP CHRONIC ATROPHIC GASTRITIS	KIRC	1
c5: Ontology gene sets	HP CHROMOSOME BREAKAGE	READ	1
c5: Ontology gene sets	HP CHOROID PLEXUS CARCINOMA	UCEC	1
c5: Ontology gene sets	HP CHONDROSARCOMA	LUAD	1
c5: Ontology gene sets	HP CHONDROCALCINOSIS	COAD	1
c5: Ontology gene sets	HP CERVICAL C2 C3 VERTEBRAL FUSION	STAD	1

c5: Ontology gene sets	HP CEREBRAL ARTERIOVENOUS MALFORMATION	KIRC	1
c5: Ontology gene sets	HP CENTROCECAL SCOTOMA	KICH	1
c5: Ontology gene sets	HP CENTRALLY NUCLEATED SKELETAL MUSCLE FIBERS	READ	1
c5: Ontology gene sets	HP CENTRAL ADRENAL INSUFFICIENCY	KIRC	1
c5: Ontology gene sets	HP CARCINOID TUMOR	COAD	1
c5: Ontology gene sets	HP CAPITATE HAMATE FUSION	READ	1
c5: Ontology gene sets	HP CALCIFICATION OF CARTILAGE	THYM	1
c5: Ontology gene sets	HP BUDD CHIARI SYNDROME	KIRC	1
c5: Ontology gene sets	HP BROAD PHILTRUM	READ	1
c5: Ontology gene sets	HP BROAD FEMORAL NECK	KIRC	1
c5: Ontology gene sets	HP BROAD FACE	READ	1
c5: Ontology gene sets	HP BREAST APLASIA	ACC	1
c5: Ontology gene sets	HP BRANCHIAL CYST	READ	1
c5: Ontology gene sets	HP BRANCHIAL ANOMALY	THYM	1
c5: Ontology gene sets	HP BOWEL DIVERTICULOSIS	BRCA	1
c5: Ontology gene sets	HP BONE FRACTURE	LIHC	1
c5: Ontology gene sets	HP BLADDER DIVERTICULUM	PCPG	1
c5: Ontology gene sets	HP BILATERAL TALIPES EQUINOVARUS	KIRC	1
c5: Ontology gene sets	HP BIFID NASAL TIP	KIRC	1
c5: Ontology gene sets	HP BIFID EPIGLOTTIS	KIRC	1
c5: Ontology gene sets	HP BICARBONATE WASTING RENAL TUBULAR ACIDOSIS	KIRC	1
c5: Ontology gene sets	HP BASILAR IMPRESSION	UVM	1
c5: Ontology gene sets	HP BASAL GANGLIA GLIOSIS	KIRC	1
c5: Ontology gene sets	HP BACTERIAL ENDOCARDITIS	KIRC	1
c5: Ontology gene sets	HP B LYMPHOCYTOPENIA	COAD	1
c5: Ontology gene sets	HP B CELL LYMPHOMA	THYM	1
c5: Ontology gene sets	HP AXONAL REGENERATION	UCEC	1

c5: Ontology gene sets	HP AXONAL DEGENERATION	KIRP	1
c5: Ontology gene sets	HP AXIAL DYSTONIA	KIRP	1
c5: Ontology gene sets	HP AVASCULAR NECROSIS OF THE CAPITAL FEMORAL EPIPHYSIS	KIRC	1
c5: Ontology gene sets	HP AUTOIMMUNE THROMBOCYTOPENIA	READ	1
c5: Ontology gene sets	HP AUTOIMMUNE HEMOLYTIC ANEMIA	THYM	1
c5: Ontology gene sets	HP AUDITORY HALLUCINATIONS	LGG	1
c5: Ontology gene sets	HP ATLANTOAXIAL DISLOCATION	KIRC	1
c5: Ontology gene sets	HP ASTERIXIS	UVM	1
c5: Ontology gene sets	HP ARTERIAL DISSECTION	ACC	1
c5: Ontology gene sets	HP ARNOLD CHIARI TYPE I MALFORMATION	TGCT	1
c5: Ontology gene sets	HP APPENDICULAR HYPOTONIA	SARC	1
c5: Ontology gene sets	HP APNEIC EPISODES PRECIPITATED BY ILLNESS FATIGUE STRESS	LUAD	1
c5: Ontology gene sets	HP APNEIC EPISODES IN INFANCY	KIRC	1
c5: Ontology gene sets	HP APLASIA OF THE ULNA	LUAD	1
c5: Ontology gene sets	HP APLASIA OF THE SEMICIRCULAR CANAL	READ	1
c5: Ontology gene sets	HP APLASIA OF THE PROXIMAL PHALANGES OF THE HAND	ACC	1
c5: Ontology gene sets	HP APLASIA OF THE PHALANGES OF THE HAND	READ	1
c5: Ontology gene sets	HP APLASIA OF METACARPAL BONES	ACC	1
c5: Ontology gene sets	HP APLASIA HYPOPLASIA OF THE THYMUS	THYM	1
c5: Ontology gene sets	HP APLASIA HYPOPLASIA OF THE PROXIMAL PHALANGES OF THE HAND	UCEC	1

c5: Ontology gene sets	HP APLASIA HYPOPLASIA OF THE PHALANGES OF THE TOES	DLBC	1
c5: Ontology gene sets	HP APLASIA HYPOPLASIA OF THE PHALANGES OF THE HALLUX	KIRP	1
c5: Ontology gene sets	HP APLASIA HYPOPLASIA OF THE FEMUR	DLBC	1
c5: Ontology gene sets	HP APLASIA HYPOPLASIA OF THE FALLOPIAN TUBE	ACC	1
c5: Ontology gene sets	HP APLASIA HYPOPLASIA OF THE DISTAL PHALANX OF THE 5TH TOE	BRCA	1
c5: Ontology gene sets	HP APLASIA HYPOPLASIA INVOLVING THE FEMORAL HEAD AND NECK	DLBC	1
c5: Ontology gene sets	HP APLASIA CUTIS CONGENITA OVER THE SCALP VERTEX	LUAD	1
c5: Ontology gene sets	HP ANTERIOR RIB CUPPING	KIRP	1
c5: Ontology gene sets	HP ANOTIA	ACC	1
c5: Ontology gene sets	HP ANGIOKERATOMA	LGG	1
c5: Ontology gene sets	HP ANEMIC PALLOR	KIRC	1
c5: Ontology gene sets	HP ANAPHYLACTIC SHOCK	KIRC	1
c5: Ontology gene sets	HP ALOPECIA UNIVERSALIS	LUSC	1
c5: Ontology gene sets	HP AGENESIS OF MAXILLARY INCISOR	KIRC	1
c5: Ontology gene sets	HP AGENESIS OF INCISOR	KIRC	1
c5: Ontology gene sets	HP ADRENOCORTICOTROPIN DEFICIENT ADRENAL INSUFFICIENCY	SKCM	1
c5: Ontology gene sets	HP ADRENOCORTICAL ABNORMALITY	UCEC	1
c5: Ontology gene sets	HP ADENOMATOUS COLONIC POLYPOSIS	KIRC	1

c5: Ontology gene sets	HP ACUTE LYMPHOBLASTIC LEUKEMIA	THYM	1
c5: Ontology gene sets	HP ACUTE INFECTIOUS PNEUMONIA	HNSC	1
c5: Ontology gene sets	HP ACROMESOMELIA	KIRC	1
c5: Ontology gene sets	HP ACROCYANOSIS	KIRC	1
c5: Ontology gene sets	HP ACNE INVERSA	UVM	1
c5: Ontology gene sets	HP ACHOLIC STOOLS	COAD	1
c5: Ontology gene sets	HP ACETABULAR SPURS	BLCA	1
c5: Ontology gene sets	HP ABSENT TESTIS	LIHC	1
c5: Ontology gene sets	HP ABSENT STERNAL OSSIFICATION	LIHC	1
c5: Ontology gene sets	HP ABSENT SKIN PIGMENTATION	SKCM	1
c5: Ontology gene sets	HP ABSENT SHORTENED DYNEIN ARMS	BRCA	1
c5: Ontology gene sets	HP ABSENT OUTER DYNEIN ARMS	BRCA	1
c5: Ontology gene sets	HP ABSENT OR MINIMALLY OSSIFIED VERTEBRAL BODIES	THYM	1
c5: Ontology gene sets	HP ABSENT FOREARM BONE	THYM	1
c5: Ontology gene sets	HP ABNORMALLY LOW PITCHED VOICE	KIRC	1
c5: Ontology gene sets	HP ABNORMALITY OF VENOUS PHYSIOLOGY	KIRC	1
c5: Ontology gene sets	HP ABNORMALITY OF UPPER LIMB METAPHYSIS	KIRC	1
c5: Ontology gene sets	HP ABNORMALITY OF THE VERTEBRAL ENDPLATES	DLBC	1
c5: Ontology gene sets	HP ABNORMALITY OF THE THORACIC CAVITY	DLBC	1
c5: Ontology gene sets	HP ABNORMALITY OF THE PARATHYROID MORPHOLOGY	THCA	1

c5: Ontology gene sets	HP ABNORMALITY OF THE NASAL SKELETON	KIRC	1
c5: Ontology gene sets	HP ABNORMALITY OF THE MUSCULATURE OF THE UPPER ARM	PAAD	1
c5: Ontology gene sets	HP ABNORMALITY OF THE MIDDLE PHALANX OF THE 5TH FINGER	READ	1
c5: Ontology gene sets	HP ABNORMALITY OF THE MIDDLE PHALANGES OF THE TOES	UVM	1
c5: Ontology gene sets	HP ABNORMALITY OF THE FORAMEN MAGNUM	THYM	1
c5: Ontology gene sets	HP ABNORMALITY OF THE FIRST METATARSAL BONE	KIRP	1
c5: Ontology gene sets	HP ABNORMALITY OF THE EUSTACHIAN TUBE	ACC	1
c5: Ontology gene sets	HP ABNORMALITY OF THE 3RD TOE	PRAD	1
c5: Ontology gene sets	HP ABNORMALITY OF OLFACTORY LOBE MORPHOLOGY	KIRC	1
c5: Ontology gene sets	HP ABNORMALITY OF NEUTROPHIL PHYSIOLOGY	LGG	1
c5: Ontology gene sets	HP ABNORMALITY OF LOWER LIMB EPIPHYSIS MORPHOLOGY	TGCT	1
c5: Ontology gene sets	HP ABNORMALITY OF COMPLEMENT SYSTEM	DLBC	1
c5: Ontology gene sets	HP ABNORMALITY OF CIRCULATING LEPTIN LEVEL	COAD	1
c5: Ontology gene sets	HP ABNORMALITY OF CHROMOSOME STABILITY	READ	1
c5: Ontology gene sets	HP ABNORMALITY OF CHROMOSOME SEGREGATION	LIHC	1
c5: Ontology gene sets	HP ABNORMALITY OF AMINO ACID METABOLISM	LIHC	1

c5: Ontology gene sets	HP ABNORMAL URINE OSMOLALITY	UVM	1
c5: Ontology gene sets	HP ABNORMAL URINE ALPHA KETOGLUTARATE CONCENTRATION	LGG	1
c5: Ontology gene sets	HP ABNORMAL UPPER MOTOR NEURON MORPHOLOGY	THYM	1
c5: Ontology gene sets	HP ABNORMAL THALAMIC MRI SIGNAL INTENSITY	DLBC	1
c5: Ontology gene sets	HP ABNORMAL SWEAT GLAND MORPHOLOGY	ACC	1
c5: Ontology gene sets	HP ABNORMAL SUBCLAVIAN ARTERY MORPHOLOGY	KIRC	1
c5: Ontology gene sets	HP ABNORMAL SHARPEY FIBER MORPHOLOGY	COAD	1
c5: Ontology gene sets	HP ABNORMAL SERUM INTERLEUKIN LEVEL	UVM	1
c5: Ontology gene sets	HP ABNORMAL RENAL COLLECTING SYSTEM MORPHOLOGY	READ	1
c5: Ontology gene sets	HP ABNORMAL QRS COMPLEX	LGG	1
c5: Ontology gene sets	HP ABNORMAL PROTEIN O LINKED GLYCOSYLATION	KIRC	1
c5: Ontology gene sets	HP ABNORMAL PR INTERVAL	THCA	1
c5: Ontology gene sets	HP ABNORMAL PLATELET GRANULES	THYM	1
c5: Ontology gene sets	HP ABNORMAL NATURAL KILLER CELL PHYSIOLOGY	UVM	1
c5: Ontology gene sets	HP ABNORMAL NASAL BASE	KIRC	1
c5: Ontology gene sets	HP ABNORMAL MUSCLE FIBER ALPHA DYSTROGLYCAN	MESO	1
c5: Ontology gene sets	HP ABNORMAL MORPHOLOGY OF THE CONJUNCTIVAL VASCULATURE	ACC	1

c5: Ontology gene sets	HP ABNORMAL MITOCHONDRIAL NUMBER	SARC	1
c5: Ontology gene sets	HP ABNORMAL MITOCHONDRIAL MORPHOLOGY	THYM	1
c5: Ontology gene sets	HP ABNORMAL FUNDUS AUTOFLUORESCENCE IMAGING	KICH	1
c5: Ontology gene sets	HP ABNORMAL FOOT BONE OSSIFICATION	KIRC	1
c5: Ontology gene sets	HP ABNORMAL ENERGY EXPENDITURE	BLCA	1
c5: Ontology gene sets	HP ABNORMAL DERMOEPIDERMAL JUNCTION MORPHOLOGY	PAAD	1
c5: Ontology gene sets	HP ABNORMAL DELAYED HYPERSENSITIVITY SKIN TEST	SKCM	1
c5: Ontology gene sets	HP ABNORMAL CUTANEOUS ELASTIC FIBER MORPHOLOGY	CESC	1
c5: Ontology gene sets	HP ABNORMAL CIRCULATING SULFUR AMINO ACID CONCENTRATION	LGG	1
c5: Ontology gene sets	HP ABNORMAL CIRCULATING LONG CHAIN FATTY ACID CONCENTRATION	KIRC	1
c5: Ontology gene sets	HP ABNORMAL CIRCULATING INHIBIN LEVEL	KIRP	1
c5: Ontology gene sets	HP ABNORMAL CIRCULATING AMYLASE LEVEL	KIRC	1
c5: Ontology gene sets	HP ABNORMAL CIRCULATING ACETYLCARNITINE CONCENTRATION	THYM	1
c5: Ontology gene sets	HP ABNORMAL CIRCLE OF WILLIS MORPHOLOGY	KIRC	1
c5: Ontology gene sets	HP ABNORMAL CHROMOSOME MORPHOLOGY	LGG	1

c5: Ontology gene sets	HP ABNORMAL BRAIN N ACETYL ASPARTATE LEVEL BY MRS	KIRC	1
c5: Ontology gene sets	HP ABNORMAL BLOOD PHOSPHATE CONCENTRATION	DLBC	1
c5: Ontology gene sets	HP ABNORMAL B CELL SUBSET DISTRIBUTION	LUAD	1
c5: Ontology gene sets	HP ABNORMAL ANTERIOR HORN CELL MORPHOLOGY	KIRC	1
c5: Ontology gene sets	HP ABNORMAL ALDOLASE LEVEL	MESO	1
c5: Ontology gene sets	HP ABNORMAL 5TH FINGER PHALANX MORPHOLOGY	READ	1
c5: Ontology gene sets	HP 4 5 TOE SYNDACTYLY	KIRC	1
c5: Ontology gene sets	HP 3 METHYLGLUTACONIC ACIDURIA	KIRC	1
c2: curated gene sets	HOWLIN CITED1 TARGETS 1 DN	READ	1
c7: immunologic signature gene sets	HOWARD B CELL INACT MONOV INFLUENZA A INDONESIA 05 2005 H5N1 AGE 18 49YO 1DY UP	UVM	1
c2: curated gene sets	HONRADO BREAST CANCER BRCA1 VS BRCA2	KIRC	1
c2: curated gene sets	HOLLEMAN PREDNISOLONE RESISTANCE B ALL UP	LIHC	1
c2: curated gene sets	HOLLEMAN PREDNISOLONE RESISTANCE ALL UP	THYM	1
c2: curated gene sets	HOLLEMAN PREDNISOLONE RESISTANCE ALL DN	PRAD	1
c2: curated gene sets	HOLLEMAN DAUNORUBICIN ALL UP	UCEC	1
c2: curated gene sets	HOLLEMAN DAUNORUBICIN ALL DN	READ	1
c2: curated gene sets	HOLLEMAN ASPARAGINASE RESISTANCE B ALL DN	KIRC	1

c7: immunologic signature gene sets	HOFT CD4 POSITIVE ALPHA BETA MEMORY T CELL BCG VACCINE AGE 18 45YO ID 7DY TOP 100 DEG EX VIVO DN	SKCM	1
c7: immunologic signature gene sets	HOFT CD4 POSITIVE ALPHA BETA MEMORY T CELL BCG VACCINE AGE 18 45YO 7DY DN	UVM	1
c7: immunologic signature gene sets	HOFT CD4 POSITIVE ALPHA BETA MEMORY T CELL BCG VACCINE AGE 18 45YO 56D TOP 100 DEG AFTER IN VITRO RE STIMULATION DN	READ	1
c2: curated gene sets	HOFMANN MYELODYSPLASTIC SYNDROME HIGH RISK UP	UVM	1
c7: immunologic signature gene sets	HOEK MYELOID DENDRITIC CELL 2011 2012 TIV ADULT 7DY UP	BRCA	1
c7: immunologic signature gene sets	HOEK MYELOID DENDRITIC CELL 2011 2012 TIV ADULT 1DY UP	SKCM	1
c7: immunologic signature gene sets	HOEK MONOCYTE 2011 2012 TIV ADULT 3DY DN	PAAD	1
c7: immunologic signature gene sets	HOEK B CELL 2011 2012 TIV ADULT 7DY DN	COAD	1
c7: immunologic signature gene sets	HOEK B CELL 2011 2012 TIV ADULT 3DY DN	LGG	1
c7: immunologic signature gene sets	HIPC SIGNATURES PROJECT PBMC TRIVALENT INFLUENZA VACCINE HIGH RESPONDERS VS LOW RESPONDERS YOUNGER ADULTS 21 35 HIGH RESPONDERS 0D UP	KIRC	1

c7: immunologic signature gene sets	HIPC SIGNATURES PROJECT PBMC TRIVALENT INFLUENZA VACCINE HIGH RESPONDERS VS LOW RESPONDERS YOUNGER ADULTS 21 35 HIGH RESPONDERS 0D DOWN	HNSC	1
c2: curated gene sets	HERNANDEZ ABERRANT MITOSIS BY DOCETACEL 4NM DN	SKCM	1
c2: curated gene sets	HE PTEN TARGETS DN	HNSC	1
c8: cell type signature gene sets	HAY BONE MARROW CD34 POS ERP	UCEC	1
c2: curated gene sets	HASINA NOL7 TARGETS DN	BLCA	1
c7: immunologic signature gene sets	HARALAMBIEVA PBMC TIV AGE 50 74YO CORRELATED WITH MEMORY B CELL RESPONSE 3DY POSITIVE	KIRC	1
c7: immunologic signature gene sets	HARALAMBIEVA PBMC FLUARIX AGE 50 74YO CORR WITH 28D MEM B CELL RESPONSE AT 28DY LEUK MIGR MAPK ACT CYTOK SIG DIAB OF THE YNG NEGATIVE	THYM	1
c7: immunologic signature gene sets	HARALAMBIEVA PBMC FLUARIX AGE 50 74YO CORR WITH 28D MEM B CELL RESPONSE AT 28DY LATE GENE EXPR INDIVID GENE MODELS PRED PEAK B CELL ELISPOT RESP NEGATIVE	LGG	1
c2: curated gene sets	HAHTOLA MYCOSIS FUNGOIDES DN	LGG	1
c2: curated gene sets	HAHTOLA CTCL PATHOGENESIS	UVM	1
c2: curated gene sets	HAHTOLA CTCL CUTANEOUS	THCA	1
c2: curated gene sets	HAEGERSTRAND RESPONSE TO IMATINIB	GBM	1

c2: curated gene sets	GUTIERREZ WALDENSTROEMS MACROGLOBULINEMIA 1 DN	HNSC	1
c2: curated gene sets	GUTIERREZ CHRONIC LYMPHOCYTIC LEUKEMIA UP	UVM	1
c2: curated gene sets	GU PDEF TARGETS DN	DLBC	1
c3: regulatory target gene sets	GTF3C1 TARGET GENES	READ	1
c3: regulatory target gene sets	GTCGATC MIR3695P	KIRC	1
c2: curated gene sets	GROSS HIF1A TARGETS UP	LUAD	1
c2: curated gene sets	GREENBAUM E2A TARGETS DN	PCPG	1
c2: curated gene sets	GRATIAS RETINOBLASTOMA 16Q24	LUAD	1
c2: curated gene sets	GRASEMANN RETINOBLASTOMA WITH 6P AMPLIFICATION	KICH	1
c2: curated gene sets	GRANDVAUX IRF3 TARGETS UP	SKCM	1
c2: curated gene sets	GRAHAM CML QUIESCENT VS CML DIVIDING UP	PRAD	1
c2: curated gene sets	GOUYER TUMOR INVASIVENESS	BLCA	1
c5: Ontology gene sets	GOMF VASCULAR ENDOTHELIAL GROWTH FACTOR ACTIVATED RECEPTOR ACTIVITY	KIRC	1
c5: Ontology gene sets	GOMF UNMETHYLATED CPG BINDING	THCA	1
c5: Ontology gene sets	GOMF UDP XYLOSYLTRANSFERASE ACTIVITY	KICH	1
c5: Ontology gene sets	GOMF UBIQUITIN UBIQUITIN LIGASE ACTIVITY	LIHC	1
c5: Ontology gene sets	GOMF UBIQUITIN SPECIFIC PROTEASE BINDING	THYM	1
c5: Ontology gene sets	GOMF UBIQUITIN DEPENDENT PROTEIN BINDING	COAD	1

c5: Ontology gene sets	GOMF TYPE II TRANSFORMING GROWTH FACTOR BETA RECEPTOR BINDING	KIRP	1
c5: Ontology gene sets	GOMF TYPE 2A SEROTONIN RECEPTOR BINDING	KIRC	1
c5: Ontology gene sets	GOMF TUMOR NECROSIS FACTOR ACTIVATED RECEPTOR ACTIVITY	COAD	1
c5: Ontology gene sets	GOMF TRIPLET CODON AMINO ACID ADAPTOR ACTIVITY	LUAD	1
c5: Ontology gene sets	GOMF TRIPEPTIDE TRANSMEMBRANE TRANSPORTER ACTIVITY	LUSC	1
c5: Ontology gene sets	GOMF TRANSMEMBRANE RECEPTOR PROTEIN PHOSPHATASE ACTIVITY	CESC	1
c5: Ontology gene sets	GOMF TRANSCRIPTION COREPRESSOR BINDING	THYM	1
c5: Ontology gene sets	GOMF TRAIL BINDING	LGG	1
c5: Ontology gene sets	GOMF THYROID HORMONE BINDING	LUAD	1
c5: Ontology gene sets	GOMF THROMBIN ACTIVATED RECEPTOR ACTIVITY	COAD	1
c5: Ontology gene sets	GOMF THREONINE TYPE PEPTIDASE ACTIVITY	READ	1
c5: Ontology gene sets	GOMF THIOREDOXIN PEROXIDASE ACTIVITY	LGG	1
c5: Ontology gene sets	GOMF THIOLESTER HYDROLASE ACTIVITY	READ	1
c5: Ontology gene sets	GOMF THIAMINE PYROPHOSPHATE BINDING	KIRC	1
c5: Ontology gene sets	GOMF TBP CLASS PROTEIN BINDING	THYM	1
c5: Ontology gene sets	GOMF SYNDECAN BINDING	LGG	1

c5: Ontology gene sets	GOMF SUPEROXIDE GENERATING NAD P H OXIDASE ACTIVITY	DLBC	1
c5: Ontology gene sets	GOMF SUMO SPECIFIC PROTEASE ACTIVITY	THYM	1
c5: Ontology gene sets	GOMF STRUCTURAL MOLECULE ACTIVITY CONFERRING ELASTICITY	ACC	1
c5: Ontology gene sets	GOMF STRUCTURAL CONSTITUENT OF POSTSYNAPTIC ACTIN CYTOSKELETON	BRCA	1
c5: Ontology gene sets	GOMF STRUCTURAL CONSTITUENT OF MYELIN SHEATH	TGCT	1
c5: Ontology gene sets	GOMF STEROID DEHYDROGENASE ACTIVITY	DLBC	1
c5: Ontology gene sets	GOMF STAT FAMILY PROTEIN BINDING	BRCA	1
c5: Ontology gene sets	GOMF SPHINGOSINE 1 PHOSPHATE PHOSPHATASE ACTIVITY	BRCA	1
c5: Ontology gene sets	GOMF SPHINGOLIPID TRANSFER ACTIVITY	THCA	1
c5: Ontology gene sets	GOMF SODIUM PHOSPHATE SYMPORTER ACTIVITY	KIRC	1
c5: Ontology gene sets	GOMF SNRNP BINDING	KIRC	1
c5: Ontology gene sets	GOMF SMALL RIBOSOMAL SUBUNIT RRNA BINDING	UVM	1
c5: Ontology gene sets	GOMF SINGLE STRANDED TELOMERIC DNA BINDING	PRAD	1
c5: Ontology gene sets	GOMF SIGNAL RECOGNITION PARTICLE BINDING	KIRC	1
c5: Ontology gene sets	GOMF SEQUENCE SPECIFIC SINGLE STRANDED DNA BINDING	PRAD	1
c5: Ontology gene sets	GOMF S100 PROTEIN BINDING	PAAD	1

c5: Ontology gene sets	GOMF S ADENOSYL L METHIONINE BINDING	THYM	1
c5: Ontology gene sets	GOMF ROUNDABOUT BINDING	LUAD	1
c5: Ontology gene sets	GOMF RNA STRAND ANNEALING ACTIVITY	KIRC	1
c5: Ontology gene sets	GOMF RNA STEM LOOP BINDING	THYM	1
c5: Ontology gene sets	GOMF RNA POLYMERASE III GENERAL TRANSCRIPTION INITIATION FACTOR ACTIVITY	SKCM	1
c5: Ontology gene sets	GOMF RNA POLYMERASE II REPRESSING TRANSCRIPTION FACTOR BINDING	THYM	1
c5: Ontology gene sets	GOMF RNA POLYMERASE II CORE PROMOTER SEQUENCE SPECIFIC DNA BINDING	THCA	1
c5: Ontology gene sets	GOMF RNA POLYMERASE II C TERMINAL DOMAIN PHOSPHOSERINE BINDING	THYM	1
c5: Ontology gene sets	GOMF RNA POLYMERASE I CORE BINDING	READ	1
c5: Ontology gene sets	GOMF RNA 7 METHYLGUANOSINE CAP BINDING	BRCA	1
c5: Ontology gene sets	GOMF RNA 2 O METHYLTRANSFERASE ACTIVITY	LIHC	1
c5: Ontology gene sets	GOMF RETROMER COMPLEX BINDING	KIRC	1
c5: Ontology gene sets	GOMF PYRIMIDINE NUCLEOTIDE SUGAR TRANSMEMBRANE TRANSPORTER ACTIVITY	KIRC	1
c5: Ontology gene sets	GOMF PURINE DEOXYRIBONUCLEOTIDE BINDING	SKCM	1

c5: Ontology gene sets	GOMF PROTEIN TYROSINE KINASE ACTIVATOR ACTIVITY	KIRC	1
c5: Ontology gene sets	GOMF PROTEIN PRENYLTRANSFERASE ACTIVITY	KIRC	1
c5: Ontology gene sets	GOMF PROTEIN KINASE C ACTIVITY	THYM	1
c5: Ontology gene sets	GOMF PROTEIN DEMETHYLASE ACTIVITY	PRAD	1
c5: Ontology gene sets	GOMF PROTEIN CARBOXYL O METHYLTRANSFERASE ACTIVITY	LGG	1
c5: Ontology gene sets	GOMF PROSTANOID RECEPTOR ACTIVITY	BRCA	1
c5: Ontology gene sets	GOMF PRENYLTRANSFERASE ACTIVITY	KICH	1
c5: Ontology gene sets	GOMF PRE MRNA INTRONIC BINDING	THYM	1
c5: Ontology gene sets	GOMF PRE MRNA 3 SPLICE SITE BINDING	PAAD	1
c5: Ontology gene sets	GOMF POTASSIUM ION ANTIPORTER ACTIVITY	THYM	1
c5: Ontology gene sets	GOMF POTASSIUM CHLORIDE SYMPORTER ACTIVITY	UCEC	1
c5: Ontology gene sets	GOMF POLYPEPTIDE N ACETYLGALACTOSAMINYLTRANSFERASE ACTIVITY	PRAD	1
c5: Ontology gene sets	GOMF PLATELET DERIVED GROWTH FACTOR BINDING	KIRP	1
c5: Ontology gene sets	GOMF PHOSPHOTRANSFERASE ACTIVITY NITROGENOUS GROUP AS ACCEPTOR	KICH	1
c5: Ontology gene sets	GOMF PHOSPHOSERINE RESIDUE BINDING	THYM	1

c5: Ontology gene sets	GOMF PHOSPHOLIPASE A2 INHIBITOR ACTIVITY	HNSC	1
c5: Ontology gene sets	GOMF PHOSPHOLIPASE A2 ACTIVITY CONSUMING 1 2 DIPALMITOYLPHOSPHATIDYLCHOLINE	HNSC	1
c5: Ontology gene sets	GOMF PHOSPHATIDYLSERINE FLIPPASE ACTIVITY	LGG	1
c5: Ontology gene sets	GOMF PHOSPHATIDYLINOSITOL PHOSPHATE KINASE ACTIVITY	READ	1
c5: Ontology gene sets	GOMF PHOSPHATIDYLINOSITOL 4 5 BISPHOSPHATE PHOSPHATASE ACTIVITY	MESO	1
c5: Ontology gene sets	GOMF PHOSPHATIDYLINOSITOL 4 5 BISPHOSPHATE 5 PHOSPHATASE ACTIVITY	MESO	1
c5: Ontology gene sets	GOMF PHOSPHATIDYLETHANOLAMINE FLIPPASE ACTIVITY	CESC	1
c5: Ontology gene sets	GOMF PHOSPHATIDYLCHOLINE FLIPPASE ACTIVITY	UVM	1
c5: Ontology gene sets	GOMF PHOSPHATIDIC ACID TRANSFER ACTIVITY	KIRC	1
c5: Ontology gene sets	GOMF PEROXISOME TARGETING SEQUENCE BINDING	KIRC	1
c5: Ontology gene sets	GOMF PEPTIDYL PROLINE DIOXYGENASE ACTIVITY	CESC	1
c5: Ontology gene sets	GOMF PEPTIDYL PROLINE 4 DIOXYGENASE ACTIVITY	CESC	1
c5: Ontology gene sets	GOMF PEPTIDE ALPHA N ACETYLTRANSFERASE ACTIVITY	KIRC	1
c5: Ontology gene sets	GOMF PALMITOYL PROTEIN HYDROLASE ACTIVITY	LUSC	1

c5: Ontology gene sets	GOMF OXIDOREDUCTASE ACTIVITY ACTING ON THE CH CH GROUP OF DONORS NAD OR NADP AS ACCEPTOR	DLBC	1
c5: Ontology gene sets	GOMF OXIDOREDUCTASE ACTIVITY ACTING ON THE CH CH GROUP OF DONORS	DLBC	1
c5: Ontology gene sets	GOMF OXIDOREDUCTASE ACTIVITY ACTING ON PAIRED DONORS WITH OXIDATION OF A PAIR OF DONORS RESULTING IN THE REDUCTION OF MOLECULAR OXYGEN TO TWO MOLECULES OF WATER	LGG	1
c5: Ontology gene sets	GOMF OXIDOREDUCTASE ACTIVITY ACTING ON OTHER NITROGENOUS COMPOUNDS AS DONORS CYTOCHROME AS ACCEPTOR	LIHC	1
c5: Ontology gene sets	GOMF OXIDOREDUCTASE ACTIVITY ACTING ON OTHER NITROGENOUS COMPOUNDS AS DONORS	LIHC	1
c5: Ontology gene sets	GOMF OXIDOREDUCTASE ACTIVITY ACTING ON A SULFUR GROUP OF DONORS NAD P AS ACCEPTOR	TGCT	1
c5: Ontology gene sets	GOMF OXIDOREDUCTASE ACTIVITY ACTING ON A SULFUR GROUP OF DONORS DISULFIDE AS ACCEPTOR	KIRP	1
c5: Ontology gene sets	GOMF OXIDIZED DNA BINDING	THYM	1
c5: Ontology gene sets	GOMF OUTWARD RECTIFIER POTASSIUM CHANNEL ACTIVITY	STAD	1

c5: Ontology gene sets	GOMF ORNITHINE DECARBOXYLASE REGULATOR ACTIVITY	PAAD	1
c5: Ontology gene sets	GOMF OLIGOSACCHARYL TRANSFERASE ACTIVITY	LGG	1
c5: Ontology gene sets	GOMF O PALMITOYLTRANSFERASE ACTIVITY	KIRC	1
c5: Ontology gene sets	GOMF O METHYLTRANSFERASE ACTIVITY	KICH	1
c5: Ontology gene sets	GOMF NUCLEOTIDE SUGAR TRANSMEMBRANE TRANSPORTER ACTIVITY	KIRC	1
c5: Ontology gene sets	GOMF NUCLEOTIDE DIPHOSPHATASE ACTIVITY	KIRC	1
c5: Ontology gene sets	GOMF NUCLEOSIDE TRIPHOSPHATE DIPHOSPHATASE ACTIVITY	KIRC	1
c5: Ontology gene sets	GOMF NUCLEOCYTOPLASMIC CARRIER ACTIVITY	THYM	1
c5: Ontology gene sets	GOMF NUCLEAR LOCALIZATION SEQUENCE BINDING	PRAD	1
c5: Ontology gene sets	GOMF NUCLEAR IMPORT SIGNAL RECEPTOR ACTIVITY	PRAD	1
c5: Ontology gene sets	GOMF NITRITE REDUCTASE ACTIVITY	LIHC	1
c5: Ontology gene sets	GOMF NITRIC OXIDE SYNTHASE REGULATOR ACTIVITY	LIHC	1
c5: Ontology gene sets	GOMF NEUROTROPHIN RECEPTOR BINDING	KIRC	1
c5: Ontology gene sets	GOMF NATURAL KILLER CELL LECTIN LIKE RECEPTOR BINDING	BRCA	1
c5: Ontology gene sets	GOMF NADPLUS BINDING	KIRC	1
c5: Ontology gene sets	GOMF NADPH BINDING	KIRC	1
c5: Ontology gene sets	GOMF NADP BINDING	DLBC	1

c5: Ontology gene sets	GOMF NADH PYROPHOSPHATASE ACTIVITY	KIRC	1
c5: Ontology gene sets	GOMF NAD P H OXIDASE H2O2 FORMING ACTIVITY	BRCA	1
c5: Ontology gene sets	GOMF N ACETYLLACTOSAMINE SYNTHASE ACTIVITY	UVM	1
c5: Ontology gene sets	GOMF N ACETYLGALACTOSAMINE 4 O SULFOTRANSFERASE ACTIVITY	COAD	1
c5: Ontology gene sets	GOMF MYRISTOYLTRANSFERASE ACTIVITY	KIRC	1
c5: Ontology gene sets	GOMF MYOSIN V BINDING	KIRC	1
c5: Ontology gene sets	GOMF MYOSIN HEAVY CHAIN BINDING	KIRC	1
c5: Ontology gene sets	GOMF MUTSALPHA COMPLEX BINDING	KICH	1
c5: Ontology gene sets	GOMF MRNA 5 UTR BINDING	THCA	1
c5: Ontology gene sets	GOMF MONOAMINE TRANSMEMBRANE TRANSPORTER ACTIVITY	COAD	1
c5: Ontology gene sets	GOMF MOLYBDOPTERIN COFACTOR BINDING	PRAD	1
c5: Ontology gene sets	GOMF MISMATCH REPAIR COMPLEX BINDING	KICH	1
c5: Ontology gene sets	GOMF MIRNA BINDING	THYM	1
c5: Ontology gene sets	GOMF MICROTUBULE PLUS END BINDING	THYM	1
c5: Ontology gene sets	GOMF MHC CLASS II PROTEIN BINDING	SKCM	1
c5: Ontology gene sets	GOMF MHC CLASS IB RECEPTOR ACTIVITY	SKCM	1
c5: Ontology gene sets	GOMF MHC CLASS I PROTEIN COMPLEX BINDING	SKCM	1

c5: Ontology gene sets	GOMF METALLOENDOPEPTIDASE INHIBITOR ACTIVITY	SKCM	1
c5: Ontology gene sets	GOMF MEMBRANE INSERTASE ACTIVITY	ESCA	1
c5: Ontology gene sets	GOMF LYSOPHOSPHATIDIC ACID BINDING	UCEC	1
c5: Ontology gene sets	GOMF LYSINE N ACETYLTRANSFERASE ACTIVITY ACTING ON ACETYL PHOSPHATE AS DONOR	KIRC	1
c5: Ontology gene sets	GOMF LIM DOMAIN BINDING	KIRC	1
c5: Ontology gene sets	GOMF LIGASE ACTIVITY FORMING CARBON CARBON BONDS	KIRP	1
c5: Ontology gene sets	GOMF LBD DOMAIN BINDING	KIRC	1
c5: Ontology gene sets	GOMF LARGE RIBOSOMAL SUBUNIT RRNA BINDING	KIRP	1
c5: Ontology gene sets	GOMF L LYSINE TRANSMEMBRANE TRANSPORTER ACTIVITY	UVM	1
c5: Ontology gene sets	GOMF L HISTIDINE TRANSMEMBRANE TRANSPORTER ACTIVITY	MESO	1
c5: Ontology gene sets	GOMF L ASCORBIC ACID BINDING	THCA	1
c5: Ontology gene sets	GOMF L ARGININE TRANSMEMBRANE TRANSPORTER ACTIVITY	LGG	1
c5: Ontology gene sets	GOMF KINESIN BINDING	THYM	1
c5: Ontology gene sets	GOMF JUN KINASE BINDING	KIRC	1
c5: Ontology gene sets	GOMF IRON ION TRANSMEMBRANE TRANSPORTER ACTIVITY	UCEC	1
c5: Ontology gene sets	GOMF INTRACILIARY TRANSPORT PARTICLE B BINDING	KIRC	1
c5: Ontology gene sets	GOMF INTERLEUKIN 2 RECEPTOR BINDING	LGG	1
c5: Ontology gene sets	GOMF INTERLEUKIN 17 RECEPTOR ACTIVITY	THYM	1

c5: Ontology gene sets	GOMF INSULIN LIKE GROWTH FACTOR II BINDING	KIRC	1
c5: Ontology gene sets	GOMF INOSITOL TRISPHOSPHATE PHOSPHATASE ACTIVITY	MESO	1
c5: Ontology gene sets	GOMF INOSITOL PHOSPHATE PHOSPHATASE ACTIVITY	LGG	1
c5: Ontology gene sets	GOMF INOSITOL HEXAKISPHOSPHATE KINASE ACTIVITY	UVM	1
c5: Ontology gene sets	GOMF INOSITOL HEXAKISPHOSPHATE 5 KINASE ACTIVITY	UVM	1
c5: Ontology gene sets	GOMF IMPORTIN ALPHA FAMILY PROTEIN BINDING	THYM	1
c5: Ontology gene sets	GOMF ICOSANOID RECEPTOR ACTIVITY	BRCA	1
c5: Ontology gene sets	GOMF ICOSANOID BINDING	LGG	1
c5: Ontology gene sets	GOMF HISTONE THREONINE KINASE ACTIVITY	COAD	1
c5: Ontology gene sets	GOMF HISTONE SERINE KINASE ACTIVITY	KIRC	1
c5: Ontology gene sets	GOMF HISTONE METHYLTRANSFERASE ACTIVITY H3 K9 SPECIFIC	LIHC	1
c5: Ontology gene sets	GOMF HISTONE DEACETYLASE ACTIVITY H3 K14 SPECIFIC	THCA	1
c5: Ontology gene sets	GOMF HISTONE ACETYLTRANSFERASE BINDING	THYM	1
c5: Ontology gene sets	GOMF HISTAMINE RECEPTOR ACTIVITY	HNSC	1
c5: Ontology gene sets	GOMF HEME TRANSMEMBRANE TRANSPORTER ACTIVITY	KICH	1
c5: Ontology gene sets	GOMF GUANYLYLTRANSFERASE ACTIVITY	KICH	1

c5: Ontology gene sets	GOMF GPI LINKED EPHRIN RECEPTOR ACTIVITY	BLCA	1
c5: Ontology gene sets	GOMF GLYCOLIPID MANNOSYLTRANSFERASE ACTIVITY	KIRC	1
c5: Ontology gene sets	GOMF GLUTATHIONE DISULFIDE OXIDOREDUCTASE ACTIVITY	UVM	1
c5: Ontology gene sets	GOMF GLUTAMATE GATED CALCIUM ION CHANNEL ACTIVITY	UCS	1
c5: Ontology gene sets	GOMF GLUTAMATE BINDING	UCEC	1
c5: Ontology gene sets	GOMF GAP JUNCTION CHANNEL ACTIVITY INVOLVED IN CELL COMMUNICATION BY ELECTRICAL COUPLING	BLCA	1
c5: Ontology gene sets	GOMF GANGLIOSIDE BINDING	UVM	1
c5: Ontology gene sets	GOMF GABA RECEPTOR BINDING	KIRC	1
c5: Ontology gene sets	GOMF G RICH STRAND TELOMERIC DNA BINDING	PRAD	1
c5: Ontology gene sets	GOMF G PROTEIN GAMMA SUBUNIT BINDING	PAAD	1
c5: Ontology gene sets	GOMF G PROTEIN COUPLED ADENOSINE RECEPTOR ACTIVITY	SKCM	1
c5: Ontology gene sets	GOMF FUCOSE BINDING	BRCA	1
c5: Ontology gene sets	GOMF FRUCTOSE BINDING	CHOL	1
c5: Ontology gene sets	GOMF FRUCTOSE 6 PHOSPHATE BINDING	COAD	1
c5: Ontology gene sets	GOMF FOUR WAY JUNCTION HELICASE ACTIVITY	LIHC	1
c5: Ontology gene sets	GOMF FOUR WAY JUNCTION DNA BINDING	LIHC	1
c5: Ontology gene sets	GOMF FLAP ENDONUCLEASE ACTIVITY	KIRC	1
c5: Ontology gene sets	GOMF FK506 BINDING	PCPG	1

c5: Ontology gene sets	GOMF FATTY ACID OMEGA HYDROXYLASE ACTIVITY	KIRC	1
c5: Ontology gene sets	GOMF ERBB 3 CLASS RECEPTOR BINDING	BRCA	1
c5: Ontology gene sets	GOMF EPOXIDE HYDROLASE ACTIVITY	LGG	1
c5: Ontology gene sets	GOMF ENDODEOXYRIBONUCLEASE ACTIVITY PRODUCING 3 PHOSPHOMONOESTERS	KICH	1
c5: Ontology gene sets	GOMF DYNEIN LIGHT CHAIN BINDING	CESC	1
c5: Ontology gene sets	GOMF DOUBLE STRANDED METHYLATED DNA BINDING	KIRC	1
c5: Ontology gene sets	GOMF DOLICHYL PHOSPHATE MANNOSE PROTEIN MANNOSYLTRANSFERASE ACTIVITY	KIRC	1
c5: Ontology gene sets	GOMF DNA POLYMERASE ACTIVITY	THYM	1
c5: Ontology gene sets	GOMF DNA METHYLTRANSFERASE ACTIVITY	LIHC	1
c5: Ontology gene sets	GOMF DNA DIRECTED DNA POLYMERASE ACTIVITY	READ	1
c5: Ontology gene sets	GOMF DNA CLAMP LOADER ACTIVITY	LIHC	1
c5: Ontology gene sets	GOMF DNA BINDING BENDING	KIRC	1
c5: Ontology gene sets	GOMF DEMETHYLASE ACTIVITY	PRAD	1
c5: Ontology gene sets	GOMF CYTOSKELETAL ANCHOR ACTIVITY	DLBC	1
c5: Ontology gene sets	GOMF CYTOCHROME B5 REDUCTASE ACTIVITY ACTING ON NAD P H	KIRC	1

c5: Ontology gene sets	GOMF CYTIDINE DEAMINASE ACTIVITY	CESC	1
c5: Ontology gene sets	GOMF CYCLOHYDROLASE ACTIVITY	BLCA	1
c5: Ontology gene sets	GOMF CYCLIN DEPENDENT PROTEIN SERINE THREONINE KINASE ACTIVATOR ACTIVITY	LIHC	1
c5: Ontology gene sets	GOMF CYCLIC NUCLEOTIDE DEPENDENT PROTEIN KINASE ACTIVITY	READ	1
c5: Ontology gene sets	GOMF CXCR3 CHEMOKINE RECEPTOR BINDING	SKCM	1
c5: Ontology gene sets	GOMF CORTICOTROPIN RELEASING HORMONE RECEPTOR BINDING	PAAD	1
c5: Ontology gene sets	GOMF CORECEPTOR ACTIVITY INVOLVED IN WNT SIGNALING PATHWAY PLANAR CELL POLARITY PATHWAY	LGG	1
c5: Ontology gene sets	GOMF COLLAGEN RECEPTOR ACTIVITY	KIRP	1
c5: Ontology gene sets	GOMF COA TRANSFERASE ACTIVITY	CESC	1
c5: Ontology gene sets	GOMF CO SMAD BINDING	THYM	1
c5: Ontology gene sets	GOMF CO RECEPTOR BINDING	BRCA	1
c5: Ontology gene sets	GOMF CILIARY NEUROTROPHIC FACTOR RECEPTOR ACTIVITY	BRCA	1
c5: Ontology gene sets	GOMF CHROMO SHADOW DOMAIN BINDING	SKCM	1
c5: Ontology gene sets	GOMF CHLORIDE CHANNEL INHIBITOR ACTIVITY	LUSC	1
c5: Ontology gene sets	GOMF CERAMIDE 1 PHOSPHATE TRANSFER ACTIVITY	THCA	1

c5: Ontology gene sets	GOMF CENTROMERIC DNA BINDING	MESO	1
c5: Ontology gene sets	GOMF CELL MATRIX ADHESION MEDIATOR ACTIVITY	LGG	1
c5: Ontology gene sets	GOMF CELL ADHESIVE PROTEIN BINDING INVOLVED IN BUNDLE OF HIS CELL PURKINJE MYOCYTE COMMUNICATION	KIRC	1
c5: Ontology gene sets	GOMF CD4 RECEPTOR BINDING	UCEC	1
c5: Ontology gene sets	GOMF CCR5 CHEMOKINE RECEPTOR BINDING	DLBC	1
c5: Ontology gene sets	GOMF CARNITINE O ACYLTRANSFERASE ACTIVITY	KIRC	1
c5: Ontology gene sets	GOMF CAMP DEPENDENT PROTEIN KINASE ACTIVITY	READ	1
c5: Ontology gene sets	GOMF CALCIUM DEPENDENT PROTEIN KINASE ACTIVITY	THYM	1
c5: Ontology gene sets	GOMF BUBBLE DNA BINDING	LIHC	1
c5: Ontology gene sets	GOMF BRANCHED CHAIN AMINO ACID TRANSMEMBRANE TRANSPORTER ACTIVITY	LUSC	1
c5: Ontology gene sets	GOMF BIOTIN BINDING	LGG	1
c5: Ontology gene sets	GOMF BETA 1 ADRENERGIC RECEPTOR BINDING	SKCM	1
c5: Ontology gene sets	GOMF BASIC AMINO ACID TRANSMEMBRANE TRANSPORTER ACTIVITY	BRCA	1
c5: Ontology gene sets	GOMF ATPASE INHIBITOR ACTIVITY	OV	1
c5: Ontology gene sets	GOMF ASPARTIC ENDOPEPTIDASE ACTIVITY INTRAMEMBRANE CLEAVING	KIRP	1

c5: Ontology gene sets	GOMF ARGININE TRANSMEMBRANE TRANSPORTER ACTIVITY	BRCA	1
c5: Ontology gene sets	GOMF ARACHIDONIC ACID EPOXYGENASE ACTIVITY	LUSC	1
c5: Ontology gene sets	GOMF APOLIPOPROTEIN A I BINDING	LUAD	1
c5: Ontology gene sets	GOMF AP 2 ADAPTOR COMPLEX BINDING	KIRC	1
c5: Ontology gene sets	GOMF ANNEALING HELICASE ACTIVITY	LGG	1
c5: Ontology gene sets	GOMF ANAPHASE PROMOTING COMPLEX BINDING	LGG	1
c5: Ontology gene sets	GOMF ALPHA N ACETYLGALACTOSAMINIDE ALPHA 2 6 SIALYLTRANSFERASE ACTIVITY	UCEC	1
c5: Ontology gene sets	GOMF ALKANE 1 MONOOXYGENASE ACTIVITY	HNSC	1
c5: Ontology gene sets	GOMF ADENYLATE CYCLASE BINDING	KIRP	1
c5: Ontology gene sets	GOMF ADENYLATE CYCLASE ACTIVITY	LUAD	1
c5: Ontology gene sets	GOMF ADENYLATE CYCLASE ACTIVATOR ACTIVITY	HNSC	1
c5: Ontology gene sets	GOMF ACYL COA OXIDASE ACTIVITY	KIRC	1
c5: Ontology gene sets	GOMF ACID PHOSPHATASE ACTIVITY	LGG	1
c5: Ontology gene sets	GOMF ABC TYPE GLUTATHIONE S CONJUGATE TRANSPORTER ACTIVITY	LIHC	1
c5: Ontology gene sets	GOMF 5 3 EXODEOXYRIBONUCLEASE ACTIVITY	KIRP	1

c5: Ontology gene sets	GOMF 3 BETA HYDROXY DELTA5 STEROID DEHYDROGENASE ACTIVITY	LGG	1
c5: Ontology gene sets	GOMF 3 5 EXODEOXYRIBONUCLEASE ACTIVITY	KIRP	1
c5: Ontology gene sets	GOMF 1 PHOSPHATIDYLINOSITOL 4 PHOSPHATE 3 KINASE ACTIVITY	READ	1
c5: Ontology gene sets	GOMF 1 PHOSPHATIDYLINOSITOL 4 KINASE ACTIVITY	DLBC	1
c2: curated gene sets	GOLUB ALL VS AML UP	BRCA	1
c2: curated gene sets	GOERING BLOOD HDL CHOLESTEROL QTL CIS	BRCA	1
c5: Ontology gene sets	GOCC ZONULA ADHERENS	KIRC	1
c5: Ontology gene sets	GOCC XY BODY	PRAD	1
c5: Ontology gene sets	GOCC WNT SIGNALOSOME	KIRC	1
c5: Ontology gene sets	GOCC WASH COMPLEX	KIRC	1
c5: Ontology gene sets	GOCC VESICLE COAT	DLBC	1
c5: Ontology gene sets	GOCC UNCONVENTIONAL MYOSIN COMPLEX	OV	1
c5: Ontology gene sets	GOCC UBIQUITIN CONJUGATING ENZYME COMPLEX	ESCA	1
c5: Ontology gene sets	GOCC U7 SNRNP	KIRP	1
c5: Ontology gene sets	GOCC U6 SNRNP	ACC	1
c5: Ontology gene sets	GOCC U2AF COMPLEX	PAAD	1
c5: Ontology gene sets	GOCC U2 TYPE CATALYTIC STEP 2 SPLICEOSOME	THYM	1
c5: Ontology gene sets	GOCC TUBULAR ENDOSOME	LGG	1
c5: Ontology gene sets	GOCC TRNA SPLICING LIGASE COMPLEX	THYM	1
c5: Ontology gene sets	GOCC TRICARBOXYLIC ACID CYCLE ENZYME COMPLEX	KIRC	1
c5: Ontology gene sets	GOCC TRANSLOCON COMPLEX	KIRC	1

c5: Ontology gene sets	GOCC TRANSCRIPTION FACTOR TFIIIC COMPLEX	LIHC	1
c5: Ontology gene sets	GOCC TRANSCRIPTION EXPORT COMPLEX 2	LIHC	1
c5: Ontology gene sets	GOCC THO COMPLEX	LIHC	1
c5: Ontology gene sets	GOCC TETRASPANIN ENRICHED MICRODOMAIN	THCA	1
c5: Ontology gene sets	GOCC TERTIARY GRANULE LUMEN	DLBC	1
c5: Ontology gene sets	GOCC SUPER ELONGATION COMPLEX	READ	1
c5: Ontology gene sets	GOCC SUMO LIGASE COMPLEX	LGG	1
c5: Ontology gene sets	GOCC SPERMATOPROTEASOME COMPLEX	TGCT	1
c5: Ontology gene sets	GOCC SPERM FIBROUS SHEATH	KIRC	1
c5: Ontology gene sets	GOCC SORTING ENDOSOME	KIRC	1
c5: Ontology gene sets	GOCC SMOOTH ENDOPLASMIC RETICULUM MEMBRANE	KIRC	1
c5: Ontology gene sets	GOCC SIN3 TYPE COMPLEX	THYM	1
c5: Ontology gene sets	GOCC SIN3 COMPLEX	THYM	1
c5: Ontology gene sets	GOCC SIGNAL PEPTIDASE COMPLEX	HNSC	1
c5: Ontology gene sets	GOCC SEX CHROMOSOME	BRCA	1
c5: Ontology gene sets	GOCC SCAR COMPLEX	UVM	1
c5: Ontology gene sets	GOCC SAGA COMPLEX	KICH	1
c5: Ontology gene sets	GOCC RNA POLYMERASE II TRANSCRIPTION REPRESSOR COMPLEX	KIRC	1
c5: Ontology gene sets	GOCC RIPOPTOSOME	LGG	1
c5: Ontology gene sets	GOCC PTW PP1 PHOSPHATASE COMPLEX	ACC	1
c5: Ontology gene sets	GOCC PROTON TRANSPORTING V TYPE ATPASE V1 DOMAIN	UCEC	1

c5: Ontology gene sets	GOCC PROTEASOME REGULATORY PARTICLE BASE SUBCOMPLEX	LIHC	1
c5: Ontology gene sets	GOCC PROTEASOME ACCESSORY COMPLEX	LIHC	1
c5: Ontology gene sets	GOCC PRESYNAPTIC CYTOSOL	KIRC	1
c5: Ontology gene sets	GOCC PRERIBOSOME LARGE SUBUNIT PRECURSOR	LIHC	1
c5: Ontology gene sets	GOCC POSTSYNAPTIC RECYCLING ENDOSOME	KIRC	1
c5: Ontology gene sets	GOCC POSTSYNAPTIC GOLGI APPARATUS	KIRC	1
c5: Ontology gene sets	GOCC POSTSYNAPTIC ENDOCYTIC ZONE	LGG	1
c5: Ontology gene sets	GOCC PODOSOME	THCA	1
c5: Ontology gene sets	GOCC PINOSOME	UVM	1
c5: Ontology gene sets	GOCC PICLN SM PROTEIN COMPLEX	ACC	1
c5: Ontology gene sets	GOCC PHOTORECEPTOR RIBBON SYNAPSE	COAD	1
c5: Ontology gene sets	GOCC PERICENTRIOLAR MATERIAL	THYM	1
c5: Ontology gene sets	GOCC PERICENTRIC HETEROCHROMATIN	THYM	1
c5: Ontology gene sets	GOCC PEPTIDASE INHIBITOR COMPLEX	MESO	1
c5: Ontology gene sets	GOCC PCAF COMPLEX	READ	1
c5: Ontology gene sets	GOCC OXOGLUTARATE DEHYDROGENASE COMPLEX	KIRC	1
c5: Ontology gene sets	GOCC OUTER DYNEIN ARM	THCA	1
c5: Ontology gene sets	GOCC OLIGOSACCHARYLTRANSFERASE COMPLEX	LGG	1
c5: Ontology gene sets	GOCC NUCLEOTIDE EXCISION REPAIR COMPLEX	KIRC	1

c5: Ontology gene sets	GOCC NUCLEOLAR RIBONUCLEASE P COMPLEX	KICH	1
c5: Ontology gene sets	GOCC NUCLEAR STRESS GRANULE	HNSC	1
c5: Ontology gene sets	GOCC NUCLEAR COHESIN COMPLEX	ACC	1
c5: Ontology gene sets	GOCC NONHOMOLOGOUS END JOINING COMPLEX	DLBC	1
c5: Ontology gene sets	GOCC MYELIN SHEATH	DLBC	1
c5: Ontology gene sets	GOCC MSL COMPLEX	CESC	1
c5: Ontology gene sets	GOCC MRNA CLEAVAGE AND POLYADENYLATION SPECIFICITY FACTOR COMPLEX	MESO	1
c5: Ontology gene sets	GOCC MON1 CCZ1 COMPLEX	UCEC	1
c5: Ontology gene sets	GOCC MITOTIC SPINDLE POLE	THYM	1
c5: Ontology gene sets	GOCC MITOTIC SPINDLE MIDZONE	PAAD	1
c5: Ontology gene sets	GOCC MITOTIC SPINDLE MICROTUBULE	LIHC	1
c5: Ontology gene sets	GOCC MITOTIC SPINDLE ASTRAL MICROTUBULE	THYM	1
c5: Ontology gene sets	GOCC MITOCHONDRIAL TRICARBOXYLIC ACID CYCLE ENZYME COMPLEX	THYM	1
c5: Ontology gene sets	GOCC MITOCHONDRIAL OUTER MEMBRANE TRANSLOCASE COMPLEX	ACC	1
c5: Ontology gene sets	GOCC MICROTUBULE PLUS END	THYM	1
c5: Ontology gene sets	GOCC MICROTUBULE END	THYM	1
c5: Ontology gene sets	GOCC MICROFIBRIL	KIRC	1
c5: Ontology gene sets	GOCC MHC PROTEIN COMPLEX	UVM	1
c5: Ontology gene sets	GOCC METHYLOSOME	ACC	1

c5: Ontology gene sets	GOCC MESSENGER RIBONUCLEOPROTEIN COMPLEX	KIRC	1
c5: Ontology gene sets	GOCC MEMBRANE ATTACK COMPLEX	LIHC	1
c5: Ontology gene sets	GOCC MEIOTIC COHESIN COMPLEX	ACC	1
c5: Ontology gene sets	GOCC LEWY BODY	KIRP	1
c5: Ontology gene sets	GOCC LATERAL ELEMENT	ACC	1
c5: Ontology gene sets	GOCC KICSTOR COMPLEX	LUAD	1
c5: Ontology gene sets	GOCC INTRINSIC COMPONENT OF THE CYTOPLASMIC SIDE OF THE PLASMA MEMBRANE	BLCA	1
c5: Ontology gene sets	GOCC INTRINSIC COMPONENT OF NUCLEAR INNER MEMBRANE	UVM	1
c5: Ontology gene sets	GOCC INTRACILIARY TRANSPORT PARTICLE B	KIRC	1
c5: Ontology gene sets	GOCC INTEGRATOR COMPLEX	KIRC	1
c5: Ontology gene sets	GOCC INSULIN RESPONSIVE COMPARTMENT	KIRC	1
c5: Ontology gene sets	GOCC IGA IMMUNOGLOBULIN COMPLEX	HNSC	1
c5: Ontology gene sets	GOCC HULC COMPLEX	ESCA	1
c5: Ontology gene sets	GOCC HOPS COMPLEX	KIRC	1
c5: Ontology gene sets	GOCC GOLGI MEDIAL CISTERNA	KIRC	1
c5: Ontology gene sets	GOCC GEMINI OF COILED BODIES	KIRC	1
c5: Ontology gene sets	GOCC GATOR1 COMPLEX	PAAD	1
c5: Ontology gene sets	GOCC GAMMA TUBULIN LARGE COMPLEX	LUAD	1
c5: Ontology gene sets	GOCC GAMMA SECRETASE COMPLEX	SKCM	1
c5: Ontology gene sets	GOCC FLEMMING BODY	THYM	1
c5: Ontology gene sets	GOCC FHF COMPLEX	ESCA	1

c5: Ontology gene sets	GOCC FANCONI ANEMIA NUCLEAR COMPLEX	LIHC	1
c5: Ontology gene sets	GOCC EXTRINSIC COMPONENT OF PRESYNAPTIC MEMBRANE	KIRC	1
c5: Ontology gene sets	GOCC EXTRINSIC COMPONENT OF ENDOPLASMIC RETICULUM MEMBRANE	UVM	1
c5: Ontology gene sets	GOCC EUKARYOTIC TRANSLATION INITIATION FACTOR 2B COMPLEX	LIHC	1
c5: Ontology gene sets	GOCC EUKARYOTIC 48S PREINITIATION COMPLEX	ACC	1
c5: Ontology gene sets	GOCC ESCRT I COMPLEX	KIRC	1
c5: Ontology gene sets	GOCC EPSILON DNA POLYMERASE COMPLEX	LIHC	1
c5: Ontology gene sets	GOCC ENZYME ACTIVATOR COMPLEX	KIRP	1
c5: Ontology gene sets	GOCC ELASTIC FIBER	LGG	1
c5: Ontology gene sets	GOCC DOPAMINERGIC SYNAPSE	THYM	1
c5: Ontology gene sets	GOCC DNA POLYMERASE COMPLEX	TGCT	1
c5: Ontology gene sets	GOCC DNA HELICASE COMPLEX	ACC	1
c5: Ontology gene sets	GOCC DIHYDROLIPOYL DEHYDROGENASE COMPLEX	KIRC	1
c5: Ontology gene sets	GOCC DEUTEROSOME	ACC	1
c5: Ontology gene sets	GOCC DERLIN 1 RETROTRANSLOCATION COMPLEX	KIRC	1
c5: Ontology gene sets	GOCC DENTATE GYRUS MOSSY FIBER	BLCA	1
c5: Ontology gene sets	GOCC DENDRITIC GROWTH CONE	UVM	1
c5: Ontology gene sets	GOCC DEATH INDUCING SIGNALING COMPLEX	LGG	1

c5: Ontology gene sets	GOCC CYTOSOLIC PROTEASOME COMPLEX	UVM	1
c5: Ontology gene sets	GOCC CYTOPLASMIC SIDE OF ROUGH ENDOPLASMIC RETICULUM MEMBRANE	ACC	1
c5: Ontology gene sets	GOCC CUL4A RING E3 UBIQUITIN LIGASE COMPLEX	KIRC	1
c5: Ontology gene sets	GOCC COPI VESICLE COAT	KIRC	1
c5: Ontology gene sets	GOCC COPI COATED VESICLE MEMBRANE	KIRC	1
c5: Ontology gene sets	GOCC CONDENSED NUCLEAR CHROMOSOME KINETOCHORE	UCEC	1
c5: Ontology gene sets	GOCC CLATHRIN SCULPTED MONOAMINE TRANSPORT VESICLE	LUAD	1
c5: Ontology gene sets	GOCC CILIARY ROOTLET	THYM	1
c5: Ontology gene sets	GOCC CHROMOCENTER	THYM	1
c5: Ontology gene sets	GOCC CHROMAFFIN GRANULE MEMBRANE	THYM	1
c5: Ontology gene sets	GOCC CHROMAFFIN GRANULE	KIRP	1
c5: Ontology gene sets	GOCC CHOLINERGIC SYNAPSE	LGG	1
c5: Ontology gene sets	GOCC CENTRIOLAR SUBDISTAL APPENDAGE	MESO	1
c5: Ontology gene sets	GOCC CCR4 NOT CORE COMPLEX	THYM	1
c5: Ontology gene sets	GOCC CATALYTIC STEP 1 SPLICEOSOME	KICH	1
c5: Ontology gene sets	GOCC CAMP DEPENDENT PROTEIN KINASE COMPLEX	KIRC	1
c5: Ontology gene sets	GOCC CALCIUM AND CALMODULIN DEPENDENT PROTEIN KINASE COMPLEX	DLBC	1
c5: Ontology gene sets	GOCC CALCINEURIN COMPLEX	PRAD	1
c5: Ontology gene sets	GOCC BRCA1 A COMPLEX	KICH	1

c5: Ontology gene sets	GOCC BOX H ACA TELOMERASE RNP COMPLEX	READ	1
c5: Ontology gene sets	GOCC BBSOME	BRCA	1
c5: Ontology gene sets	GOCC BASAL CORTEX	KIRC	1
c5: Ontology gene sets	GOCC BARR BODY	THYM	1
c5: Ontology gene sets	GOCC ASTER	THYM	1
c5: Ontology gene sets	GOCC ARYL HYDROCARBON RECEPTOR COMPLEX	LGG	1
c5: Ontology gene sets	GOCC AP 4 ADAPTOR COMPLEX	LGG	1
c5: Ontology gene sets	GOCC AP 3 ADAPTOR COMPLEX	UVM	1
c5: Ontology gene sets	GOCC ANNULATE LAMELLAE	ACC	1
c5: Ontology gene sets	GOCC ANCHORED COMPONENT OF EXTERNAL SIDE OF PLASMA MEMBRANE	COAD	1
c5: Ontology gene sets	GOCC AMINOACYL TRNA SYNTHETASE MULTIENZYME COMPLEX	READ	1
c5: Ontology gene sets	GOCC AGGRESOME	THYM	1
c5: Ontology gene sets	GOCC ADA2 GCN5 ADA3 TRANSCRIPTION ACTIVATOR COMPLEX	THCA	1
c5: Ontology gene sets	GOBP ZINC ION TRANSPORT	COAD	1
c5: Ontology gene sets	GOBP ZINC ION IMPORT ACROSS PLASMA MEMBRANE	COAD	1
c5: Ontology gene sets	GOBP VIRAL TRANSLATIONAL TERMINATION REINITIATION	ACC	1
c5: Ontology gene sets	GOBP VIRAL RELEASE FROM HOST CELL	READ	1
c5: Ontology gene sets	GOBP VIRAL LATENCY	KIRC	1
c5: Ontology gene sets	GOBP VESTIBULOCOCHLEAR NERVE FORMATION	BLCA	1
c5: Ontology gene sets	GOBP VESTIBULOCOCHLEAR NERVE DEVELOPMENT	BLCA	1

c5: Ontology gene sets	GOBP VESICLE TETHERING TO GOLGI	SKCM	1
c5: Ontology gene sets	GOBP VESICLE TETHERING	KIRC	1
c5: Ontology gene sets	GOBP VESICLE DOCKING INVOLVED IN EXOCYTOSIS	DLBC	1
c5: Ontology gene sets	GOBP VERY LONG CHAIN FATTY ACID CATABOLIC PROCESS	PCPG	1
c5: Ontology gene sets	GOBP VAGINA DEVELOPMENT	KIRC	1
c5: Ontology gene sets	GOBP VACUOLAR PROTON TRANSPORTING V TYPE ATPASE COMPLEX ASSEMBLY	UCEC	1
c5: Ontology gene sets	GOBP URATE TRANSPORT	KIRC	1
c5: Ontology gene sets	GOBP UBIQUITIN DEPENDENT GLYCOPROTEIN ERAD PATHWAY	COAD	1
c5: Ontology gene sets	GOBP TYPE I PNEUMOCYTE DIFFERENTIATION	KIRC	1
c5: Ontology gene sets	GOBP TYPE B PANCREATIC CELL PROLIFERATION	LUSC	1
c5: Ontology gene sets	GOBP TUBULIN COMPLEX ASSEMBLY	UCEC	1
c5: Ontology gene sets	GOBP TRYPTOPHAN TRANSPORT	MESO	1
c5: Ontology gene sets	GOBP TRYPTOPHAN CATABOLIC PROCESS TO KYNURENINE	BRCA	1
c5: Ontology gene sets	GOBP TROPHOBLAST GIANT CELL DIFFERENTIATION	KIRC	1
c5: Ontology gene sets	GOBP TRNA PSEUDOURIDINE SYNTHESIS	LGG	1
c5: Ontology gene sets	GOBP TRICUSPID VALVE DEVELOPMENT	KIRC	1
c5: Ontology gene sets	GOBP TRICARBOXYLIC ACID METABOLIC PROCESS	PRAD	1

c5: Ontology gene sets	GOBP TRANSLOCATION OF MOLECULES INTO HOST	KICH	1
c5: Ontology gene sets	GOBP TRANSCRIPTION DEPENDENT TETHERING OF RNA POLYMERASE II GENE DNA AT NUCLEAR PERIPHERY	ACC	1
c5: Ontology gene sets	GOBP TRABECULA FORMATION	READ	1
c5: Ontology gene sets	GOBP TOOTH ERUPTION	LGG	1
c5: Ontology gene sets	GOBP TOLL LIKE RECEPTOR 3 SIGNALING PATHWAY	READ	1
c5: Ontology gene sets	GOBP TOLL LIKE RECEPTOR 2 SIGNALING PATHWAY	PRAD	1
c5: Ontology gene sets	GOBP THYMIC T CELL SELECTION	UCEC	1
c5: Ontology gene sets	GOBP THROMBOPOIETIN MEDIATED SIGNALING PATHWAY	KIRC	1
c5: Ontology gene sets	GOBP THROMBIN ACTIVATED RECEPTOR SIGNALING PATHWAY	KIRC	1
c5: Ontology gene sets	GOBP THREONINE METABOLIC PROCESS	COAD	1
c5: Ontology gene sets	GOBP THORAX AND ANTERIOR ABDOMEN DETERMINATION	PCPG	1
c5: Ontology gene sets	GOBP TELOMERE MAINTENANCE VIA RECOMBINATION	LIHC	1
c5: Ontology gene sets	GOBP TELOMERASE RNA LOCALIZATION	LGG	1
c5: Ontology gene sets	GOBP TELENCEPHALON REGIONALIZATION	KIRC	1
c5: Ontology gene sets	GOBP TAURINE TRANSPORT	READ	1
c5: Ontology gene sets	GOBP TAIL ANCHORED MEMBRANE PROTEIN INSERTION INTO ER MEMBRANE	ESCA	1
c5: Ontology gene sets	GOBP T CELL EXTRAVASATION	LGG	1

c5: Ontology gene sets	GOBP SYNAPTIC VESICLE ENDOSOMAL PROCESSING	READ	1
c5: Ontology gene sets	GOBP SYNAPTIC VESICLE CLUSTERING	UCS	1
c5: Ontology gene sets	GOBP SUCCINYL COA METABOLIC PROCESS	KIRC	1
c5: Ontology gene sets	GOBP STRIATED MUSCLE ATROPHY	SKCM	1
c5: Ontology gene sets	GOBP STRESS GRANULE DISASSEMBLY	ACC	1
c5: Ontology gene sets	GOBP SRP DEPENDENT COTRANSLATIONAL PROTEIN TARGETING TO MEMBRANE TRANSLOCATION	UCEC	1
c5: Ontology gene sets	GOBP SPONTANEOUS NEUROTRANSMITTER SECRETION	BRCA	1
c5: Ontology gene sets	GOBP SPINDLE MIDZONE ASSEMBLY	LGG	1
c5: Ontology gene sets	GOBP SPINDLE ASSEMBLY INVOLVED IN MEIOSIS	ACC	1
c5: Ontology gene sets	GOBP SPHINGOMYELIN BIOSYNTHETIC PROCESS	KIRC	1
c5: Ontology gene sets	GOBP SPERM CHROMATIN CONDENSATION	PRAD	1
c5: Ontology gene sets	GOBP SODIUM DEPENDENT PHOSPHATE TRANSPORT	KIRC	1
c5: Ontology gene sets	GOBP SNRNA MODIFICATION	READ	1
c5: Ontology gene sets	GOBP SNORNA LOCALIZATION	ACC	1
c5: Ontology gene sets	GOBP SMOOTH MUSCLE TISSUE DEVELOPMENT	KIRC	1
c5: Ontology gene sets	GOBP SKELETAL MUSCLE SATELLITE CELL ACTIVATION	LUAD	1

c5: Ontology gene sets	GOBP SKELETAL MUSCLE CELL PROLIFERATION	READ	1
c5: Ontology gene sets	GOBP SIGNAL COMPLEX ASSEMBLY	KIRC	1
c5: Ontology gene sets	GOBP SERINE FAMILY AMINO ACID CATABOLIC PROCESS	THCA	1
c5: Ontology gene sets	GOBP SENSORY PERCEPTION OF TOUCH	BRCA	1
c5: Ontology gene sets	GOBP SELENIUM COMPOUND METABOLIC PROCESS	KIRP	1
c5: Ontology gene sets	GOBP SECRETION OF LYSOSOMAL ENZYMES	KIRC	1
c5: Ontology gene sets	GOBP SECONDARY HEART FIELD SPECIFICATION	CESC	1
c5: Ontology gene sets	GOBP SA NODE CELL TO ATRIAL CARDIAC MUSCLE CELL SIGNALING	PAAD	1
c5: Ontology gene sets	GOBP SA NODE CELL TO ATRIAL CARDIAC MUSCLE CELL COMMUNICATION	THCA	1
c5: Ontology gene sets	GOBP S SHAPED BODY MORPHOGENESIS	MESO	1
c5: Ontology gene sets	GOBP RRNA CONTAINING RIBONUCLEOPROTEIN COMPLEX EXPORT FROM NUCLEUS	THCA	1
c5: Ontology gene sets	GOBP RNA POLYMERASE II PREINITIATION COMPLEX ASSEMBLY	THYM	1
c5: Ontology gene sets	GOBP RNA GUANINE N7 METHYLATION	KIRC	1
c5: Ontology gene sets	GOBP RIGHTING REFLEX	LGG	1
c5: Ontology gene sets	GOBP RIBOSOMAL SMALL SUBUNIT EXPORT FROM NUCLEUS	KICH	1

c5: Ontology gene sets	GOBP RIBOSOMAL SMALL SUBUNIT ASSEMBLY	UVM	1
c5: Ontology gene sets	GOBP RIBONUCLEOPROTEIN COMPLEX DISASSEMBLY	KIRC	1
c5: Ontology gene sets	GOBP REVERSE CHOLESTEROL TRANSPORT	THYM	1
c5: Ontology gene sets	GOBP RETICULOPHAGY	READ	1
c5: Ontology gene sets	GOBP RESPONSE TO UV C	BRCA	1
c5: Ontology gene sets	GOBP RESPONSE TO REDOX STATE	KIRC	1
c5: Ontology gene sets	GOBP RESPONSE TO PHORBOL 13 ACETATE 12 MYRISTATE	KIRP	1
c5: Ontology gene sets	GOBP RESPONSE TO OLEIC ACID	UVM	1
c5: Ontology gene sets	GOBP RESPONSE TO MURAMYL DIPEPTIDE	THYM	1
c5: Ontology gene sets	GOBP RESPONSE TO MOLECULE OF FUNGAL ORIGIN	LGG	1
c5: Ontology gene sets	GOBP RESPONSE TO MAGNESIUM ION	READ	1
c5: Ontology gene sets	GOBP RESPONSE TO MACROPHAGE COLONY STIMULATING FACTOR	PCPG	1
c5: Ontology gene sets	GOBP RESPONSE TO INTERLEUKIN 9	BRCA	1
c5: Ontology gene sets	GOBP RESPONSE TO INTERLEUKIN 3	LUAD	1
c5: Ontology gene sets	GOBP RESPONSE TO INTERLEUKIN 15	COAD	1
c5: Ontology gene sets	GOBP RESPONSE TO INTERFERON ALPHA	SKCM	1
c5: Ontology gene sets	GOBP RESPONSE TO GLYCOSIDE	LGG	1
c5: Ontology gene sets	GOBP RESPONSE TO GLYCOPROTEIN	COAD	1

c5: Ontology gene sets	GOBP RESPONSE TO COBALT ION	KIRC	1
c5: Ontology gene sets	GOBP RESPONSE TO CISPLATIN	UVM	1
c5: Ontology gene sets	GOBP RESPONSE TO CELL CYCLE CHECKPOINT SIGNALING	LIHC	1
c5: Ontology gene sets	GOBP RESPONSE TO ALUMINUM ION	KIRC	1
c5: Ontology gene sets	GOBP RESPIRATORY BURST	DLBC	1
c5: Ontology gene sets	GOBP RENAL SYSTEM VASCULATURE MORPHOGENESIS	LGG	1
c5: Ontology gene sets	GOBP RELAXATION OF VASCULAR ASSOCIATED SMOOTH MUSCLE	BRCA	1
c5: Ontology gene sets	GOBP RELAXATION OF SMOOTH MUSCLE	BRCA	1
c5: Ontology gene sets	GOBP REGULATION OF VITAMIN METABOLIC PROCESS	LGG	1
c5: Ontology gene sets	GOBP REGULATION OF VITAMIN D RECEPTOR SIGNALING PATHWAY	THYM	1
c5: Ontology gene sets	GOBP REGULATION OF VESICLE SIZE	KIRC	1
c5: Ontology gene sets	GOBP REGULATION OF VENTRICULAR CARDIAC MUSCLE CELL MEMBRANE REPOLARIZATION	BRCA	1
c5: Ontology gene sets	GOBP REGULATION OF VENTRICULAR CARDIAC MUSCLE CELL ACTION POTENTIAL	KIRP	1
c5: Ontology gene sets	GOBP REGULATION OF TUBULIN DEACETYLATION	KIRC	1
c5: Ontology gene sets	GOBP REGULATION OF TRIGLYCERIDE CATABOLIC PROCESS	BRCA	1

c5: Ontology gene sets	GOBP REGULATION OF TRANSLATION AT SYNAPSE MODULATING SYNAPTIC TRANSMISSION	UVM	1
c5: Ontology gene sets	GOBP REGULATION OF TRANSCRIPTION FROM RNA POLYMERASE II PROMOTER INVOLVED IN HEART DEVELOPMENT	UVM	1
c5: Ontology gene sets	GOBP REGULATION OF TRAIL ACTIVATED APOPTOTIC SIGNALING PATHWAY	ACC	1
c5: Ontology gene sets	GOBP REGULATION OF TOLL LIKE RECEPTOR 3 SIGNALING PATHWAY	HNSC	1
c5: Ontology gene sets	GOBP REGULATION OF T HELPER 17 CELL LINEAGE COMMITMENT	BLCA	1
c5: Ontology gene sets	GOBP REGULATION OF T CELL RECEPTOR SIGNALING PATHWAY	PCPG	1
c5: Ontology gene sets	GOBP REGULATION OF T CELL DIFFERENTIATION IN THYMUS	DLBC	1
c5: Ontology gene sets	GOBP REGULATION OF SYSTEMIC ARTERIAL BLOOD PRESSURE BY VASOPRESSIN	LUAD	1
c5: Ontology gene sets	GOBP REGULATION OF SYNAPTIC TRANSMISSION CHOLINERGIC	ACC	1
c5: Ontology gene sets	GOBP REGULATION OF SUPEROXIDE ANION GENERATION	CESC	1
c5: Ontology gene sets	GOBP REGULATION OF STEROID HORMONE BIOSYNTHETIC PROCESS	KIRC	1
c5: Ontology gene sets	GOBP REGULATION OF SPINDLE ASSEMBLY	THYM	1

c5: Ontology gene sets	GOBP REGULATION OF SODIUM DEPENDENT PHOSPHATE TRANSPORT	KIRC	1
c5: Ontology gene sets	GOBP REGULATION OF SKELETAL MUSCLE TISSUE REGENERATION	LUAD	1
c5: Ontology gene sets	GOBP REGULATION OF SKELETAL MUSCLE CELL PROLIFERATION	READ	1
c5: Ontology gene sets	GOBP REGULATION OF SKELETAL MUSCLE ACETYLCHOLINE GATED CHANNEL CLUSTERING	UCEC	1
c5: Ontology gene sets	GOBP REGULATION OF SECONDARY HEART FIELD CARDIOBLAST PROLIFERATION	ACC	1
c5: Ontology gene sets	GOBP REGULATION OF RIBONUCLEOPROTEIN COMPLEX LOCALIZATION	KIRC	1
c5: Ontology gene sets	GOBP REGULATION OF RETROGRADE TRANSPORT ENDOSOME TO GOLGI	KIRC	1
c5: Ontology gene sets	GOBP REGULATION OF RESPONSE TO INTERFERON GAMMA	THYM	1
c5: Ontology gene sets	GOBP REGULATION OF RESPIRATORY SYSTEM PROCESS	LUAD	1
c5: Ontology gene sets	GOBP REGULATION OF RENAL SYSTEM PROCESS	PCPG	1
c5: Ontology gene sets	GOBP REGULATION OF REMOVAL OF SUPEROXIDE RADICALS	COAD	1
c5: Ontology gene sets	GOBP REGULATION OF RECEPTOR RECYCLING	THCA	1
c5: Ontology gene sets	GOBP REGULATION OF RECEPTOR BINDING	DLBC	1

c5: Ontology gene sets	GOBP REGULATION OF RAB PROTEIN SIGNAL TRANSDUCTION	READ	1
c5: Ontology gene sets	GOBP REGULATION OF PSEUDOPODIUM ASSEMBLY	PCPG	1
c5: Ontology gene sets	GOBP REGULATION OF PROTEIN MONOUBIQUITINATION	LIHC	1
c5: Ontology gene sets	GOBP REGULATION OF PROTEIN LOCALIZATION TO CHROMOSOME TELOMERIC REGION	KIRP	1
c5: Ontology gene sets	GOBP REGULATION OF PROTEIN LOCALIZATION TO CHROMATIN	KIRC	1
c5: Ontology gene sets	GOBP REGULATION OF PROTEIN LOCALIZATION TO CENTROSOME	KICH	1
c5: Ontology gene sets	GOBP REGULATION OF PROTEIN DEUBIQUITINATION	BRCA	1
c5: Ontology gene sets	GOBP REGULATION OF PROTEIN ADP RIBOSYLATION	KIRC	1
c5: Ontology gene sets	GOBP REGULATION OF PROSTATIC BUD FORMATION	ACC	1
c5: Ontology gene sets	GOBP REGULATION OF POTASSIUM ION EXPORT ACROSS PLASMA MEMBRANE	UCEC	1
c5: Ontology gene sets	GOBP REGULATION OF POLYAMINE TRANSMEMBRANE TRANSPORT	PAAD	1
c5: Ontology gene sets	GOBP REGULATION OF PODOSOME ASSEMBLY	LGG	1
c5: Ontology gene sets	GOBP REGULATION OF PLATELET DERIVED GROWTH FACTOR RECEPTOR BETA SIGNALING PATHWAY	UVM	1
c5: Ontology gene sets	GOBP REGULATION OF PHOSPHOLIPID TRANSPORT	DLBC	1

c5: Ontology gene sets	GOBP REGULATION OF PHOSPHOLIPID TRANSLOCATION	LGG	1
c5: Ontology gene sets	GOBP REGULATION OF PHOSPHOLIPASE C ACTIVATING G PROTEIN COUPLED RECEPTOR SIGNALING PATHWAY	KIRP	1
c5: Ontology gene sets	GOBP REGULATION OF PHOSPHATIDYLINOSITOL BIOSYNTHETIC PROCESS	LGG	1
c5: Ontology gene sets	GOBP REGULATION OF PHOSPHATE TRANSPORT	KIRP	1
c5: Ontology gene sets	GOBP REGULATION OF PEPTIDYL SERINE DEPHOSPHORYLATION	UCEC	1
c5: Ontology gene sets	GOBP REGULATION OF PANCREATIC JUICE SECRETION	COAD	1
c5: Ontology gene sets	GOBP REGULATION OF OVARIAN FOLLICLE DEVELOPMENT	KIRC	1
c5: Ontology gene sets	GOBP REGULATION OF NK T CELL PROLIFERATION	SARC	1
c5: Ontology gene sets	GOBP REGULATION OF NK T CELL DIFFERENTIATION	KIRC	1
c5: Ontology gene sets	GOBP REGULATION OF NADP METABOLIC PROCESS	BRCA	1
c5: Ontology gene sets	GOBP REGULATION OF MYELOID DENDRITIC CELL ACTIVATION	SKCM	1
c5: Ontology gene sets	GOBP REGULATION OF MYD88 INDEPENDENT TOLL LIKE RECEPTOR SIGNALING PATHWAY	KIRC	1
c5: Ontology gene sets	GOBP REGULATION OF MRNA 3 END PROCESSING	THYM	1
c5: Ontology gene sets	GOBP REGULATION OF MOTOR NEURON APOPTOTIC PROCESS	PCPG	1

c5: Ontology gene sets	GOBP REGULATION OF MODIFICATION OF SYNAPTIC STRUCTURE	LGG	1
c5: Ontology gene sets	GOBP REGULATION OF MITOTIC CYTOKINESIS	LUAD	1
c5: Ontology gene sets	GOBP REGULATION OF MITOCHONDRIAL ELECTRON TRANSPORT NADH TO UBIQUINONE	ACC	1
c5: Ontology gene sets	GOBP REGULATION OF MICROVILLUS ORGANIZATION	KIRC	1
c5: Ontology gene sets	GOBP REGULATION OF MICROVILLUS LENGTH	KIRC	1
c5: Ontology gene sets	GOBP REGULATION OF MICROTUBULE BINDING	THYM	1
c5: Ontology gene sets	GOBP REGULATION OF MESENCHYMAL STEM CELL DIFFERENTIATION	LGG	1
c5: Ontology gene sets	GOBP REGULATION OF MEMBRANE REPOLARIZATION DURING VENTRICULAR CARDIAC MUSCLE CELL ACTION POTENTIAL	BRCA	1
c5: Ontology gene sets	GOBP REGULATION OF MEMBRANE PROTEIN ECTODOMAIN PROTEOLYSIS	UVM	1
c5: Ontology gene sets	GOBP REGULATION OF MACROPHAGE MIGRATION	DLBC	1
c5: Ontology gene sets	GOBP REGULATION OF MACROPHAGE FUSION	KIRP	1
c5: Ontology gene sets	GOBP REGULATION OF LYSOSOME ORGANIZATION	COAD	1
c5: Ontology gene sets	GOBP REGULATION OF LONG CHAIN FATTY ACID IMPORT INTO CELL	PRAD	1

c5: Ontology gene sets	GOBP REGULATION OF LIPOPHAGY	PAAD	1
c5: Ontology gene sets	GOBP REGULATION OF KILLING OF CELLS OF OTHER ORGANISM	UVM	1
c5: Ontology gene sets	GOBP REGULATION OF ISOTYPE SWITCHING TO IGE ISOTYPES	SKCM	1
c5: Ontology gene sets	GOBP REGULATION OF INTESTINAL ABSORPTION	BRCA	1
c5: Ontology gene sets	GOBP REGULATION OF INORGANIC ANION TRANSMEMBRANE TRANSPORT	SKCM	1
c5: Ontology gene sets	GOBP REGULATION OF HYDROGEN PEROXIDE MEDIATED PROGRAMMED CELL DEATH	DLBC	1
c5: Ontology gene sets	GOBP REGULATION OF HISTONE PHOSPHORYLATION	KIRC	1
c5: Ontology gene sets	GOBP REGULATION OF HISTONE H3 K9 METHYLATION	MESO	1
c5: Ontology gene sets	GOBP REGULATION OF HISTONE H3 K36 METHYLATION	THYM	1
c5: Ontology gene sets	GOBP REGULATION OF HIPPO SIGNALING	THCA	1
c5: Ontology gene sets	GOBP REGULATION OF HEMOGLOBIN BIOSYNTHETIC PROCESS	KIRC	1
c5: Ontology gene sets	GOBP REGULATION OF HEART MORPHOGENESIS	PCPG	1
c5: Ontology gene sets	GOBP REGULATION OF GROWTH RATE	SKCM	1
c5: Ontology gene sets	GOBP REGULATION OF GLYCOGEN CATABOLIC PROCESS	ACC	1
c5: Ontology gene sets	GOBP REGULATION OF GLUCOCORTICOID METABOLIC PROCESS	KIRC	1

c5: Ontology gene sets	GOBP REGULATION OF GLIAL CELL MIGRATION	LUAD	1
c5: Ontology gene sets	GOBP REGULATION OF GAP JUNCTION ASSEMBLY	UVM	1
c5: Ontology gene sets	GOBP REGULATION OF GAMMA DELTA T CELL DIFFERENTIATION	BRCA	1
c5: Ontology gene sets	GOBP REGULATION OF FOREBRAIN NEURON DIFFERENTIATION	BLCA	1
c5: Ontology gene sets	GOBP REGULATION OF FOCAL ADHESION DISASSEMBLY	LUAD	1
c5: Ontology gene sets	GOBP REGULATION OF FC GAMMA RECEPTOR SIGNALING PATHWAY INVOLVED IN PHAGOCYTOSIS	BRCA	1
c5: Ontology gene sets	GOBP REGULATION OF EXIT FROM MITOSIS	LUAD	1
c5: Ontology gene sets	GOBP REGULATION OF ESTABLISHMENT OR MAINTENANCE OF CELL POLARITY	THYM	1
c5: Ontology gene sets	GOBP REGULATION OF ESTABLISHMENT OF PROTEIN LOCALIZATION TO CHROMOSOME	KIRP	1
c5: Ontology gene sets	GOBP REGULATION OF ER TO GOLGI VESICLE MEDIATED TRANSPORT	UVM	1
c5: Ontology gene sets	GOBP REGULATION OF ER ASSOCIATED UBIQUITIN DEPENDENT PROTEIN CATABOLIC PROCESS	KIRC	1
c5: Ontology gene sets	GOBP REGULATION OF EPIDERMAL GROWTH FACTOR ACTIVATED RECEPTOR ACTIVITY	THCA	1
c5: Ontology gene sets	GOBP REGULATION OF ENDOSOME SIZE	LUAD	1

c5: Ontology gene sets	GOBP REGULATION OF ENDODERMAL CELL DIFFERENTIATION	KIRC	1
c5: Ontology gene sets	GOBP REGULATION OF ENDOCYTIC RECYCLING	COAD	1
c5: Ontology gene sets	GOBP REGULATION OF DNA TOPOISOMERASE ATP HYDROLYZING ACTIVITY	BLCA	1
c5: Ontology gene sets	GOBP REGULATION OF DNA LIGATION	BRCA	1
c5: Ontology gene sets	GOBP REGULATION OF DNA HELICASE ACTIVITY	LIHC	1
c5: Ontology gene sets	GOBP REGULATION OF DNA DUPLEX UNWINDING	LIHC	1
c5: Ontology gene sets	GOBP REGULATION OF DNA DEPENDENT DNA REPLICATION INITIATION	SARC	1
c5: Ontology gene sets	GOBP REGULATION OF CYTOPLASMIC TRANSLATIONAL INITIATION	KIRC	1
c5: Ontology gene sets	GOBP REGULATION OF CORE PROMOTER BINDING	THYM	1
c5: Ontology gene sets	GOBP REGULATION OF COHESIN LOADING	KIRC	1
c5: Ontology gene sets	GOBP REGULATION OF CILIUM BEAT FREQUENCY	BRCA	1
c5: Ontology gene sets	GOBP REGULATION OF CHONDROCYTE DEVELOPMENT	UCEC	1
c5: Ontology gene sets	GOBP REGULATION OF CHAPERONE MEDIATED PROTEIN FOLDING	BLCA	1
c5: Ontology gene sets	GOBP REGULATION OF CHAPERONE MEDIATED AUTOPHAGY	UCEC	1

c5: Ontology gene sets	GOBP REGULATION OF CD8 POSITIVE ALPHA BETA T CELL DIFFERENTIATION	LGG	1
c5: Ontology gene sets	GOBP REGULATION OF BARBED END ACTIN FILAMENT CAPPING	KICH	1
c5: Ontology gene sets	GOBP REGULATION OF ATRIAL CARDIAC MUSCLE CELL MEMBRANE DEPOLARIZATION	KIRP	1
c5: Ontology gene sets	GOBP REGULATION OF ATPASE COUPLED CALCIUM TRANSMEMBRANE TRANSPORTER ACTIVITY	KIRC	1
c5: Ontology gene sets	GOBP REGULATION OF ARP2 3 COMPLEX MEDIATED ACTIN NUCLEATION	COAD	1
c5: Ontology gene sets	GOBP REGULATION OF ANION CHANNEL ACTIVITY	SKCM	1
c5: Ontology gene sets	GOBP REGULATION BY VIRUS OF VIRAL PROTEIN LEVELS IN HOST CELL	KIRC	1
c5: Ontology gene sets	GOBP RECEPTOR RECYCLING	THYM	1
c5: Ontology gene sets	GOBP PYRIMIDINE NUCLEOTIDE SALVAGE	ACC	1
c5: Ontology gene sets	GOBP PYRIMIDINE NUCLEOSIDE DIPHOSPHATE METABOLIC PROCESS	MESO	1
c5: Ontology gene sets	GOBP PYRIDINE CONTAINING COMPOUND CATABOLIC PROCESS	ACC	1
c5: Ontology gene sets	GOBP PURKINJE MYOCYTE TO VENTRICULAR CARDIAC MUSCLE CELL SIGNALING	KIRC	1
c5: Ontology gene sets	GOBP PURINE RIBONUCLEOSIDE SALVAGE	KIRC	1

c5: Ontology gene sets	GOBP PURINE NUCLEOSIDE DIPHOSPHATE CATABOLIC PROCESS	KIRC	1
c5: Ontology gene sets	GOBP PURINE DEOXYRIBONUCLEOTIDE CATABOLIC PROCESS	ACC	1
c5: Ontology gene sets	GOBP PURINE DEOXYRIBONUCLEOSIDE TRIPHOSPHATE CATABOLIC PROCESS	ACC	1
c5: Ontology gene sets	GOBP PULMONARY VALVE MORPHOGENESIS	READ	1
c5: Ontology gene sets	GOBP PULMONARY VALVE DEVELOPMENT	READ	1
c5: Ontology gene sets	GOBP PTERIDINE CONTAINING COMPOUND BIOSYNTHETIC PROCESS	UVM	1
c5: Ontology gene sets	GOBP PSEUDOPODIUM ORGANIZATION	PCPG	1
c5: Ontology gene sets	GOBP PROTON TRANSPORTING V TYPE ATPASE COMPLEX ASSEMBLY	UCEC	1
c5: Ontology gene sets	GOBP PROTON TRANSPORTING ATP SYNTHASE COMPLEX ASSEMBLY	BRCA	1
c5: Ontology gene sets	GOBP PROTEIN QUALITY CONTROL FOR MISFOLDED OR INCOMPLETELY SYNTHESIZED PROTEINS	THYM	1
c5: Ontology gene sets	GOBP PROTEIN POLYGLUTAMYLATION	PAAD	1
c5: Ontology gene sets	GOBP PROTEIN POLY ADP RIBOSYLATION	UVM	1
c5: Ontology gene sets	GOBP PROTEIN O LINKED MANNOSYLATION	KIRC	1

c5: Ontology gene sets	GOBP PROTEIN O LINKED GLYCOSYLATION VIA THREONINE	PRAD	1
c5: Ontology gene sets	GOBP PROTEIN MANNOSYLATION	THYM	1
c5: Ontology gene sets	GOBP PROTEIN LOCALIZATION TO NUCLEOLUS	KIRP	1
c5: Ontology gene sets	GOBP PROTEIN LOCALIZATION TO MICROTUBULE	ACC	1
c5: Ontology gene sets	GOBP PROTEIN LOCALIZATION TO MEMBRANE RAFT	COAD	1
c5: Ontology gene sets	GOBP PROTEIN LOCALIZATION TO KINETOCHORE	LIHC	1
c5: Ontology gene sets	GOBP PROTEIN LOCALIZATION TO ENDOPLASMIC RETICULUM EXIT SITE	SKCM	1
c5: Ontology gene sets	GOBP PROTEIN LOCALIZATION TO CHROMOSOME CENTROMERIC REGION	LIHC	1
c5: Ontology gene sets	GOBP PROTEIN LOCALIZATION TO CELL CORTEX	LUAD	1
c5: Ontology gene sets	GOBP PROTEIN LOCALIZATION TO ADHERENS JUNCTION	KIRC	1
c5: Ontology gene sets	GOBP PROTEIN K27 LINKED UBIQUITINATION	READ	1
c5: Ontology gene sets	GOBP PROTEIN INSERTION INTO MITOCHONDRIAL MEMBRANE INVOLVED IN APOPTOTIC SIGNALING PATHWAY	BRCA	1
c5: Ontology gene sets	GOBP PROTEIN IMPORT INTO MITOCHONDRIAL MATRIX	ACC	1
c5: Ontology gene sets	GOBP PROTEIN HYDROXYLATION	KICH	1
c5: Ontology gene sets	GOBP PROTEIN DESUMOYLATION	THYM	1

c5: Ontology gene sets	GOBP PROTEIN DEPALMITOYLATION	LUSC	1
c5: Ontology gene sets	GOBP PROTEIN DEMETHYLATION	PRAD	1
c5: Ontology gene sets	GOBP PROTEASOME ASSEMBLY	LIHC	1
c5: Ontology gene sets	GOBP PROSTHETIC GROUP METABOLIC PROCESS	PAAD	1
c5: Ontology gene sets	GOBP PRIMARY MIRNA PROCESSING	COAD	1
c5: Ontology gene sets	GOBP POSTTRANSLATIONAL PROTEIN TARGETING TO ENDOPLASMIC RETICULUM MEMBRANE	KIRC	1
c5: Ontology gene sets	GOBP POSTSYNAPTIC DENSITY PROTEIN 95 CLUSTERING	LGG	1
c5: Ontology gene sets	GOBP POST CHAPERONIN TUBULIN FOLDING PATHWAY	LUAD	1
c5: Ontology gene sets	GOBP POSITIVE THYMIC T CELL SELECTION	UCEC	1
c5: Ontology gene sets	GOBP POSITIVE REGULATION OF WOUND HEALING SPREADING OF EPIDERMAL CELLS	UCEC	1
c5: Ontology gene sets	GOBP POSITIVE REGULATION OF WOUND HEALING	DLBC	1
c5: Ontology gene sets	GOBP POSITIVE REGULATION OF VOLTAGE GATED CALCIUM CHANNEL ACTIVITY	BRCA	1
c5: Ontology gene sets	GOBP POSITIVE REGULATION OF VASCULAR ASSOCIATED SMOOTH MUSCLE CELL APOPTOTIC PROCESS	BRCA	1
c5: Ontology gene sets	GOBP POSITIVE REGULATION OF UTERINE SMOOTH MUSCLE CONTRACTION	KIRC	1

c5: Ontology gene sets	GOBP POSITIVE REGULATION OF TUMOR NECROSIS FACTOR MEDIATED SIGNALING PATHWAY	KIRC	1
c5: Ontology gene sets	GOBP POSITIVE REGULATION OF TRIGLYCERIDE LIPASE ACTIVITY	BRCA	1
c5: Ontology gene sets	GOBP POSITIVE REGULATION OF TRANSFORMING GROWTH FACTOR BETA1 PRODUCTION	GBM	1
c5: Ontology gene sets	GOBP POSITIVE REGULATION OF TOLL LIKE RECEPTOR 4 SIGNALING PATHWAY	KIRC	1
c5: Ontology gene sets	GOBP POSITIVE REGULATION OF TELOMERE CAPPING	THYM	1
c5: Ontology gene sets	GOBP POSITIVE REGULATION OF T HELPER 2 CELL DIFFERENTIATION	SKCM	1
c5: Ontology gene sets	GOBP POSITIVE REGULATION OF T HELPER 2 CELL CYTOKINE PRODUCTION	SKCM	1
c5: Ontology gene sets	GOBP POSITIVE REGULATION OF T HELPER 17 CELL DIFFERENTIATION	BLCA	1
c5: Ontology gene sets	GOBP POSITIVE REGULATION OF T CELL RECEPTOR SIGNALING PATHWAY	BRCA	1
c5: Ontology gene sets	GOBP POSITIVE REGULATION OF T CELL MEDIATED CYTOTOXICITY	THYM	1
c5: Ontology gene sets	GOBP POSITIVE REGULATION OF T CELL DIFFERENTIATION IN THYMUS	THYM	1
c5: Ontology gene sets	GOBP POSITIVE REGULATION OF SYNAPSE MATURATION	KIRP	1

c5: Ontology gene sets	GOBP POSITIVE REGULATION OF STRESS FIBER ASSEMBLY	DLBC	1
c5: Ontology gene sets	GOBP POSITIVE REGULATION OF RIG I SIGNALING PATHWAY	PRAD	1
c5: Ontology gene sets	GOBP POSITIVE REGULATION OF RESPONSE TO OXIDATIVE STRESS	KIRP	1
c5: Ontology gene sets	GOBP POSITIVE REGULATION OF RESPONSE TO ENDOPLASMIC RETICULUM STRESS	THYM	1
c5: Ontology gene sets	GOBP POSITIVE REGULATION OF REGULATORY T CELL DIFFERENTIATION	KIRC	1
c5: Ontology gene sets	GOBP POSITIVE REGULATION OF RECEPTOR BINDING	KIRC	1
c5: Ontology gene sets	GOBP POSITIVE REGULATION OF RAC PROTEIN SIGNAL TRANSDUCTION	KIRC	1
c5: Ontology gene sets	GOBP POSITIVE REGULATION OF PROTEIN MONOUBIQUITINATION	LIHC	1
c5: Ontology gene sets	GOBP POSITIVE REGULATION OF PROTEIN LOCALIZATION TO CENTROSOME	KICH	1
c5: Ontology gene sets	GOBP POSITIVE REGULATION OF PROTEIN KINASE A SIGNALING	KIRC	1
c5: Ontology gene sets	GOBP POSITIVE REGULATION OF PHOSPHOLIPID TRANSLOCATION	LGG	1
c5: Ontology gene sets	GOBP POSITIVE REGULATION OF PHAGOCYTOSIS ENGULFMENT	BRCA	1
c5: Ontology gene sets	GOBP POSITIVE REGULATION OF PENILE ERECTION	READ	1

c5: Ontology gene sets	GOBP POSITIVE REGULATION OF OXIDATIVE STRESS INDUCED NEURON DEATH	SKCM	1
c5: Ontology gene sets	GOBP POSITIVE REGULATION OF ODONTOGENESIS	KIRC	1
c5: Ontology gene sets	GOBP POSITIVE REGULATION OF NUCLEOTIDE BINDING OLIGOMERIZATION DOMAIN CONTAINING SIGNALING PATHWAY	THCA	1
c5: Ontology gene sets	GOBP POSITIVE REGULATION OF NUCLEAR CELL CYCLE DNA REPLICATION	KIRC	1
c5: Ontology gene sets	GOBP POSITIVE REGULATION OF NEUROTRANSMITTER UPTAKE	SARC	1
c5: Ontology gene sets	GOBP POSITIVE REGULATION OF NEURON MIGRATION	KIRC	1
c5: Ontology gene sets	GOBP POSITIVE REGULATION OF MYELOID CELL APOPTOTIC PROCESS	COAD	1
c5: Ontology gene sets	GOBP POSITIVE REGULATION OF METAPHASE ANAPHASE TRANSITION OF CELL CYCLE	LIHC	1
c5: Ontology gene sets	GOBP POSITIVE REGULATION OF METALLOENDOPEPTIDASE ACTIVITY	UVM	1
c5: Ontology gene sets	GOBP POSITIVE REGULATION OF MESENCHYMAL STEM CELL PROLIFERATION	LGG	1
c5: Ontology gene sets	GOBP POSITIVE REGULATION OF MEMORY T CELL DIFFERENTIATION	CESC	1

c5: Ontology gene sets	GOBP POSITIVE REGULATION OF MEMBRANE TUBULATION	KIRC	1
c5: Ontology gene sets	GOBP POSITIVE REGULATION OF MAST CELL ACTIVATION	READ	1
c5: Ontology gene sets	GOBP POSITIVE REGULATION OF MACROPHAGE CYTOKINE PRODUCTION	KIRC	1
c5: Ontology gene sets	GOBP POSITIVE REGULATION OF LYMPHOCYTE APOPTOTIC PROCESS	THYM	1
c5: Ontology gene sets	GOBP POSITIVE REGULATION OF LONG-TERM SYNAPTIC DEPRESSION	LGG	1
c5: Ontology gene sets	GOBP POSITIVE REGULATION OF LONG TERM NEURONAL SYNAPTIC PLASTICITY	HNSC	1
c5: Ontology gene sets	GOBP POSITIVE REGULATION OF LIPOPROTEIN METABOLIC PROCESS	KIRC	1
c5: Ontology gene sets	GOBP POSITIVE REGULATION OF LEUKOCYTE TETHERING OR ROLLING	SARC	1
c5: Ontology gene sets	GOBP POSITIVE REGULATION OF LEUKOCYTE DEGRANULATION	READ	1
c5: Ontology gene sets	GOBP POSITIVE REGULATION OF LAMELLIPODIUM ASSEMBLY	DLBC	1
c5: Ontology gene sets	GOBP POSITIVE REGULATION OF INTERLEUKIN 4 PRODUCTION	UVM	1
c5: Ontology gene sets	GOBP POSITIVE REGULATION OF INTERLEUKIN 1 ALPHA PRODUCTION	LUAD	1

c5: Ontology gene sets	GOBP POSITIVE REGULATION OF IMMATURE T CELL PROLIFERATION	CESC	1
c5: Ontology gene sets	GOBP POSITIVE REGULATION OF HISTONE UBIQUITINATION	KIRC	1
c5: Ontology gene sets	GOBP POSITIVE REGULATION OF HISTONE PHOSPHORYLATION	KIRC	1
c5: Ontology gene sets	GOBP POSITIVE REGULATION OF HISTONE METHYLATION	THYM	1
c5: Ontology gene sets	GOBP POSITIVE REGULATION OF HISTONE H4 ACETYLATION	GBM	1
c5: Ontology gene sets	GOBP POSITIVE REGULATION OF HISTONE H3 K27 METHYLATION	COAD	1
c5: Ontology gene sets	GOBP POSITIVE REGULATION OF HIGH VOLTAGE GATED CALCIUM CHANNEL ACTIVITY	BRCA	1
c5: Ontology gene sets	GOBP POSITIVE REGULATION OF GRANULOCYTE MACROPHAGE COLONY STIMULATING FACTOR PRODUCTION	CESC	1
c5: Ontology gene sets	GOBP POSITIVE REGULATION OF GRANULOCYTE DIFFERENTIATION	BRCA	1
c5: Ontology gene sets	GOBP POSITIVE REGULATION OF GONADOTROPIN SECRETION	THCA	1
c5: Ontology gene sets	GOBP POSITIVE REGULATION OF GLIAL CELL MIGRATION	LUAD	1
c5: Ontology gene sets	GOBP POSITIVE REGULATION OF GAMMA DELTA T CELL ACTIVATION	UVM	1
c5: Ontology gene sets	GOBP POSITIVE REGULATION OF FIBROBLAST MIGRATION	KIRP	1

c5: Ontology gene sets	GOBP POSITIVE REGULATION OF EXTRINSIC APOPTOTIC SIGNALING PATHWAY IN ABSENCE OF LIGAND	LGG	1
c5: Ontology gene sets	GOBP POSITIVE REGULATION OF ERBB SIGNALING PATHWAY	DLBC	1
c5: Ontology gene sets	GOBP POSITIVE REGULATION OF ER ASSOCIATED UBIQUITIN DEPENDENT PROTEIN CATABOLIC PROCESS	KIRC	1
c5: Ontology gene sets	GOBP POSITIVE REGULATION OF EPITHELIAL CELL DIFFERENTIATION INVOLVED IN KIDNEY DEVELOPMENT	KIRP	1
c5: Ontology gene sets	GOBP POSITIVE REGULATION OF ENDOCYTIC RECYCLING	COAD	1
c5: Ontology gene sets	GOBP POSITIVE REGULATION OF DNA TEMPLATED TRANSCRIPTION ELONGATION	LIHC	1
c5: Ontology gene sets	GOBP POSITIVE REGULATION OF DNA METHYLATION DEPENDENT HETEROCHROMATIN ASSEMBLY	THYM	1
c5: Ontology gene sets	GOBP POSITIVE REGULATION OF DNA LIGATION	BRCA	1
c5: Ontology gene sets	GOBP POSITIVE REGULATION OF DNA CATABOLIC PROCESS	UVM	1
c5: Ontology gene sets	GOBP POSITIVE REGULATION OF DENDRITIC CELL CHEMOTAXIS	CHOL	1
c5: Ontology gene sets	GOBP POSITIVE REGULATION OF DENDRITIC CELL ANTIGEN PROCESSING AND PRESENTATION	UCEC	1

c5: Ontology gene sets	GOBP POSITIVE REGULATION OF DENDRITE DEVELOPMENT	KIRC	1
c5: Ontology gene sets	GOBP POSITIVE REGULATION OF CYTOPLASMIC MRNA PROCESSING BODY ASSEMBLY	THYM	1
c5: Ontology gene sets	GOBP POSITIVE REGULATION OF CYCLIC NUCLEOTIDE PHOSPHODIESTERASE ACTIVITY	HNSC	1
c5: Ontology gene sets	GOBP POSITIVE REGULATION OF CHOLESTEROL METABOLIC PROCESS	UCEC	1
c5: Ontology gene sets	GOBP POSITIVE REGULATION OF CHOLESTEROL BIOSYNTHETIC PROCESS	UCEC	1
c5: Ontology gene sets	GOBP POSITIVE REGULATION OF CHEMOKINE C X C MOTIF LIGAND 2 PRODUCTION	UVM	1
c5: Ontology gene sets	GOBP POSITIVE REGULATION OF CGMP MEDIATED SIGNALING	KIRC	1
c5: Ontology gene sets	GOBP POSITIVE REGULATION OF CEREBELLAR GRANULE CELL PRECURSOR PROLIFERATION	LGG	1
c5: Ontology gene sets	GOBP POSITIVE REGULATION OF CENTRIOLE REPLICATION	KIRC	1
c5: Ontology gene sets	GOBP POSITIVE REGULATION OF CELL CELL ADHESION MEDIATED BY INTEGRIN	KIRC	1
c5: Ontology gene sets	GOBP POSITIVE REGULATION OF CD8 POSITIVE ALPHA BETA T CELL DIFFERENTIATION	LGG	1

c5: Ontology gene sets	GOBP POSITIVE REGULATION OF CD8 POSITIVE ALPHA BETA T CELL ACTIVATION	LGG	1
c5: Ontology gene sets	GOBP POSITIVE REGULATION OF CAMP DEPENDENT PROTEIN KINASE ACTIVITY	KIRP	1
c5: Ontology gene sets	GOBP POSITIVE REGULATION OF BLOOD PRESSURE	DLBC	1
c5: Ontology gene sets	GOBP POSITIVE REGULATION OF AUTOPHAGOSOME MATURATION	KIRC	1
c5: Ontology gene sets	GOBP POSITIVE REGULATION OF ATTACHMENT OF SPINDLE MICROTUBULES TO KINETOCHORE	LIHC	1
c5: Ontology gene sets	GOBP POSITIVE REGULATION OF APOPTOTIC PROCESS INVOLVED IN DEVELOPMENT	SKCM	1
c5: Ontology gene sets	GOBP POSITIVE REGULATION OF APOPTOTIC DNA FRAGMENTATION	UVM	1
c5: Ontology gene sets	GOBP POSITIVE REGULATION OF AMYLOID BETA CLEARANCE	KIRC	1
c5: Ontology gene sets	GOBP POSITIVE REGULATION OF ACTIN NUCLEATION	UVM	1
c5: Ontology gene sets	GOBP POSITIVE REGULATION OF ACTIN FILAMENT DEPOLYMERIZATION	KIRC	1
c5: Ontology gene sets	GOBP PORE COMPLEX ASSEMBLY	UVM	1
c5: Ontology gene sets	GOBP POLARIZED EPITHELIAL CELL DIFFERENTIATION	BRCA	1
c5: Ontology gene sets	GOBP PODOSOME ASSEMBLY	LGG	1

c5: Ontology gene sets	GOBP PLUS END DIRECTED ORGANELLE TRANSPORT ALONG MICROTUBULE	KIRC	1
c5: Ontology gene sets	GOBP PLASMA MEMBRANE TO ENDOSOME TRANSPORT	UVM	1
c5: Ontology gene sets	GOBP PLASMA MEMBRANE PHOSPHOLIPID SCRAMBLING	UCS	1
c5: Ontology gene sets	GOBP PLANAR CELL POLARITY PATHWAY INVOLVED IN NEURAL TUBE CLOSURE	UVM	1
c5: Ontology gene sets	GOBP PIGMENT GRANULE MATURATION	KIRC	1
c5: Ontology gene sets	GOBP PIGMENT GRANULE LOCALIZATION	DLBC	1
c5: Ontology gene sets	GOBP PIGMENT ACCUMULATION	KIRC	1
c5: Ontology gene sets	GOBP PHOSPHATIDYLGLYCEROL BIOSYNTHETIC PROCESS	KIRC	1
c5: Ontology gene sets	GOBP PHOSPHATIDYLCHOLINE BIOSYNTHETIC PROCESS	TGCT	1
c5: Ontology gene sets	GOBP PHOSPHAGEN METABOLIC PROCESS	PRAD	1
c5: Ontology gene sets	GOBP PEROXISOMAL MEMBRANE TRANSPORT	KIRC	1
c5: Ontology gene sets	GOBP PERICARDIUM MORPHOGENESIS	KIRC	1
c5: Ontology gene sets	GOBP PENTOSE CATABOLIC PROCESS	LIHC	1
c5: Ontology gene sets	GOBP PARAXIAL MESODERM FORMATION	ESCA	1
c5: Ontology gene sets	GOBP PARANODAL JUNCTION ASSEMBLY	ACC	1

c5: Ontology gene sets	GOBP OXALOACETATE METABOLIC PROCESS	LGG	1
c5: Ontology gene sets	GOBP OUTER EAR MORPHOGENESIS	ACC	1
c5: Ontology gene sets	GOBP OSTEOCLAST FUSION	GBM	1
c5: Ontology gene sets	GOBP ONE CARBON METABOLIC PROCESS	READ	1
c5: Ontology gene sets	GOBP OLIGOPEPTIDE IMPORT ACROSS PLASMA MEMBRANE	HNSC	1
c5: Ontology gene sets	GOBP OLIGODENDROCYTE PROGENITOR PROLIFERATION	LUSC	1
c5: Ontology gene sets	GOBP NUCLEOTIDE SUGAR TRANSMEMBRANE TRANSPORT	KIRC	1
c5: Ontology gene sets	GOBP NUCLEOSOME POSITIONING	THYM	1
c5: Ontology gene sets	GOBP NUCLEOSIDE MONOPHOSPHATE CATABOLIC PROCESS	SKCM	1
c5: Ontology gene sets	GOBP NUCLEOSIDE BISPHOSPHATE CATABOLIC PROCESS	LUAD	1
c5: Ontology gene sets	GOBP NUCLEOLAR LARGE RRNA TRANSCRIPTION BY RNA POLYMERASE I	KIRC	1
c5: Ontology gene sets	GOBP NUCLEAR RETENTION OF PRE MRNA AT THE SITE OF TRANSCRIPTION	LIHC	1
c5: Ontology gene sets	GOBP NUCLEAR PORE ORGANIZATION	KIRC	1
c5: Ontology gene sets	GOBP NUCLEAR PORE COMPLEX ASSEMBLY	PRAD	1
c5: Ontology gene sets	GOBP NUCLEAR NCRNA SURVEILLANCE	LIHC	1
c5: Ontology gene sets	GOBP NOTCH RECEPTOR PROCESSING	THYM	1

c5: Ontology gene sets	GOBP NOREPINEPHRINE METABOLIC PROCESS	LUAD	1
c5: Ontology gene sets	GOBP NON-CANONICAL WNT SIGNALING PATHWAY VIA MAPK CASCADE	KIRP	1
c5: Ontology gene sets	GOBP NLRP3 INFLAMMASOME COMPLEX ASSEMBLY	SKCM	1
c5: Ontology gene sets	GOBP NK T CELL DIFFERENTIATION	UVM	1
c5: Ontology gene sets	GOBP NEUROTRANSMITTER GATED ION CHANNEL CLUSTERING	KIRC	1
c5: Ontology gene sets	GOBP NEURONAL ACTION POTENTIAL PROPAGATION	ACC	1
c5: Ontology gene sets	GOBP NEURON PROJECTION MAINTENANCE	BLCA	1
c5: Ontology gene sets	GOBP NEURON FATE DETERMINATION	COAD	1
c5: Ontology gene sets	GOBP NEUROBLAST DIVISION	UCEC	1
c5: Ontology gene sets	GOBP NEURAL CREST CELL MIGRATION INVOLVED IN AUTONOMIC NERVOUS SYSTEM DEVELOPMENT	BLCA	1
c5: Ontology gene sets	GOBP NETRIN ACTIVATED SIGNALING PATHWAY	MESO	1
c5: Ontology gene sets	GOBP NERVE GROWTH FACTOR SIGNALING PATHWAY	KIRC	1
c5: Ontology gene sets	GOBP NEGATIVE T CELL SELECTION	UCEC	1
c5: Ontology gene sets	GOBP NEGATIVE REGULATION OF VIRAL RELEASE FROM HOST CELL	KIRC	1

c5: Ontology gene sets	GOBP NEGATIVE REGULATION OF VIRAL INDUCED CYTOPLASMIC PATTERN RECOGNITION RECEPTOR SIGNALING PATHWAY	LUAD	1
c5: Ontology gene sets	GOBP NEGATIVE REGULATION OF UBIQUITIN PROTEIN LIGASE ACTIVITY	KIRP	1
c5: Ontology gene sets	GOBP NEGATIVE REGULATION OF TRANSLATIONAL INITIATION	BRCA	1
c5: Ontology gene sets	GOBP NEGATIVE REGULATION OF TRANSFORMING GROWTH FACTOR BETA1 PRODUCTION	KIRP	1
c5: Ontology gene sets	GOBP NEGATIVE REGULATION OF TRANSFORMING GROWTH FACTOR BETA PRODUCTION	THYM	1
c5: Ontology gene sets	GOBP NEGATIVE REGULATION OF TRANSCRIPTION REGULATORY REGION DNA BINDING	THYM	1
c5: Ontology gene sets	GOBP NEGATIVE REGULATION OF TRANSCRIPTION BY RNA POLYMERASE I	KIRP	1
c5: Ontology gene sets	GOBP NEGATIVE REGULATION OF TRANSCRIPTION BY COMPETITIVE PROMOTER BINDING	LGG	1
c5: Ontology gene sets	GOBP NEGATIVE REGULATION OF THYMOCYTE APOPTOTIC PROCESS	LUAD	1
c5: Ontology gene sets	GOBP NEGATIVE REGULATION OF T HELPER 17 TYPE IMMUNE RESPONSE	READ	1

c5: Ontology gene sets	GOBP NEGATIVE REGULATION OF T CELL RECEPTOR SIGNALING PATHWAY	READ	1
c5: Ontology gene sets	GOBP NEGATIVE REGULATION OF SYSTEMIC ARTERIAL BLOOD PRESSURE	PCPG	1
c5: Ontology gene sets	GOBP NEGATIVE REGULATION OF SYNAPSE ORGANIZATION	LGG	1
c5: Ontology gene sets	GOBP NEGATIVE REGULATION OF STEM CELL DIFFERENTIATION	MESO	1
c5: Ontology gene sets	GOBP NEGATIVE REGULATION OF SMAD PROTEIN COMPLEX ASSEMBLY	KIRC	1
c5: Ontology gene sets	GOBP NEGATIVE REGULATION OF SISTER CHROMATID COHESION	KIRC	1
c5: Ontology gene sets	GOBP NEGATIVE REGULATION OF SERINE TYPE PEPTIDASE ACTIVITY	STAD	1
c5: Ontology gene sets	GOBP NEGATIVE REGULATION OF SECONDARY METABOLITE BIOSYNTHETIC PROCESS	LGG	1
c5: Ontology gene sets	GOBP NEGATIVE REGULATION OF RESPONSE TO REACTIVE OXYGEN SPECIES	KIRC	1
c5: Ontology gene sets	GOBP NEGATIVE REGULATION OF RESPONSE TO INTERFERON GAMMA	BRCA	1
c5: Ontology gene sets	GOBP NEGATIVE REGULATION OF RESPIRATORY BURST INVOLVED IN INFLAMMATORY RESPONSE	BLCA	1

c5: Ontology gene sets	GOBP NEGATIVE REGULATION OF REGULATED SECRETORY PATHWAY	PCPG	1
c5: Ontology gene sets	GOBP NEGATIVE REGULATION OF PROTEIN TYROSINE KINASE ACTIVITY	THYM	1
c5: Ontology gene sets	GOBP NEGATIVE REGULATION OF PROTEIN IMPORT	MESO	1
c5: Ontology gene sets	GOBP NEGATIVE REGULATION OF PROTEIN FOLDING	KIRC	1
c5: Ontology gene sets	GOBP NEGATIVE REGULATION OF PRODUCTION OF MIRNAS INVOLVED IN GENE SILENCING BY MIRNA	KIRC	1
c5: Ontology gene sets	GOBP NEGATIVE REGULATION OF PLATELET ACTIVATION	DLBC	1
c5: Ontology gene sets	GOBP NEGATIVE REGULATION OF PINOCYTOSIS	COAD	1
c5: Ontology gene sets	GOBP NEGATIVE REGULATION OF PHAGOCYTOSIS	THYM	1
c5: Ontology gene sets	GOBP NEGATIVE REGULATION OF ORGANELLE ASSEMBLY	DLBC	1
c5: Ontology gene sets	GOBP NEGATIVE REGULATION OF NUCLEASE ACTIVITY	UVM	1
c5: Ontology gene sets	GOBP NEGATIVE REGULATION OF NLRP3 INFLAMMASOME COMPLEX ASSEMBLY	SKCM	1
c5: Ontology gene sets	GOBP NEGATIVE REGULATION OF NEURON MIGRATION	LGG	1
c5: Ontology gene sets	GOBP NEGATIVE REGULATION OF NEUROBLAST PROLIFERATION	LGG	1

c5: Ontology gene sets	GOBP NEGATIVE REGULATION OF NATURAL KILLER CELL MEDIATED IMMUNITY	BLCA	1
c5: Ontology gene sets	GOBP NEGATIVE REGULATION OF MYOTUBE DIFFERENTIATION	COAD	1
c5: Ontology gene sets	GOBP NEGATIVE REGULATION OF MYELOID LEUKOCYTE MEDIATED IMMUNITY	LGG	1
c5: Ontology gene sets	GOBP NEGATIVE REGULATION OF MYELINATION	THYM	1
c5: Ontology gene sets	GOBP NEGATIVE REGULATION OF MITOCHONDRION ORGANIZATION	DLBC	1
c5: Ontology gene sets	GOBP NEGATIVE REGULATION OF MITOCHONDRIAL MEMBRANE PERMEABILITY INVOLVED IN APOPTOTIC PROCESS	LGG	1
c5: Ontology gene sets	GOBP NEGATIVE REGULATION OF MICROTUBULE POLYMERIZATION OR DEPOLYMERIZATION	THYM	1
c5: Ontology gene sets	GOBP NEGATIVE REGULATION OF MEIOTIC NUCLEAR DIVISION	LUSC	1
c5: Ontology gene sets	GOBP NEGATIVE REGULATION OF MAST CELL DEGRANULATION	LGG	1
c5: Ontology gene sets	GOBP NEGATIVE REGULATION OF MACROPHAGE DIFFERENTIATION	KIRC	1
c5: Ontology gene sets	GOBP NEGATIVE REGULATION OF MACROPHAGE CYTOKINE PRODUCTION	LGG	1
c5: Ontology gene sets	GOBP NEGATIVE REGULATION OF MACROPHAGE CHEMOTAXIS	SKCM	1

c5: Ontology gene sets	GOBP NEGATIVE REGULATION OF MACROAUTOPHAGY	DLBC	1
c5: Ontology gene sets	GOBP NEGATIVE REGULATION OF LIPOPOLYSACCHARIDE MEDIATED SIGNALING PATHWAY	ESCA	1
c5: Ontology gene sets	GOBP NEGATIVE REGULATION OF LEUKOCYTE MIGRATION	READ	1
c5: Ontology gene sets	GOBP NEGATIVE REGULATION OF LEUKOCYTE DEGRANULATION	LGG	1
c5: Ontology gene sets	GOBP NEGATIVE REGULATION OF KERATINOCYTE DIFFERENTIATION	BRCA	1
c5: Ontology gene sets	GOBP NEGATIVE REGULATION OF JUN KINASE ACTIVITY	KIRC	1
c5: Ontology gene sets	GOBP NEGATIVE REGULATION OF INTERLEUKIN 12 PRODUCTION	KIRC	1
c5: Ontology gene sets	GOBP NEGATIVE REGULATION OF INTERFERON BETA PRODUCTION	UCEC	1
c5: Ontology gene sets	GOBP NEGATIVE REGULATION OF IMMUNOGLOBULIN PRODUCTION	HNSC	1
c5: Ontology gene sets	GOBP NEGATIVE REGULATION OF HUMORAL IMMUNE RESPONSE MEDIATED BY CIRCULATING IMMUNOGLOBULIN	LGG	1
c5: Ontology gene sets	GOBP NEGATIVE REGULATION OF HORMONE METABOLIC PROCESS	LGG	1
c5: Ontology gene sets	GOBP NEGATIVE REGULATION OF HISTONE H3 K9 METHYLATION	KIRC	1
c5: Ontology gene sets	GOBP NEGATIVE REGULATION OF HISTONE H3 K27 METHYLATION	COAD	1
c5: Ontology gene sets	GOBP NEGATIVE REGULATION OF HIPPO SIGNALING	KIRC	1

c5: Ontology gene sets	GOBP NEGATIVE REGULATION OF GUANYL NUCLEOTIDE EXCHANGE FACTOR ACTIVITY	UVM	1
c5: Ontology gene sets	GOBP NEGATIVE REGULATION OF GLUCOCORTICOID RECEPTOR SIGNALING PATHWAY	KIRC	1
c5: Ontology gene sets	GOBP NEGATIVE REGULATION OF GLIAL CELL PROLIFERATION	PRAD	1
c5: Ontology gene sets	GOBP NEGATIVE REGULATION OF FIBROBLAST MIGRATION	COAD	1
c5: Ontology gene sets	GOBP NEGATIVE REGULATION OF FIBRINOLYSIS	LIHC	1
c5: Ontology gene sets	GOBP NEGATIVE REGULATION OF FATTY ACID BETA OXIDATION	BRCA	1
c5: Ontology gene sets	GOBP NEGATIVE REGULATION OF EXTRACELLULAR MATRIX ORGANIZATION	STAD	1
c5: Ontology gene sets	GOBP NEGATIVE REGULATION OF EXOCYTOSIS	PCPG	1
c5: Ontology gene sets	GOBP NEGATIVE REGULATION OF DOUBLE STRAND BREAK REPAIR VIA HOMOLOGOUS RECOMBINATION	MESO	1
c5: Ontology gene sets	GOBP NEGATIVE REGULATION OF DNA REPAIR	THYM	1
c5: Ontology gene sets	GOBP NEGATIVE REGULATION OF DNA DAMAGE CHECKPOINT	KIRC	1
c5: Ontology gene sets	GOBP NEGATIVE REGULATION OF CENTROSOME DUPLICATION	MESO	1
c5: Ontology gene sets	GOBP NEGATIVE REGULATION OF CELLULAR RESPONSE TO HYPOXIA	KIRC	1

c5: Ontology gene sets	GOBP NEGATIVE REGULATION OF CELLULAR AMINE METABOLIC PROCESS	LGG	1
c5: Ontology gene sets	GOBP NEGATIVE REGULATION OF CELL SUBSTRATE JUNCTION ORGANIZATION	KIRC	1
c5: Ontology gene sets	GOBP NEGATIVE REGULATION OF CELL DIVISION	KIRC	1
c5: Ontology gene sets	GOBP NEGATIVE REGULATION OF CELL CHEMOTAXIS TO FIBROBLAST GROWTH FACTOR	SKCM	1
c5: Ontology gene sets	GOBP NEGATIVE REGULATION OF CD8 POSITIVE ALPHA BETA T CELL ACTIVATION	LGG	1
c5: Ontology gene sets	GOBP NEGATIVE REGULATION OF B CELL PROLIFERATION	SKCM	1
c5: Ontology gene sets	GOBP NEGATIVE REGULATION OF B CELL APOPTOTIC PROCESS	UVM	1
c5: Ontology gene sets	GOBP NEGATIVE REGULATION OF ARP2 3 COMPLEX MEDIATED ACTIN NUCLEATION	READ	1
c5: Ontology gene sets	GOBP NEGATIVE REGULATION OF ANTIGEN PROCESSING AND PRESENTATION	KIRC	1
c5: Ontology gene sets	GOBP NEGATIVE REGULATION OF ACTIVATED T CELL PROLIFERATION	SKCM	1
c5: Ontology gene sets	GOBP NEGATIVE REGULATION OF ACTIN NUCLEATION	READ	1
c5: Ontology gene sets	GOBP NEGATIVE REGULATION BY HOST OF SYMBIONT MOLECULAR FUNCTION	KIRP	1

c5: Ontology gene sets	GOBP NATURAL KILLER CELL MEDIATED IMMUNE RESPONSE TO TUMOR CELL	CESC	1
c5: Ontology gene sets	GOBP NATURAL KILLER CELL DEGRANULATION	KIRC	1
c5: Ontology gene sets	GOBP NATURAL KILLER CELL CHEMOTAXIS	LGG	1
c5: Ontology gene sets	GOBP NADH OXIDATION	LGG	1
c5: Ontology gene sets	GOBP N ACYLETHANOLAMINE METABOLIC PROCESS	LIHC	1
c5: Ontology gene sets	GOBP MYOFIBROBLAST DIFFERENTIATION	LGG	1
c5: Ontology gene sets	GOBP MYELOID PROGENITOR CELL DIFFERENTIATION	UVM	1
c5: Ontology gene sets	GOBP MYELOID LEUKOCYTE CYTOKINE PRODUCTION	THYM	1
c5: Ontology gene sets	GOBP MULTINUCLEAR OSTEOCLAST DIFFERENTIATION	LUAD	1
c5: Ontology gene sets	GOBP MUCOCILIARY CLEARANCE	BRCA	1
c5: Ontology gene sets	GOBP MRNA PSEUDOURIDINE SYNTHESIS	LGG	1
c5: Ontology gene sets	GOBP MRNA METHYLATION	READ	1
c5: Ontology gene sets	GOBP MRNA CLEAVAGE	THYM	1
c5: Ontology gene sets	GOBP MRNA 3 SPLICE SITE RECOGNITION	LIHC	1
c5: Ontology gene sets	GOBP MOTOR NEURON AXON GUIDANCE	READ	1
c5: Ontology gene sets	GOBP MONOACYLGLYCEROL METABOLIC PROCESS	PAAD	1
c5: Ontology gene sets	GOBP MONOACYLGLYCEROL CATABOLIC PROCESS	PAAD	1

c5: Ontology gene sets	GOBP MODULATION OF AGE RELATED BEHAVIORAL DECLINE	KIRC	1
c5: Ontology gene sets	GOBP MODULATION BY HOST OF VIRAL PROCESS	BRCA	1
c5: Ontology gene sets	GOBP MODULATION BY HOST OF VIRAL GENOME REPLICATION	BRCA	1
c5: Ontology gene sets	GOBP MODULATION BY HOST OF SYMBIONT MOLECULAR FUNCTION	KIRP	1
c5: Ontology gene sets	GOBP MITOTIC G2 DNA DAMAGE CHECKPOINT	KIRC	1
c5: Ontology gene sets	GOBP MITOTIC DNA REPLICATION	LIHC	1
c5: Ontology gene sets	GOBP MITOTIC CELL CYCLE ARREST	UCEC	1
c5: Ontology gene sets	GOBP MITOCHONDRION MORPHOGENESIS	READ	1
c5: Ontology gene sets	GOBP MITOCHONDRIAL TRNA PROCESSING	KIRC	1
c5: Ontology gene sets	GOBP MITOCHONDRIAL TRNA METHYLATION	KIRC	1
c5: Ontology gene sets	GOBP MITOCHONDRIAL RNA MODIFICATION	KIRC	1
c5: Ontology gene sets	GOBP MITOCHONDRIAL RESPIRATORY CHAIN COMPLEX III ASSEMBLY	KIRC	1
c5: Ontology gene sets	GOBP MITOCHONDRIAL GENOME MAINTENANCE	THCA	1
c5: Ontology gene sets	GOBP MITOCHONDRIAL DNA REPAIR	LGG	1
c5: Ontology gene sets	GOBP MITOCHONDRIAL DNA METABOLIC PROCESS	THCA	1

c5: Ontology gene sets	GOBP MITOCHONDRIAL ATP SYNTHESIS COUPLED PROTON TRANSPORT	ACC	1
c5: Ontology gene sets	GOBP MINUS END DIRECTED ORGANELLE TRANSPORT ALONG MICROTUBULE	ACC	1
c5: Ontology gene sets	GOBP MICROTUBULE ANCHORING	THYM	1
c5: Ontology gene sets	GOBP MICROGLIA DIFFERENTIATION	KIRC	1
c5: Ontology gene sets	GOBP MHC PROTEIN COMPLEX ASSEMBLY	LGG	1
c5: Ontology gene sets	GOBP METHYLGLYOXAL METABOLIC PROCESS	SARC	1
c5: Ontology gene sets	GOBP METANEPHRIC S SHAPED BODY MORPHOGENESIS	LGG	1
c5: Ontology gene sets	GOBP METANEPHRIC RENAL VESICLE MORPHOGENESIS	KIRC	1
c5: Ontology gene sets	GOBP METANEPHRIC PART OF URETERIC BUD DEVELOPMENT	LGG	1
c5: Ontology gene sets	GOBP METANEPHRIC MESENCHYME DEVELOPMENT	LUAD	1
c5: Ontology gene sets	GOBP METANEPHRIC GLOMERULUS VASCULATURE DEVELOPMENT	LGG	1
c5: Ontology gene sets	GOBP MESENCHYMAL TO EPITHELIAL TRANSITION INVOLVED IN METANEPHROS MORPHOGENESIS	KIRC	1
c5: Ontology gene sets	GOBP MESENCHYMAL STEM CELL DIFFERENTIATION	LGG	1
c5: Ontology gene sets	GOBP MESENCHYMAL EPITHELIAL CELL SIGNALING	LGG	1
c5: Ontology gene sets	GOBP MESENCHYMAL CELL APOPTOTIC PROCESS	BLCA	1

c5: Ontology gene sets	GOBP MEMBRANE TO MEMBRANE DOCKING	KIRC	1
c5: Ontology gene sets	GOBP MEMBRANE PROTEIN INTRACELLULAR DOMAIN PROTEOLYSIS	THYM	1
c5: Ontology gene sets	GOBP MEMBRANE DEPOLARIZATION DURING SA NODE CELL ACTION POTENTIAL	KIRP	1
c5: Ontology gene sets	GOBP MEMBRANE DEPOLARIZATION DURING AV NODE CELL ACTION POTENTIAL	KIRP	1
c5: Ontology gene sets	GOBP MELANOSOME ASSEMBLY	UCEC	1
c5: Ontology gene sets	GOBP MEIOTIC DNA DOUBLE STRAND BREAK FORMATION	KIRP	1
c5: Ontology gene sets	GOBP MEGAKARYOCYTE DEVELOPMENT	KIRC	1
c5: Ontology gene sets	GOBP MATURATION OF LSU RRNA FROM TRICISTRONIC RRNA TRANSCRIPT SSU RRNA 5 8S RRNA LSU RRNA	READ	1
c5: Ontology gene sets	GOBP MATURATION OF 5 8S RRNA	TGCT	1
c5: Ontology gene sets	GOBP MAST CELL PROLIFERATION	SKCM	1
c5: Ontology gene sets	GOBP MAST CELL DIFFERENTIATION	LUSC	1
c5: Ontology gene sets	GOBP MAMMARY GLAND EPITHELIAL CELL PROLIFERATION	THYM	1
c5: Ontology gene sets	GOBP MAINTENANCE OF DNA METHYLATION	THYM	1
c5: Ontology gene sets	GOBP MAINTENANCE OF APICAL BASAL CELL POLARITY	MESO	1
c5: Ontology gene sets	GOBP MACROPINOCYTOSIS	COAD	1

c5: Ontology gene sets	GOBP MACROMOLECULE DEPALMITOYLATION	LUSC	1
c5: Ontology gene sets	GOBP LYSINE METABOLIC PROCESS	KIRC	1
c5: Ontology gene sets	GOBP LYMPHANGIOGENESIS	THYM	1
c5: Ontology gene sets	GOBP LUTEOLYSIS	GBM	1
c5: Ontology gene sets	GOBP LUNG VASCULATURE DEVELOPMENT	GBM	1
c5: Ontology gene sets	GOBP LIPOXYGENASE PATHWAY	THYM	1
c5: Ontology gene sets	GOBP LIPOXIN METABOLIC PROCESS	BRCA	1
c5: Ontology gene sets	GOBP LEUKOTRIENE SIGNALING PATHWAY	UVM	1
c5: Ontology gene sets	GOBP LEUKEMIA INHIBITORY FACTOR SIGNALING PATHWAY	UVM	1
c5: Ontology gene sets	GOBP LEUCINE IMPORT ACROSS PLASMA MEMBRANE	LUAD	1
c5: Ontology gene sets	GOBP LEUCINE CATABOLIC PROCESS	KIRC	1
c5: Ontology gene sets	GOBP LEPTIN MEDIATED SIGNALING PATHWAY	READ	1
c5: Ontology gene sets	GOBP LEFT RIGHT AXIS SPECIFICATION	STAD	1
c5: Ontology gene sets	GOBP LAYER FORMATION IN CEREBRAL CORTEX	KIRC	1
c5: Ontology gene sets	GOBP LATE ENDOSOME TO LYSOSOME TRANSPORT	BRCA	1
c5: Ontology gene sets	GOBP LATE ENDOSOME TO GOLGI TRANSPORT	ESCA	1
c5: Ontology gene sets	GOBP L SERINE BIOSYNTHETIC PROCESS	KICH	1

c5: Ontology gene sets	GOBP L LYSINE TRANSMEMBRANE TRANSPORT	UVM	1
c5: Ontology gene sets	GOBP L HISTIDINE TRANSMEMBRANE TRANSPORT	MESO	1
c5: Ontology gene sets	GOBP L ARGININE TRANSMEMBRANE TRANSPORT	BRCA	1
c5: Ontology gene sets	GOBP L ARGININE IMPORT ACROSS PLASMA MEMBRANE	LGG	1
c5: Ontology gene sets	GOBP KERATAN SULFATE CATABOLIC PROCESS	DLBC	1
c5: Ontology gene sets	GOBP ISOLEUCINE METABOLIC PROCESS	CESC	1
c5: Ontology gene sets	GOBP ISG15 PROTEIN CONJUGATION	LGG	1
c5: Ontology gene sets	GOBP INTRACILIARY TRANSPORT INVOLVED IN CILIUM ASSEMBLY	DLBC	1
c5: Ontology gene sets	GOBP INTERLEUKIN 7 MEDIATED SIGNALING PATHWAY	SKCM	1
c5: Ontology gene sets	GOBP INTERLEUKIN 4 MEDIATED SIGNALING PATHWAY	LGG	1
c5: Ontology gene sets	GOBP INTERLEUKIN 35 MEDIATED SIGNALING PATHWAY	SKCM	1
c5: Ontology gene sets	GOBP INTERLEUKIN 21 MEDIATED SIGNALING PATHWAY	BRCA	1
c5: Ontology gene sets	GOBP INOSITOL PHOSPHATE BIOSYNTHETIC PROCESS	THCA	1
c5: Ontology gene sets	GOBP INOSITOL METABOLIC PROCESS	COAD	1
c5: Ontology gene sets	GOBP IMP BIOSYNTHETIC PROCESS	THCA	1
c5: Ontology gene sets	GOBP IMMUNE RESPONSE TO TUMOR CELL	THYM	1

c5: Ontology gene sets	GOBP IMMATURE T CELL PROLIFERATION	KIRC	1
c5: Ontology gene sets	GOBP HYPOTHALAMUS CELL DIFFERENTIATION	LGG	1
c5: Ontology gene sets	GOBP HYALURONAN BIOSYNTHETIC PROCESS	KIRC	1
c5: Ontology gene sets	GOBP HOMOSERINE METABOLIC PROCESS	LUSC	1
c5: Ontology gene sets	GOBP HOMOCYSTEINE METABOLIC PROCESS	UCEC	1
c5: Ontology gene sets	GOBP HISTONE THREONINE PHOSPHORYLATION	COAD	1
c5: Ontology gene sets	GOBP HISTONE SERINE PHOSPHORYLATION	KIRC	1
c5: Ontology gene sets	GOBP HISTONE H4 K5 ACETYLATION	PAAD	1
c5: Ontology gene sets	GOBP HISTONE H4 K20 METHYLATION	MESO	1
c5: Ontology gene sets	GOBP HISTONE H4 K12 ACETYLATION	LUAD	1
c5: Ontology gene sets	GOBP HISTONE H3 K9 TRIMETHYLATION	KIRC	1
c5: Ontology gene sets	GOBP HISTONE H3 K4 DEMETHYLATION	TGCT	1
c5: Ontology gene sets	GOBP HISTONE H2A PHOSPHORYLATION	LGG	1
c5: Ontology gene sets	GOBP HISTONE H2A K63 LINKED UBIQUITINATION	LIHC	1
c5: Ontology gene sets	GOBP HISTONE H2A K119 MONOUBIQUITINATION	ACC	1
c5: Ontology gene sets	GOBP HISTAMINE TRANSPORT	COAD	1

c5: Ontology gene sets	GOBP HISTAMINE PRODUCTION INVOLVED IN INFLAMMATORY RESPONSE	SKCM	1
c5: Ontology gene sets	GOBP HEPOXILIN METABOLIC PROCESS	THYM	1
c5: Ontology gene sets	GOBP HEPATOCYTE APOPTOTIC PROCESS	KIRC	1
c5: Ontology gene sets	GOBP HEPARAN SULFATE PROTEOGLYCAN METABOLIC PROCESS	PRAD	1
c5: Ontology gene sets	GOBP HEPARAN SULFATE PROTEOGLYCAN BIOSYNTHETIC PROCESS ENZYMATIC MODIFICATION	LUSC	1
c5: Ontology gene sets	GOBP HEMOGLOBIN BIOSYNTHETIC PROCESS	GBM	1
c5: Ontology gene sets	GOBP HEART FIELD SPECIFICATION	CESC	1
c5: Ontology gene sets	GOBP GTP BIOSYNTHETIC PROCESS	ACC	1
c5: Ontology gene sets	GOBP GROWTH INVOLVED IN HEART MORPHOGENESIS	KIRC	1
c5: Ontology gene sets	GOBP GRANZYME MEDIATED PROGRAMMED CELL DEATH SIGNALING PATHWAY	KIRC	1
c5: Ontology gene sets	GOBP GOLGI DISASSEMBLY	LGG	1
c5: Ontology gene sets	GOBP GLYCOLIPID TRANSPORT	LIHC	1
c5: Ontology gene sets	GOBP GLYCEROL 3 PHOSPHATE METABOLIC PROCESS	LUAD	1
c5: Ontology gene sets	GOBP GLUTATHIONE TRANSMEMBRANE TRANSPORT	BRCA	1
c5: Ontology gene sets	GOBP GLUCOSYLCERAMIDE METABOLIC PROCESS	LUSC	1

c5: Ontology gene sets	GOBP GLUCOSE IMPORT ACROSS PLASMA MEMBRANE	LUAD	1
c5: Ontology gene sets	GOBP GLOMERULUS VASCULATURE MORPHOGENESIS	LGG	1
c5: Ontology gene sets	GOBP GLOMERULAR VISCERAL EPITHELIAL CELL MIGRATION	THCA	1
c5: Ontology gene sets	GOBP GLOMERULAR MESANGIAL CELL DIFFERENTIATION	SARC	1
c5: Ontology gene sets	GOBP GLOMERULAR EPITHELIUM DEVELOPMENT	THCA	1
c5: Ontology gene sets	GOBP GLOMERULAR EPITHELIAL CELL DEVELOPMENT	KIRC	1
c5: Ontology gene sets	GOBP GLOMERULAR BASEMENT MEMBRANE DEVELOPMENT	LGG	1
c5: Ontology gene sets	GOBP GDP METABOLIC PROCESS	PRAD	1
c5: Ontology gene sets	GOBP GAMMA DELTA T CELL ACTIVATION	UVM	1
c5: Ontology gene sets	GOBP G PROTEIN COUPLED PURINERGIC RECEPTOR SIGNALING PATHWAY	BRCA	1
c5: Ontology gene sets	GOBP FRUCTOSE 6 PHOSPHATE METABOLIC PROCESS	READ	1
c5: Ontology gene sets	GOBP FORMATION OF TRANSLATION PREINITIATION COMPLEX	KIRC	1
c5: Ontology gene sets	GOBP FOLLICLE STIMULATING HORMONE SECRETION	GBM	1
c5: Ontology gene sets	GOBP FOLATE IMPORT ACROSS PLASMA MEMBRANE	COAD	1
c5: Ontology gene sets	GOBP FEMALE MEIOTIC NUCLEAR DIVISION	THYM	1
c5: Ontology gene sets	GOBP FATTY ACID BETA OXIDATION USING ACYL COA OXIDASE	KIRC	1

c5: Ontology gene sets	GOBP FATTY ACID BETA OXIDATION USING ACYL COA DEHYDROGENASE	KIRP	1
c5: Ontology gene sets	GOBP EYELID DEVELOPMENT IN CAMERA TYPE EYE	BRCA	1
c5: Ontology gene sets	GOBP EXCITATORY CHEMICAL SYNAPTIC TRANSMISSION	COAD	1
c5: Ontology gene sets	GOBP ESTABLISHMENT OR MAINTENANCE OF BIPOLAR CELL POLARITY	DLBC	1
c5: Ontology gene sets	GOBP ESTABLISHMENT OF PROTEIN LOCALIZATION TO TELOMERE	KICH	1
c5: Ontology gene sets	GOBP ESTABLISHMENT OF PROTEIN LOCALIZATION TO CHROMOSOME	KICH	1
c5: Ontology gene sets	GOBP ESTABLISHMENT OF PROTEIN LOCALIZATION TO CHROMATIN	KIRC	1
c5: Ontology gene sets	GOBP ESTABLISHMENT OF PLANAR POLARITY INVOLVED IN NEURAL TUBE CLOSURE	HNSC	1
c5: Ontology gene sets	GOBP ESTABLISHMENT OF PIGMENT GRANULE LOCALIZATION	DLBC	1
c5: Ontology gene sets	GOBP ESTABLISHMENT OF MITOTIC SPINDLE LOCALIZATION	THYM	1
c5: Ontology gene sets	GOBP ESTABLISHMENT OF GOLGI LOCALIZATION	LIHC	1
c5: Ontology gene sets	GOBP ERYTHROCYTE MATURATION	KICH	1
c5: Ontology gene sets	GOBP ERROR PRONE TRANSLESION SYNTHESIS	PRAD	1
c5: Ontology gene sets	GOBP ER OVERLOAD RESPONSE	THCA	1

c5: Ontology gene sets	GOBP ER ASSOCIATED MISFOLDED PROTEIN CATABOLIC PROCESS	THYM	1
c5: Ontology gene sets	GOBP EPOXIDE METABOLIC PROCESS	DLBC	1
c5: Ontology gene sets	GOBP EPITHELIAL FLUID TRANSPORT	KIRC	1
c5: Ontology gene sets	GOBP EPITHELIAL CILIUM MOVEMENT INVOLVED IN DETERMINATION OF LEFT RIGHT ASYMMETRY	LGG	1
c5: Ontology gene sets	GOBP EPITHELIAL CELL PROLIFERATION INVOLVED IN SALIVARY GLAND MORPHOGENESIS	CESC	1
c5: Ontology gene sets	GOBP EPITHELIAL CELL PROLIFERATION INVOLVED IN PROSTATE GLAND DEVELOPMENT	KIRC	1
c5: Ontology gene sets	GOBP EOSINOPHIL MIGRATION	PRAD	1
c5: Ontology gene sets	GOBP EOSINOPHIL DIFFERENTIATION	BRCA	1
c5: Ontology gene sets	GOBP ENUCLEATE ERYTHROCYTE DIFFERENTIATION	COAD	1
c5: Ontology gene sets	GOBP ENTRY OF BACTERIUM INTO HOST CELL	THYM	1
c5: Ontology gene sets	GOBP ENGULFMENT OF APOPTOTIC CELL	KIRC	1
c5: Ontology gene sets	GOBP ENERGY COUPLED PROTON TRANSMEMBRANE TRANSPORT AGAINST ELECTROCHEMICAL GRADIENT	ACC	1
c5: Ontology gene sets	GOBP ENDOTHELIN RECEPTOR SIGNALING PATHWAY	LUAD	1

c5: Ontology gene sets	GOBP ENDOPLASMIC RETICULUM TUBULAR NETWORK ORGANIZATION	KIRC	1
c5: Ontology gene sets	GOBP ENDOPLASMIC RETICULUM MANNOSE TRIMMING	KIRC	1
c5: Ontology gene sets	GOBP ENDONUCLEOLYTIC CLEAVAGE INVOLVED IN RRNA PROCESSING	KIRP	1
c5: Ontology gene sets	GOBP ENDONUCLEOLYTIC CLEAVAGE IN ITS1 TO SEPARATE SSU RRNA FROM 5 8S RRNA AND LSU RRNA FROM TRICISTRONIC RRNA TRANSCRIPT SSU RRNA 5 8S RRNA LSU RRNA	KIRP	1
c5: Ontology gene sets	GOBP ENDONUCLEOLYTIC CLEAVAGE IN 5 ETS OF TRICISTRONIC RRNA TRANSCRIPT SSU RRNA 5 8S RRNA LSU RRNA	THYM	1
c5: Ontology gene sets	GOBP ENDOCYTIC RECYCLING	DLBC	1
c5: Ontology gene sets	GOBP ENDOCARDIAL CUSHION MORPHOGENESIS	THYM	1
c5: Ontology gene sets	GOBP ENDOCARDIAL CUSHION FORMATION	THYM	1
c5: Ontology gene sets	GOBP ENDOCARDIAL CELL DIFFERENTIATION	THYM	1
c5: Ontology gene sets	GOBP EMBRYONIC SKELETAL JOINT MORPHOGENESIS	KIRC	1
c5: Ontology gene sets	GOBP EMBRYONIC SKELETAL JOINT DEVELOPMENT	KIRC	1
c5: Ontology gene sets	GOBP EMBRYONIC PLACENTA MORPHOGENESIS	THYM	1

c5: Ontology gene sets	GOBP EMBRYONIC NEUROCRANIUM MORPHOGENESIS	UVM	1
c5: Ontology gene sets	GOBP EMBRYONIC DIGIT MORPHOGENESIS	READ	1
c5: Ontology gene sets	GOBP EMBRYONIC DIGESTIVE TRACT DEVELOPMENT	PCPG	1
c5: Ontology gene sets	GOBP EMBRYONIC CLEAVAGE	LIHC	1
c5: Ontology gene sets	GOBP EMBRYONIC CAMERA TYPE EYE FORMATION	KIRC	1
c5: Ontology gene sets	GOBP ELASTIN CATABOLIC PROCESS	ESCA	1
c5: Ontology gene sets	GOBP ECTOPIC GERM CELL PROGRAMMED CELL DEATH	SKCM	1
c5: Ontology gene sets	GOBP DOUBLE STRAND BREAK REPAIR VIA SYNTHESIS DEPENDENT STRAND ANNEALING	KICH	1
c5: Ontology gene sets	GOBP DOUBLE STRAND BREAK REPAIR VIA SINGLE STRAND ANNEALING	THYM	1
c5: Ontology gene sets	GOBP DOUBLE STRAND BREAK REPAIR INVOLVED IN MEIOTIC RECOMBINATION	KIRP	1
c5: Ontology gene sets	GOBP DORSAL AORTA MORPHOGENESIS	KIRC	1
c5: Ontology gene sets	GOBP DORSAL AORTA DEVELOPMENT	KIRC	1
c5: Ontology gene sets	GOBP DNA TOPOLOGICAL CHANGE	PRAD	1
c5: Ontology gene sets	GOBP DNA STRAND RENATURATION	LGG	1
c5: Ontology gene sets	GOBP DNA STRAND ELONGATION	THYM	1
c5: Ontology gene sets	GOBP DNA PROTECTION	KIRC	1

c5: Ontology gene sets	GOBP DNA INTEGRATION	THYM	1
c5: Ontology gene sets	GOBP DNA CYTOSINE DEAMINATION	CESC	1
c5: Ontology gene sets	GOBP DNA CATABOLIC PROCESS EXONUCLEOLYTIC	LGG	1
c5: Ontology gene sets	GOBP DITERPENOID BIOSYNTHETIC PROCESS	COAD	1
c5: Ontology gene sets	GOBP DISTAL TUBULE DEVELOPMENT	KIRC	1
c5: Ontology gene sets	GOBP DISACCHARIDE METABOLIC PROCESS	BLCA	1
c5: Ontology gene sets	GOBP DISACCHARIDE BIOSYNTHETIC PROCESS	BLCA	1
c5: Ontology gene sets	GOBP DIRECT OSSIFICATION	MESO	1
c5: Ontology gene sets	GOBP DIOL METABOLIC PROCESS	THCA	1
c5: Ontology gene sets	GOBP DIADENOSINE POLYPHOSPHATE METABOLIC PROCESS	COAD	1
c5: Ontology gene sets	GOBP DETOXIFICATION OF NITROGEN COMPOUND	BRCA	1
c5: Ontology gene sets	GOBP DETERMINATION OF DORSAL VENTRAL ASYMMETRY	KIRP	1
c5: Ontology gene sets	GOBP DEOXYRIBONUCLEOSIDE TRIPHOSPHATE CATABOLIC PROCESS	BRCA	1
c5: Ontology gene sets	GOBP DEOXYRIBONUCLEOSIDE TRIPHOSPHATE BIOSYNTHETIC PROCESS	LGG	1
c5: Ontology gene sets	GOBP DEOXYRIBONUCLEOSIDE METABOLIC PROCESS	KICH	1
c5: Ontology gene sets	GOBP DENDRITIC CELL APOPTOTIC PROCESS	SKCM	1
c5: Ontology gene sets	GOBP DENDRITIC CELL ANTIGEN PROCESSING AND PRESENTATION	KIRC	1

c5: Ontology gene sets	GOBP DENDRITE ARBORIZATION	LGG	1
c5: Ontology gene sets	GOBP DEFENSE RESPONSE TO TUMOR CELL	UVM	1
c5: Ontology gene sets	GOBP DE NOVO PYRIMIDINE NUCLEOBASE BIOSYNTHETIC PROCESS	MESO	1
c5: Ontology gene sets	GOBP CYTOPLASMIC SEQUESTERING OF NF KAPPAB	SARC	1
c5: Ontology gene sets	GOBP CYSTEINE CATABOLIC PROCESS	LIHC	1
c5: Ontology gene sets	GOBP CORTICOSTERONE SECRETION	MESO	1
c5: Ontology gene sets	GOBP COPPER ION TRANSPORT	KIRC	1
c5: Ontology gene sets	GOBP COPI COATED VESICLE BUDDING	LGG	1
c5: Ontology gene sets	GOBP COMMON PARTNER SMAD PROTEIN PHOSPHORYLATION	KIRC	1
c5: Ontology gene sets	GOBP COBALAMIN TRANSPORT	KIRC	1
c5: Ontology gene sets	GOBP CLEAVAGE FURROW FORMATION	KIRC	1
c5: Ontology gene sets	GOBP CILIARY NEUROTROPHIC FACTOR MEDIATED SIGNALING PATHWAY	LUAD	1
c5: Ontology gene sets	GOBP CHROMATIN MEDIATED MAINTENANCE OF TRANSCRIPTION	KIRC	1
c5: Ontology gene sets	GOBP CHORION DEVELOPMENT	LIHC	1
c5: Ontology gene sets	GOBP CHONDROITIN SULFATE BIOSYNTHETIC PROCESS	THYM	1
c5: Ontology gene sets	GOBP CHONDROCYTE HYPERTROPHY	LUAD	1
c5: Ontology gene sets	GOBP CHOLINE TRANSPORT	UCEC	1

c5: Ontology gene sets	GOBP CHOLINE CATABOLIC PROCESS	KIRC	1
c5: Ontology gene sets	GOBP CHEMOKINE C C MOTIF LIGAND 5 PRODUCTION	HNSC	1
c5: Ontology gene sets	GOBP CHANGES TO DNA METHYLATION INVOLVED IN EMBRYO DEVELOPMENT	MESO	1
c5: Ontology gene sets	GOBP CEREBELLAR GRANULAR LAYER DEVELOPMENT	LUSC	1
c5: Ontology gene sets	GOBP CERAMIDE 1 PHOSPHATE TRANSPORT	THCA	1
c5: Ontology gene sets	GOBP CENTROMERIC SISTER CHROMATID COHESION	PAAD	1
c5: Ontology gene sets	GOBP CENTRIOLE ELONGATION	THYM	1
c5: Ontology gene sets	GOBP CENTRIOLE CENTRIOLE COHESION	THYM	1
c5: Ontology gene sets	GOBP CENTRAL NERVOUS SYSTEM NEURON AXONOGENESIS	READ	1
c5: Ontology gene sets	GOBP CELLULAR RESPONSE TO UV C	LGG	1
c5: Ontology gene sets	GOBP CELLULAR RESPONSE TO STEROL	PRAD	1
c5: Ontology gene sets	GOBP CELLULAR RESPONSE TO PURINE CONTAINING COMPOUND	UCEC	1
c5: Ontology gene sets	GOBP CELLULAR RESPONSE TO PROSTAGLANDIN E STIMULUS	KIRC	1
c5: Ontology gene sets	GOBP CELLULAR RESPONSE TO NITROGEN STARVATION	KICH	1
c5: Ontology gene sets	GOBP CELLULAR RESPONSE TO MAGNESIUM ION	LGG	1
c5: Ontology gene sets	GOBP CELLULAR RESPONSE TO IRON ION	KIRC	1

c5: Ontology gene sets	GOBP CELLULAR RESPONSE TO INSULIN LIKE GROWTH FACTOR STIMULUS	ACC	1
c5: Ontology gene sets	GOBP CELLULAR RESPONSE TO HYPEROXIA	UVM	1
c5: Ontology gene sets	GOBP CELLULAR RESPONSE TO HISTAMINE	LGG	1
c5: Ontology gene sets	GOBP CELLULAR RESPONSE TO HEPARIN	LUSC	1
c5: Ontology gene sets	GOBP CELLULAR RESPONSE TO FRUCTOSE STIMULUS	LIHC	1
c5: Ontology gene sets	GOBP CELLULAR RESPONSE TO ELECTRICAL STIMULUS	UCEC	1
c5: Ontology gene sets	GOBP CELLULAR RESPONSE TO COLD	LGG	1
c5: Ontology gene sets	GOBP CELLULAR RESPONSE TO CISPLATIN	UVM	1
c5: Ontology gene sets	GOBP CELLULAR RESPONSE TO CAFFEINE	UCEC	1
c5: Ontology gene sets	GOBP CELLULAR MODIFIED AMINO ACID CATABOLIC PROCESS	PRAD	1
c5: Ontology gene sets	GOBP CELLULAR METABOLIC COMPOUND SALVAGE	DLBC	1
c5: Ontology gene sets	GOBP CELLULAR HYPOTONIC RESPONSE	COAD	1
c5: Ontology gene sets	GOBP CELLULAR HYPEROSMOTIC SALINITY RESPONSE	UVM	1
c5: Ontology gene sets	GOBP CELL MIGRATION INVOLVED IN KIDNEY DEVELOPMENT	KIRP	1
c5: Ontology gene sets	GOBP CELL DEATH IN RESPONSE TO HYDROGEN PEROXIDE	THYM	1

c5: Ontology gene sets	GOBP CDP DIACYLGLYCEROL BIOSYNTHETIC PROCESS	KIRC	1
c5: Ontology gene sets	GOBP CD8 POSITIVE ALPHA BETA T CELL PROLIFERATION	KIRC	1
c5: Ontology gene sets	GOBP CD8 POSITIVE ALPHA BETA T CELL DIFFERENTIATION	THYM	1
c5: Ontology gene sets	GOBP CARNITINE TRANSPORT	GBM	1
c5: Ontology gene sets	GOBP CARNITINE SHUTTLE	HNSC	1
c5: Ontology gene sets	GOBP CARNITINE METABOLIC PROCESS	KIRC	1
c5: Ontology gene sets	GOBP CARNITINE BIOSYNTHETIC PROCESS	KIRC	1
c5: Ontology gene sets	GOBP CARDIOLIPIN BIOSYNTHETIC PROCESS	KIRC	1
c5: Ontology gene sets	GOBP CARDIAC NEURAL CREST CELL MIGRATION INVOLVED IN OUTFLOW TRACT MORPHOGENESIS	LUAD	1
c5: Ontology gene sets	GOBP CARDIAC EPITHELIAL TO MESENCHYMAL TRANSITION	LUAD	1
c5: Ontology gene sets	GOBP CARBON CATABOLITE REGULATION OF TRANSCRIPTION	KIRC	1
c5: Ontology gene sets	GOBP CANONICAL WNT SIGNALING PATHWAY INVOLVED IN HEART DEVELOPMENT	GBM	1
c5: Ontology gene sets	GOBP CAMP BIOSYNTHETIC PROCESS	LUAD	1
c5: Ontology gene sets	GOBP CALMODULIN DEPENDENT KINASE SIGNALING PATHWAY	KIRC	1
c5: Ontology gene sets	GOBP CALCIUM MEDIATED SIGNALING USING INTRACELLULAR CALCIUM SOURCE	THYM	1

c5: Ontology gene sets	GOBP CALCIUM IMPORT INTO THE MITOCHONDRION	KIRC	1
c5: Ontology gene sets	GOBP C TERMINAL PROTEIN LIPIDATION	DLBC	1
c5: Ontology gene sets	GOBP BRANCHIOMOTOR NEURON AXON GUIDANCE	KIRC	1
c5: Ontology gene sets	GOBP BRANCHING MORPHOGENESIS OF A NERVE	UCEC	1
c5: Ontology gene sets	GOBP BRANCHING INVOLVED IN MAMMARY GLAND DUCT MORPHOGENESIS	KIRC	1
c5: Ontology gene sets	GOBP BRAIN DERIVED NEUROTROPHIC FACTOR RECEPTOR SIGNALING PATHWAY	KIRC	1
c5: Ontology gene sets	GOBP BOX C D SNORNP ASSEMBLY	LIHC	1
c5: Ontology gene sets	GOBP BODY MORPHOGENESIS	DLBC	1
c5: Ontology gene sets	GOBP BMP SIGNALING PATHWAY INVOLVED IN HEART DEVELOPMENT	KIRC	1
c5: Ontology gene sets	GOBP BLEB ASSEMBLY	LUAD	1
c5: Ontology gene sets	GOBP BLASTODERM SEGMENTATION	PCPG	1
c5: Ontology gene sets	GOBP BENZENE CONTAINING COMPOUND METABOLIC PROCESS	BRCA	1
c5: Ontology gene sets	GOBP BEHAVIORAL RESPONSE TO PAIN	PRAD	1
c5: Ontology gene sets	GOBP BEHAVIORAL RESPONSE TO COCAINE	DLBC	1
c5: Ontology gene sets	GOBP B CELL PROLIFERATION INVOLVED IN IMMUNE RESPONSE	SKCM	1

c5: Ontology gene sets	GOBP B CELL LINEAGE COMMITMENT	THYM	1
c5: Ontology gene sets	GOBP B CELL HOMEOSTASIS	THYM	1
c5: Ontology gene sets	GOBP AXONEMAL CENTRAL APPARATUS ASSEMBLY	LGG	1
c5: Ontology gene sets	GOBP AXONAL TRANSPORT OF MITOCHONDRION	UCEC	1
c5: Ontology gene sets	GOBP AV NODE CELL TO BUNDLE OF HIS CELL COMMUNICATION	KIRP	1
c5: Ontology gene sets	GOBP AUTOPHAGY OF PEROXISOME	THYM	1
c5: Ontology gene sets	GOBP AUTOPHAGIC CELL DEATH	LGG	1
c5: Ontology gene sets	GOBP ATRIOVENTRICULAR VALVE FORMATION	KIRC	1
c5: Ontology gene sets	GOBP ATRIAL CARDIAC MUSCLE TISSUE DEVELOPMENT	DLBC	1
c5: Ontology gene sets	GOBP ATF6 MEDIATED UNFOLDED PROTEIN RESPONSE	UVM	1
c5: Ontology gene sets	GOBP ASYMMETRIC CELL DIVISION	KIRC	1
c5: Ontology gene sets	GOBP ASPARAGINE METABOLIC PROCESS	KIRP	1
c5: Ontology gene sets	GOBP ARTERY SMOOTH MUSCLE CONTRACTION	CESC	1
c5: Ontology gene sets	GOBP ARGININE TRANSMEMBRANE TRANSPORT	BRCA	1
c5: Ontology gene sets	GOBP ARACHIDONIC ACID METABOLIC PROCESS	DLBC	1
c5: Ontology gene sets	GOBP APOPTOTIC PROCESS INVOLVED IN HEART MORPHOGENESIS	LGG	1
c5: Ontology gene sets	GOBP APOPTOTIC CELL CLEARANCE	DLBC	1

c5: Ontology gene sets	GOBP ANTIVIRAL INNATE IMMUNE RESPONSE	SKCM	1
c5: Ontology gene sets	GOBP ANTIGEN PROCESSING AND PRESENTATION OF ENDOGENOUS PEPTIDE ANTIGEN VIA MHC CLASS I VIA ER PATHWAY	MESO	1
c5: Ontology gene sets	GOBP ANTEROGRADE AXONAL PROTEIN TRANSPORT	KIRC	1
c5: Ontology gene sets	GOBP AMINO SUGAR CATABOLIC PROCESS	DLBC	1
c5: Ontology gene sets	GOBP AMINO ACID BETAINE METABOLIC PROCESS	KIRC	1
c5: Ontology gene sets	GOBP AMINO ACID BETAINE BIOSYNTHETIC PROCESS	KIRC	1
c5: Ontology gene sets	GOBP ALDITOL CATABOLIC PROCESS	LUAD	1
c5: Ontology gene sets	GOBP AGGRESSIVE BEHAVIOR	HNSC	1
c5: Ontology gene sets	GOBP ADIPONECTIN ACTIVATED SIGNALING PATHWAY	KICH	1
c5: Ontology gene sets	GOBP ADHERENS JUNCTION MAINTENANCE	KIRC	1
c5: Ontology gene sets	GOBP ACYL CARNITINE TRANSPORT	BRCA	1
c5: Ontology gene sets	GOBP ACTOMYOSIN CONTRACTILE RING ORGANIZATION	KIRP	1
c5: Ontology gene sets	GOBP ACTIVATION OF PROTEIN KINASE C ACTIVITY	LUAD	1
c5: Ontology gene sets	GOBP ACTIVATION OF JNKK ACTIVITY	COAD	1
c5: Ontology gene sets	GOBP ACTIVATION OF CYSTEINE TYPE ENDOPEPTIDASE ACTIVITY	SKCM	1
c5: Ontology gene sets	GOBP ACTIN MODIFICATION	MESO	1

c5: Ontology gene sets	GOBP ACTIN FILAMENT REORGANIZATION	BRCA	1
c5: Ontology gene sets	GOBP ABSCISSION	KIRP	1
c5: Ontology gene sets	GOBP 5S CLASS RRNA TRANSCRIPTION BY RNA POLYMERASE III	LIHC	1
c4: computational gene sets	GNF2 TTN	HNSC	1
c4: computational gene sets	GNF2 SMC2L1	LUAD	1
c4: computational gene sets	GNF2 SMC1L1	THYM	1
c4: computational gene sets	GNF2 SERPINB5	PAAD	1
c4: computational gene sets	GNF2 RRM2	LUAD	1
c4: computational gene sets	GNF2 MSH2	LIHC	1
c4: computational gene sets	GNF2 MATK	UVM	1
c4: computational gene sets	GNF2 JAK1	THCA	1
c4: computational gene sets	GNF2 ICAM3	CESC	1
c4: computational gene sets	GNF2 ESPL1	LUAD	1
c4: computational gene sets	GNF2 DENR	TGCT	1
c4: computational gene sets	GNF2 DEK	TGCT	1
c4: computational gene sets	GNF2 CKS1B	LUAD	1
c4: computational gene sets	GNF2 CENPE	LUAD	1
c4: computational gene sets	GNF2 CASP8	PCPG	1
c4: computational gene sets	GNF2 BUB3	LIHC	1

c4: computational gene sets	GNF2 APEX1	TGCT	1
c6: oncogenic signature gene sets	GLI1 UP.V1 UP	THYM	1
c4: computational gene sets	GCM PTPRD	DLBC	1
c4: computational gene sets	GCM MAX	THYM	1
c4: computational gene sets	GCM FANCL	LIHC	1
c2: curated gene sets	GAZIN EPIGENETIC SILENCING BY KRAS	THYM	1
c2: curated gene sets	GAVIN IL2 RESPONSIVE FOXP3 TARGETS UP	KIRC	1
c2: curated gene sets	GAVIN IL2 RESPONSIVE FOXP3 TARGETS DN	SARC	1
c2: curated gene sets	GAUSSMANN MLL AF4 FUSION TARGETS D DN	LGG	1
c7: immunologic signature gene sets	GAUCHER PBMC YF VAX STAMARIL UNKNOWN AGE 14DY DN	SKCM	1
c2: curated gene sets	GARGALOVIC RESPONSE TO OXIDIZED PHOSPHOLIPIDS LIGHTYELLOW UP	THYM	1
c2: curated gene sets	GARGALOVIC RESPONSE TO OXIDIZED PHOSPHOLIPIDS GREEN DN	THYM	1
c2: curated gene sets	GALI TP53 TARGETS APOPTOTIC DN	LGG	1
c2: curated gene sets	GAJATE RESPONSE TO TRABECTEDIN DN	THCA	1
c2: curated gene sets	FUNG IL2 TARGETS WITH STAT5 BINDING SITES	KIRC	1
c7: immunologic signature gene sets	FULLER PBMC F TULARENSIS VACCINE LVS AGE 22 54YO 18HR TO 336HR SUSTAINED UP	THYM	1

c2: curated gene sets	FRASOR TAMOXIFEN RESPONSE DN	KIRC	1
c2: curated gene sets	FRASOR RESPONSE TO SERM OR FULVESTRANT UP	THYM	1
c7: immunologic signature gene sets	FRANCO BLOOD SANOFI PASTEUR SA INACTIVATED INFLUENZA VACCINE CORRELATED WITH ANTIBODY RESPONSE AGE 18 40YO 14DY POSITIVE	BLCA	1
c3: regulatory target gene sets	FOXF2 TARGET GENES	LIHC	1
c2: curated gene sets	FOURNIER ACINAR DEVELOPMENT EARLY DN	BLCA	1
c7: immunologic signature gene sets	FOURATI BLOOD TWINRIX AGE 65 83YO POOR RESPONDERS VS RESPONDERS 0DY NETWORK INFERENCE UP	SKCM	1
c7: immunologic signature gene sets	FOURATI BLOOD TWINRIX AGE 65 81YO RESPONDERS VS POOR RESPONDERS TRAINING SET 0DY NETWORK INFERENCE DN	MESO	1
c7: immunologic signature gene sets	FOURATI BLOOD TWINRIX AGE 65 81Y0 RESPONDERS VS POOR RESPONDERS TRAINING SET 0DY NETWORK INFERENCE UP	KIRC	1
c2: curated gene sets	FIGUEROA AML METHYLATION CLUSTER 2 DN	THCA	1
c2: curated gene sets	FERRANDO LYL1 NEIGHBORS	KIRC	1
c2: curated gene sets	FARMER BREAST CANCER CLUSTER 4	LGG	1
c8: cell type signature gene sets	FAN EMBRYONIC CTX BIG GROUPS INHIBITORY	BLCA	1

c3: regulatory target gene sets	F2RL1 TARGET GENES	PAAD	1
c8: cell type signature gene sets	DURANTE ADULT OLFACTORY NEUROEPITHELIUM PLASMA CELLS	UVM	1
c2: curated gene sets	DISTECHE ESCAPED FROM X INACTIVATION	KIRC	1
c8: cell type signature gene sets	DESCARTES FETAL THYMUS THYMOCYTES	BRCA	1
c2: curated gene sets	DER IFN GAMMA RESPONSE DN	LGG	1
c2: curated gene sets	DEMAGALHAES AGING DN	PCPG	1
c2: curated gene sets	DAVIES MULTIPLE MYELOMA VS MGUS UP	LUAD	1
c2: curated gene sets	DACOSTA LOW DOSE UV RESPONSE VIA ERCC3 XPCS DN	UVM	1
c8: cell type signature gene sets	CUI DEVELOPING HEART RIGHT ATRIAL CARDIOMYOCYTE	DLBC	1
c8: cell type signature gene sets	CUI DEVELOPING HEART COMPACT ATRIAL CARDIOMYOCYTE	KIRP	1
c2: curated gene sets	COLIN PILOCYTIC ASTROCYTOMA VS GLIOBLASTOMA DN	DLBC	1
c7: immunologic signature gene sets	COLE BLOOD FLUZONE FLUARIX AGE 03 17YO 7DY DN	SKCM	1
c2: curated gene sets	CLIMENT BREAST CANCER COPY NUMBER DN	KIRC	1
c2: curated gene sets	CLAUS PGR POSITIVE MENINGIOMA UP	DLBC	1
c1: positional gene sets	chr3p23	CESC	1
c2: curated gene sets	CHIARETTI T ALL RELAPSE PROGNOSIS	THYM	1
c2: curated gene sets	CHESLER BRAIN HIGHEST GENETIC VARIANCE	PRAD	1

c2: curated gene sets	CHEOK RESPONSE TO HD MTX UP	DLBC	1
c2: curated gene sets	CHEN LUNG CANCER SURVIVAL	PRAD	1
c2: curated gene sets	CHEN HOXA5 TARGETS 6HR UP	LGG	1
c2: curated gene sets	CHEN HOXA5 TARGETS 6HR DN	KIRC	1
c2: curated gene sets	CHASSOT SKIN WOUND	OV	1
c3: regulatory target gene sets	CEBPE TARGET GENES	TGCT	1
c2: curated gene sets	CASTELLANO NRAS TARGETS DN	KIRP	1
c2: curated gene sets	CASTELLANO HRAS TARGETS UP	ACC	1
c7: immunologic signature gene sets	CAO BLOOD FLUZONE AGE 05 14YO 7DY DN	LUAD	1
c7: immunologic signature gene sets	CAO BLOOD FLUZONE AGE 05 14YO 30DY DN	BRCA	1
c7: immunologic signature gene sets	CAO BLOOD FLUZONE AGE 05 14YO 1DY UP	SKCM	1
c7: immunologic signature gene sets	CAO BLOOD FLUMIST AGE 05 14YO 1DY DN	LUAD	1
c2: curated gene sets	CALVET IRINOTECAN SENSITIVE VS REVERTED DN	LGG	1
c2: curated gene sets	CAFFAREL RESPONSE TO THC UP	READ	1
c2: curated gene sets	CAFFAREL RESPONSE TO THC 8HR 3 DN	DLBC	1
c2: curated gene sets	CAFFAREL RESPONSE TO THC 24HR 3 UP	KIRP	1
c3: regulatory target gene sets	CACGTTT MIR302A	THYM	1
c8: cell type signature gene sets	BUSSLINGER GASTRIC TUFT CELLS	BRCA	1
c2: curated gene sets	BURTON ADIPOGENESIS 12	THYM	1
c2: curated gene sets	BRUNEAU SEPTATION ATRIAL	KIRP	1
c2: curated gene sets	BROWNE HCMV INFECTION 1HR UP	DLBC	1

c2: curated gene sets	BOYAULT LIVER CANCER SUBCLASS G12 UP	READ	1
c2: curated gene sets	BOWIE RESPONSE TO TAMOXIFEN	SKCM	1
c2: curated gene sets	BIOCARTA WNT LRP6 PATHWAY	BRCA	1
c2: curated gene sets	BIOCARTA TRKA PATHWAY	KIRC	1
c2: curated gene sets	BIOCARTA TOB1 PATHWAY	SKCM	1
c2: curated gene sets	BIOCARTA TH1TH2 PATHWAY	UVM	1
c2: curated gene sets	BIOCARTA TERT PATHWAY	LGG	1
c2: curated gene sets	BIOCARTA STATHMIN PATHWAY	UVM	1
c2: curated gene sets	BIOCARTA SRCRPTP PATHWAY	KIRC	1
c2: curated gene sets	BIOCARTA SODD PATHWAY	LGG	1
c2: curated gene sets	BIOCARTA SLRP PATHWAY	KIRC	1
c2: curated gene sets	BIOCARTA SARS PATHWAY	KICH	1
c2: curated gene sets	BIOCARTA SALMONELLA PATHWAY	SARC	1
c2: curated gene sets	BIOCARTA RHO PATHWAY	KIRC	1
c2: curated gene sets	BIOCARTA REELIN PATHWAY	KIRC	1
c2: curated gene sets	BIOCARTA RECK PATHWAY	GBM	1
c2: curated gene sets	BIOCARTA RB PATHWAY	STAD	1
c2: curated gene sets	BIOCARTA RARRXR PATHWAY	SKCM	1
c2: curated gene sets	BIOCARTA RANKL PATHWAY	OV	1
c2: curated gene sets	BIOCARTA RAB PATHWAY	KIRC	1
c2: curated gene sets	BIOCARTA PS1 PATHWAY	KIRC	1
c2: curated gene sets	BIOCARTA PRION PATHWAY	KIRC	1
c2: curated gene sets	BIOCARTA PPARG PATHWAY	KIRC	1
c2: curated gene sets	BIOCARTA PML PATHWAY	KIRC	1
c2: curated gene sets	BIOCARTA NTHI PATHWAY	THYM	1
c2: curated gene sets	BIOCARTA NPC PATHWAY	KIRC	1
c2: curated gene sets	BIOCARTA NOTCH PATHWAY	LUAD	1
c2: curated gene sets	BIOCARTA NKT PATHWAY	SKCM	1
c2: curated gene sets	BIOCARTA NEUTROPHIL PATHWAY	BRCA	1
c2: curated gene sets	BIOCARTA MRP PATHWAY	SKCM	1

c2: curated gene sets	BIOCARTA MEF2D PATHWAY	SKCM	1
c2: curated gene sets	BIOCARTA MCM PATHWAY	KIRC	1
c2: curated gene sets	BIOCARTA LYM PATHWAY	BRCA	1
c2: curated gene sets	BIOCARTA INTEGRIN PATHWAY	DLBC	1
c2: curated gene sets	BIOCARTA INSULIN PATHWAY	PCPG	1
c2: curated gene sets	BIOCARTA IL7 PATHWAY	SKCM	1
c2: curated gene sets	BIOCARTA IL6 PATHWAY	READ	1
c2: curated gene sets	BIOCARTA IL4 PATHWAY	SKCM	1
c2: curated gene sets	BIOCARTA IL22BP PATHWAY	BRCA	1
c2: curated gene sets	BIOCARTA IL10 PATHWAY	KIRC	1
c2: curated gene sets	BIOCARTA IGF1MTOR PATHWAY	DLBC	1
c2: curated gene sets	BIOCARTA HBX PATHWAY	KIRC	1
c2: curated gene sets	BIOCARTA GRANULOCYTES PATHWAY	SKCM	1
c2: curated gene sets	BIOCARTA GLEEVEC PATHWAY	PCPG	1
c2: curated gene sets	BIOCARTA GH PATHWAY	PCPG	1
c2: curated gene sets	BIOCARTA FIBRINOLYSIS PATHWAY	STAD	1
c2: curated gene sets	BIOCARTA ERK5 PATHWAY	LIHC	1
c2: curated gene sets	BIOCARTA EIF2 PATHWAY	LIHC	1
c2: curated gene sets	BIOCARTA EICOSANOID PATHWAY	BRCA	1
c2: curated gene sets	BIOCARTA CPSF PATHWAY	LIHC	1
c2: curated gene sets	BIOCARTA CIRCADIAN PATHWAY	KIRC	1
c2: curated gene sets	BIOCARTA CHEMICAL PATHWAY	KIRC	1
c2: curated gene sets	BIOCARTA CBL PATHWAY	KIRC	1
c2: curated gene sets	BIOCARTA BTG2 PATHWAY	KIRC	1
c2: curated gene sets	BIOCARTA BOTULIN PATHWAY	LGG	1
c2: curated gene sets	BIOCARTA BARD1 PATHWAY	LIHC	1
c2: curated gene sets	BIOCARTA ARF PATHWAY	ACC	1
c2: curated gene sets	BIOCARTA AKT PATHWAY	THYM	1
c2: curated gene sets	BILD SRC ONCOGENIC SIGNATURE	DLBC	1

c2: curated gene sets	BIERIE INFLAMMATORY RESPONSE TGFB1	ACC	1
c2: curated gene sets	BEIER GLIOMA STEM CELL UP	CHOL	1
c2: curated gene sets	BARRIER CANCER RELAPSE TUMOR SAMPLE DN	LGG	1
c2: curated gene sets	BANDRES RESPONSE TO CARMUSTIN WITHOUT MGMT 48HR DN	READ	1
c2: curated gene sets	BANDRES RESPONSE TO CARMUSTIN WITHOUT MGMT 24HR DN	ACC	1
c2: curated gene sets	BANDRES RESPONSE TO CARMUSTIN MGMT 24HR UP	LIHC	1
c2: curated gene sets	BALLIF DEVELOPMENTAL DISABILITY P16 P12 DELETION	READ	1
c2: curated gene sets	BAKER HEMATOPOIESIS STAT1 TARGETS	THCA	1
c3: regulatory target gene sets	ATGCACG MIR517B	READ	1
c2: curated gene sets	AMIT EGF RESPONSE 480 MCF10A	READ	1
c8: cell type signature gene sets	AIZARANI LIVER C8 RESIDENT B CELLS 1	THYM	1
c8: cell type signature gene sets	AIZARANI LIVER C38 RESIDENT B CELLS 3	GBM	1
c3: regulatory target gene sets	AGTGCGT MIR521	THYM	1
c3: regulatory target gene sets	AGTCTAG MIR151	THCA	1
c3: regulatory target gene sets	AGCGCAG MIR191	KIRC	1
c3: regulatory target gene sets	AACGGTT MIR451	COAD	1
